# The loci recommended as universal barcodes for plants on the basis of floristic studies may not work with congeneric species as exemplified by DNA barcoding of *Dendrobium *species

**DOI:** 10.1186/1756-0500-5-42

**Published:** 2012-01-19

**Authors:** Hemant Kumar Singh, Iffat Parveen, Saurabh Raghuvanshi, Shashi B Babbar

**Affiliations:** 1 Department of Botany, University of Delhi, Delhi, India; 2 Department of Plant Molecular Biology, University of Delhi South Campus, New Delhi, India

**Keywords:** *Dendrobium*, DNA barcoding, ITS, *matK*

## Abstract

**Background:**

Based on the testing of several loci, predominantly against floristic backgrounds, individual or different combinations of loci have been suggested as possible universal DNA barcodes for plants. The present investigation was undertaken to check the applicability of the recommended locus/loci for congeneric species with *Dendrobium *species as an illustrative example.

**Results:**

Six loci, *matK, rbcL, rpoB, rpoC1, trnH-psbA *spacer from the chloroplast genome and ITS, from the nuclear genome, were compared for their amplification, sequencing and species discrimination success rates among multiple accessions of 36 *Dendrobium *species. The *trnH-psbA *spacer could not be considered for analysis as good quality sequences were not obtained with its forward primer. Among the tested loci, ITS, recommended by some as a possible barcode for plants, provided 100% species identification. Another locus, *matK*, also recommended as a universal barcode for plants, resolved 80.56% species. ITS remained the best even when sequences of investigated loci of additional *Dendrobium *species available on the NCBI GenBank (93, 33, 20, 18 and 17 of ITS, *matK, rbcL, rpoB *and *rpoC1*, respectively) were also considered for calculating the percent species resolution capabilities. The species discrimination of various combinations of the loci was also compared based on the 36 investigated species and additional 16 for which sequences of all the five loci were available on GenBank. Two-locus combination of *matK+rbcL *recommended by the Plant Working Group of Consortium for Barcoding of Life (CBOL) could discriminate 86.11% of 36 species. The species discriminating ability of this barcode was reduced to 80.77% when additional sequences available on NCBI were included in the analysis. Among the recommended combinations, the barcode based on three loci - *matK, rpoB *and *rpoC1*- resolved maximum number of species.

**Conclusions:**

Any recommended barcode based on the loci tested so far, is not likely to provide 100% species identification across the plant kingdom and thus is not likely to act as a universal barcode. It appears that barcodes, if based on single or limited locus(i), would be taxa specific as is exemplified by the success of ITS among *Dendrobium *species, though it may not be suitable for other plants because of the problems that are discussed.

## Background

DNA barcoding is an emerging technology, which has been projected as a powerful species level identification tool. Hebert et al. [1,2] proposed that sequence from a small standardized region of the genome could serve as a species recognition tag. Thus, an unidentified organism or tissue could be ascribed to a species when such a sequence from it is compared with those available in a database that is intended to possess sequences of the standardized region of almost all the organisms on the planet Earth [1]. However, if the DNA sequence from unidentified organism/tissue fails to match with any of the reference sequences, the specimen would be flagged as a possible new species, requiring a detailed study. Thus, besides providing a rapid identification tool, utilizing only minute amount of tissue from any stage of development of a plant or animal, DNA barcoding could also enhance discovery of new species [3,4]. DNA barcodes could also be used (i) for rapid inventorization of biodiversity [5], (ii) as genetic resource tags for species [6], (iii) for the identification of cryptic and polymorphic species [4,7-9], (iv) in linking different stages of life cycle in difficult to identify taxa [10], (v) for checking the herbal formulations and food stuffs for adulteration and/or substitution [11-13], (vi) in forensic investigations [14], (vii) in controlling plant invasions by identifying the propagules of invasive species right at quarantine stage [15], (viii) in tackling illegal trade of endangered species of both plants and animals [6,16,17] and (ix) in identifying complex food webs by analyzing the DNA in the gut contents of animals [18,19]. In animals, the applicability of this technique has been amply demonstrated through the use of a short fragment at the 5' end of the mitochondrial cytochrome *c *oxidase 1 (*CO1*) gene, known as Folmer region [1-4]. However, usefulness of a comparable sequence is yet to be established for plants. A number of loci from the plastid genome, including *rbcL, rpoB, rpoC1, trnH-psbA *spacer and *matK*, have been tested for DNA barcoding of plants with different degrees of success [5,20-32]. So far, no consensus has emerged for a universal barcode for land plants. However, realization among majority is that, for accurate and reproducible species identification, use of more than one locus would be required [5,20-23,25-27]. Thus, Chase et al. [33] proposed that a combination of three loci may be needed for species level identification in plants. Two combinations suggested by them were, *rpoC1, rpoB, matK *and *rpoC1, matK, trnH-psbA*. On the contrary, Kress and Erickson [23] proposed a two-locus global DNA barcode, consisting of coding *rbcL *and non-coding *trnH-psbA *spacer region for land plants. Lahaye et al. [5] based on the study of more than 1000 plants, predominantly orchids, recommended that a small region of plastid *matK *gene could be effectively employed as a universal barcode. CBOL Plant Working Group [27] recommended two-locus combination of *matK*+*rbcL *as the plant barcode, though success of species discrimination using this combination was limited to 72%. The internal transcribed spacer (ITS) region of the nuclear ribosomal cistron (18S-5.8S-26S) has also been suggested as a possible plant barcode by some groups [11,20,22]. The second internal transcribed spacer (ITS2) exhibited a discrimination ability of 92.7% at species level in more than 6600 plant samples, belonging to 4800 species from 753 distinct genera [11]. One conclusion that appears to be emerging from these reports is that the multi-locus barcode which may afford maximum species resolution would most likely be from four loci - ITS, *matK, rbcL *and *trnH-psbA *spacer, notwithstanding a recent report where 16 Indian species of *Berberis *could not be resolved based on the sequence comparison of all these four loci, either individually or in combination [34]. However, the same group observed 100% species resolution for four species of *Gossypium *and 11 species of *Ficus *on the basis of only the ITS sequences [34]. Recently, the use of complete sequences of the chloroplast genomes, obtained by cost-effective massively parallel sequencing (MPS), has been suggested as a single locus barcode for identification and establishing phylogenetic relationships of species [35].

Most of the studies related to DNA barcoding have been carried out with a floristic backdrop where the species were not necessarily closely related. However, to assess the ability of different target loci to discriminate species, the investigations should include maximum number of species from each genus as was highlighted by Seberg and Petersen [26] who studied 98% of the known species in the genus *Crocus*. In the present investigation too, four loci from the chloroplast genome (*rbcL, rpoC1, rpoB *and *matK*) and one locus from the nuclear genome (nuclear ribosomal ITS) were assessed for their intra- and inter-specific divergences, either individually or in various combinations, to determine their suitability for the resolution of congeneric species of *Dendrobium *Sw. (Orchidaceae). Many species of the genus *Dendrobium *have long been used in commercial production of cut flowers. In Asian countries, many *Dendrobium *species, owing to their diverse therapeutic properties, are also utilized in traditional medicine [36]. Because of its high commercial value the genus *Dendrobium *was chosen for this study. Another advantage was the availability of complete sequence of chloroplast genome of the orchid, *Phalaenopsis aphrodite *[37] that helped in designing of the primers for amplifying the targeted regions.

## Results and discussion

### Amplification and sequencing success

Among six tested loci, ITS, *rbcL, rpoB *and *rpoC1 *upon amplification yielded single band. Whereas, in few samples of *matK *and all of *trnH-psbA *spacer amplification resulted in multiple bands, from which the band having molecular weight nearest to the targeted one was purified using gel extraction. Despite many attempts utilizing multiple samples of 33 species good quality sequences of *trnH*-*psbA *spacer with forward primer were not obtained. Therefore, this locus was not considered for further analysis.

Among the loci tested, *rpoC1 *exhibited highest (100%) amplification success rate, followed by *matK *(99.32%), *rpoB *(99.2%), ITS (98.97%) and *rbcL *(96.91%). The number of finished sequences obtained from the PCR products for ITS, *matK, rbcL, rpoB *and *rpoC1 *were 269, 267, 174, 232 and 238, respectively. Their respective sequencing success rates were 93.08%, 92.07%, 92.55%, 93.93% and 95.58% (Table 1).

**Table 1:** Amplification and sequencing success rates of the five candidate loci for 36 *Dendrobium *species

Locus	No. of samples used for PCR Amplification	No. of amplicons obtained	Amplification success	No. of finished sequences generated	Sequencing success
**ITS**	292	289	98.97%	269	93.08%

***matK***	292	290	99.32%	267	92.07%

***rbcL***	194	188	96.91%	174	92.55%

***rpoB***	249	247	99.2%	232	93.93%

***rpoC1***	249	249	100%	238	95.58%

To ascertain that the ITS sequences generated in the present study and those down loaded from the GenBank were only of *Dendrobium *species and not of contaminations of fungi or from the host tissue (*Dendrobium *generally being epiphytic), a BLAST analysis for sequences of each of the tested species (both self generated and downloaded sequences) was performed. It was observed that all the sequences closely matched with only *Dendrobium *species (see Additional file 1).

### Data set I: analyses based on 36 self collected/procured species

#### Determination of intra- and inter-specific variation

The computational analysis pertaining to intra-specific sequence divergence, as calculated by MEGA 4.0 software [38] with Kimura-2-Parameter (K2P) distance method for 33 *Dendrobium *species (a total of 289 accessions for 33 species excluding three of rest of the species having only one accession each), revealed that there was no intra-specific variation in *rbcL, rpoC1 *and *rpoB *(Table 2). On the other hand, *matK *and ITS exhibited intra-specific distances up to 0.0015 and 0.0101, respectively. Average inter-specific K2P distances for ITS, *matK, rbcL, rpoB *and *rpoC1 *among 36 species (based on 292 accessions) were 0.1714 (0.0152 - 0.3251), 0.0126 (0 - 0.0371), 0.0061 (0 - 0.023), 0.0077 (0 - 0.0296) and 0.0042 (0 - 0.0171), respectively (Table 2) [see Additional file 2(a)-(e)].

**Table 2:** Intra- and inter-specific K2P distances of the five candidate loci for 36 *Dendrobium *species

Locus	No. of sp. used for intra-specific distance	Intra-specific K2P distance	No. of sp. used for inter-specific distance	Average inter-specific K2P distance (Range)
**ITS**	33	0 - 0.0101	36	0.1714 (0.0152 - 0.3251)

***matK***	33	0 - 0.0015	36	0.0126 (0 - 0.0371)

***rbcL***	33	0	36	0.0061 (0 - 0.023)

***rpoB***	33	0	36	0.0077 (0 - 0.0296)

***rpoC1***	33	0	36	0.0042 (0 - 0.0171)

#### Determination of species resolution

**Figure 1:**
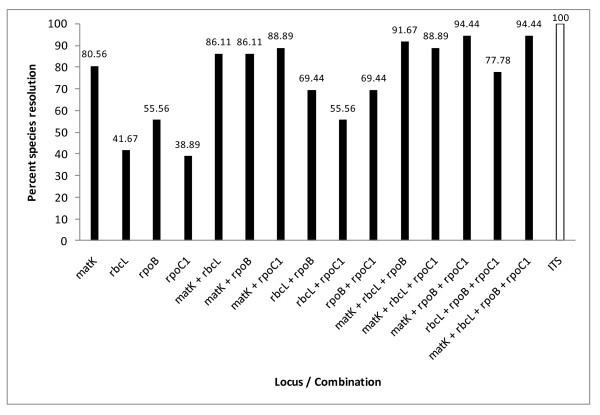
**Analysis of 36 species using single and multi-locus combinations**. Percent species resolution, based on single as well as multi-locus combinations for 36 species of *Dendrobium*

Among the tested loci, ITS provided 100% species resolution, whereas for other loci different number of species pairs with distance estimate of zero were obtained. These were minimum with *matK *and maximum with *rpoC1 *(Table 3). The species identification success rates or percent species resolution for *matK, rbcL, rpoB *and *rpoC1 *were 80.56%, 41.67%, 55.56% and 38.89%, respectively (Table 3). Two-locus, three-locus and four-locus combinations involving *matK, rbcL, rpoB*, and *rpoC1 *were also tested for their ability to discriminate investigated species. The two-locus combinations of, *matK*+*rbcL, matK*+*rpoB, matK*+*rpoC1, rbcL*+*rpoB, rbcL*+*rpoC1 *and *rpoB*+*rpoC1 *exhibited species resolution of 86.11%, 86.11%, 88.89%, 69.44%, 55.56% and 69.44%, respectively. Among three-locus combinations *matK*+*rpoB*+*rpoC1 *could discriminate 94.44% (34 out of 36) species, the rest viz., *matK*+*rbcL*+*rpoB, matK*+*rbcL*+*rpoC1 *and *rbcL*+*rpoB*+*rpoC1 *provided 91.67%, 88.89% and 77.78% species resolution, respectively. Even four-locus combination (*matK*+*rbcL*+*rpoB*+*rpoC1*) could not resolve beyond 94.44% (34 out of 36) species (Figure 1).

**Table 3:** Analysis of species in Data Set I. Percent species resolution of 36 species of *Dendrobium*, based on each of the five candidate loci

Locus	No. of species analyzed (A)	No. of species pairs with zero distance estimate	No. of species with zero distance estimate (B)	No. of species successfully discriminated (C = A-B)	% Species resolution (C/A) × 100
**ITS**	36	0	0	36	100.00

***matK***	36	5	7	29	80.56

***rbcL***	36	32	21	15	41.67

***rpoB***	36	48	16	20	55.56

***rpoC1***	36	140	22	14	38.89

A species pair, *D. macrostachyum *Lindl.*/D. aphyllum *(Roxb.) C.E.C. Fisch. had zero distance estimates with all the chloroplast loci [see Additional file 2(b) - (e)]. However, when compared for ITS, the inter-specific K2P distance was 0.0172 [see Additional file 2(a)], thus indicating that the two species are distinct. These two species look quite similar in their vegetative phase. However, at the flowering stage two are easily distinguishable because of the color of their flowers as the former produces pink flowers in contrast to pale green flowers of the latter (see Additional file 3). The inability of the loci from chloroplast genome in resolving the closely related species could be ascribed to the fact that the chloroplast genome is uniparentally inherited [39]; and even after speciation, the chloroplast genome constitution of the newly evolved species might remain similar to the donor parent. Moreover, nucleotide substitution rate of chloroplast genome has been reported to be lower than the nuclear genome [40].

### Data set II: analyses based on 36 self collected/procured species along with the species for which sequences are available on GenBank

To determine the efficacy of the tested loci as DNA barcodes, the analyses were extended to a higher number of *Dendrobium *species (in addition to self investigated 36 species) for which the DNA sequences were available in GenBank. Different number of sequences, 93, 33, 20, 18 and 17 for ITS, *matK, rbcL, rpoB *and *rpoC1*, respectively, representing as many species, available on GenBank were downloaded (see Additional file 4).

#### Determination of inter-specific variation

Average inter-specific K2P distances calculated for ITS, *matK, rbcL, rpoB *and *rpoC1 *among 129, 69, 56, 54, and 53 species, were observed to be 0.2133 (0.011 - 0.4109), 0.0134 (0 - 0.0372), 0.0061 (0 - 0.023), 0.0067 (0 - 0.0296) and 0.0042 (0 - 0.0171), respectively (Table 4) [see Additional file 2(f)-(j)].

**Table 4:** Analysis of all species in Data Set II. Inter-specific K2P distances and per cent species resolution of different loci using downloaded sequences for *Dendrobium *species as well as sequences of 36 species generated during the study

Locus	No. of species analyzed	Average inter-specific K2P distance (Range)	% Species Resolution using K2P distances
**ITS**	129	0.2133 (0.011 - 0.4109)	100.00

***matK***	69	0.0134 (0 - 0.0372)	76.81

***rbcL***	56	0.0061 (0 - 0.023)	37.50

***rpoB***	54	0.0067 (0 - 0.0296)	48.15

***rpoC1***	53	0.0042 (0 - 0.0171)	39.62

In addition to 36 species analyzed presently, sequences of all the loci from chloroplast genome (*matK, rbcL, rpoB *and *rpoC1*) of only 16 additional species were available. Thus, analyses involving multi-locus combinations of the tested loci for 52 species were made. Average inter-specific distances among these 52 species were 0.0121 (0 - 0.0371), 0.0062 (0 - 0.023), 0.0069 (0 - 0.0296) and 0.0041 (0 - 0.0171) for *matK, rbcL, rpoB *and *rpoC1*, respectively (Table 5) [see Additional file 2(k)-(n)].

**Table 5:** Analysis of 52 species from Data Set II. Inter-specific K2P distances and per cent species resolution of different loci from chloroplast genome using downloaded as well as self generated sequences for 52 *Dendrobium *species for which sequences of all the four loci (*matK, rbcL, rpoB *and *rpoC1*) were present

Locus	No. of species analyzed	Average inter-specific K2P distance (Range)	No. of species successfully discriminated	% Species Resolution using K2P distances
***matK***	52	0.0121 (0 - 0.0371)	40	76.92

***rbcL***	52	0.0062 (0 - 0.023)	20	38.46

***rpoB***	52	0.0069 (0 - 0.0296)	27	51.92

***rpoC1***	52	0.0041 (0 - 0.0171)	22	42.31

#### Determination of species resolution

Out of 129 species analyzed for ITS, two species pairs exhibited distance estimates lower than the maximum intra-specific variation recorded. Therefore, these species could not be discriminated on the basis of ITS sequences. However, on literature survey, it was realized that both these species pairs, *D. macrostachyum *Lindl./*D. stuartii *F.M. Bailey [41]; and *D. goldschmidtianum/D. miyakei *(http://orchid.unibas.ch/site.synonyms.php) in fact represent the same species as the names are synonyms. This provided an example of congruence of conventional taxonomy and DNA barcoding.

Of the 69 species analyzed for *matK*, 53 could be successfully discriminated on the basis of K2P distances. Therefore, the species resolution was 76.81%. Likewise, the species resolution was observed to be 37.5% when *rbcL *sequences of 56 species were analyzed. Other two loci from chloroplast genome - *rpoB *and *rpoC1 *represented by 54 and 53 species, respectively, resolved 48.15% and 39.62% species, respectively (Table 4).

**Figure 2:**
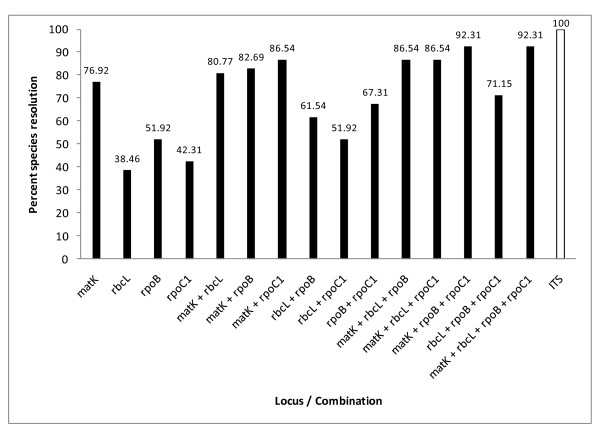
**Analysis of 52 species using single and multi-locus combinations**. Percent species resolution based on single as well as multi-locus combinations of the four chloroplast loci for 52 species of *Dendrobium*. Species resolution value for ITS is for 129 species.

Species resolution values calculated separately for 52 species for which sequences of all the chloroplast genome loci (*matK, rbcL, rpoB *and *rpoC1*) were available, were 76.92%, 38.46%, 51.92% and 42.31% for *matK, rbcL, rpoB *and *rpoC1*, respectively (Table 5). Two-locus combinations of *matK*+*rbcL, matK*+*rpoB, matK*+*rpoC1, rbcL*+*rpoB, rbcL*+*rpoC1 *and *rpoB*+*rpoC1 *exhibited species resolution of 80.77%, 82.69%, 86.54%, 61.54%, 51.92% and 67.31%, respectively. Among three-locus combinations, *matK*+*rpoB*+*rpoC1 *could discriminate 92.31% (48 out of 52) species, the rest viz., *matK*+*rbcL*+*rpoB, matK*+*rbcL*+*rpoC1 *and *rbcL*+*rpoB*+*rpoC1 *resolved 86.54%, 86.54% and 71.15% species, respectively. Even four-locus combination (*matK*+*rbcL*+*rpoB*+*rpoC1*) could not resolve beyond 92.31% (48 out of 52) species (Figure 2).

The present study based on congeneric species of *Dendrobium *revealed ITS to be the best DNA barcode affording 100% species resolution, thus apparently pointing towards its suitability as one of the candidate DNA barcodes for land plants. Earlier, some of the other groups had also recommended the use of ITS as DNA barcode for plants because of the presence of its multiple copies in the cells, easy retrieval of amplicons, high quality bidirectional sequences and a high resolution at species level [20,24]. However, the CBOL Plant Working Group [27] did not recognize ITS as a suitable locus for DNA barcoding due to the presence of intra-genomic variability, divergent paralogous copies within individuals [42] and pseudogenes [43], which could lead to difficulties in obtaining good quality sequences by direct sequencing of PCR products. Though its use as a supplementary barcode was recommended for those taxa in which loci from the chloroplast genome fail to resolve species and the direct sequencing of the PCR product is possible. There are several other limitations which restrict the use of ITS as a core barcode. For example, where the plants possess endophytic fungi there is a possibility of amplification of fungal ITS along with plant ITS [42]. Gonzalez et al. [44], in their study on 285 samples of Amazonian trees, reported that amplification and sequencing success rate for ITS was only 41%. Likewise, despite having highest sequence variation among the tested loci ITS could discriminate only 50% of the Indian Paphiopedilums as opposed to *matK *which provided 100% species resolution [6]. These reports pose a question on the universality of ITS.

An alternative to such problem could be the use of any one of the spacers, especially the second internal transcribed spacer (ITS2) as a barcode [11,28]. This small portion of ITS has been used in several studies and has proved to be useful in species discrimination [45-47]. The problems associated with amplification and sequencing of the entire ITS (ITS1-5.8S rRNA-ITS2) region were also reduced by selecting only ITS2 [11]. When tested for its ability to identify medicinal plants and their close relatives, ITS2 exhibited a discrimination ability of 92.7% at species level in more than 6600 plant samples, belonging to 4800 species from 753 genera [11]. Yao et al. [48] downloaded 50, 790 and 12, 221 ITS2 sequences belonging to plants and animals, respectively, from the GenBank and reported that this locus could successfully discriminate 76.1% dicotyledons, 74.2% monocotyledons, 67.1% gymnosperms, 88.1% ferns, 77.4% mosses and 91.7% animals at the species level. Since length of ITS2 is more conserved across plants than ITS1, it becomes easier to recognize the amplicon and sequence it in both directions [11]. However, there is a trade-off between high universality and the number of informative characters available for identification. Thus, ITS2 alone may not be suitable because of small sequence length (approx. 300 bp) which may not possess adequate amount of molecular information to discriminate congeneric species. This is best exemplified by the investigation on the members of the family Euphorbiaceae [47]. Using ITS2, species discrimination rate within the family was 91% but was only 68% among congeneric species of one genus - *Glochidion *[47].

Lahaye et al. [5] studied more than 1036 species of Mesoamerican orchids for checking the suitability of *matK *for cataloguing the plant biodiversity. They reported that *matK *alone or in combination with *trnH-psbA *could correctly identify > 90% of the investigated species. In the present study too, among the chloroplast loci studied, *matK *provided maximum species resolution of 80.56% when compared individually. However, the two-locus combination of *matK*+*rbcL *suggested by CBOL Plant Working Group for the land plants resolved 80.77% species as opposed to 86.54% provided by the combination of *matK*+*rpoC1 *in the analysis based on 52 species of *Dendrobium*. Based on only the chloroplast loci, the species resolution of 92.31% was provided by a combination of *matK*+*rpoB*+*rpoC1*, one of the three locus combinations suggested by Chase et al. [33] as DNA barcode. The trends were essentially similar when the data of 36 species were analyzed. These conclusions indicate the futility of including *rbcL *in the DNA barcode of at least *Dendrobium *species. The need for taxa specific barcode was also amply demonstrated by the study of Seberg and Petersen [26] who tested six plastid regions in different combinations for discriminating 86 species of *Crocus *and obtained maximum species resolution of 92% with a four locus combination of *ndhF*+*matK*+*trnH*-*psbA*+*rps8*-*rpl36*.

The *trnH-psbA *intergenic spacer has been reported as an effective barcode for *Dendrobium *species [28]. However, in our experience this posed problem in sequencing, which could be due to the occurrence of mono-nucleotide repeats or poly(A) structure within its sequence [32,49]. Even other workers have commented on the un-suitability of this locus as a barcode as its length varies from 300 - 1000 bp which could pose problem in sequence alignment [33]. Furthermore, it has also been reported that in orchids and amaryllids there is an insertion of *rps19 *and *rpl22 *genes within this spacer [5,22,37], hence causing difficulties in identification of the correct band among the amplicons, in case multiple bands are obtained. Recently, this spacer has also been found to contain intra-specific inversions in some species of Gentianaceae, which might lead to overestimation of sequence divergence among conspecific individuals [50].

Mostly the efficacy of different loci in discriminating plant species has been investigated among species occurring in a restricted geographic region or a floristic assemblage [11,23,25,27,44,51-55]. Even the most recently recommended barcode for the land plants comprising *matK+rbcL *by CBOL Plant Working Group was on the basis of comparison of the efficacy of seven loci among 397 plants belonging to taxonomically diverse groups [27]. In such a situation when phylogenetic distances are more among the species being resolved, the resolving power of any locus or a combination of loci would tend to be higher. Despite this, species resolution was only 72% [27]. This implies that 28 of the 100 identifications, using the suggested barcode, could be wrong. Similarly, studies dealing with limited number of species of a genus could result into premature conclusions. One such study based on only five species of *Dendrobium *concluded that the suggested two locus barcode of *matK*+*rbcL *was able to discriminate all species [56]. To highlight the artifacts of such studies carried out with limited number of species, out of the 52 species, we selected 10 species that were completely resolved by each locus from chloroplast genome individually (Table 6). When these species were analyzed for their inter-specific distances, all loci except *rpoC1 *showed more than 0.01 average inter-specific distance (Table 7) [see Additional file 2(o) - (r)]. This indicates that had we included only these 10 species in our analysis, the conclusion would have been that each of the loci is individually capable of providing 100% species resolution. Following the same argument, the conclusions of the present study may also change if more or all species of *Dendrobium *are included in the study.

**Table 6:** List of ten *Dendrobium *species that are completely resolved (100%) by each of the four tested chloroplast loci

**S. No**.	Species Name
1.	*Dendrobium capillipes*

2.	*Dendrobium hancockii*

3.	*Dendrobium harveyanum*

4.	*Dendrobium moniliforme*

5.	*Dendrobium trigonopus*

6.	*Dendrobium crumenatum*

7.	*Dendrobium haemoglossum*

8.	*Dendrobium herbaceum*

9.	*Dendrobium microbulbon*

10.	*Dendrobium peguanum*

**Table 7:** Analysis of selected ten species. Average inter-specific K2P distances for four loci from chloroplast genome involving 10 species that were successfully resolved by each locus

**S. No**.	Locus	Average inter-specific divergence (range)
1.	*matK*	0.02 (0.0041 - 0.0391)

2.	*rbcL*	0.0111 (0.0050 - 0.0185)

3.	*rpoB*	0.0104 (0.0058 - 0.0203)

4.	*rpoC1*	0.0097 (0.0048 - 0.017)

From the above, it becomes apparent that a universal barcode for plants, whether based on single locus or multiple loci, is still comparable to the "holy grail". In such a situation, the suggested use of the whole chloroplast sequence as a single locus barcode [35,57] might become a distinct possibility in near future; especially with advancements and significant cost reduction in sequencing technology. Moreover, this approach would not be dependent on the availability of universal primers as PCR amplification is not required and due to the availability of increased matrix length and number of informative sites the resolution would be tremendously increased. This has been well demonstrated and highlighted by an investigation on 32 gymnosperms, where resolving powers of the suggested two-locus barcode (*rbcL-matK*) and whole chloroplast genome were compared [57]. The present limitations to use of chloroplast sequences generated through MPS of total DNA for DNA barcoding are (i) inability of recovering indels necessary for distinguishing recently diverged species, (ii) availability of limited number of chloroplast genome sequences as reference sequences for assembly of short sequences generated by this method, and (iii) still to be demonstrated applicability of this approach for taxa having large genomes [35].

## Conclusions

In conclusion, one can say that a universal barcode for plants is as illusive as it was in 2005 when the first substantive study on DNA barcoding of plants appeared [20]. Rather, it needs to be accepted that DNA barcodes would be taxa specific. Thus, these are not likely to have as wider applicability; especially the capability of identifying the source of a totally unknown plant tissue, as has been continually envisaged and projected. However, if the use of whole chloroplast genome as single locus barcode becomes a reality the projected wider applicability of DNA barcoding might be restored.

## Methods

### Plants

The plants under investigation (292 accessions belonging to 36 species of *Dendrobium*) were collected/procured from different geographical locations of India viz., Pachmarhi (Madhya Pradesh), Nainital, Dehradun, Mussoorie and adjoining areas (Uttarakhand), Kolhapur and adjoining areas (Maharashtra), Kalimpong (West Bengal), Tropical Botanic Garden and Research Institute (TBGRI), Thiruvananthapuram (Kerala), Dibrugarh University (Assam), Bio-Resource Development Center (BRDC), Shillong (Meghalaya) and Botanical Survey of India (BSI), Shillong (Meghalaya) (see Additional file 5). During collection, it was ensured that no vegetative link existed between the two different accessions of the same species.

### Loci and primers

Five loci (*matK, rbcL, rpoB, rpoC1 *and *trnH-psbA *spacer) from the chloroplast genome and one locus (ITS) from nuclear genome of 292 individuals, belonging to 36 species of *Dendrobium *were tested for their ability to resolve congeneric species and to infer their applicability and efficacy as DNA barcodes. Primers for the amplification of *matK, rbcL, rpoB *and *rpoC1 *were taken from the Kew website (http://www.kew.org/barcoding/protocols.html) and were aligned with the chloroplast genome of *Phalaenopsis aphrodite *subsp. *formosana *[GenBank: NC_007499.1] [37]. The corresponding sequences were then taken as the primers for amplification of the respective loci. These primer sequences have also been used by us for the DNA barcoding of *Paphiopedilum*, another orchid [6]. The primers used for *trnH-psbA *spacer were those that were originally used by Tate and Simpson [58] and subsequently by Kress et al. [20]. ITS was amplified using the primers IT1 and IT2 [59], which have been reported to amplify ITS in both plants [6,59], (http://tdares.coa.gov.tw/htmlarea_file/web_articles/tdais/617/64-2.pdf) and animals (http://scialert.net/fulltext/?doi=jbs.2009.51.56).

### DNA isolation and amplification

Total genomic DNA of each accession was extracted, using (1) CTAB method [60], (2) genomic DNA purification kit (Fermentas #K0512), or (3) a modified CTAB protocol [61]. The last method was used for species with high mucilage content in their leaves and for those accessions in which pseudobulbs were the source of genomic DNA. PCR reaction mixture (20 μl) consisted of 1 unit of *Pfu *DNA polymerase (Fermentas #EP0502), 2 μl 10× PCR buffer with MgSO_4_, 2 μl of 2 mM dNTPs, 2 μl of each primer (10 μM) and 20 - 30 ng of template DNA. The thermal cycle for amplification of ITS was the same as followed by Tsai et al. [59]. For the loci from the chloroplast genome, thermal cycle consisted of an initial incubation for 5 min at 94°C, followed by 35 cycles of 30 sec at 94°C, 40 sec at 50°C, 1 min at 72°C, with a final extension of 7 min at 72°C [6]. PCR products were electrophoresed in 1% TAE (Tris-acetate-EDTA) agarose gels containing 0.5 μg/mL ethidium bromide (EtBr) and visualized on a UV trans-illuminator.

### Sequencing and analysis

The samples for which a single band of amplicon was obtained, 2 μl mixture of Exonuclease I (Exo I, Fermentas #EN0582) and Shrimp alkaline phosphatase (SAP, Fermentas #EF0511) containing 10 U Exo I and 1 U SAP was used to clean up 8 μl of PCR product. For the samples that produced multiple bands after amplification, the correct band was purified using GeneJET Gel Extraction Kit (Fermentas #K0692). The final product was subjected to forward and reverse sequencing using BigDye terminator v3.1 cycle sequencing kit on ABI Prism 3700 sequencer (Applied Biosystems, USA). The sequencing reaction mixture (10 μl) contained 0.5 μl of BigDye v3.1 ready reaction mixture, 3 μl of PCR product, 2 μl of 5× sequencing buffer, 1 μl of 10 μM primer, 3.5 μl of autoclaved MQ. For cycle sequencing 30 cycles of 10 sec at 96°C, 5 sec at 50°C, and 4 min at 60°C were carried out. Chromatograms were base-called using PHRED; thereafter, forward and reverse sequences were trimmed and assembled using Sequencher (Gene Codes Corporation, Ann Arbor, Michigan, USA). Each sequencher project file consisted of all the sequences of a single species of *Dendrobium *and its consensus sequence was taken as the representative sequence for that particular species. The identity of each sequence of all the five loci was checked by conducting BLAST analysis on NCBI. All 1180 sequences generated were submitted to the GenBank and their accession numbers [GenBank: HM054534 - HM055361 and GenBank: JF713083 - JF713434] were obtained (see Additional file 5). The intra- and inter-specific K2P distances were determined using MEGA 4.0. The representative sequence for each species was used for determining the inter-specific K2P distances. Multi-locus combinations of the chloroplast genome loci were also tested for their ability to discriminate among the investigated species. To check the performance of various loci, the analyses were extended to the DNA sequences of *Dendrobium *species already present in GenBank. Different number of sequences - 93, 33, 20, 18 and 17 for ITS, *matK, rbcL, rpoB *and *rpoC1*, respectively (see Additional file 4),-representing as many species in addition to the 36 species investigated under the present study, were downloaded from the GenBank. The species resolution was calculated by preparing a K2P distance matrix of all the species from the aligned DNA sequences of a particular locus using MEGA 4.0 [38]. Two species were considered as distinct, if their inter-specific K2P distance was more than the maximum intra-specific distance. Thus, species resolution of each locus was calculated according to the following formula:(A - B) × 100/A, where A = total no. of species and B = no. of species with K2P distance less than or equal to the intra-specific distance.

## Abbreviations

CBOL: Consortium for Barcode of Life; CO1: cytochrome *c *oxidase 1; CTAB: Cetyl trimethyl ammonium bromide; dNTP: Deoxy Nucleotide triphosphate; ITS: Nuclear Ribosomal Internal Transcribed Spacer; K2P: Kimura-2-Parameter; *matK*: maturase K; MEGA: Molecular Evolutionary Genetic Analysis; MQ: Milli-Q; NCBI: National Centre for Biotechnology Information; PCR: Polymerase Chain Reaction; *Pfu*: *Pyrococcus furiosus*; *rbcL*: Rubisco Large sub-unit; *rpoB*: RNA polymerase β sub-unit; *rpoC1*: RNA polymerase β' sub-unit; TAE: Tris-acetate-EDTA; *trnH*-*psbA*: Transfer RNA for histidine - D1 protein of photosystem II; UV: Ultraviolet

## Competing interests

The authors declare that they have no competing interests.

## Authors' contributions

SBB formulated the research problem. SBB, HKS and IP were involved in planning of the experiments. The experiments were executed exclusively by HKS and IP. The results were analyzed by HKS, IP, SR and SBB. All authors read and approved the final manuscript.

## Supplementary Material

Additional file 1 **First page screen shots of BLAST results of ITS sequences belonging to 129 species of *Dendrobium***. Click here for file

Additional file 2 **Pairwise K2P distance matrices**. Click here for file

Additional file 3 **Enlarged view of flowers of *D. aphyllum *(A) and *D. macrostachyum *(B)**. Click here for file

Additional file 4 **Accession numbers of sequences of five loci downloaded from the GenBank**. Click here for file

Additional file 5 **Voucher numbers, herbarium accession numbers, location/source and Genbank accession number for sequences of all the loci amplified and sequenced from individuals of *Dendrobium *species collected/procured from different geographical locations in India**. Click here for file
