# Supplementary material for: The loci recommended as universal barcodes for plants on the basis of floristic studies may not work with congeneric species as exemplified by DNA barcoding of Dendrobium species
Source: BMC Res Notes. 2012 Jan 19;5:42. doi: 10.1186/1756-0500-5-42 (PMC3292824; doi:10.1186/1756-0500-5-42)
Supplement: Additional file 1 — First page screen shots of BLAST results of ITS sequences belonging to 129 species of Dendrobium. [file 1756-0500-5-42-S1.PDF]

Additional file 1: First page screen shots of BLAST results of ITS sequences belonging to 129 species of *Dendrobium*.

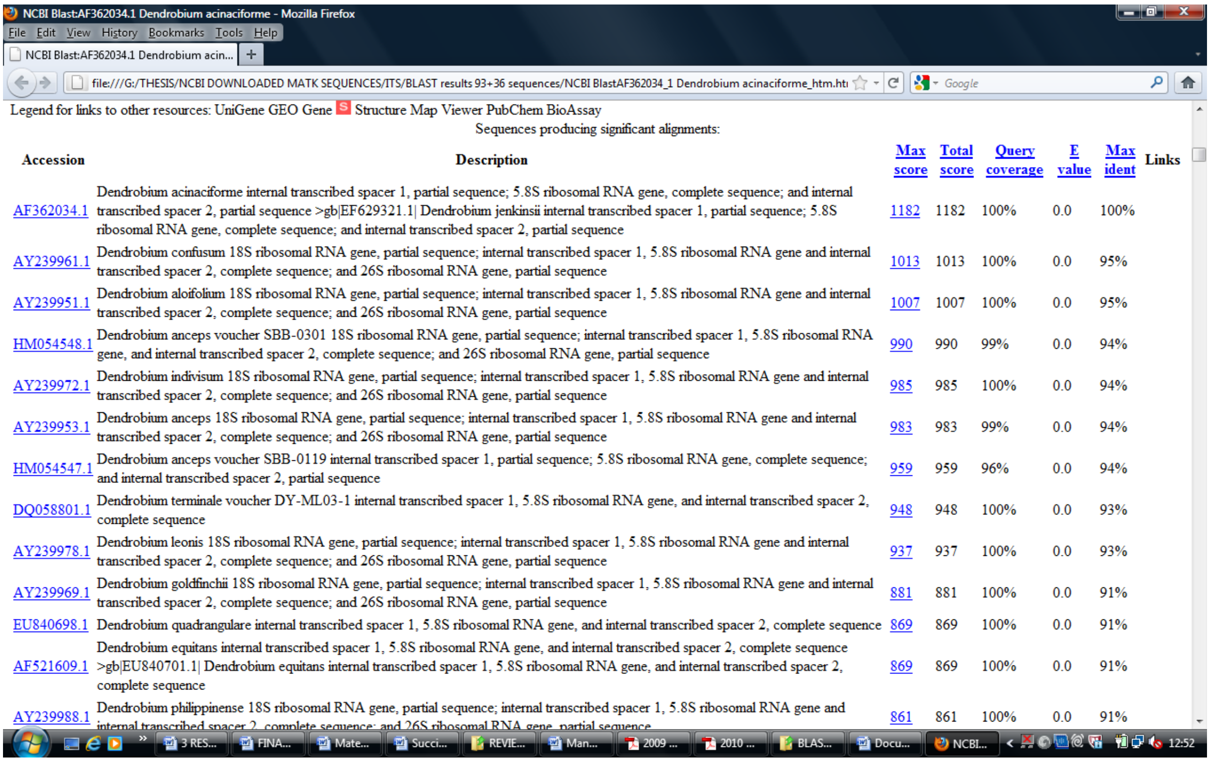

NCBI Blast:AF362034.1 Dendrobium acinaciforme - Mozilla Firefox

file:///G:/THESIS/NCBI DOWNLOADED MATK SEQUENCES/ITS/BLAST results 93+36 sequences/NCBI BlastAF362034_1 Dendrobium acinaciforme_htm.htm

Legend for links to other resources: UniGene GEO Gene Structure Map Viewer PubChem BioAssay

Sequences producing significant alignments:

Accession	Description	Max score	Total score	Query coverage	E value	Max ident	Links
AF362034.1	Dendrobium acinaciforme internal transcribed spacer 1, partial sequence; 5.8S ribosomal RNA gene, complete sequence; and internal transcribed spacer 2, partial sequence >gb|EF629321.1| Dendrobium jenkinsii internal transcribed spacer 1, partial sequence; 5.8S ribosomal RNA gene, complete sequence; and internal transcribed spacer 2, partial sequence	1182	1182	100%	0.0	100%	
AY239961.1	Dendrobium confusum 18S ribosomal RNA gene, partial sequence; internal transcribed spacer 1, 5.8S ribosomal RNA gene and internal transcribed spacer 2, complete sequence; and 26S ribosomal RNA gene, partial sequence	1013	1013	100%	0.0	95%	
AY239951.1	Dendrobium aloifolium 18S ribosomal RNA gene, partial sequence; internal transcribed spacer 1, 5.8S ribosomal RNA gene and internal transcribed spacer 2, complete sequence; and 26S ribosomal RNA gene, partial sequence	1007	1007	100%	0.0	95%	
HM054548.1	Dendrobium anceps voucher SBB-0301 18S ribosomal RNA gene, partial sequence; internal transcribed spacer 1, 5.8S ribosomal RNA gene, and internal transcribed spacer 2, complete sequence; and 26S ribosomal RNA gene, partial sequence	990	990	99%	0.0	94%	
AY239972.1	Dendrobium indivisum 18S ribosomal RNA gene, partial sequence; internal transcribed spacer 1, 5.8S ribosomal RNA gene and internal transcribed spacer 2, complete sequence; and 26S ribosomal RNA gene, partial sequence	985	985	100%	0.0	94%	
AY239953.1	Dendrobium anceps 18S ribosomal RNA gene, partial sequence; internal transcribed spacer 1, 5.8S ribosomal RNA gene and internal transcribed spacer 2, complete sequence; and 26S ribosomal RNA gene, partial sequence	983	983	99%	0.0	94%	
HM054547.1	Dendrobium anceps voucher SBB-0119 internal transcribed spacer 1, partial sequence; 5.8S ribosomal RNA gene, complete sequence; and internal transcribed spacer 2, partial sequence	959	959	96%	0.0	94%	
DQ058801.1	Dendrobium terminale voucher DY-ML03-1 internal transcribed spacer 1, 5.8S ribosomal RNA gene, and internal transcribed spacer 2, complete sequence	948	948	100%	0.0	93%	
AY239978.1	Dendrobium leonis 18S ribosomal RNA gene, partial sequence; internal transcribed spacer 1, 5.8S ribosomal RNA gene and internal transcribed spacer 2, complete sequence; and 26S ribosomal RNA gene, partial sequence	937	937	100%	0.0	93%	
AY239969.1	Dendrobium goldfinchii 18S ribosomal RNA gene, partial sequence; internal transcribed spacer 1, 5.8S ribosomal RNA gene and internal transcribed spacer 2, complete sequence; and 26S ribosomal RNA gene, partial sequence	881	881	100%	0.0	91%	
EU840698.1	Dendrobium quadrangulare internal transcribed spacer 1, 5.8S ribosomal RNA gene, and internal transcribed spacer 2, complete sequence	869	869	100%	0.0	91%	
AF521609.1	Dendrobium equitans internal transcribed spacer 1, 5.8S ribosomal RNA gene, and internal transcribed spacer 2, complete sequence >gb|EU840701.1| Dendrobium equitans internal transcribed spacer 1, 5.8S ribosomal RNA gene, and internal transcribed spacer 2, complete sequence	869	869	100%	0.0	91%	
AY239988.1	Dendrobium philippinense 18S ribosomal RNA gene, partial sequence; internal transcribed spacer 1, 5.8S ribosomal RNA gene and internal transcribed spacer 2, complete sequence; and 26S ribosomal RNA gene, partial sequence	861	861	100%	0.0	91%	

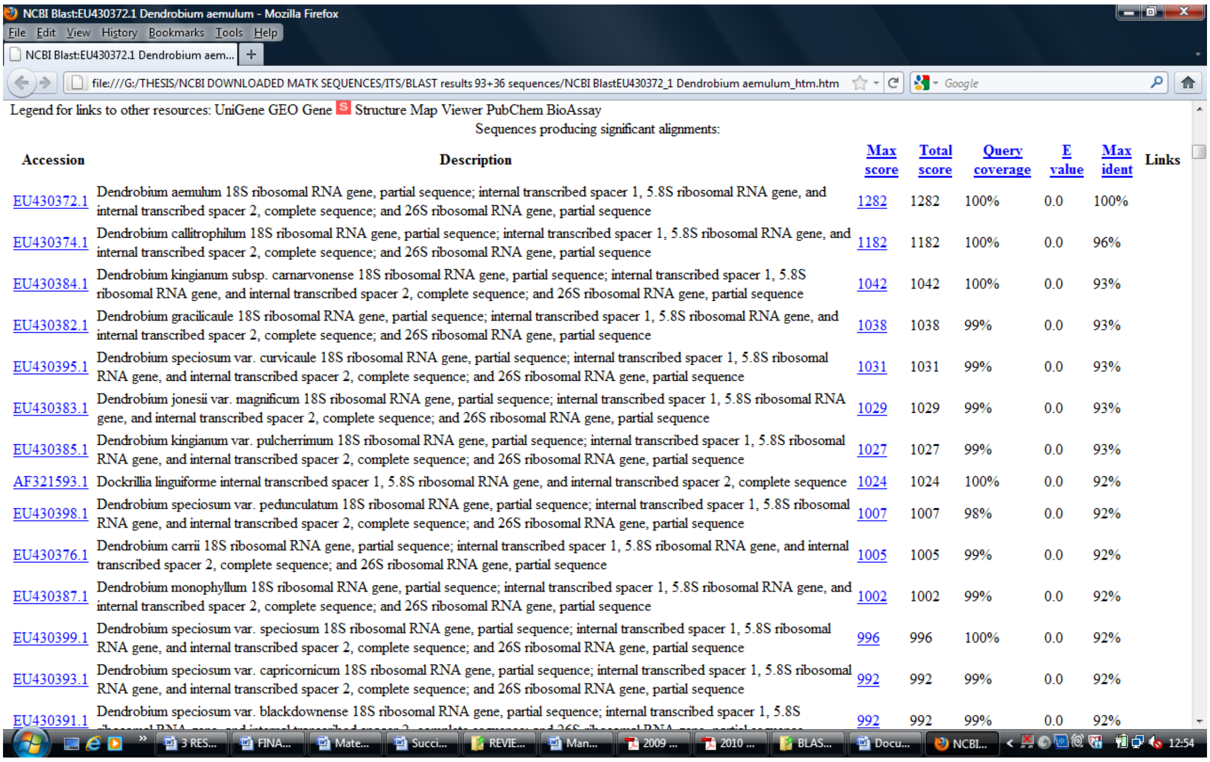

NCBI Blast:EU430372.1 Dendrobium aemulum - Mozilla Firefox

file:///G:/THESIS/NCBI DOWNLOADED MATK SEQUENCES/ITS/BLAST results 93+36 sequences/NCBI BlastEU430372_1 Dendrobium aemulum_htm.htm

Legend for links to other resources: UniGene GEO Gene Structure Map Viewer PubChem BioAssay

Sequences producing significant alignments:

Accession	Description	Max score	Total score	Query coverage	E value	Max ident	Links
EU430372.1	Dendrobium aemulum 18S ribosomal RNA gene, partial sequence; internal transcribed spacer 1, 5.8S ribosomal RNA gene, and internal transcribed spacer 2, complete sequence; and 26S ribosomal RNA gene, partial sequence	1282	1282	100%	0.0	100%	
EU430374.1	Dendrobium callitrophilum 18S ribosomal RNA gene, partial sequence; internal transcribed spacer 1, 5.8S ribosomal RNA gene, and internal transcribed spacer 2, complete sequence; and 26S ribosomal RNA gene, partial sequence	1182	1182	100%	0.0	96%	
EU430384.1	Dendrobium kingianum subsp. carnarvonense 18S ribosomal RNA gene, partial sequence; internal transcribed spacer 1, 5.8S ribosomal RNA gene, and internal transcribed spacer 2, complete sequence; and 26S ribosomal RNA gene, partial sequence	1042	1042	100%	0.0	93%	
EU430382.1	Dendrobium gracilicaule 18S ribosomal RNA gene, partial sequence; internal transcribed spacer 1, 5.8S ribosomal RNA gene, and internal transcribed spacer 2, complete sequence; and 26S ribosomal RNA gene, partial sequence	1038	1038	99%	0.0	93%	
EU430395.1	Dendrobium speciosum var. curvicaule 18S ribosomal RNA gene, partial sequence; internal transcribed spacer 1, 5.8S ribosomal RNA gene, and internal transcribed spacer 2, complete sequence; and 26S ribosomal RNA gene, partial sequence	1031	1031	99%	0.0	93%	
EU430383.1	Dendrobium jonesii var. magnificum 18S ribosomal RNA gene, partial sequence; internal transcribed spacer 1, 5.8S ribosomal RNA gene, and internal transcribed spacer 2, complete sequence; and 26S ribosomal RNA gene, partial sequence	1029	1029	99%	0.0	93%	
EU430385.1	Dendrobium kingianum var. pulcherrimum 18S ribosomal RNA gene, partial sequence; internal transcribed spacer 1, 5.8S ribosomal RNA gene, and internal transcribed spacer 2, complete sequence; and 26S ribosomal RNA gene, partial sequence	1027	1027	99%	0.0	93%	
AF321593.1	Dockrillia linguiforme internal transcribed spacer 1, 5.8S ribosomal RNA gene, and internal transcribed spacer 2, complete sequence	1024	1024	100%	0.0	92%	
EU430398.1	Dendrobium speciosum var. pedunculatum 18S ribosomal RNA gene, partial sequence; internal transcribed spacer 1, 5.8S ribosomal RNA gene, and internal transcribed spacer 2, complete sequence; and 26S ribosomal RNA gene, partial sequence	1007	1007	98%	0.0	92%	
EU430376.1	Dendrobium carrii 18S ribosomal RNA gene, partial sequence; internal transcribed spacer 1, 5.8S ribosomal RNA gene, and internal transcribed spacer 2, complete sequence; and 26S ribosomal RNA gene, partial sequence	1005	1005	99%	0.0	92%	
EU430387.1	Dendrobium monophyllum 18S ribosomal RNA gene, partial sequence; internal transcribed spacer 1, 5.8S ribosomal RNA gene, and internal transcribed spacer 2, complete sequence; and 26S ribosomal RNA gene, partial sequence	1002	1002	99%	0.0	92%	
EU430399.1	Dendrobium speciosum var. speciosum 18S ribosomal RNA gene, partial sequence; internal transcribed spacer 1, 5.8S ribosomal RNA gene, and internal transcribed spacer 2, complete sequence; and 26S ribosomal RNA gene, partial sequence	996	996	100%	0.0	92%	
EU430393.1	Dendrobium speciosum var. capricornicum 18S ribosomal RNA gene, partial sequence; internal transcribed spacer 1, 5.8S ribosomal RNA gene, and internal transcribed spacer 2, complete sequence; and 26S ribosomal RNA gene, partial sequence	992	992	99%	0.0	92%	
EU430391.1	Dendrobium speciosum var. blackdownense 18S ribosomal RNA gene, partial sequence; internal transcribed spacer 1, 5.8S [illegible]	992	992	99%	0.0	92%	

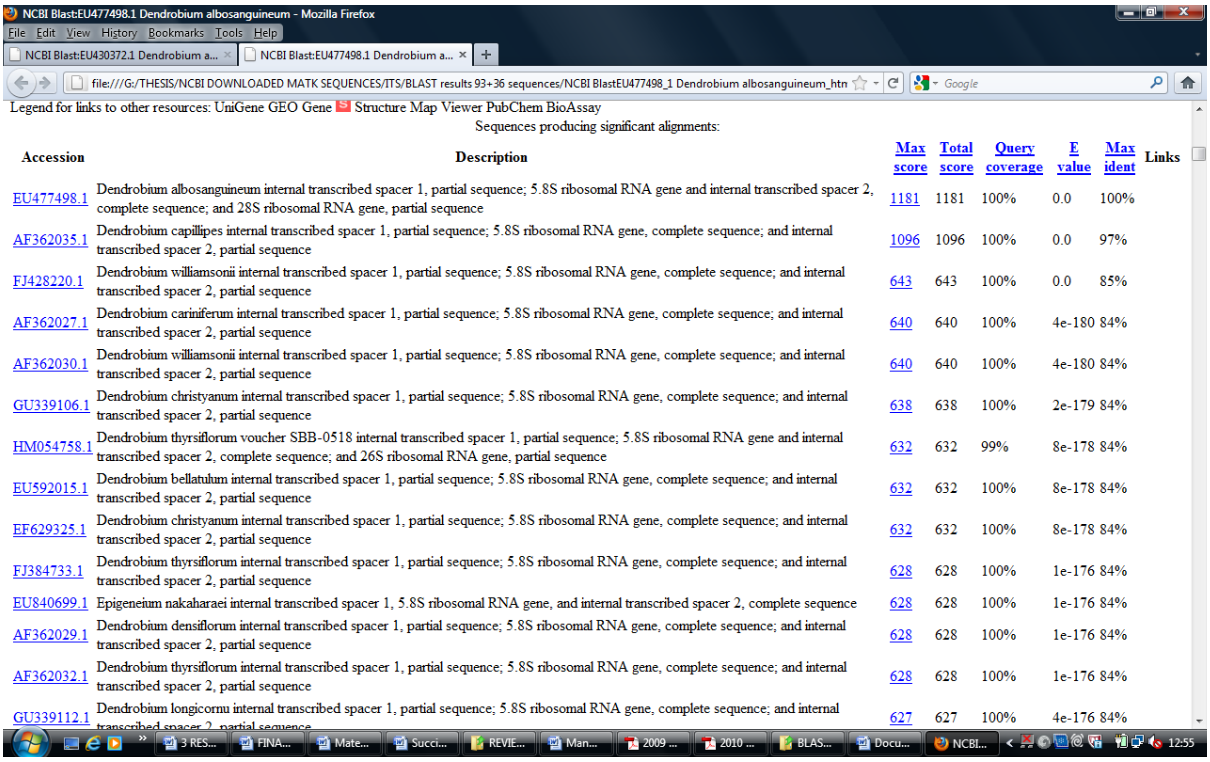

NCBI Blast:EU477498.1 Dendrobium albosanguineum - Mozilla Firefox

file:///G:/THESIS/NCBI DOWNLOADED MATK SEQUENCES/ITS/BLAST results 93+36 sequences/NCBI BlastEU477498_1 Dendrobium albosanguineum_htn

Legend for links to other resources: UniGene GEO Gene Structure Map Viewer PubChem BioAssay

Sequences producing significant alignments:

Accession	Description	Max score	Total score	Query coverage	E value	Max ident	Links
EU477498.1	Dendrobium albosanguineum internal transcribed spacer 1, partial sequence; 5.8S ribosomal RNA gene and internal transcribed spacer 2, complete sequence; and 28S ribosomal RNA gene, partial sequence	1181	1181	100%	0.0	100%	
AF362035.1	Dendrobium capillipes internal transcribed spacer 1, partial sequence; 5.8S ribosomal RNA gene, complete sequence; and internal transcribed spacer 2, partial sequence	1096	1096	100%	0.0	97%	
FJ428220.1	Dendrobium williamsonii internal transcribed spacer 1, partial sequence; 5.8S ribosomal RNA gene, complete sequence; and internal transcribed spacer 2, partial sequence	643	643	100%	0.0	85%	
AF362027.1	Dendrobium cariniferum internal transcribed spacer 1, partial sequence; 5.8S ribosomal RNA gene, complete sequence; and internal transcribed spacer 2, partial sequence	640	640	100%	4e-180	84%	
AF362030.1	Dendrobium williamsonii internal transcribed spacer 1, partial sequence; 5.8S ribosomal RNA gene, complete sequence; and internal transcribed spacer 2, partial sequence	640	640	100%	4e-180	84%	
GU339106.1	Dendrobium christyanum internal transcribed spacer 1, partial sequence; 5.8S ribosomal RNA gene, complete sequence; and internal transcribed spacer 2, partial sequence	638	638	100%	2e-179	84%	
HM054758.1	Dendrobium thyrsiflorum voucher SBB-0518 internal transcribed spacer 1, partial sequence; 5.8S ribosomal RNA gene and internal transcribed spacer 2, complete sequence; and 26S ribosomal RNA gene, partial sequence	632	632	99%	8e-178	84%	
EU592015.1	Dendrobium bellatulum internal transcribed spacer 1, partial sequence; 5.8S ribosomal RNA gene, complete sequence; and internal transcribed spacer 2, partial sequence	632	632	100%	8e-178	84%	
EF629325.1	Dendrobium christyanum internal transcribed spacer 1, partial sequence; 5.8S ribosomal RNA gene, complete sequence; and internal transcribed spacer 2, partial sequence	632	632	100%	8e-178	84%	
FJ384733.1	Dendrobium thyrsiflorum internal transcribed spacer 1, partial sequence; 5.8S ribosomal RNA gene, complete sequence; and internal transcribed spacer 2, partial sequence	628	628	100%	1e-176	84%	
EU840699.1	Epigeneium nakaharaei internal transcribed spacer 1, 5.8S ribosomal RNA gene, and internal transcribed spacer 2, complete sequence	628	628	100%	1e-176	84%	
AF362029.1	Dendrobium densiflorum internal transcribed spacer 1, partial sequence; 5.8S ribosomal RNA gene, complete sequence; and internal transcribed spacer 2, partial sequence	628	628	100%	1e-176	84%	
AF362032.1	Dendrobium thyrsiflorum internal transcribed spacer 1, partial sequence; 5.8S ribosomal RNA gene, complete sequence; and internal transcribed spacer 2, partial sequence	628	628	100%	1e-176	84%	
GU339112.1	Dendrobium longicornu internal transcribed spacer 1, partial sequence; 5.8S ribosomal RNA gene, complete sequence; and internal transcribed spacer 2, partial sequence	627	627	100%	4e-176	84%	

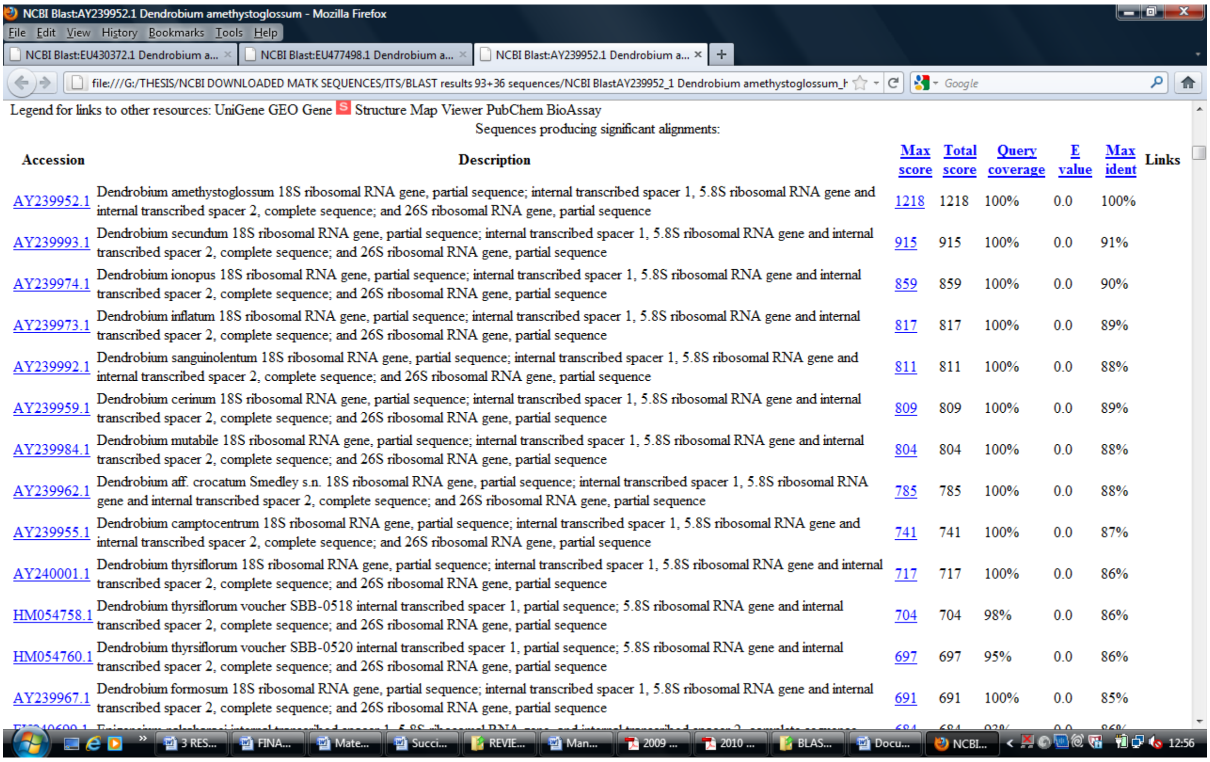

NCBI Blast:AY239952.1 Dendrobium amethystoglossum - Mozilla Firefox

file:///G:/THESIS/NCBI DOWNLOADED MATK SEQUENCES/ITS/BLAST results 93+36 sequences/NCBI BlastAY239952_1 Dendrobium amethystoglossum_h

Legend for links to other resources: UniGene GEO Gene Structure Map Viewer PubChem BioAssay

Sequences producing significant alignments:

Accession	Description	Max score	Total score	Query coverage	E value	Max ident	Links
AY239952.1	Dendrobium amethystoglossum 18S ribosomal RNA gene, partial sequence; internal transcribed spacer 1, 5.8S ribosomal RNA gene and internal transcribed spacer 2, complete sequence; and 26S ribosomal RNA gene, partial sequence	1218	1218	100%	0.0	100%	
AY239993.1	Dendrobium secundum 18S ribosomal RNA gene, partial sequence; internal transcribed spacer 1, 5.8S ribosomal RNA gene and internal transcribed spacer 2, complete sequence; and 26S ribosomal RNA gene, partial sequence	915	915	100%	0.0	91%	
AY239974.1	Dendrobium ionopus 18S ribosomal RNA gene, partial sequence; internal transcribed spacer 1, 5.8S ribosomal RNA gene and internal transcribed spacer 2, complete sequence; and 26S ribosomal RNA gene, partial sequence	859	859	100%	0.0	90%	
AY239973.1	Dendrobium inflatum 18S ribosomal RNA gene, partial sequence; internal transcribed spacer 1, 5.8S ribosomal RNA gene and internal transcribed spacer 2, complete sequence; and 26S ribosomal RNA gene, partial sequence	817	817	100%	0.0	89%	
AY239992.1	Dendrobium sanguinolentum 18S ribosomal RNA gene, partial sequence; internal transcribed spacer 1, 5.8S ribosomal RNA gene and internal transcribed spacer 2, complete sequence; and 26S ribosomal RNA gene, partial sequence	811	811	100%	0.0	88%	
AY239959.1	Dendrobium cerinum 18S ribosomal RNA gene, partial sequence; internal transcribed spacer 1, 5.8S ribosomal RNA gene and internal transcribed spacer 2, complete sequence; and 26S ribosomal RNA gene, partial sequence	809	809	100%	0.0	89%	
AY239984.1	Dendrobium mutabile 18S ribosomal RNA gene, partial sequence; internal transcribed spacer 1, 5.8S ribosomal RNA gene and internal transcribed spacer 2, complete sequence; and 26S ribosomal RNA gene, partial sequence	804	804	100%	0.0	88%	
AY239962.1	Dendrobium aff. crocatum Smedley s.n. 18S ribosomal RNA gene, partial sequence; internal transcribed spacer 1, 5.8S ribosomal RNA gene and internal transcribed spacer 2, complete sequence; and 26S ribosomal RNA gene, partial sequence	785	785	100%	0.0	88%	
AY239955.1	Dendrobium camptocentrum 18S ribosomal RNA gene, partial sequence; internal transcribed spacer 1, 5.8S ribosomal RNA gene and internal transcribed spacer 2, complete sequence; and 26S ribosomal RNA gene, partial sequence	741	741	100%	0.0	87%	
AY240001.1	Dendrobium thyrsiflorum 18S ribosomal RNA gene, partial sequence; internal transcribed spacer 1, 5.8S ribosomal RNA gene and internal transcribed spacer 2, complete sequence; and 26S ribosomal RNA gene, partial sequence	717	717	100%	0.0	86%	
HM054758.1	Dendrobium thyrsiflorum voucher SBB-0518 internal transcribed spacer 1, partial sequence; 5.8S ribosomal RNA gene and internal transcribed spacer 2, complete sequence; and 26S ribosomal RNA gene, partial sequence	704	704	98%	0.0	86%	
HM054760.1	Dendrobium thyrsiflorum voucher SBB-0520 internal transcribed spacer 1, partial sequence; 5.8S ribosomal RNA gene and internal transcribed spacer 2, complete sequence; and 26S ribosomal RNA gene, partial sequence	697	697	95%	0.0	86%	
AY239967.1	Dendrobium formosum 18S ribosomal RNA gene, partial sequence; internal transcribed spacer 1, 5.8S ribosomal RNA gene and internal transcribed spacer 2, complete sequence; and 26S ribosomal RNA gene, partial sequence	691	691	100%	0.0	85%	
[illegible]	[illegible]	[illegible]	[illegible]	[illegible]	[illegible]	[illegible]	

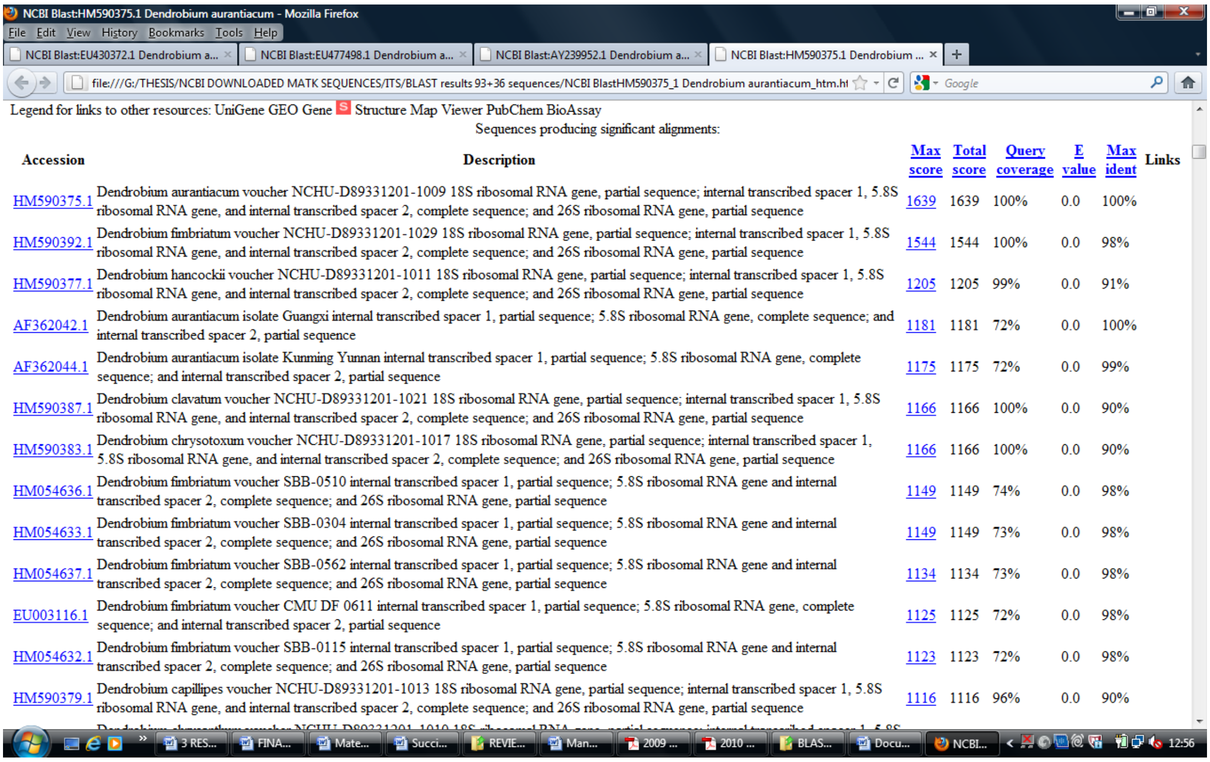

NCBI Blast:HM590375.1 Dendrobium aurantiacum - Mozilla Firefox

Legend for links to other resources: UniGene GEO Gene Structure Map Viewer PubChem BioAssay

Sequences producing significant alignments:

Accession	Description	Max score	Total score	Query coverage	E value	Max ident	Links
HM590375.1	Dendrobium aurantiacum voucher NCHU-D89331201-1009 18S ribosomal RNA gene, partial sequence; internal transcribed spacer 1, 5.8S ribosomal RNA gene, and internal transcribed spacer 2, complete sequence; and 26S ribosomal RNA gene, partial sequence	1639	1639	100%	0.0	100%	
HM590392.1	Dendrobium fimbriatum voucher NCHU-D89331201-1029 18S ribosomal RNA gene, partial sequence; internal transcribed spacer 1, 5.8S ribosomal RNA gene, and internal transcribed spacer 2, complete sequence; and 26S ribosomal RNA gene, partial sequence	1544	1544	100%	0.0	98%	
HM590377.1	Dendrobium hancockii voucher NCHU-D89331201-1011 18S ribosomal RNA gene, partial sequence; internal transcribed spacer 1, 5.8S ribosomal RNA gene, and internal transcribed spacer 2, complete sequence; and 26S ribosomal RNA gene, partial sequence	1205	1205	99%	0.0	91%	
AF362042.1	Dendrobium aurantiacum isolate Guangxi internal transcribed spacer 1, partial sequence; 5.8S ribosomal RNA gene, complete sequence; and internal transcribed spacer 2, partial sequence	1181	1181	72%	0.0	100%	
AF362044.1	Dendrobium aurantiacum isolate Kunming Yunnan internal transcribed spacer 1, partial sequence; 5.8S ribosomal RNA gene, complete sequence; and internal transcribed spacer 2, partial sequence	1175	1175	72%	0.0	99%	
HM590387.1	Dendrobium clavatum voucher NCHU-D89331201-1021 18S ribosomal RNA gene, partial sequence; internal transcribed spacer 1, 5.8S ribosomal RNA gene, and internal transcribed spacer 2, complete sequence; and 26S ribosomal RNA gene, partial sequence	1166	1166	100%	0.0	90%	
HM590383.1	Dendrobium chrysotoxum voucher NCHU-D89331201-1017 18S ribosomal RNA gene, partial sequence; internal transcribed spacer 1, 5.8S ribosomal RNA gene, and internal transcribed spacer 2, complete sequence; and 26S ribosomal RNA gene, partial sequence	1166	1166	100%	0.0	90%	
HM054636.1	Dendrobium fimbriatum voucher SBB-0510 internal transcribed spacer 1, partial sequence; 5.8S ribosomal RNA gene and internal transcribed spacer 2, complete sequence; and 26S ribosomal RNA gene, partial sequence	1149	1149	74%	0.0	98%	
HM054633.1	Dendrobium fimbriatum voucher SBB-0304 internal transcribed spacer 1, partial sequence; 5.8S ribosomal RNA gene and internal transcribed spacer 2, complete sequence; and 26S ribosomal RNA gene, partial sequence	1149	1149	73%	0.0	98%	
HM054637.1	Dendrobium fimbriatum voucher SBB-0562 internal transcribed spacer 1, partial sequence; 5.8S ribosomal RNA gene and internal transcribed spacer 2, complete sequence; and 26S ribosomal RNA gene, partial sequence	1134	1134	73%	0.0	98%	
EU003116.1	Dendrobium fimbriatum voucher CMU DF 0611 internal transcribed spacer 1, partial sequence; 5.8S ribosomal RNA gene, complete sequence; and internal transcribed spacer 2, partial sequence	1125	1125	72%	0.0	98%	
HM054632.1	Dendrobium fimbriatum voucher SBB-0115 internal transcribed spacer 1, partial sequence; 5.8S ribosomal RNA gene and internal transcribed spacer 2, complete sequence; and 26S ribosomal RNA gene, partial sequence	1123	1123	72%	0.0	98%	
HM590379.1	Dendrobium capillipes voucher NCHU-D89331201-1013 18S ribosomal RNA gene, partial sequence; internal transcribed spacer 1, 5.8S ribosomal RNA gene, and internal transcribed spacer 2, complete sequence; and 26S ribosomal RNA gene, partial sequence	1116	1116	96%	0.0	90%	
[illegible]	[illegible]	[illegible]	[illegible]	[illegible]	[illegible]	[illegible]	

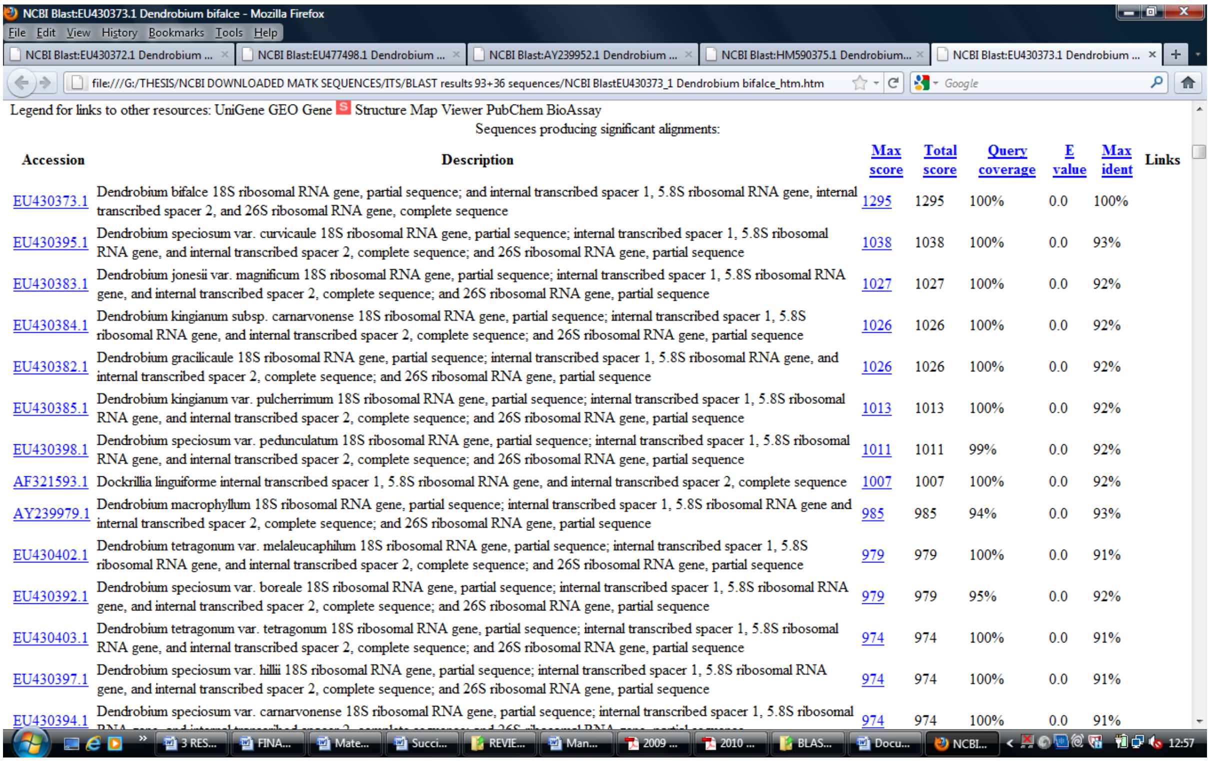

NCBI Blast:EU430373.1 Dendrobium bifalce - Mozilla Firefox

Legend for links to other resources: UniGene GEO Gene Structure Map Viewer PubChem BioAssay

Sequences producing significant alignments:

Accession	Description	Max score	Total score	Query coverage	E value	Max ident	Links
EU430373.1	Dendrobium bifalce 18S ribosomal RNA gene, partial sequence; and internal transcribed spacer 1, 5.8S ribosomal RNA gene, internal transcribed spacer 2, and 26S ribosomal RNA gene, complete sequence	1295	1295	100%	0.0	100%	
EU430395.1	Dendrobium speciosum var. curvicaule 18S ribosomal RNA gene, partial sequence; internal transcribed spacer 1, 5.8S ribosomal RNA gene, and internal transcribed spacer 2, complete sequence; and 26S ribosomal RNA gene, partial sequence	1038	1038	100%	0.0	93%	
EU430383.1	Dendrobium jonesii var. magnificum 18S ribosomal RNA gene, partial sequence; internal transcribed spacer 1, 5.8S ribosomal RNA gene, and internal transcribed spacer 2, complete sequence; and 26S ribosomal RNA gene, partial sequence	1027	1027	100%	0.0	92%	
EU430384.1	Dendrobium kingianum subsp. carnarvonense 18S ribosomal RNA gene, partial sequence; internal transcribed spacer 1, 5.8S ribosomal RNA gene, and internal transcribed spacer 2, complete sequence; and 26S ribosomal RNA gene, partial sequence	1026	1026	100%	0.0	92%	
EU430382.1	Dendrobium gracilicaule 18S ribosomal RNA gene, partial sequence; internal transcribed spacer 1, 5.8S ribosomal RNA gene, and internal transcribed spacer 2, complete sequence; and 26S ribosomal RNA gene, partial sequence	1026	1026	100%	0.0	92%	
EU430385.1	Dendrobium kingianum var. pulcherrimum 18S ribosomal RNA gene, partial sequence; internal transcribed spacer 1, 5.8S ribosomal RNA gene, and internal transcribed spacer 2, complete sequence; and 26S ribosomal RNA gene, partial sequence	1013	1013	100%	0.0	92%	
EU430398.1	Dendrobium speciosum var. pedunculatum 18S ribosomal RNA gene, partial sequence; internal transcribed spacer 1, 5.8S ribosomal RNA gene, and internal transcribed spacer 2, complete sequence; and 26S ribosomal RNA gene, partial sequence	1011	1011	99%	0.0	92%	
AF321593.1	Dockrillia linguiforme internal transcribed spacer 1, 5.8S ribosomal RNA gene, and internal transcribed spacer 2, complete sequence	1007	1007	100%	0.0	92%	
AY239979.1	Dendrobium macrophyllum 18S ribosomal RNA gene, partial sequence; internal transcribed spacer 1, 5.8S ribosomal RNA gene and internal transcribed spacer 2, complete sequence; and 26S ribosomal RNA gene, partial sequence	985	985	94%	0.0	93%	
EU430402.1	Dendrobium tetragonum var. melaleucaphilum 18S ribosomal RNA gene, partial sequence; internal transcribed spacer 1, 5.8S ribosomal RNA gene, and internal transcribed spacer 2, complete sequence; and 26S ribosomal RNA gene, partial sequence	979	979	100%	0.0	91%	
EU430392.1	Dendrobium speciosum var. boreale 18S ribosomal RNA gene, partial sequence; internal transcribed spacer 1, 5.8S ribosomal RNA gene, and internal transcribed spacer 2, complete sequence; and 26S ribosomal RNA gene, partial sequence	979	979	95%	0.0	92%	
EU430403.1	Dendrobium tetragonum var. tetragonum 18S ribosomal RNA gene, partial sequence; internal transcribed spacer 1, 5.8S ribosomal RNA gene, and internal transcribed spacer 2, complete sequence; and 26S ribosomal RNA gene, partial sequence	974	974	100%	0.0	91%	
EU430397.1	Dendrobium speciosum var. hillii 18S ribosomal RNA gene, partial sequence; internal transcribed spacer 1, 5.8S ribosomal RNA gene, and internal transcribed spacer 2, complete sequence; and 26S ribosomal RNA gene, partial sequence	974	974	100%	0.0	91%	
EU430394.1	Dendrobium speciosum var. carnarvonense 18S ribosomal RNA gene, partial sequence; internal transcribed spacer 1, 5.8S ribosomal [illegible]	974	974	100%	0.0	91%	

NCBI Blast:AY239954.1 Dendrobium bracteosum - Mozilla Firefox

file:///G:/THESIS/NCBI DOWNLOADED MATK SEQUENCES/ITS/BLAST results 93+36 sequences/NCBI BlastAY239954_1 Dendrobium bracteosum_htm.htm

Legend for links to other resources: UniGene GEO Gene Structure Map Viewer PubChem BioAssay

Sequences producing significant alignments:

Accession	Description	Max score	Total score	Query coverage	E value	Max ident	Links
AY239954.1	Dendrobium bracteosum 18S ribosomal RNA gene, partial sequence; internal transcribed spacer 1, 5.8S ribosomal RNA gene and internal transcribed spacer 2, complete sequence; and 26S ribosomal RNA gene, partial sequence	1216	1216	100%	0.0	100%	
AY239968.1	Dendrobium fulgidum 18S ribosomal RNA gene, partial sequence; internal transcribed spacer 1, 5.8S ribosomal RNA gene and internal transcribed spacer 2, complete sequence; and 26S ribosomal RNA gene, partial sequence	917	917	100%	0.0	91%	
AY239986.1	Dendrobium nothofagicola 18S ribosomal RNA gene, partial sequence; internal transcribed spacer 1, 5.8S ribosomal RNA gene and internal transcribed spacer 2, complete sequence; and 26S ribosomal RNA gene, partial sequence	885	885	100%	0.0	90%	
AY239997.1	Dendrobium sophronites 18S ribosomal RNA gene, partial sequence; internal transcribed spacer 1, 5.8S ribosomal RNA gene and internal transcribed spacer 2, complete sequence; and 26S ribosomal RNA gene, partial sequence	876	876	100%	0.0	90%	
AY239950.1	Dendrobium agathodaemonis 18S ribosomal RNA gene, partial sequence; internal transcribed spacer 1, 5.8S ribosomal RNA gene and internal transcribed spacer 2, complete sequence; and 26S ribosomal RNA gene, partial sequence	876	876	100%	0.0	90%	
AY240006.1	Dendrobium yeageri 18S ribosomal RNA gene, partial sequence; internal transcribed spacer 1, 5.8S ribosomal RNA gene and internal transcribed spacer 2, complete sequence; and 26S ribosomal RNA gene, partial sequence	843	843	100%	0.0	89%	
AY239994.1	Dendrobium serratilabium 18S ribosomal RNA gene, partial sequence; internal transcribed spacer 1, 5.8S ribosomal RNA gene and internal transcribed spacer 2, complete sequence; and 26S ribosomal RNA gene, partial sequence	843	843	100%	0.0	89%	
AY239980.1	Dendrobium mohlianum 18S ribosomal RNA gene, partial sequence; internal transcribed spacer 1, 5.8S ribosomal RNA gene and internal transcribed spacer 2, complete sequence; and 26S ribosomal RNA gene, partial sequence	843	843	100%	0.0	89%	
AY239976.1	Dendrobium lancifolium 18S ribosomal RNA gene, partial sequence; internal transcribed spacer 1, 5.8S ribosomal RNA gene and internal transcribed spacer 2, complete sequence; and 26S ribosomal RNA gene, partial sequence	843	843	100%	0.0	89%	
AY239958.1	Dendrobium ceraula 18S ribosomal RNA gene, partial sequence; internal transcribed spacer 1, 5.8S ribosomal RNA gene and internal transcribed spacer 2, complete sequence; and 26S ribosomal RNA gene, partial sequence	837	837	100%	0.0	89%	
HM590385.1	Dendrobium chameleon voucher NCHU-D89331201-1019 18S ribosomal RNA gene, partial sequence; internal transcribed spacer 1, 5.8S ribosomal RNA gene, and internal transcribed spacer 2, complete sequence; and 26S ribosomal RNA gene, partial sequence	833	833	100%	0.0	89%	
AY240004.1	Dendrobium victoriae-reginae 18S ribosomal RNA gene, partial sequence; internal transcribed spacer 1, 5.8S ribosomal RNA gene and internal transcribed spacer 2, complete sequence; and 26S ribosomal RNA gene, partial sequence	832	832	100%	0.0	89%	
AY239960.1	Dendrobium chameleon 18S ribosomal RNA gene, partial sequence; internal transcribed spacer 1, 5.8S ribosomal RNA gene and internal transcribed spacer 2, complete sequence; and 26S ribosomal RNA gene, partial sequence	832	832	100%	0.0	89%	
[illegible]	[illegible]	[illegible]	[illegible]	[illegible]	[illegible]	[illegible]	

NCBI Blast:AF362036.1 Dendrobium brymerianum - Mozilla Firefox

file:///G:/THESIS/NCBI DOWNLOADED MATK SEQUENCES/ITS/BLAST results 93+36 sequences/NCBI BlastAF362036_1 Dendrobium brymerianum_htm.ht

Legend for links to other resources: UniGene GEO Gene Structure Map Viewer PubChem BioAssay

Sequences producing significant alignments:

Accession	Description	Max score	Total score	Query coverage	E value	Max ident	Links
AF362036.1	Dendrobium brymerianum internal transcribed spacer 1, partial sequence; 5.8S ribosomal RNA gene, complete sequence; and internal transcribed spacer 2, partial sequence	1182	1182	100%	0.0	100%	
EU477500.1	Dendrobium brymerianum internal transcribed spacer 1, partial sequence; 5.8S ribosomal RNA gene and internal transcribed spacer 2, complete sequence; and 28S ribosomal RNA gene, partial sequence	1166	1166	100%	0.0	99%	
AF362040.1	Dendrobium aurantiacum var. denneanum internal transcribed spacer 1, partial sequence; 5.8S ribosomal RNA gene, complete sequence; and internal transcribed spacer 2, partial sequence >gb|FJ384731.1| Dendrobium aurantiacum var. denneanum strain D3 internal transcribed spacer 1, partial sequence; 5.8S ribosomal RNA gene, complete sequence; and internal transcribed spacer 2, partial sequence	878	878	100%	0.0	91%	
DQ058788.1	Dendrobium dixanthum voucher HH-JL03-1 internal transcribed spacer 1, 5.8S ribosomal RNA gene, and internal transcribed spacer 2, complete sequence	850	850	100%	0.0	90%	
FJ428221.1	Dendrobium brymerianum internal transcribed spacer 1, partial sequence; 5.8S ribosomal RNA gene, complete sequence; and internal transcribed spacer 2, partial sequence	850	850	100%	0.0	90%	
GU339103.1	Dendrobium dixanthum internal transcribed spacer 1, partial sequence; 5.8S ribosomal RNA gene, complete sequence; and internal transcribed spacer 2, partial sequence	845	845	100%	0.0	90%	
AF362025.1	Dendrobium hancockii internal transcribed spacer 1, partial sequence; 5.8S ribosomal RNA gene, complete sequence; and internal transcribed spacer 2, partial sequence	845	845	100%	0.0	90%	
HM590375.1	Dendrobium aurantiacum voucher NCHU-D89331201-1009 18S ribosomal RNA gene, partial sequence; internal transcribed spacer 1, 5.8S ribosomal RNA gene, and internal transcribed spacer 2, complete sequence; and 26S ribosomal RNA gene, partial sequence	843	843	100%	0.0	90%	
AF362042.1	Dendrobium aurantiacum isolate Guangxi internal transcribed spacer 1, partial sequence; 5.8S ribosomal RNA gene, complete sequence; and internal transcribed spacer 2, partial sequence	843	843	100%	0.0	90%	
HM590377.1	Dendrobium hancockii voucher NCHU-D89331201-1011 18S ribosomal RNA gene, partial sequence; internal transcribed spacer 1, 5.8S ribosomal RNA gene, and internal transcribed spacer 2, complete sequence; and 26S ribosomal RNA gene, partial sequence	839	839	100%	0.0	90%	
AF362044.1	Dendrobium aurantiacum isolate Kunming Yunnan internal transcribed spacer 1, partial sequence; 5.8S ribosomal RNA gene, complete sequence; and internal transcribed spacer 2, partial sequence	837	837	100%	0.0	90%	
EU477506.1	Dendrobium harveyanum internal transcribed spacer 1, partial sequence; 5.8S ribosomal RNA gene and internal transcribed spacer 2, complete sequence; and 28S ribosomal RNA gene, partial sequence	835	835	100%	0.0	90%	
DQ058787.1	Dendrobium hancockii voucher XY-GZ03-1 internal transcribed spacer 1, 5.8S ribosomal RNA gene, and internal transcribed spacer 2, complete sequence	832	832	100%	0.0	90%	

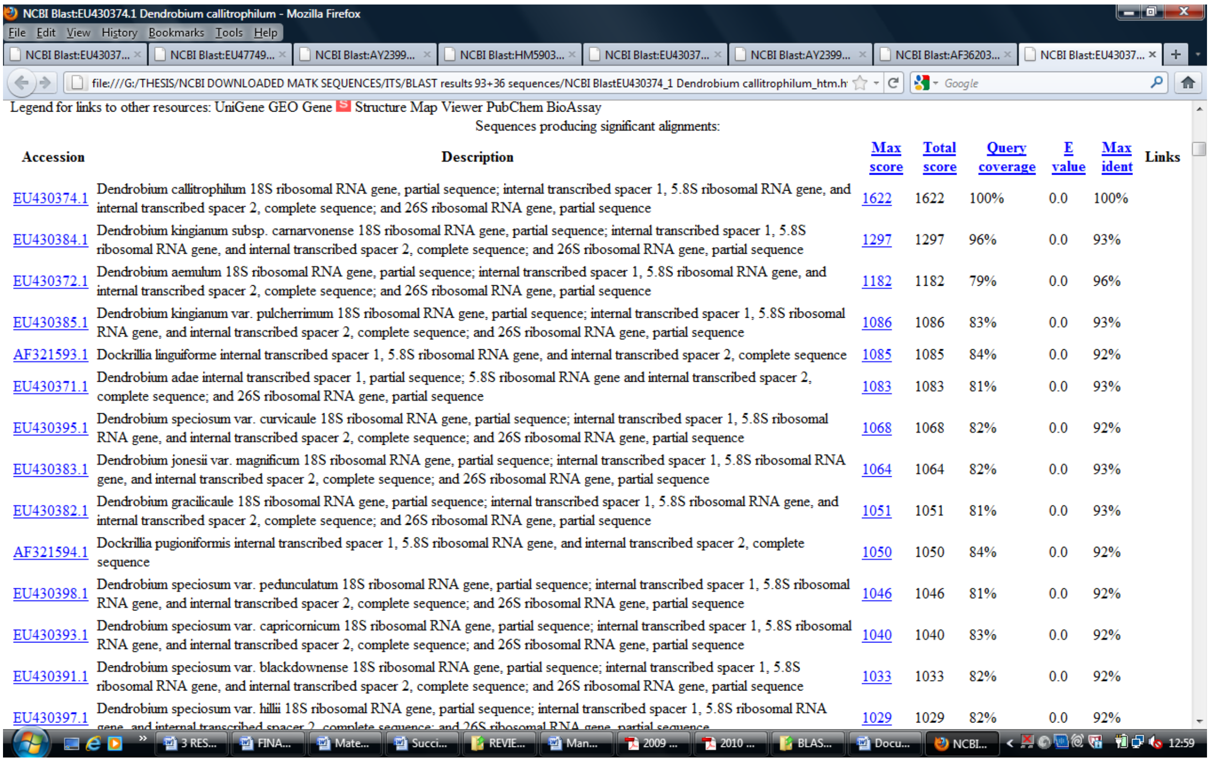

Accession	Description	Max score	Total score	Query coverage	E value	Max ident	Links
EU430374.1	Dendrobium callitrophilum 18S ribosomal RNA gene, partial sequence; internal transcribed spacer 1, 5.8S ribosomal RNA gene, and internal transcribed spacer 2, complete sequence; and 26S ribosomal RNA gene, partial sequence	1622	1622	100%	0.0	100%	
EU430384.1	Dendrobium kingianum subsp. carnarvonense 18S ribosomal RNA gene, partial sequence; internal transcribed spacer 1, 5.8S ribosomal RNA gene, and internal transcribed spacer 2, complete sequence; and 26S ribosomal RNA gene, partial sequence	1297	1297	96%	0.0	93%	
EU430372.1	Dendrobium aemulum 18S ribosomal RNA gene, partial sequence; internal transcribed spacer 1, 5.8S ribosomal RNA gene, and internal transcribed spacer 2, complete sequence; and 26S ribosomal RNA gene, partial sequence	1182	1182	79%	0.0	96%	
EU430385.1	Dendrobium kingianum var. pulcherrimum 18S ribosomal RNA gene, partial sequence; internal transcribed spacer 1, 5.8S ribosomal RNA gene, and internal transcribed spacer 2, complete sequence; and 26S ribosomal RNA gene, partial sequence	1086	1086	83%	0.0	93%	
AF321593.1	Dockrillia linguiforme internal transcribed spacer 1, 5.8S ribosomal RNA gene, and internal transcribed spacer 2, complete sequence	1085	1085	84%	0.0	92%	
EU430371.1	Dendrobium adae internal transcribed spacer 1, partial sequence; 5.8S ribosomal RNA gene and internal transcribed spacer 2, complete sequence; and 26S ribosomal RNA gene, partial sequence	1083	1083	81%	0.0	93%	
EU430395.1	Dendrobium speciosum var. curvicaule 18S ribosomal RNA gene, partial sequence; internal transcribed spacer 1, 5.8S ribosomal RNA gene, and internal transcribed spacer 2, complete sequence; and 26S ribosomal RNA gene, partial sequence	1068	1068	82%	0.0	92%	
EU430383.1	Dendrobium jonesii var. magnificum 18S ribosomal RNA gene, partial sequence; internal transcribed spacer 1, 5.8S ribosomal RNA gene, and internal transcribed spacer 2, complete sequence; and 26S ribosomal RNA gene, partial sequence	1064	1064	82%	0.0	93%	
EU430382.1	Dendrobium gracilicaule 18S ribosomal RNA gene, partial sequence; internal transcribed spacer 1, 5.8S ribosomal RNA gene, and internal transcribed spacer 2, complete sequence; and 26S ribosomal RNA gene, partial sequence	1051	1051	81%	0.0	93%	
AF321594.1	Dockrillia pugioniformis internal transcribed spacer 1, 5.8S ribosomal RNA gene, and internal transcribed spacer 2, complete sequence	1050	1050	84%	0.0	92%	
EU430398.1	Dendrobium speciosum var. pedunculatum 18S ribosomal RNA gene, partial sequence; internal transcribed spacer 1, 5.8S ribosomal RNA gene, and internal transcribed spacer 2, complete sequence; and 26S ribosomal RNA gene, partial sequence	1046	1046	81%	0.0	92%	
EU430393.1	Dendrobium speciosum var. capricornicum 18S ribosomal RNA gene, partial sequence; internal transcribed spacer 1, 5.8S ribosomal RNA gene, and internal transcribed spacer 2, complete sequence; and 26S ribosomal RNA gene, partial sequence	1040	1040	83%	0.0	92%	
EU430391.1	Dendrobium speciosum var. blackdownense 18S ribosomal RNA gene, partial sequence; internal transcribed spacer 1, 5.8S ribosomal RNA gene, and internal transcribed spacer 2, complete sequence; and 26S ribosomal RNA gene, partial sequence	1033	1033	82%	0.0	92%	
EU430397.1	Dendrobium speciosum var. hillii 18S ribosomal RNA gene, partial sequence; internal transcribed spacer 1, 5.8S ribosomal RNA gene, and internal transcribed spacer 2, complete sequence; and 26S ribosomal RNA gene, partial sequence	1029	1029	82%	0.0	92%	

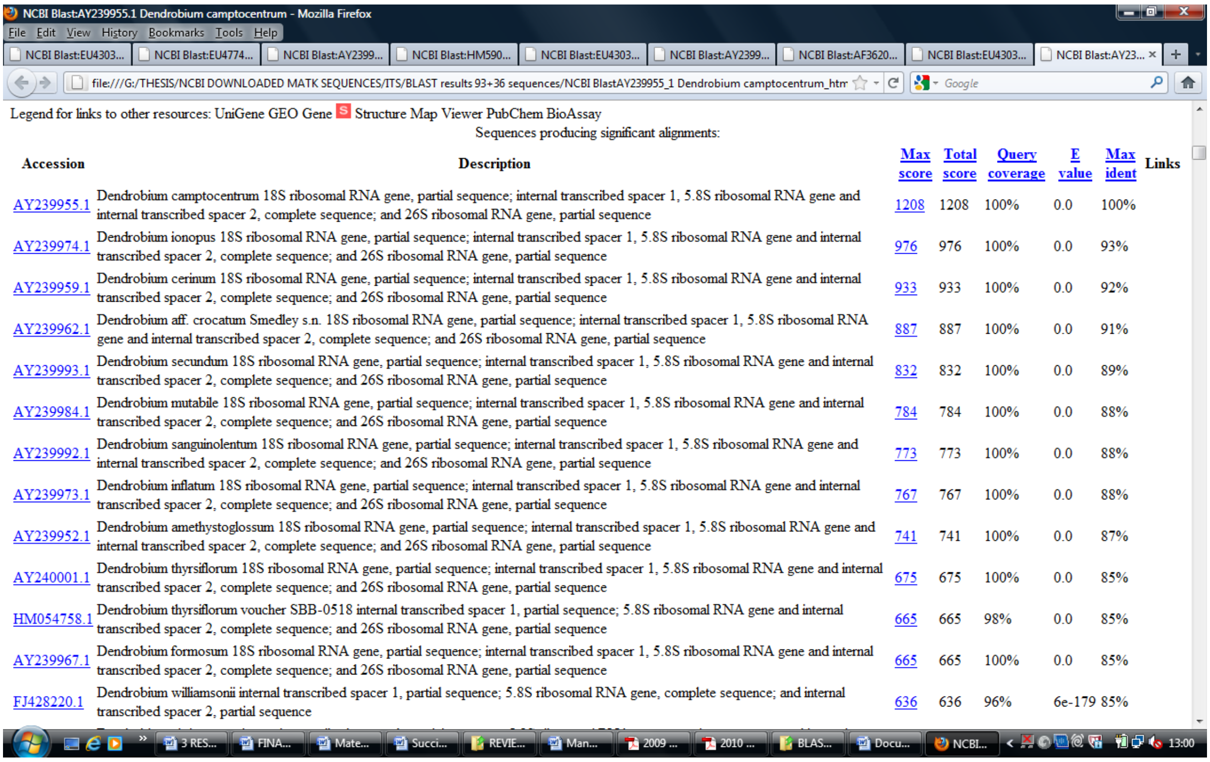

Accession	Description	Max score	Total score	Query coverage	E value	Max ident	Links
AY239955.1	Dendrobium camptocentrum 18S ribosomal RNA gene, partial sequence; internal transcribed spacer 1, 5.8S ribosomal RNA gene and internal transcribed spacer 2, complete sequence; and 26S ribosomal RNA gene, partial sequence	1208	1208	100%	0.0	100%	
AY239974.1	Dendrobium ionopus 18S ribosomal RNA gene, partial sequence; internal transcribed spacer 1, 5.8S ribosomal RNA gene and internal transcribed spacer 2, complete sequence; and 26S ribosomal RNA gene, partial sequence	976	976	100%	0.0	93%	
AY239959.1	Dendrobium cerinum 18S ribosomal RNA gene, partial sequence; internal transcribed spacer 1, 5.8S ribosomal RNA gene and internal transcribed spacer 2, complete sequence; and 26S ribosomal RNA gene, partial sequence	933	933	100%	0.0	92%	
AY239962.1	Dendrobium aff. crocatum Smedley s.n. 18S ribosomal RNA gene, partial sequence; internal transcribed spacer 1, 5.8S ribosomal RNA gene and internal transcribed spacer 2, complete sequence; and 26S ribosomal RNA gene, partial sequence	887	887	100%	0.0	91%	
AY239993.1	Dendrobium secundum 18S ribosomal RNA gene, partial sequence; internal transcribed spacer 1, 5.8S ribosomal RNA gene and internal transcribed spacer 2, complete sequence; and 26S ribosomal RNA gene, partial sequence	832	832	100%	0.0	89%	
AY239984.1	Dendrobium mutabile 18S ribosomal RNA gene, partial sequence; internal transcribed spacer 1, 5.8S ribosomal RNA gene and internal transcribed spacer 2, complete sequence; and 26S ribosomal RNA gene, partial sequence	784	784	100%	0.0	88%	
AY239992.1	Dendrobium sanguinolentum 18S ribosomal RNA gene, partial sequence; internal transcribed spacer 1, 5.8S ribosomal RNA gene and internal transcribed spacer 2, complete sequence; and 26S ribosomal RNA gene, partial sequence	773	773	100%	0.0	88%	
AY239973.1	Dendrobium inflatum 18S ribosomal RNA gene, partial sequence; internal transcribed spacer 1, 5.8S ribosomal RNA gene and internal transcribed spacer 2, complete sequence; and 26S ribosomal RNA gene, partial sequence	767	767	100%	0.0	88%	
AY239952.1	Dendrobium amethystoglossum 18S ribosomal RNA gene, partial sequence; internal transcribed spacer 1, 5.8S ribosomal RNA gene and internal transcribed spacer 2, complete sequence; and 26S ribosomal RNA gene, partial sequence	741	741	100%	0.0	87%	
AY240001.1	Dendrobium thyrsiflorum 18S ribosomal RNA gene, partial sequence; internal transcribed spacer 1, 5.8S ribosomal RNA gene and internal transcribed spacer 2, complete sequence; and 26S ribosomal RNA gene, partial sequence	675	675	100%	0.0	85%	
HM054758.1	Dendrobium thyrsiflorum voucher SBB-0518 internal transcribed spacer 1, partial sequence; 5.8S ribosomal RNA gene and internal transcribed spacer 2, complete sequence; and 26S ribosomal RNA gene, partial sequence	665	665	98%	0.0	85%	
AY239967.1	Dendrobium formosum 18S ribosomal RNA gene, partial sequence; internal transcribed spacer 1, 5.8S ribosomal RNA gene and internal transcribed spacer 2, complete sequence; and 26S ribosomal RNA gene, partial sequence	665	665	100%	0.0	85%	
FJ428220.1	Dendrobium williamsonii internal transcribed spacer 1, partial sequence; 5.8S ribosomal RNA gene, complete sequence; and internal transcribed spacer 2, partial sequence	636	636	96%	6e-179	85%	

NCBI Blast:EU430375.1 Dendrobium canaliculatum - Mozilla Firefox

Legend for links to other resources: UniGene GEO Gene Structure Map Viewer PubChem BioAssay

Sequences producing significant alignments:

Accession	Description	Max score	Total score	Query coverage	E value	Max ident	Links
EU430375.1	Dendrobium canaliculatum 18S ribosomal RNA gene, partial sequence; internal transcribed spacer 1, 5.8S ribosomal RNA gene, and internal transcribed spacer 2, complete sequence; and 26S ribosomal RNA gene, partial sequence	1282	1282	100%	0.0	100%	
EU430383.1	Dendrobium jonesii var. magnificum 18S ribosomal RNA gene, partial sequence; internal transcribed spacer 1, 5.8S ribosomal RNA gene, and internal transcribed spacer 2, complete sequence; and 26S ribosomal RNA gene, partial sequence	990	990	99%	0.0	92%	
EU430382.1	Dendrobium gracilicaule 18S ribosomal RNA gene, partial sequence; internal transcribed spacer 1, 5.8S ribosomal RNA gene, and internal transcribed spacer 2, complete sequence; and 26S ribosomal RNA gene, partial sequence	987	987	98%	0.0	92%	
EU430395.1	Dendrobium speciosum var. curvicaule 18S ribosomal RNA gene, partial sequence; internal transcribed spacer 1, 5.8S ribosomal RNA gene, and internal transcribed spacer 2, complete sequence; and 26S ribosomal RNA gene, partial sequence	985	985	99%	0.0	92%	
AF321593.1	Dockrillia linguiforme internal transcribed spacer 1, 5.8S ribosomal RNA gene, and internal transcribed spacer 2, complete sequence	981	981	99%	0.0	92%	
AY239985.1	Dendrobium nindii 18S ribosomal RNA gene, partial sequence; internal transcribed spacer 1, 5.8S ribosomal RNA gene and internal transcribed spacer 2, complete sequence; and 26S ribosomal RNA gene, partial sequence	977	977	95%	0.0	93%	
EU430384.1	Dendrobium kingianum subsp. carnarvonense 18S ribosomal RNA gene, partial sequence; internal transcribed spacer 1, 5.8S ribosomal RNA gene, and internal transcribed spacer 2, complete sequence; and 26S ribosomal RNA gene, partial sequence	976	976	99%	0.0	92%	
EU430398.1	Dendrobium speciosum var. pedunculatum 18S ribosomal RNA gene, partial sequence; internal transcribed spacer 1, 5.8S ribosomal RNA gene, and internal transcribed spacer 2, complete sequence; and 26S ribosomal RNA gene, partial sequence	966	966	99%	0.0	91%	
EU430392.1	Dendrobium speciosum var. boreale 18S ribosomal RNA gene, partial sequence; internal transcribed spacer 1, 5.8S ribosomal RNA gene, and internal transcribed spacer 2, complete sequence; and 26S ribosomal RNA gene, partial sequence	965	965	98%	0.0	92%	
EU430377.1	Dendrobium falcorostrum 18S ribosomal RNA gene, partial sequence; internal transcribed spacer 1, 5.8S ribosomal RNA gene, and internal transcribed spacer 2, complete sequence; and 26S ribosomal RNA gene, partial sequence	961	961	96%	0.0	92%	
EU430389.1	Dendrobium racemosum 18S ribosomal RNA gene, partial sequence; internal transcribed spacer 1, 5.8S ribosomal RNA gene, and internal transcribed spacer 2, complete sequence; and 26S ribosomal RNA gene, partial sequence	959	959	99%	0.0	91%	
EU430376.1	Dendrobium carrii 18S ribosomal RNA gene, partial sequence; internal transcribed spacer 1, 5.8S ribosomal RNA gene, and internal transcribed spacer 2, complete sequence; and 26S ribosomal RNA gene, partial sequence	957	957	99%	0.0	91%	
EU430388.1	Dendrobium moorei 18S ribosomal RNA gene, partial sequence; internal transcribed spacer 1 and 5.8S ribosomal RNA gene, complete sequence; and internal transcribed spacer 2, partial sequence	952	952	96%	0.0	92%	
EU430380.1	Dendrobium fleckeri 18S ribosomal RNA gene, partial sequence; internal transcribed spacer 1, 5.8S ribosomal RNA gene, and internal transcribed spacer 2, complete sequence; and 26S ribosomal RNA gene, partial sequence	952	952	97%	0.0	91%	

NCBI Blast:AY239956.1 Dendrobium capituliflorum - Mozilla Firefox

Legend for links to other resources: UniGene GEO Gene Structure Map Viewer PubChem BioAssay

Sequences producing significant alignments:

Accession	Description	Max score	Total score	Query coverage	E value	Max ident	Links
AY239956.1	Dendrobium capituliflorum 18S ribosomal RNA gene, partial sequence; internal transcribed spacer 1, 5.8S ribosomal RNA gene and internal transcribed spacer 2, complete sequence; and 26S ribosomal RNA gene, partial sequence	1219	1219	100%	0.0	100%	
AY239994.1	Dendrobium serratilabium 18S ribosomal RNA gene, partial sequence; internal transcribed spacer 1, 5.8S ribosomal RNA gene and internal transcribed spacer 2, complete sequence; and 26S ribosomal RNA gene, partial sequence	839	839	100%	0.0	89%	
AY239957.1	Dendrobium cauliculimentum 18S ribosomal RNA gene, partial sequence; internal transcribed spacer 1, 5.8S ribosomal RNA gene and internal transcribed spacer 2, complete sequence; and 26S ribosomal RNA gene, partial sequence	833	833	100%	0.0	89%	
HM590385.1	Dendrobium chameleon voucher NCHU-D89331201-1019 18S ribosomal RNA gene, partial sequence; internal transcribed spacer 1, 5.8S ribosomal RNA gene, and internal transcribed spacer 2, complete sequence; and 26S ribosomal RNA gene, partial sequence	830	830	100%	0.0	89%	
AY240006.1	Dendrobium yeageri 18S ribosomal RNA gene, partial sequence; internal transcribed spacer 1, 5.8S ribosomal RNA gene and internal transcribed spacer 2, complete sequence; and 26S ribosomal RNA gene, partial sequence	828	828	100%	0.0	89%	
AY239976.1	Dendrobium lancifolium 18S ribosomal RNA gene, partial sequence; internal transcribed spacer 1, 5.8S ribosomal RNA gene and internal transcribed spacer 2, complete sequence; and 26S ribosomal RNA gene, partial sequence	828	828	100%	0.0	89%	
AY239960.1	Dendrobium chameleon 18S ribosomal RNA gene, partial sequence; internal transcribed spacer 1, 5.8S ribosomal RNA gene and internal transcribed spacer 2, complete sequence; and 26S ribosomal RNA gene, partial sequence	828	828	100%	0.0	89%	
AY239958.1	Dendrobium ceraula 18S ribosomal RNA gene, partial sequence; internal transcribed spacer 1, 5.8S ribosomal RNA gene and internal transcribed spacer 2, complete sequence; and 26S ribosomal RNA gene, partial sequence	822	822	100%	0.0	89%	
AY240004.1	Dendrobium victoriae-reginae 18S ribosomal RNA gene, partial sequence; internal transcribed spacer 1, 5.8S ribosomal RNA gene and internal transcribed spacer 2, complete sequence; and 26S ribosomal RNA gene, partial sequence	817	817	100%	0.0	89%	
AY239991.1	Dendrobium rhododioides 18S ribosomal RNA gene, partial sequence; internal transcribed spacer 1, 5.8S ribosomal RNA gene and internal transcribed spacer 2, complete sequence; and 26S ribosomal RNA gene, partial sequence	811	811	100%	0.0	88%	
AY239980.1	Dendrobium mohlianum 18S ribosomal RNA gene, partial sequence; internal transcribed spacer 1, 5.8S ribosomal RNA gene and internal transcribed spacer 2, complete sequence; and 26S ribosomal RNA gene, partial sequence	811	811	100%	0.0	88%	
HM590386.1	Dendrobium miyakei voucher NCHU-D89331201-1020 18S ribosomal RNA gene, partial sequence; internal transcribed spacer 1, 5.8S ribosomal RNA gene, and internal transcribed spacer 2, complete sequence; and 26S ribosomal RNA gene, partial sequence	797	797	100%	0.0	88%	
AY239970.1	Dendrobium goldschmidtianum 18S ribosomal RNA gene, partial sequence; internal transcribed spacer 1, 5.8S ribosomal RNA gene and internal transcribed spacer 2, complete sequence; and 26S ribosomal RNA gene, partial sequence	797	797	100%	0.0	88%	

NCBI Blast:AY485710.1 Dendrobium cariniferum - Mozilla Firefox

file:///G:/THESIS/NCBI DOWNLOADED MATK SEQUENCES/ITS/BLAST results 93+36 sequences/NCBI BlastAY485710_1 Dendrobium cariniferum_htm.htm

Legend for links to other resources: UniGene GEO Gene Structure Map Viewer PubChem BioAssay

Sequences producing significant alignments:

Accession	Description	Max score	Total score	Query coverage	E value	Max ident	Links
AY485710.1	Dendrobium cariniferum specimen-voucher ICMdcar2204-1 internal transcribed spacer 1, partial sequence; 5.8S ribosomal RNA gene, complete sequence; and internal transcribed spacer 2, partial sequence	1181	1181	100%	0.0	100%	
AY485711.1	Dendrobium gratiosissimum specimen-voucher ICMdgra2201-1 internal transcribed spacer 1, partial sequence; 5.8S ribosomal RNA gene, complete sequence; and internal transcribed spacer 2, partial sequence	813	813	100%	0.0	89%	
AY485714.1	Dendrobium fimbriatum specimen-voucher XYD0006-02 internal transcribed spacer 1, partial sequence; 5.8S ribosomal RNA gene, complete sequence; and internal transcribed spacer 2, partial sequence	787	787	100%	0.0	88%	
AY485694.1	Dendrobium hercoglossum specimen-voucher ICMdher2103-1 internal transcribed spacer 1, partial sequence; 5.8S ribosomal RNA gene, complete sequence; and internal transcribed spacer 2, partial sequence	773	773	100%	0.0	88%	
DQ058789.1	Dendrobium wardianum voucher DBQ-JL04-01 internal transcribed spacer 1, 5.8S ribosomal RNA gene, and internal transcribed spacer 2, complete sequence	767	767	97%	0.0	89%	
HM054544.1	Dendrobium amoenum voucher SBB-0560 internal transcribed spacer 1, partial sequence; 5.8S ribosomal RNA gene and internal transcribed spacer 2, complete sequence; and 26S ribosomal RNA gene, partial sequence	761	761	98%	0.0	88%	
AF420245.1	Dendrobium wardianum internal transcribed spacer 1, partial sequence; 5.8S ribosomal RNA gene, complete sequence; and internal transcribed spacer 2, partial sequence	761	761	96%	0.0	89%	
HM054546.1	Dendrobium amoenum voucher SBB-0576 internal transcribed spacer 1, partial sequence; 5.8S ribosomal RNA gene and internal transcribed spacer 2, complete sequence; and 26S ribosomal RNA gene, partial sequence	758	758	97%	0.0	88%	
HM054538.1	Dendrobium amoenum voucher SBB-0140 internal transcribed spacer 1, partial sequence; 5.8S ribosomal RNA gene and internal transcribed spacer 2, complete sequence; and 26S ribosomal RNA gene, partial sequence	758	758	98%	0.0	88%	
HM054537.1	Dendrobium amoenum voucher SBB-0139 internal transcribed spacer 1, partial sequence; 5.8S ribosomal RNA gene and internal transcribed spacer 2, complete sequence; and 26S ribosomal RNA gene, partial sequence	758	758	98%	0.0	88%	
HM054534.1	Dendrobium amoenum voucher SBB-0135 internal transcribed spacer 1, partial sequence; 5.8S ribosomal RNA gene and internal transcribed spacer 2, complete sequence; and 26S ribosomal RNA gene, partial sequence	758	758	98%	0.0	88%	
HM054536.1	Dendrobium amoenum voucher SBB-0138 internal transcribed spacer 1, partial sequence; 5.8S ribosomal RNA gene and internal transcribed spacer 2, complete sequence; and 26S ribosomal RNA gene, partial sequence	758	758	98%	0.0	88%	
HM054539.1	Dendrobium amoenum voucher SBB-0142 internal transcribed spacer 1, partial sequence; 5.8S ribosomal RNA gene and internal transcribed spacer 2, complete sequence; and 26S ribosomal RNA gene, partial sequence	756	756	98%	0.0	88%	
[illegible]	Dendrobium amoenum voucher SBB-0247 internal transcribed spacer 1, partial sequence; 5.8S ribosomal RNA gene and internal transcribed [illegible]	[illegible]	[illegible]	[illegible]	[illegible]	[illegible]	

NCBI Blast:EU430376.1 Dendrobium carrii - Mozilla Firefox

file:///G:/THESIS/NCBI DOWNLOADED MATK SEQUENCES/ITS/BLAST results 93+36 sequences/NCBI BlastEU430376_1 Dendrobium carrii_htm.htm

Legend for links to other resources: UniGene GEO Gene Structure Map Viewer PubChem BioAssay

Sequences producing significant alignments:

Accession	Description	Max score	Total score	Query coverage	E value	Max ident	Links
EU430376.1	Dendrobium carrii 18S ribosomal RNA gene, partial sequence; internal transcribed spacer 1, 5.8S ribosomal RNA gene, and internal transcribed spacer 2, complete sequence; and 26S ribosomal RNA gene, partial sequence	1376	1376	100%	0.0	100%	
EU430395.1	Dendrobium speciosum var. curvicaule 18S ribosomal RNA gene, partial sequence; internal transcribed spacer 1, 5.8S ribosomal RNA gene, and internal transcribed spacer 2, complete sequence; and 26S ribosomal RNA gene, partial sequence	1127	1127	97%	0.0	94%	
EU430385.1	Dendrobium kingianum var. pulcherrimum 18S ribosomal RNA gene, partial sequence; internal transcribed spacer 1, 5.8S ribosomal RNA gene, and internal transcribed spacer 2, complete sequence; and 26S ribosomal RNA gene, partial sequence	1114	1114	99%	0.0	93%	
EU430387.1	Dendrobium monophyllum 18S ribosomal RNA gene, partial sequence; internal transcribed spacer 1, 5.8S ribosomal RNA gene, and internal transcribed spacer 2, complete sequence; and 26S ribosomal RNA gene, partial sequence	1112	1112	93%	0.0	95%	
EU430384.1	Dendrobium kingianum subsp. carnarvonense 18S ribosomal RNA gene, partial sequence; internal transcribed spacer 1, 5.8S ribosomal RNA gene, and internal transcribed spacer 2, complete sequence; and 26S ribosomal RNA gene, partial sequence	1109	1109	98%	0.0	93%	
EU430383.1	Dendrobium jonesii var. magnificum 18S ribosomal RNA gene, partial sequence; internal transcribed spacer 1, 5.8S ribosomal RNA gene, and internal transcribed spacer 2, complete sequence; and 26S ribosomal RNA gene, partial sequence	1107	1107	97%	0.0	94%	
EU430398.1	Dendrobium speciosum var. pedunculatum 18S ribosomal RNA gene, partial sequence; internal transcribed spacer 1, 5.8S ribosomal RNA gene, and internal transcribed spacer 2, complete sequence; and 26S ribosomal RNA gene, partial sequence	1098	1098	96%	0.0	94%	
EU430382.1	Dendrobium gracilicaule 18S ribosomal RNA gene, partial sequence; internal transcribed spacer 1, 5.8S ribosomal RNA gene, and internal transcribed spacer 2, complete sequence; and 26S ribosomal RNA gene, partial sequence	1098	1098	96%	0.0	94%	
EU430393.1	Dendrobium speciosum var. capricornicum 18S ribosomal RNA gene, partial sequence; internal transcribed spacer 1, 5.8S ribosomal RNA gene, and internal transcribed spacer 2, complete sequence; and 26S ribosomal RNA gene, partial sequence	1085	1085	99%	0.0	93%	
EU430402.1	Dendrobium tetragonum var. melaleucaphilum 18S ribosomal RNA gene, partial sequence; internal transcribed spacer 1, 5.8S ribosomal RNA gene, and internal transcribed spacer 2, complete sequence; and 26S ribosomal RNA gene, partial sequence	1081	1081	98%	0.0	93%	
EU430391.1	Dendrobium speciosum var. blackdownense 18S ribosomal RNA gene, partial sequence; internal transcribed spacer 1, 5.8S ribosomal RNA gene, and internal transcribed spacer 2, complete sequence; and 26S ribosomal RNA gene, partial sequence	1077	1077	98%	0.0	93%	
EU430397.1	Dendrobium speciosum var. hillii 18S ribosomal RNA gene, partial sequence; internal transcribed spacer 1, 5.8S ribosomal RNA gene, and internal transcribed spacer 2, complete sequence; and 26S ribosomal RNA gene, partial sequence	1075	1075	98%	0.0	93%	
EU430396.1	Dendrobium speciosum var. grandiflorum 18S ribosomal RNA gene, partial sequence; internal transcribed spacer 1, 5.8S ribosomal RNA gene, and internal transcribed spacer 2, complete sequence; and 26S ribosomal RNA gene, partial sequence	1074	1074	98%	0.0	93%	

NCBI Blast:HM590385.1 Dendrobium chameleon - Mozilla Firefox

file:///G:/THESIS/NCBI DOWNLOADED MATK SEQUENCES/ITS/BLAST results 93+36 sequences/NCBI BlastHM590385_1 Dendrobium chameleon_htm.htn

Legend for links to other resources: UniGene GEO Gene Structure Map Viewer PubChem BioAssay

Sequences producing significant alignments:

Accession	Description	Max score	Total score	Query coverage	E value	Max ident	Links
HM590385.1	Dendrobium chameleon voucher NCHU-D89331201-1019 18S ribosomal RNA gene, partial sequence; internal transcribed spacer 1, 5.8S ribosomal RNA gene, and internal transcribed spacer 2, complete sequence; and 26S ribosomal RNA gene, partial sequence	1637	1637	100%	0.0	100%	
HM590386.1	Dendrobium miyakei voucher NCHU-D89331201-1020 18S ribosomal RNA gene, partial sequence; internal transcribed spacer 1, 5.8S ribosomal RNA gene, and internal transcribed spacer 2, complete sequence; and 26S ribosomal RNA gene, partial sequence	1471	1471	99%	0.0	96%	
AY239994.1	Dendrobium serratilabium 18S ribosomal RNA gene, partial sequence; internal transcribed spacer 1, 5.8S ribosomal RNA gene and internal transcribed spacer 2, complete sequence; and 26S ribosomal RNA gene, partial sequence	1208	1208	74%	0.0	99%	
AY239960.1	Dendrobium chameleon 18S ribosomal RNA gene, partial sequence; internal transcribed spacer 1, 5.8S ribosomal RNA gene and internal transcribed spacer 2, complete sequence; and 26S ribosomal RNA gene, partial sequence	1120	1120	74%	0.0	97%	
AY240006.1	Dendrobium yeageri 18S ribosomal RNA gene, partial sequence; internal transcribed spacer 1, 5.8S ribosomal RNA gene and internal transcribed spacer 2, complete sequence; and 26S ribosomal RNA gene, partial sequence	1114	1114	74%	0.0	96%	
AY239958.1	Dendrobium ceraula 18S ribosomal RNA gene, partial sequence; internal transcribed spacer 1, 5.8S ribosomal RNA gene and internal transcribed spacer 2, complete sequence; and 26S ribosomal RNA gene, partial sequence	1103	1103	74%	0.0	96%	
AY240004.1	Dendrobium victoriae-reginae 18S ribosomal RNA gene, partial sequence; internal transcribed spacer 1, 5.8S ribosomal RNA gene and internal transcribed spacer 2, complete sequence; and 26S ribosomal RNA gene, partial sequence	1098	1098	74%	0.0	96%	
AY239970.1	Dendrobium goldschmidtianum 18S ribosomal RNA gene, partial sequence; internal transcribed spacer 1, 5.8S ribosomal RNA gene and internal transcribed spacer 2, complete sequence; and 26S ribosomal RNA gene, partial sequence	1083	1083	74%	0.0	96%	
AF521607.1	Dendrobium chameleon internal transcribed spacer 1, 5.8S ribosomal RNA gene, and internal transcribed spacer 2, complete sequence	1077	1077	72%	0.0	97%	
AF521614.1	Dendrobium miyakei internal transcribed spacer 1, 5.8S ribosomal RNA gene, and internal transcribed spacer 2, complete sequence	1046	1046	72%	0.0	96%	
EU840695.1	Dendrobium goldschmidtianum internal transcribed spacer 1, 5.8S ribosomal RNA gene, and internal transcribed spacer 2, complete sequence	1040	1040	72%	0.0	95%	
HM590377.1	Dendrobium hancockii voucher NCHU-D89331201-1011 18S ribosomal RNA gene, partial sequence; internal transcribed spacer 1, 5.8S ribosomal RNA gene, and internal transcribed spacer 2, complete sequence; and 26S ribosomal RNA gene, partial sequence	1018	1018	99%	0.0	87%	
EU840694.1	Dendrobium victoriae-reginae internal transcribed spacer 1, 5.8S ribosomal RNA gene, and internal transcribed spacer 2, complete sequence	1018	1018	72%	0.0	95%	
HM590370.1	Dendrobium crumenatum voucher NCHU-D89331201-1004 18S ribosomal RNA gene, partial sequence; internal transcribed spacer 1, 5.8S ribosomal RNA gene, and internal transcribed spacer 2, complete sequence; and 26S ribosomal RNA gene, partial sequence	1000	1000	100%	0.0	87%	

NCBI Blast:HM590387.1 Dendrobium clavatum - Mozilla Firefox

file:///G:/THESIS/NCBI DOWNLOADED MATK SEQUENCES/ITS/BLAST results 93+36 sequences/NCBI BlastHM590387_1 Dendrobium clavatum_htm.htm

Legend for links to other resources: UniGene GEO Gene Structure Map Viewer PubChem BioAssay

Sequences producing significant alignments:

Accession	Description	Max score	Total score	Query coverage	E value	Max ident	Links
HM590387.1	Dendrobium clavatum voucher NCHU-D89331201-1021 18S ribosomal RNA gene, partial sequence; internal transcribed spacer 1, 5.8S ribosomal RNA gene, and internal transcribed spacer 2, complete sequence; and 26S ribosomal RNA gene, partial sequence	1637	1637	100%	0.0	100%	
HM590377.1	Dendrobium hancockii voucher NCHU-D89331201-1011 18S ribosomal RNA gene, partial sequence; internal transcribed spacer 1, 5.8S ribosomal RNA gene, and internal transcribed spacer 2, complete sequence; and 26S ribosomal RNA gene, partial sequence	1186	1186	99%	0.0	90%	
EU121419.1	Dendrobium clavatum var. aurantiacum internal transcribed spacer 1, 5.8S ribosomal RNA gene, and internal transcribed spacer 2, complete sequence	1173	1173	72%	0.0	99%	
HM590383.1	Dendrobium chrysotoxum voucher NCHU-D89331201-1017 18S ribosomal RNA gene, partial sequence; internal transcribed spacer 1, 5.8S ribosomal RNA gene, and internal transcribed spacer 2, complete sequence; and 26S ribosomal RNA gene, partial sequence	1171	1171	100%	0.0	90%	
AF521606.1	Dendrobium aurantiacum internal transcribed spacer 1, 5.8S ribosomal RNA gene, and internal transcribed spacer 2, complete sequence	1168	1168	72%	0.0	99%	
HM590375.1	Dendrobium aurantiacum voucher NCHU-D89331201-1009 18S ribosomal RNA gene, partial sequence; internal transcribed spacer 1, 5.8S ribosomal RNA gene, and internal transcribed spacer 2, complete sequence; and 26S ribosomal RNA gene, partial sequence	1166	1166	99%	0.0	90%	
HM590392.1	Dendrobium fimbriatum voucher NCHU-D89331201-1029 18S ribosomal RNA gene, partial sequence; internal transcribed spacer 1, 5.8S ribosomal RNA gene, and internal transcribed spacer 2, complete sequence; and 26S ribosomal RNA gene, partial sequence	1149	1149	99%	0.0	90%	
AF362043.1	Dendrobium aurantiacum var. denneanum isolate Xishuangbanna Yunnan internal transcribed spacer 1, partial sequence; 5.8S ribosomal RNA gene, complete sequence; and internal transcribed spacer 2, partial sequence >gb|EU840702.1| Dendrobium aurantiacum var. denneanum internal transcribed spacer 1, 5.8S ribosomal RNA gene, and internal transcribed spacer 2, complete sequence >gb|FJ384732.1| Dendrobium aurantiacum var. denneanum strain D4 internal transcribed spacer 1, partial sequence; 5.8S ribosomal RNA gene, complete sequence; and internal transcribed spacer 2, partial sequence	1140	1140	72%	0.0	98%	
GU339113.1	Dendrobium aurantiacum var. denneanum internal transcribed spacer 1, partial sequence; 5.8S ribosomal RNA gene, complete sequence; and internal transcribed spacer 2, partial sequence	1134	1134	72%	0.0	98%	
FJ384730.1	Dendrobium aurantiacum var. denneanum strain D2 internal transcribed spacer 1, partial sequence; 5.8S ribosomal RNA gene, complete sequence; and internal transcribed spacer 2, partial sequence	1134	1134	72%	0.0	98%	
FJ530949.1	Dendrobium aurantiacum var. denneanum strain D5 internal transcribed spacer 1, partial sequence; 5.8S ribosomal RNA gene, complete sequence; and internal transcribed spacer 2, partial sequence	1134	1134	72%	0.0	98%	
FJ384729.1	Dendrobium aurantiacum var. denneanum strain D1 internal transcribed spacer 1, partial sequence; 5.8S ribosomal RNA gene, complete sequence; and internal transcribed spacer 2, partial sequence	1123	1123	72%	0.0	98%	
	Dendrobium capillipes voucher NCHU-D89331201-1013 18S ribosomal RNA gene, partial sequence; internal transcribed spacer 1, 5.8S [illegible]						

NCBI Blast:AY239961.1 Dendrobium confusum - Mozilla Firefox

file:///G:/THESIS/NCBI DOWNLOADED MATK SEQUENCES/ITS/BLAST results 93+36 sequences/NCBI BlastAY239961_1 Dendrobium confusum_htm.htm

Legend for links to other resources: UniGene GEO Gene Structure Map Viewer PubChem BioAssay

Sequences producing significant alignments:

Accession	Description	Max score	Total score	Query coverage	E value	Max ident	Links
AY239961.1	Dendrobium confusum 18S ribosomal RNA gene, partial sequence; internal transcribed spacer 1, 5.8S ribosomal RNA gene and internal transcribed spacer 2, complete sequence; and 26S ribosomal RNA gene, partial sequence	1230	1230	100%	0.0	100%	
AY239951.1	Dendrobium aloifolium 18S ribosomal RNA gene, partial sequence; internal transcribed spacer 1, 5.8S ribosomal RNA gene and internal transcribed spacer 2, complete sequence; and 26S ribosomal RNA gene, partial sequence	1147	1147	100%	0.0	97%	
AY239953.1	Dendrobium anceps 18S ribosomal RNA gene, partial sequence; internal transcribed spacer 1, 5.8S ribosomal RNA gene and internal transcribed spacer 2, complete sequence; and 26S ribosomal RNA gene, partial sequence	1131	1131	100%	0.0	97%	
AY239972.1	Dendrobium indivisum 18S ribosomal RNA gene, partial sequence; internal transcribed spacer 1, 5.8S ribosomal RNA gene and internal transcribed spacer 2, complete sequence; and 26S ribosomal RNA gene, partial sequence	1120	1120	100%	0.0	97%	
HM054548.1	Dendrobium anceps voucher SBB-0301 18S ribosomal RNA gene, partial sequence; internal transcribed spacer 1, 5.8S ribosomal RNA gene, and internal transcribed spacer 2, complete sequence; and 26S ribosomal RNA gene, partial sequence	1107	1107	97%	0.0	97%	
HM054547.1	Dendrobium anceps voucher SBB-0119 internal transcribed spacer 1, partial sequence; 5.8S ribosomal RNA gene, complete sequence; and internal transcribed spacer 2, partial sequence	1062	1062	93%	0.0	97%	
AY239978.1	Dendrobium leonis 18S ribosomal RNA gene, partial sequence; internal transcribed spacer 1, 5.8S ribosomal RNA gene and internal transcribed spacer 2, complete sequence; and 26S ribosomal RNA gene, partial sequence	1061	1061	100%	0.0	95%	
AF362034.1	Dendrobium acinaciforme internal transcribed spacer 1, partial sequence; 5.8S ribosomal RNA gene, complete sequence; and internal transcribed spacer 2, partial sequence >gb|EF629321.1| Dendrobium jenkinsii internal transcribed spacer 1, partial sequence; 5.8S ribosomal RNA gene, complete sequence; and internal transcribed spacer 2, partial sequence	1013	1013	96%	0.0	95%	
DQ058801.1	Dendrobium terminale voucher DY-ML03-1 internal transcribed spacer 1, 5.8S ribosomal RNA gene, and internal transcribed spacer 2, complete sequence	1011	1011	96%	0.0	95%	
AY239969.1	Dendrobium goldfinchii 18S ribosomal RNA gene, partial sequence; internal transcribed spacer 1, 5.8S ribosomal RNA gene and internal transcribed spacer 2, complete sequence; and 26S ribosomal RNA gene, partial sequence	972	972	100%	0.0	93%	
AY239988.1	Dendrobium philippinense 18S ribosomal RNA gene, partial sequence; internal transcribed spacer 1, 5.8S ribosomal RNA gene and internal transcribed spacer 2, complete sequence; and 26S ribosomal RNA gene, partial sequence	952	952	100%	0.0	92%	
AY239975.1	Dendrobium junceum 18S ribosomal RNA gene, partial sequence; internal transcribed spacer 1, 5.8S ribosomal RNA gene and internal transcribed spacer 2, complete sequence; and 26S ribosomal RNA gene, partial sequence	948	948	100%	0.0	92%	
AY239989.1	Dendrobium quadrangulare 18S ribosomal RNA gene, partial sequence; internal transcribed spacer 1, 5.8S ribosomal RNA gene and internal transcribed spacer 2, complete sequence; and 26S ribosomal RNA gene, partial sequence	937	937	100%	0.0	92%	

NCBI Blast:GU339116.1 Dendrobium crystallinum - Mozilla Firefox

file:///G:/THESIS/NCBI DOWNLOADED MATK SEQUENCES/ITS/BLAST results 93+36 sequences/NCBI BlastGU339116_1 Dendrobium crystallinum_htm.htr

Legend for links to other resources: UniGene GEO Gene Structure Map Viewer PubChem BioAssay

Sequences producing significant alignments:

Accession	Description	Max score	Total score	Query coverage	E value	Max ident	Links
GU339116.1	Dendrobium crystallinum internal transcribed spacer 1, partial sequence; 5.8S ribosomal RNA gene, complete sequence; and internal transcribed spacer 2, partial sequence	1182	1182	100%	0.0	100%	
HM054579.1	Dendrobium bensoniae voucher SBB-0543 internal transcribed spacer 1, partial sequence; 5.8S ribosomal RNA gene and internal transcribed spacer 2, complete sequence; and 26S ribosomal RNA gene, partial sequence	1074	1074	98%	0.0	97%	
HM054580.1	Dendrobium bensoniae voucher SBB-0544 internal transcribed spacer 1, partial sequence; 5.8S ribosomal RNA gene and internal transcribed spacer 2, complete sequence; and 26S ribosomal RNA gene, partial sequence	1068	1068	97%	0.0	97%	
AF363023.1	Dendrobium crystallinum internal transcribed spacer 1, partial sequence; 5.8S ribosomal RNA gene, complete sequence; and internal transcribed spacer 2, partial sequence	1003	1003	100%	0.0	95%	
DQ058790.1	Dendrobium gratiosissimum voucher BQ-JL0401-1 internal transcribed spacer 1, 5.8S ribosomal RNA gene, and internal transcribed spacer 2, complete sequence	929	929	100%	0.0	92%	
DQ058789.1	Dendrobium wardianum voucher DBQ-JL04-01 internal transcribed spacer 1, 5.8S ribosomal RNA gene, and internal transcribed spacer 2, complete sequence	924	924	100%	0.0	92%	
FJ384737.1	Dendrobium gratiosissimum internal transcribed spacer 1, partial sequence; 5.8S ribosomal RNA gene, complete sequence; and internal transcribed spacer 2, partial sequence	924	924	100%	0.0	92%	
HM054544.1	Dendrobium amoenum voucher SBB-0560 internal transcribed spacer 1, partial sequence; 5.8S ribosomal RNA gene and internal transcribed spacer 2, complete sequence; and 26S ribosomal RNA gene, partial sequence	920	920	100%	0.0	92%	
HM054538.1	Dendrobium amoenum voucher SBB-0140 internal transcribed spacer 1, partial sequence; 5.8S ribosomal RNA gene and internal transcribed spacer 2, complete sequence; and 26S ribosomal RNA gene, partial sequence	917	917	100%	0.0	92%	
HM054539.1	Dendrobium amoenum voucher SBB-0142 internal transcribed spacer 1, partial sequence; 5.8S ribosomal RNA gene and internal transcribed spacer 2, complete sequence; and 26S ribosomal RNA gene, partial sequence	915	915	100%	0.0	92%	
HM054540.1	Dendrobium amoenum voucher SBB-0247 internal transcribed spacer 1, partial sequence; 5.8S ribosomal RNA gene and internal transcribed spacer 2, complete sequence; and 26S ribosomal RNA gene, partial sequence	915	915	100%	0.0	92%	
HM054534.1	Dendrobium amoenum voucher SBB-0135 internal transcribed spacer 1, partial sequence; 5.8S ribosomal RNA gene and internal transcribed spacer 2, complete sequence; and 26S ribosomal RNA gene, partial sequence	915	915	99%	0.0	92%	
HM054536.1	Dendrobium amoenum voucher SBB-0138 internal transcribed spacer 1, partial sequence; 5.8S ribosomal RNA gene and internal transcribed spacer 2, complete sequence; and 26S ribosomal RNA gene, partial sequence	915	915	99%	0.0	92%	

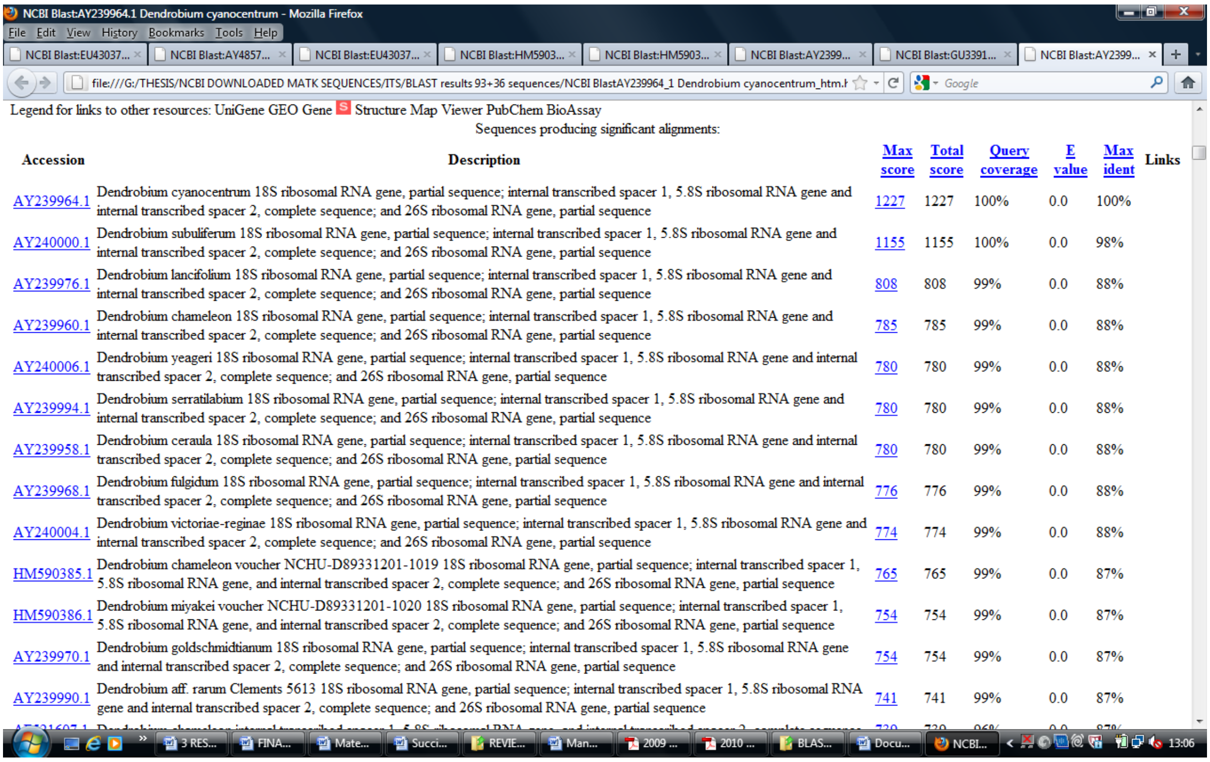
NCBI Blast:AY239964.1 Dendrobium cyanocentrum - Mozilla Firefox

Legend for links to other resources: UniGene GEO Gene Structure Map Viewer PubChem BioAssay

Sequences producing significant alignments:

Accession	Description	Max score	Total score	Query coverage	E value	Max ident	Links
AY239964.1	Dendrobium cyanocentrum 18S ribosomal RNA gene, partial sequence; internal transcribed spacer 1, 5.8S ribosomal RNA gene and internal transcribed spacer 2, complete sequence; and 26S ribosomal RNA gene, partial sequence	1227	1227	100%	0.0	100%	
AY240000.1	Dendrobium subuliferum 18S ribosomal RNA gene, partial sequence; internal transcribed spacer 1, 5.8S ribosomal RNA gene and internal transcribed spacer 2, complete sequence; and 26S ribosomal RNA gene, partial sequence	1155	1155	100%	0.0	98%	
AY239976.1	Dendrobium lancifolium 18S ribosomal RNA gene, partial sequence; internal transcribed spacer 1, 5.8S ribosomal RNA gene and internal transcribed spacer 2, complete sequence; and 26S ribosomal RNA gene, partial sequence	808	808	99%	0.0	88%	
AY239960.1	Dendrobium chameleon 18S ribosomal RNA gene, partial sequence; internal transcribed spacer 1, 5.8S ribosomal RNA gene and internal transcribed spacer 2, complete sequence; and 26S ribosomal RNA gene, partial sequence	785	785	99%	0.0	88%	
AY240006.1	Dendrobium yeageri 18S ribosomal RNA gene, partial sequence; internal transcribed spacer 1, 5.8S ribosomal RNA gene and internal transcribed spacer 2, complete sequence; and 26S ribosomal RNA gene, partial sequence	780	780	99%	0.0	88%	
AY239994.1	Dendrobium serratilabium 18S ribosomal RNA gene, partial sequence; internal transcribed spacer 1, 5.8S ribosomal RNA gene and internal transcribed spacer 2, complete sequence; and 26S ribosomal RNA gene, partial sequence	780	780	99%	0.0	88%	
AY239958.1	Dendrobium ceraula 18S ribosomal RNA gene, partial sequence; internal transcribed spacer 1, 5.8S ribosomal RNA gene and internal transcribed spacer 2, complete sequence; and 26S ribosomal RNA gene, partial sequence	780	780	99%	0.0	88%	
AY239968.1	Dendrobium fulgidum 18S ribosomal RNA gene, partial sequence; internal transcribed spacer 1, 5.8S ribosomal RNA gene and internal transcribed spacer 2, complete sequence; and 26S ribosomal RNA gene, partial sequence	776	776	99%	0.0	88%	
AY240004.1	Dendrobium victoriae-reginae 18S ribosomal RNA gene, partial sequence; internal transcribed spacer 1, 5.8S ribosomal RNA gene and internal transcribed spacer 2, complete sequence; and 26S ribosomal RNA gene, partial sequence	774	774	99%	0.0	88%	
HM590385.1	Dendrobium chameleon voucher NCHU-D89331201-1019 18S ribosomal RNA gene, partial sequence; internal transcribed spacer 1, 5.8S ribosomal RNA gene, and internal transcribed spacer 2, complete sequence; and 26S ribosomal RNA gene, partial sequence	765	765	99%	0.0	87%	
HM590386.1	Dendrobium miyakei voucher NCHU-D89331201-1020 18S ribosomal RNA gene, partial sequence; internal transcribed spacer 1, 5.8S ribosomal RNA gene, and internal transcribed spacer 2, complete sequence; and 26S ribosomal RNA gene, partial sequence	754	754	99%	0.0	87%	
AY239970.1	Dendrobium goldschmidtianum 18S ribosomal RNA gene, partial sequence; internal transcribed spacer 1, 5.8S ribosomal RNA gene and internal transcribed spacer 2, complete sequence; and 26S ribosomal RNA gene, partial sequence	754	754	99%	0.0	87%	
AY239990.1	Dendrobium aff. rarum Clements 5613 18S ribosomal RNA gene, partial sequence; internal transcribed spacer 1, 5.8S ribosomal RNA gene and internal transcribed spacer 2, complete sequence; and 26S ribosomal RNA gene, partial sequence	741	741	99%	0.0	87%	
[illegible]	[illegible]	[illegible]	[illegible]	[illegible]	[illegible]	[illegible]	

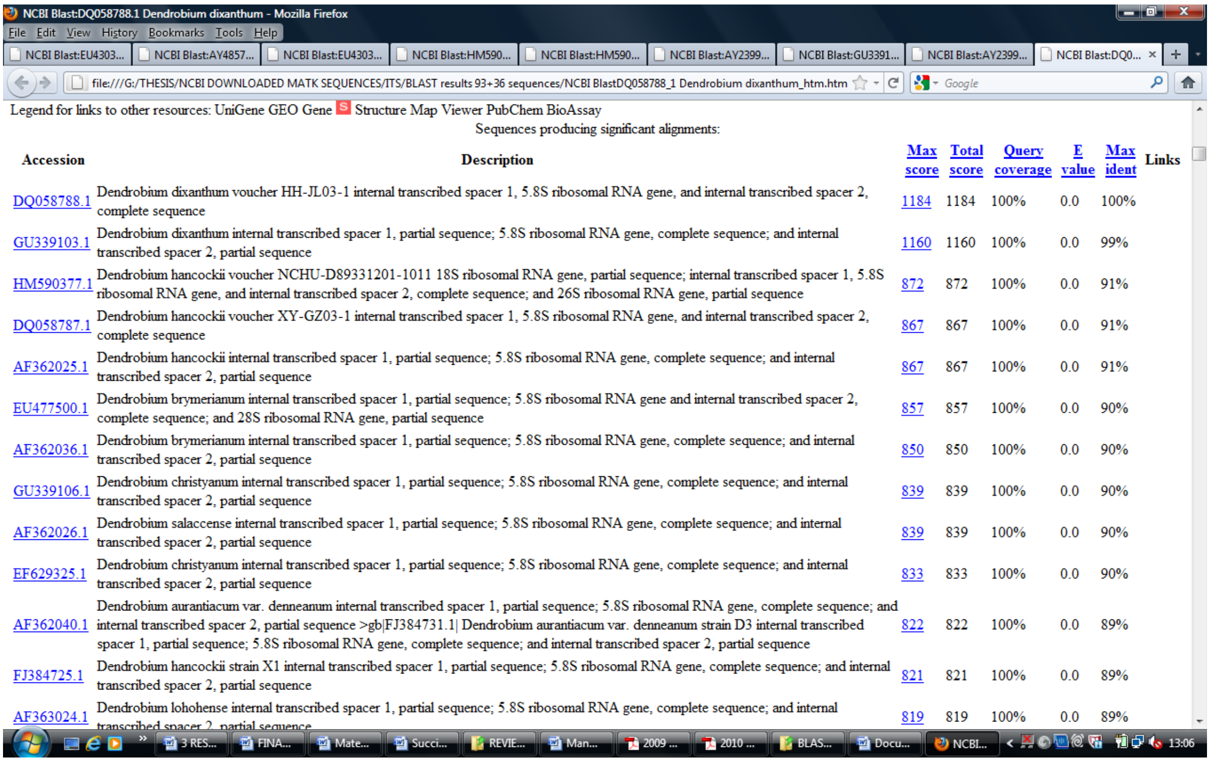
NCBI Blast:DQ058788.1 Dendrobium dixanthum - Mozilla Firefox

Legend for links to other resources: UniGene GEO Gene Structure Map Viewer PubChem BioAssay

Sequences producing significant alignments:

Accession	Description	Max score	Total score	Query coverage	E value	Max ident	Links
DQ058788.1	Dendrobium dixanthum voucher HH-JL03-1 internal transcribed spacer 1, 5.8S ribosomal RNA gene, and internal transcribed spacer 2, complete sequence	1184	1184	100%	0.0	100%	
GU339103.1	Dendrobium dixanthum internal transcribed spacer 1, partial sequence; 5.8S ribosomal RNA gene, complete sequence; and internal transcribed spacer 2, partial sequence	1160	1160	100%	0.0	99%	
HM590377.1	Dendrobium hancockii voucher NCHU-D89331201-1011 18S ribosomal RNA gene, partial sequence; internal transcribed spacer 1, 5.8S ribosomal RNA gene, and internal transcribed spacer 2, complete sequence; and 26S ribosomal RNA gene, partial sequence	872	872	100%	0.0	91%	
DQ058787.1	Dendrobium hancockii voucher XY-GZ03-1 internal transcribed spacer 1, 5.8S ribosomal RNA gene, and internal transcribed spacer 2, complete sequence	867	867	100%	0.0	91%	
AF362025.1	Dendrobium hancockii internal transcribed spacer 1, partial sequence; 5.8S ribosomal RNA gene, complete sequence; and internal transcribed spacer 2, partial sequence	867	867	100%	0.0	91%	
EU477500.1	Dendrobium brymerianum internal transcribed spacer 1, partial sequence; 5.8S ribosomal RNA gene and internal transcribed spacer 2, complete sequence; and 28S ribosomal RNA gene, partial sequence	857	857	100%	0.0	90%	
AF362036.1	Dendrobium brymerianum internal transcribed spacer 1, partial sequence; 5.8S ribosomal RNA gene, complete sequence; and internal transcribed spacer 2, partial sequence	850	850	100%	0.0	90%	
GU339106.1	Dendrobium christyanum internal transcribed spacer 1, partial sequence; 5.8S ribosomal RNA gene, complete sequence; and internal transcribed spacer 2, partial sequence	839	839	100%	0.0	90%	
AF362026.1	Dendrobium salaccense internal transcribed spacer 1, partial sequence; 5.8S ribosomal RNA gene, complete sequence; and internal transcribed spacer 2, partial sequence	839	839	100%	0.0	90%	
EF629325.1	Dendrobium christyanum internal transcribed spacer 1, partial sequence; 5.8S ribosomal RNA gene, complete sequence; and internal transcribed spacer 2, partial sequence	833	833	100%	0.0	90%	
AF362040.1	Dendrobium aurantiacum var. denneanum internal transcribed spacer 1, partial sequence; 5.8S ribosomal RNA gene, complete sequence; and internal transcribed spacer 2, partial sequence >gb|FJ384731.1| Dendrobium aurantiacum var. denneanum strain D3 internal transcribed spacer 1, partial sequence; 5.8S ribosomal RNA gene, complete sequence; and internal transcribed spacer 2, partial sequence	822	822	100%	0.0	89%	
FJ384725.1	Dendrobium hancockii strain X1 internal transcribed spacer 1, partial sequence; 5.8S ribosomal RNA gene, complete sequence; and internal transcribed spacer 2, partial sequence	821	821	100%	0.0	89%	
AF363024.1	Dendrobium lohohense internal transcribed spacer 1, partial sequence; 5.8S ribosomal RNA gene, complete sequence; and internal transcribed spacer 2, partial sequence	819	819	100%	0.0	89%	

NCBI Blast:AY239965.1 Dendrobium ellipsophyllum - Mozilla Firefox

file:///G:/THESIS/NCBI DOWNLOADED MATK SEQUENCES/ITS/BLAST results 93+36 sequences/NCBI BlastAY239965_1 Dendrobium ellipsophyllum_htm.h

Legend for links to other resources: UniGene GEO Gene S Structure Map Viewer PubChem BioAssay

Sequences producing significant alignments:

Accession	Description	Max score	Total score	Query coverage	E value	Max ident	Links
AY239965.1	Dendrobium ellipsophyllum 18S ribosomal RNA gene, partial sequence; internal transcribed spacer 1, 5.8S ribosomal RNA gene and internal transcribed spacer 2, complete sequence; and 26S ribosomal RNA gene, partial sequence	1230	1230	100%	0.0	100%	
AF362033.1	Dendrobium ellipsophyllum internal transcribed spacer 1, partial sequence; 5.8S ribosomal RNA gene, complete sequence; and internal transcribed spacer 2, partial sequence	1129	1129	96%	0.0	98%	
AY239967.1	Dendrobium formosum 18S ribosomal RNA gene, partial sequence; internal transcribed spacer 1, 5.8S ribosomal RNA gene and internal transcribed spacer 2, complete sequence; and 26S ribosomal RNA gene, partial sequence	843	843	100%	0.0	89%	
GU339106.1	Dendrobium christyanum internal transcribed spacer 1, partial sequence; 5.8S ribosomal RNA gene, complete sequence; and internal transcribed spacer 2, partial sequence	835	835	96%	0.0	90%	
EF629325.1	Dendrobium christyanum internal transcribed spacer 1, partial sequence; 5.8S ribosomal RNA gene, complete sequence; and internal transcribed spacer 2, partial sequence	828	828	96%	0.0	90%	
EU592015.1	Dendrobium bellatulum internal transcribed spacer 1, partial sequence; 5.8S ribosomal RNA gene, complete sequence; and internal transcribed spacer 2, partial sequence	819	819	96%	0.0	89%	
AY240001.1	Dendrobium thyrsiflorum 18S ribosomal RNA gene, partial sequence; internal transcribed spacer 1, 5.8S ribosomal RNA gene and internal transcribed spacer 2, complete sequence; and 26S ribosomal RNA gene, partial sequence	819	819	100%	0.0	88%	
AF362027.1	Dendrobium cariniferum internal transcribed spacer 1, partial sequence; 5.8S ribosomal RNA gene, complete sequence; and internal transcribed spacer 2, partial sequence	815	815	96%	0.0	89%	
AF362030.1	Dendrobium williamsonii internal transcribed spacer 1, partial sequence; 5.8S ribosomal RNA gene, complete sequence; and internal transcribed spacer 2, partial sequence	815	815	96%	0.0	89%	
HM054758.1	Dendrobium thyrsiflorum voucher SBB-0518 internal transcribed spacer 1, partial sequence; 5.8S ribosomal RNA gene and internal transcribed spacer 2, complete sequence; and 26S ribosomal RNA gene, partial sequence	809	809	98%	0.0	88%	
HM054668.1	Dendrobium infundibulum voucher SBB-0529 internal transcribed spacer 1, partial sequence; 5.8S ribosomal RNA gene and internal transcribed spacer 2, complete sequence; and 26S ribosomal RNA gene, partial sequence	809	809	94%	0.0	89%	
HM054669.1	Dendrobium infundibulum voucher SBB-0530 internal transcribed spacer 1, partial sequence; 5.8S ribosomal RNA gene and internal transcribed spacer 2, complete sequence; and 26S ribosomal RNA gene, partial sequence	797	797	93%	0.0	89%	
FJ428220.1	Dendrobium williamsonii internal transcribed spacer 1, partial sequence; 5.8S ribosomal RNA gene, complete sequence; and internal transcribed spacer 2, partial sequence	791	791	96%	0.0	88%	

NCBI Blast:AY485696.1 Dendrobium epidendropsis - Mozilla Firefox

file:///G:/THESIS/NCBI DOWNLOADED MATK SEQUENCES/ITS/BLAST results 93+36 sequences/NCBI BlastAY485696_1 Dendrobium epidendropsis_htm.h

Legend for links to other resources: UniGene GEO Gene S Structure Map Viewer PubChem BioAssay

Sequences producing significant alignments:

Accession	Description	Max score	Total score	Query coverage	E value	Max ident	Links
AY485696.1	Dendrobium epidendropsis specimen-voucher ICMdepi2204-1 internal transcribed spacer 1, partial sequence; 5.8S ribosomal RNA gene, complete sequence; and internal transcribed spacer 2, partial sequence	1175	1175	100%	0.0	100%	
AY485695.1	Dendrobium moschatum specimen-voucher ICMdmos2103-2 internal transcribed spacer 1, partial sequence; 5.8S ribosomal RNA gene, complete sequence; and internal transcribed spacer 2, partial sequence	828	828	100%	0.0	90%	
AY485711.1	Dendrobium gratiosissimum specimen-voucher ICMdgra2201-1 internal transcribed spacer 1, partial sequence; 5.8S ribosomal RNA gene, complete sequence; and internal transcribed spacer 2, partial sequence	737	737	100%	0.0	87%	
AY485694.1	Dendrobium hercoglossum specimen-voucher ICMdher2103-1 internal transcribed spacer 1, partial sequence; 5.8S ribosomal RNA gene, complete sequence; and internal transcribed spacer 2, partial sequence	737	737	100%	0.0	87%	
AY485708.1	Dendrobium falconeri specimen-voucher XYD0006-32 internal transcribed spacer 1, partial sequence; 5.8S ribosomal RNA gene, complete sequence; and internal transcribed spacer 2, partial sequence	732	732	100%	0.0	87%	
AY485714.1	Dendrobium fimbriatum specimen-voucher XYD0006-02 internal transcribed spacer 1, partial sequence; 5.8S ribosomal RNA gene, complete sequence; and internal transcribed spacer 2, partial sequence	702	702	100%	0.0	86%	
AY485718.1	Dendrobium moniliforme specimen-voucher XYD0206-43 internal transcribed spacer 1, partial sequence; 5.8S ribosomal RNA gene, complete sequence; and internal transcribed spacer 2, partial sequence	701	701	100%	0.0	86%	
AY485692.1	Dendrobium officinale specimen-voucher ICMdoff2103-7 internal transcribed spacer 1, partial sequence; 5.8S ribosomal RNA gene, complete sequence; and internal transcribed spacer 2, partial sequence	686	686	100%	0.0	86%	
AF363024.1	Dendrobium lohohense internal transcribed spacer 1, partial sequence; 5.8S ribosomal RNA gene, complete sequence; and internal transcribed spacer 2, partial sequence	686	686	100%	0.0	86%	
AY485712.1	Dendrobium pendulum specimen-voucher ICMdpen2103-1 internal transcribed spacer 1, partial sequence; 5.8S ribosomal RNA gene, complete sequence; and internal transcribed spacer 2, partial sequence	682	682	100%	0.0	86%	
AF359254.1	Dendrobium moniliforme specimen-voucher XYD00035 internal transcribed spacer 1, partial sequence; 5.8S ribosomal RNA gene, complete sequence; and internal transcribed spacer 2, partial sequence	676	676	100%	0.0	86%	
AY485715.1	Dendrobium primulinum specimen-voucher ICMdpri2103-1 internal transcribed spacer 1, partial sequence; 5.8S ribosomal RNA gene, complete sequence; and internal transcribed spacer 2, partial sequence	675	675	100%	0.0	85%	
AF311780.1	Dendrobium gratiosissimum internal transcribed spacer 1, partial sequence; 5.8S ribosomal RNA gene, complete sequence; and internal transcribed spacer 2, partial sequence	671	671	99%	0.0	86%	

NCBI Blast:HM590388.1 Dendrobium equitans - Mozilla Firefox

file:///G:/THESIS/NCBI DOWNLOADED MATK SEQUENCES/ITS/BLAST results 93+36 sequences/NCBI BlastHM590388_1 Dendrobium equitans_htm.htm

Legend for links to other resources: UniGene GEO Gene Structure Map Viewer PubChem BioAssay

Sequences producing significant alignments:

Accession	Description	Max score	Total score	Query coverage	E value	Max ident	Links
HM590388.1	Dendrobium equitans voucher NCHU-D89331201-1023 18S ribosomal RNA gene, partial sequence; internal transcribed spacer 1, 5.8S ribosomal RNA gene, and internal transcribed spacer 2, complete sequence; and 26S ribosomal RNA gene, partial sequence	1642	1642	100%	0.0	100%	
HM590370.1	Dendrobium crumenatum voucher NCHU-D89331201-1004 18S ribosomal RNA gene, partial sequence; internal transcribed spacer 1, 5.8S ribosomal RNA gene, and internal transcribed spacer 2, complete sequence; and 26S ribosomal RNA gene, partial sequence	1085	1085	100%	0.0	88%	
AF521609.1	Dendrobium equitans internal transcribed spacer 1, 5.8S ribosomal RNA gene, and internal transcribed spacer 2, complete sequence >gb|EU840701.1| Dendrobium equitans internal transcribed spacer 1, 5.8S ribosomal RNA gene, and internal transcribed spacer 2, complete sequence	1002	1002	72%	0.0	94%	
HM590385.1	Dendrobium chameleon voucher NCHU-D89331201-1019 18S ribosomal RNA gene, partial sequence; internal transcribed spacer 1, 5.8S ribosomal RNA gene, and internal transcribed spacer 2, complete sequence; and 26S ribosomal RNA gene, partial sequence	891	891	100%	0.0	85%	
HM590386.1	Dendrobium miyakei voucher NCHU-D89331201-1020 18S ribosomal RNA gene, partial sequence; internal transcribed spacer 1, 5.8S ribosomal RNA gene, and internal transcribed spacer 2, complete sequence; and 26S ribosomal RNA gene, partial sequence	843	843	89%	0.0	86%	
AY239975.1	Dendrobium junceum 18S ribosomal RNA gene, partial sequence; internal transcribed spacer 1, 5.8S ribosomal RNA gene and internal transcribed spacer 2, complete sequence; and 26S ribosomal RNA gene, partial sequence	843	843	74%	0.0	89%	
HM590377.1	Dendrobium hancockii voucher NCHU-D89331201-1011 18S ribosomal RNA gene, partial sequence; internal transcribed spacer 1, 5.8S ribosomal RNA gene, and internal transcribed spacer 2, complete sequence; and 26S ribosomal RNA gene, partial sequence	837	837	81%	0.0	87%	
HM590383.1	Dendrobium chrysotoxum voucher NCHU-D89331201-1017 18S ribosomal RNA gene, partial sequence; internal transcribed spacer 1, 5.8S ribosomal RNA gene, and internal transcribed spacer 2, complete sequence; and 26S ribosomal RNA gene, partial sequence	822	822	81%	0.0	87%	
HM590392.1	Dendrobium fimbriatum voucher NCHU-D89331201-1029 18S ribosomal RNA gene, partial sequence; internal transcribed spacer 1, 5.8S ribosomal RNA gene, and internal transcribed spacer 2, complete sequence; and 26S ribosomal RNA gene, partial sequence	819	819	81%	0.0	87%	
HM590375.1	Dendrobium aurantiacum voucher NCHU-D89331201-1009 18S ribosomal RNA gene, partial sequence; internal transcribed spacer 1, 5.8S ribosomal RNA gene, and internal transcribed spacer 2, complete sequence; and 26S ribosomal RNA gene, partial sequence	813	813	81%	0.0	87%	
AY273708.1	Dendrobium crumenatum isolate 105 internal transcribed spacer 1, 5.8S ribosomal RNA gene, and internal transcribed spacer 2, complete sequence	806	806	81%	0.0	87%	
AY240003.1	Dendrobium usterioides 18S ribosomal RNA gene, partial sequence; internal transcribed spacer 1, 5.8S ribosomal RNA gene and internal transcribed spacer 2, complete sequence; and 26S ribosomal RNA gene, partial sequence	800	800	74%	0.0	88%	
AY239989.1	Dendrobium quadrangulare 18S ribosomal RNA gene, partial sequence; internal transcribed spacer 1, 5.8S ribosomal RNA gene and internal transcribed spacer 2, complete sequence; and 26S ribosomal RNA gene, partial sequence	798	798	74%	0.0	88%	

NCBI Blast:AY485706.1 Dendrobium erosum - Mozilla Firefox

file:///G:/THESIS/NCBI DOWNLOADED MATK SEQUENCES/ITS/BLAST results 93+36 sequences/NCBI BlastAY485706_1 Dendrobium erosum_htm.htm

Legend for links to other resources: UniGene GEO Gene Structure Map Viewer PubChem BioAssay

Sequences producing significant alignments:

Accession	Description	Max score	Total score	Query coverage	E value	Max ident	Links
AY485706.1	Dendrobium erosum specimen-voucher ICMdero2201-1 internal transcribed spacer 1, partial sequence; 5.8S ribosomal RNA gene, complete sequence; and internal transcribed spacer 2, partial sequence	1181	1181	100%	0.0	100%	
AY485692.1	Dendrobium officinale specimen-voucher ICMdoff2103-7 internal transcribed spacer 1, partial sequence; 5.8S ribosomal RNA gene, complete sequence; and internal transcribed spacer 2, partial sequence	797	797	99%	0.0	89%	
AY485694.1	Dendrobium hercoglossum specimen-voucher ICMdher2103-1 internal transcribed spacer 1, partial sequence; 5.8S ribosomal RNA gene, complete sequence; and internal transcribed spacer 2, partial sequence	774	774	99%	0.0	88%	
AY485707.1	Dendrobium bellatulum specimen-voucher ICMdbel2103-1 internal transcribed spacer 1, partial sequence; 5.8S ribosomal RNA gene, complete sequence; and internal transcribed spacer 2, partial sequence	749	749	99%	0.0	88%	
FJ384743.1	Dendrobium flexicaule internal transcribed spacer 1, partial sequence; 5.8S ribosomal RNA gene, complete sequence; and internal transcribed spacer 2, partial sequence	747	747	99%	0.0	87%	
AY485703.1	Dendrobium loddigesii specimen-voucher XYD0006-12 internal transcribed spacer 1, partial sequence; 5.8S ribosomal RNA gene, complete sequence; and internal transcribed spacer 2, partial sequence	747	747	99%	0.0	87%	
AY485708.1	Dendrobium falconeri specimen-voucher XYD0006-32 internal transcribed spacer 1, partial sequence; 5.8S ribosomal RNA gene, complete sequence; and internal transcribed spacer 2, partial sequence	737	737	99%	0.0	87%	
HM590391.1	Dendrobium candidum voucher NCHU-D89331201-1028 18S ribosomal RNA gene, partial sequence; internal transcribed spacer 1, 5.8S ribosomal RNA gene, and internal transcribed spacer 2, complete sequence; and 26S ribosomal RNA gene, partial sequence	736	736	99%	0.0	87%	
FJ384723.1	Dendrobium officinale strain T1 internal transcribed spacer 1, partial sequence; 5.8S ribosomal RNA gene, complete sequence; and internal transcribed spacer 2, partial sequence	736	736	99%	0.0	87%	
FJ384724.1	Dendrobium officinale strain T2 internal transcribed spacer 1, partial sequence; 5.8S ribosomal RNA gene, complete sequence; and internal transcribed spacer 2, partial sequence >gb|FJ530944.1| Dendrobium officinale strain T4 internal transcribed spacer 1, partial sequence; 5.8S ribosomal RNA gene, complete sequence; and internal transcribed spacer 2, partial sequence >gb|FJ530945.1| Dendrobium officinale strain T5 internal transcribed spacer 1, partial sequence; 5.8S ribosomal RNA gene, complete sequence; and internal transcribed spacer 2, partial sequence >gb|FJ530946.1| Dendrobium officinale strain T6 internal transcribed spacer 1, partial sequence; 5.8S ribosomal RNA gene, complete sequence; and internal transcribed spacer 2, partial sequence >gb|FJ530947.1| Dendrobium officinale strain T7 internal transcribed spacer 1, partial sequence; 5.8S ribosomal RNA gene, complete sequence; and internal transcribed spacer 2, partial sequence >gb|FJ588871.1| Dendrobium officinale strain T3 internal transcribed spacer 1, partial sequence; 5.8S ribosomal RNA gene, complete sequence; and internal transcribed spacer 2, partial sequence >gb|FJ588872.1| Dendrobium officinale strain T8 internal transcribed spacer 1, partial sequence; 5.8S [illegible]	736	736	99%	0.0	87%	

NCBI Blast:AF362024.1 Dendrobium exile - Mozilla Firefox

file:///G:/THESIS/NCBI DOWNLOADED MATK SEQUENCES/ITS/BLAST results 93+36 sequences/NCBI BlastAF362024_1 Dendrobium exile_htm.htm

Legend for links to other resources: UniGene GEO Gene S Structure Map Viewer PubChem BioAssay

Sequences producing significant alignments:

Accession	Description	Max score	Total score	Query coverage	E value	Max ident	Links
AF362024.1	Dendrobium exile internal transcribed spacer 1, partial sequence; 5.8S ribosomal RNA gene, complete sequence; and internal transcribed spacer 2, partial sequence	1186	1186	100%	0.0	100%	
AY239961.1	Dendrobium confusum 18S ribosomal RNA gene, partial sequence; internal transcribed spacer 1, 5.8S ribosomal RNA gene and internal transcribed spacer 2, complete sequence; and 26S ribosomal RNA gene, partial sequence	843	843	100%	0.0	90%	
AY239951.1	Dendrobium aloifolium 18S ribosomal RNA gene, partial sequence; internal transcribed spacer 1, 5.8S ribosomal RNA gene and internal transcribed spacer 2, complete sequence; and 26S ribosomal RNA gene, partial sequence	817	817	99%	0.0	89%	
HM054548.1	Dendrobium anceps voucher SBB-0301 18S ribosomal RNA gene, partial sequence; internal transcribed spacer 1, 5.8S ribosomal RNA gene, and internal transcribed spacer 2, complete sequence; and 26S ribosomal RNA gene, partial sequence	804	804	99%	0.0	89%	
AY239988.1	Dendrobium philippinense 18S ribosomal RNA gene, partial sequence; internal transcribed spacer 1, 5.8S ribosomal RNA gene and internal transcribed spacer 2, complete sequence; and 26S ribosomal RNA gene, partial sequence	797	797	100%	0.0	89%	
AY239953.1	Dendrobium anceps 18S ribosomal RNA gene, partial sequence; internal transcribed spacer 1, 5.8S ribosomal RNA gene and internal transcribed spacer 2, complete sequence; and 26S ribosomal RNA gene, partial sequence	797	797	99%	0.0	89%	
AY239969.1	Dendrobium goldfinchii 18S ribosomal RNA gene, partial sequence; internal transcribed spacer 1, 5.8S ribosomal RNA gene and internal transcribed spacer 2, complete sequence; and 26S ribosomal RNA gene, partial sequence	789	789	100%	0.0	89%	
AY239972.1	Dendrobium indivisum 18S ribosomal RNA gene, partial sequence; internal transcribed spacer 1, 5.8S ribosomal RNA gene and internal transcribed spacer 2, complete sequence; and 26S ribosomal RNA gene, partial sequence	787	787	100%	0.0	88%	
AY239975.1	Dendrobium junceum 18S ribosomal RNA gene, partial sequence; internal transcribed spacer 1, 5.8S ribosomal RNA gene and internal transcribed spacer 2, complete sequence; and 26S ribosomal RNA gene, partial sequence	782	782	100%	0.0	88%	
HM054547.1	Dendrobium anceps voucher SBB-0119 internal transcribed spacer 1, partial sequence; 5.8S ribosomal RNA gene, complete sequence; and internal transcribed spacer 2, partial sequence	780	780	96%	0.0	89%	
AY239978.1	Dendrobium leonis 18S ribosomal RNA gene, partial sequence; internal transcribed spacer 1, 5.8S ribosomal RNA gene and internal transcribed spacer 2, complete sequence; and 26S ribosomal RNA gene, partial sequence	773	773	100%	0.0	88%	
AF521609.1	Dendrobium equitans internal transcribed spacer 1, 5.8S ribosomal RNA gene, and internal transcribed spacer 2, complete sequence >gb|EU840701.1| Dendrobium equitans internal transcribed spacer 1, 5.8S ribosomal RNA gene, and internal transcribed spacer 2, complete sequence	771	771	100%	0.0	88%	
AF362034.1	Dendrobium acinaciforme internal transcribed spacer 1, partial sequence; 5.8S ribosomal RNA gene, complete sequence; and internal transcribed spacer 2, partial sequence >gb|EF629321.1| Dendrobium jenkinsii internal transcribed spacer 1, partial sequence; 5.8S [illegible]	763	763	100%	0.0	88%	

NCBI Blast:AY239966.1 Dendrobium fairchildae - Mozilla Firefox

file:///G:/THESIS/NCBI DOWNLOADED MATK SEQUENCES/ITS/BLAST results 93+36 sequences/NCBI BlastAY239966_1 Dendrobium fairchildae_htm.htm

Legend for links to other resources: UniGene GEO Gene S Structure Map Viewer PubChem BioAssay

Sequences producing significant alignments:

Accession	Description	Max score	Total score	Query coverage	E value	Max ident	Links
AY239966.1	Dendrobium fairchildae 18S ribosomal RNA gene, partial sequence; internal transcribed spacer 1, 5.8S ribosomal RNA gene and internal transcribed spacer 2, complete sequence; and 26S ribosomal RNA gene, partial sequence	1227	1227	100%	0.0	100%	
AY239994.1	Dendrobium serratilabium 18S ribosomal RNA gene, partial sequence; internal transcribed spacer 1, 5.8S ribosomal RNA gene and internal transcribed spacer 2, complete sequence; and 26S ribosomal RNA gene, partial sequence	937	937	100%	0.0	92%	
HM590385.1	Dendrobium chameleon voucher NCHU-D89331201-1019 18S ribosomal RNA gene, partial sequence; internal transcribed spacer 1, 5.8S ribosomal RNA gene, and internal transcribed spacer 2, complete sequence; and 26S ribosomal RNA gene, partial sequence	922	922	100%	0.0	91%	
AY239987.1	Dendrobium papilio 18S ribosomal RNA gene, partial sequence; internal transcribed spacer 1, 5.8S ribosomal RNA gene and internal transcribed spacer 2, complete sequence; and 26S ribosomal RNA gene, partial sequence	922	922	100%	0.0	91%	
AY239960.1	Dendrobium chameleon 18S ribosomal RNA gene, partial sequence; internal transcribed spacer 1, 5.8S ribosomal RNA gene and internal transcribed spacer 2, complete sequence; and 26S ribosomal RNA gene, partial sequence	920	920	100%	0.0	91%	
AY240006.1	Dendrobium yeageri 18S ribosomal RNA gene, partial sequence; internal transcribed spacer 1, 5.8S ribosomal RNA gene and internal transcribed spacer 2, complete sequence; and 26S ribosomal RNA gene, partial sequence	915	915	100%	0.0	91%	
AY239958.1	Dendrobium ceraula 18S ribosomal RNA gene, partial sequence; internal transcribed spacer 1, 5.8S ribosomal RNA gene and internal transcribed spacer 2, complete sequence; and 26S ribosomal RNA gene, partial sequence	915	915	100%	0.0	91%	
AY240004.1	Dendrobium victoriae-reginae 18S ribosomal RNA gene, partial sequence; internal transcribed spacer 1, 5.8S ribosomal RNA gene and internal transcribed spacer 2, complete sequence; and 26S ribosomal RNA gene, partial sequence	909	909	100%	0.0	91%	
AY239976.1	Dendrobium lancifolium 18S ribosomal RNA gene, partial sequence; internal transcribed spacer 1, 5.8S ribosomal RNA gene and internal transcribed spacer 2, complete sequence; and 26S ribosomal RNA gene, partial sequence	887	887	100%	0.0	90%	
HM590386.1	Dendrobium miyakei voucher NCHU-D89331201-1020 18S ribosomal RNA gene, partial sequence; internal transcribed spacer 1, 5.8S ribosomal RNA gene, and internal transcribed spacer 2, complete sequence; and 26S ribosomal RNA gene, partial sequence	878	878	100%	0.0	90%	
AY239970.1	Dendrobium goldschmidtianum 18S ribosomal RNA gene, partial sequence; internal transcribed spacer 1, 5.8S ribosomal RNA gene and internal transcribed spacer 2, complete sequence; and 26S ribosomal RNA gene, partial sequence	878	878	100%	0.0	90%	
AF521607.1	Dendrobium chameleon internal transcribed spacer 1, 5.8S ribosomal RNA gene, and internal transcribed spacer 2, complete sequence	867	867	96%	0.0	91%	
AY239968.1	Dendrobium fulgidum 18S ribosomal RNA gene, partial sequence; internal transcribed spacer 1, 5.8S ribosomal RNA gene and internal transcribed spacer 2, complete sequence; and 26S ribosomal RNA gene, partial sequence	856	856	100%	0.0	90%	
EU840695.1	Dendrobium goldschmidtianum internal transcribed spacer 1, 5.8S ribosomal RNA gene, and internal transcribed spacer 2, complete sequence	839	839	96%	0.0	90%	

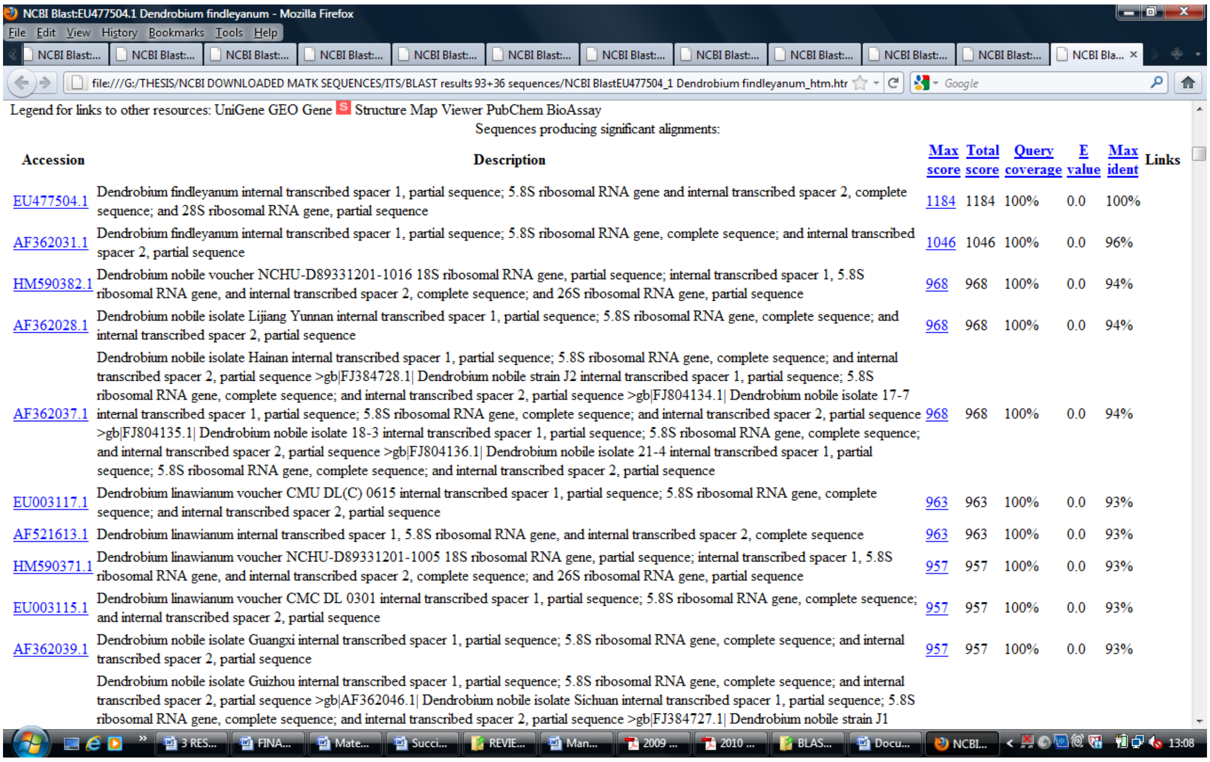

Legend for links to other resources: UniGene GEO Gene Structure Map Viewer PubChem BioAssay

Sequences producing significant alignments:

Accession	Description	Max score	Total score	Query coverage	E value	Max ident	Links
EU477504.1	Dendrobium findleyanum internal transcribed spacer 1, partial sequence; 5.8S ribosomal RNA gene and internal transcribed spacer 2, complete sequence; and 28S ribosomal RNA gene, partial sequence	1184	1184	100%	0.0	100%	
AF362031.1	Dendrobium findleyanum internal transcribed spacer 1, partial sequence; 5.8S ribosomal RNA gene, complete sequence; and internal transcribed spacer 2, partial sequence	1046	1046	100%	0.0	96%	
HM590382.1	Dendrobium nobile voucher NCHU-D89331201-1016 18S ribosomal RNA gene, partial sequence; internal transcribed spacer 1, 5.8S ribosomal RNA gene, and internal transcribed spacer 2, complete sequence; and 26S ribosomal RNA gene, partial sequence	968	968	100%	0.0	94%	
AF362028.1	Dendrobium nobile isolate Lijiang Yunnan internal transcribed spacer 1, partial sequence; 5.8S ribosomal RNA gene, complete sequence; and internal transcribed spacer 2, partial sequence	968	968	100%	0.0	94%	
AF362037.1	Dendrobium nobile isolate Hainan internal transcribed spacer 1, partial sequence; 5.8S ribosomal RNA gene, complete sequence; and internal transcribed spacer 2, partial sequence >gb|FJ384728.1| Dendrobium nobile strain J2 internal transcribed spacer 1, partial sequence; 5.8S ribosomal RNA gene, complete sequence; and internal transcribed spacer 2, partial sequence >gb|FJ804134.1| Dendrobium nobile isolate 17-7 internal transcribed spacer 1, partial sequence; 5.8S ribosomal RNA gene, complete sequence; and internal transcribed spacer 2, partial sequence >gb|FJ804135.1| Dendrobium nobile isolate 18-3 internal transcribed spacer 1, partial sequence; 5.8S ribosomal RNA gene, complete sequence; and internal transcribed spacer 2, partial sequence >gb|FJ804136.1| Dendrobium nobile isolate 21-4 internal transcribed spacer 1, partial sequence; 5.8S ribosomal RNA gene, complete sequence; and internal transcribed spacer 2, partial sequence	968	968	100%	0.0	94%	
EU003117.1	Dendrobium linawianum voucher CMU DL(C) 0615 internal transcribed spacer 1, partial sequence; 5.8S ribosomal RNA gene, complete sequence; and internal transcribed spacer 2, partial sequence	963	963	100%	0.0	93%	
AF521613.1	Dendrobium linawianum internal transcribed spacer 1, 5.8S ribosomal RNA gene, and internal transcribed spacer 2, complete sequence	963	963	100%	0.0	93%	
HM590371.1	Dendrobium linawianum voucher NCHU-D89331201-1005 18S ribosomal RNA gene, partial sequence; internal transcribed spacer 1, 5.8S ribosomal RNA gene, and internal transcribed spacer 2, complete sequence; and 26S ribosomal RNA gene, partial sequence	957	957	100%	0.0	93%	
EU003115.1	Dendrobium linawianum voucher CMC DL 0301 internal transcribed spacer 1, partial sequence; 5.8S ribosomal RNA gene, complete sequence; and internal transcribed spacer 2, partial sequence	957	957	100%	0.0	93%	
AF362039.1	Dendrobium nobile isolate Guangxi internal transcribed spacer 1, partial sequence; 5.8S ribosomal RNA gene, complete sequence; and internal transcribed spacer 2, partial sequence	957	957	100%	0.0	93%	
	Dendrobium nobile isolate Guizhou internal transcribed spacer 1, partial sequence; 5.8S ribosomal RNA gene, complete sequence; and internal transcribed spacer 2, partial sequence >gb|AF362046.1| Dendrobium nobile isolate Sichuan internal transcribed spacer 1, partial sequence; 5.8S ribosomal RNA gene, complete sequence; and internal transcribed spacer 2, partial sequence >gb|FJ384727.1| Dendrobium nobile strain J1						

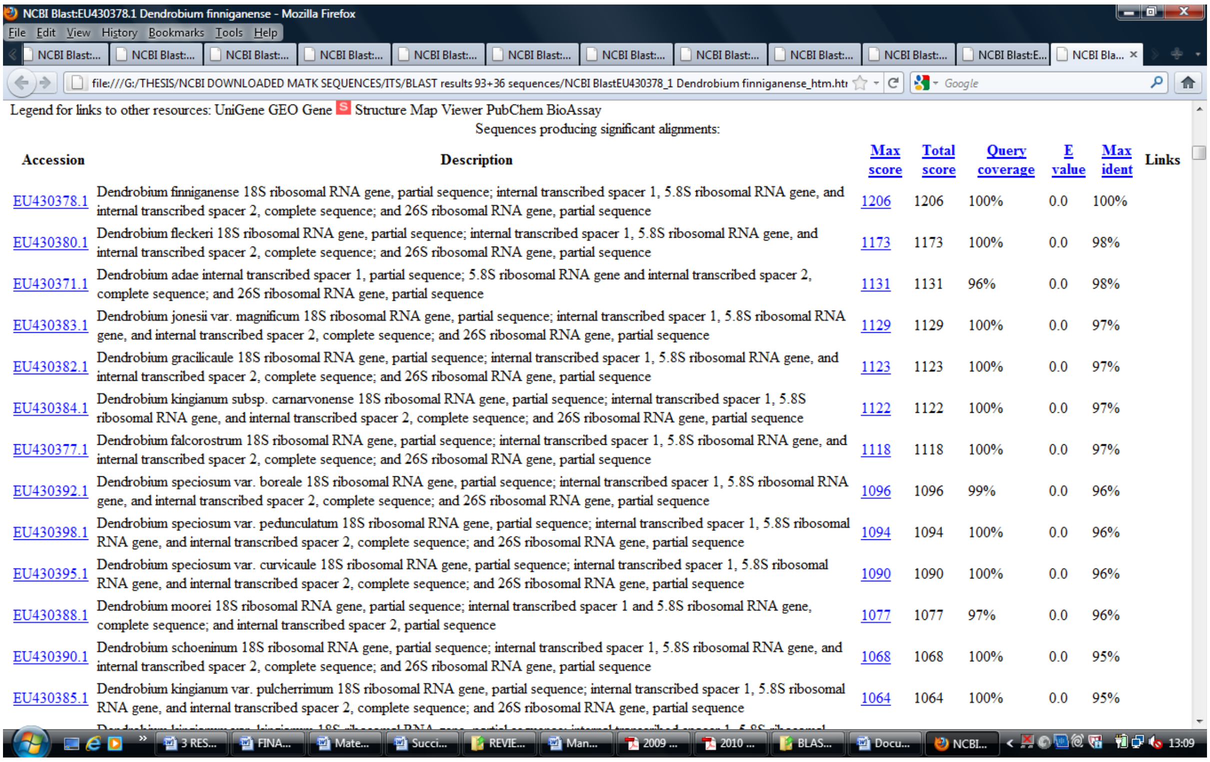

Legend for links to other resources: UniGene GEO Gene Structure Map Viewer PubChem BioAssay

Sequences producing significant alignments:

Accession	Description	Max score	Total score	Query coverage	E value	Max ident	Links
EU430378.1	Dendrobium finniganense 18S ribosomal RNA gene, partial sequence; internal transcribed spacer 1, 5.8S ribosomal RNA gene, and internal transcribed spacer 2, complete sequence; and 26S ribosomal RNA gene, partial sequence	1206	1206	100%	0.0	100%	
EU430380.1	Dendrobium fleckeri 18S ribosomal RNA gene, partial sequence; internal transcribed spacer 1, 5.8S ribosomal RNA gene, and internal transcribed spacer 2, complete sequence; and 26S ribosomal RNA gene, partial sequence	1173	1173	100%	0.0	98%	
EU430371.1	Dendrobium adae internal transcribed spacer 1, partial sequence; 5.8S ribosomal RNA gene and internal transcribed spacer 2, complete sequence; and 26S ribosomal RNA gene, partial sequence	1131	1131	96%	0.0	98%	
EU430383.1	Dendrobium jonesii var. magnificum 18S ribosomal RNA gene, partial sequence; internal transcribed spacer 1, 5.8S ribosomal RNA gene, and internal transcribed spacer 2, complete sequence; and 26S ribosomal RNA gene, partial sequence	1129	1129	100%	0.0	97%	
EU430382.1	Dendrobium gracilicaule 18S ribosomal RNA gene, partial sequence; internal transcribed spacer 1, 5.8S ribosomal RNA gene, and internal transcribed spacer 2, complete sequence; and 26S ribosomal RNA gene, partial sequence	1123	1123	100%	0.0	97%	
EU430384.1	Dendrobium kingianum subsp. carnarvonense 18S ribosomal RNA gene, partial sequence; internal transcribed spacer 1, 5.8S ribosomal RNA gene, and internal transcribed spacer 2, complete sequence; and 26S ribosomal RNA gene, partial sequence	1122	1122	100%	0.0	97%	
EU430377.1	Dendrobium falcorostrum 18S ribosomal RNA gene, partial sequence; internal transcribed spacer 1, 5.8S ribosomal RNA gene, and internal transcribed spacer 2, complete sequence; and 26S ribosomal RNA gene, partial sequence	1118	1118	100%	0.0	97%	
EU430392.1	Dendrobium speciosum var. boreale 18S ribosomal RNA gene, partial sequence; internal transcribed spacer 1, 5.8S ribosomal RNA gene, and internal transcribed spacer 2, complete sequence; and 26S ribosomal RNA gene, partial sequence	1096	1096	99%	0.0	96%	
EU430398.1	Dendrobium speciosum var. pedunculatum 18S ribosomal RNA gene, partial sequence; internal transcribed spacer 1, 5.8S ribosomal RNA gene, and internal transcribed spacer 2, complete sequence; and 26S ribosomal RNA gene, partial sequence	1094	1094	100%	0.0	96%	
EU430395.1	Dendrobium speciosum var. curvicaule 18S ribosomal RNA gene, partial sequence; internal transcribed spacer 1, 5.8S ribosomal RNA gene, and internal transcribed spacer 2, complete sequence; and 26S ribosomal RNA gene, partial sequence	1090	1090	100%	0.0	96%	
EU430388.1	Dendrobium moorei 18S ribosomal RNA gene, partial sequence; internal transcribed spacer 1 and 5.8S ribosomal RNA gene, complete sequence; and internal transcribed spacer 2, partial sequence	1077	1077	97%	0.0	96%	
EU430390.1	Dendrobium schoeninum 18S ribosomal RNA gene, partial sequence; internal transcribed spacer 1, 5.8S ribosomal RNA gene, and internal transcribed spacer 2, complete sequence; and 26S ribosomal RNA gene, partial sequence	1068	1068	100%	0.0	95%	
EU430385.1	Dendrobium kingianum var. pulcherrimum 18S ribosomal RNA gene, partial sequence; internal transcribed spacer 1, 5.8S ribosomal RNA gene, and internal transcribed spacer 2, complete sequence; and 26S ribosomal RNA gene, partial sequence	1064	1064	100%	0.0	95%	

NCBI Blast:AY239967.1 Dendrobium formosum - Mozilla Firefox

Legend for links to other resources: UniGene GEO Gene Structure Map Viewer PubChem BioAssay

Sequences producing significant alignments:

Accession	Description	Max score	Total score	Query coverage	E value	Max ident	Links
AY239967.1	Dendrobium formosum 18S ribosomal RNA gene, partial sequence; internal transcribed spacer 1, 5.8S ribosomal RNA gene and internal transcribed spacer 2, complete sequence; and 26S ribosomal RNA gene, partial sequence	1219	1219	100%	0.0	100%	
AF362027.1	Dendrobium cariniferum internal transcribed spacer 1, partial sequence; 5.8S ribosomal RNA gene, complete sequence; and internal transcribed spacer 2, partial sequence	1120	1120	96%	0.0	98%	
AF362030.1	Dendrobium williamsonii internal transcribed spacer 1, partial sequence; 5.8S ribosomal RNA gene, complete sequence; and internal transcribed spacer 2, partial sequence	1120	1120	96%	0.0	98%	
GU339106.1	Dendrobium christyanum internal transcribed spacer 1, partial sequence; 5.8S ribosomal RNA gene, complete sequence; and internal transcribed spacer 2, partial sequence	1051	1051	96%	0.0	96%	
EF629325.1	Dendrobium christyanum internal transcribed spacer 1, partial sequence; 5.8S ribosomal RNA gene, complete sequence; and internal transcribed spacer 2, partial sequence	1050	1050	96%	0.0	96%	
EU592015.1	Dendrobium bellatulum internal transcribed spacer 1, partial sequence; 5.8S ribosomal RNA gene, complete sequence; and internal transcribed spacer 2, partial sequence	1024	1024	96%	0.0	95%	
HM054668.1	Dendrobium infundibulum voucher SBB-0529 internal transcribed spacer 1, partial sequence; 5.8S ribosomal RNA gene and internal transcribed spacer 2, complete sequence; and 26S ribosomal RNA gene, partial sequence	1020	1020	94%	0.0	96%	
FJ428220.1	Dendrobium williamsonii internal transcribed spacer 1, partial sequence; 5.8S ribosomal RNA gene, complete sequence; and internal transcribed spacer 2, partial sequence	1018	1018	96%	0.0	95%	
GU339112.1	Dendrobium longicornu internal transcribed spacer 1, partial sequence; 5.8S ribosomal RNA gene, complete sequence; and internal transcribed spacer 2, partial sequence	1013	1013	96%	0.0	95%	
HM054669.1	Dendrobium infundibulum voucher SBB-0530 internal transcribed spacer 1, partial sequence; 5.8S ribosomal RNA gene and internal transcribed spacer 2, complete sequence; and 26S ribosomal RNA gene, partial sequence	1007	1007	93%	0.0	95%	
DQ058796.1	Dendrobium longicornu voucher CJ-SC02-1 internal transcribed spacer 1, 5.8S ribosomal RNA gene, and internal transcribed spacer 2, complete sequence	1007	1007	96%	0.0	95%	
HM054628.1	Dendrobium draconis voucher SBB-0546 18S ribosomal RNA gene, partial sequence; internal transcribed spacer 1, 5.8S ribosomal RNA gene, and internal transcribed spacer 2, complete sequence; and 26S ribosomal RNA gene, partial sequence	994	994	99%	0.0	93%	
EF641112.1	Dendrobium chapaense internal transcribed spacer 1, 5.8S ribosomal RNA gene, and internal transcribed spacer 2, complete sequence	985	985	96%	0.0	94%	
GU339102.1	Dendrobium williamsonii internal transcribed spacer 1, partial sequence; 5.8S ribosomal RNA gene, complete sequence; and internal [illegible]	970	970	96%	0.0	94%	

NCBI Blast:AY239968.1 Dendrobium fulgidum - Mozilla Firefox

Legend for links to other resources: UniGene GEO Gene Structure Map Viewer PubChem BioAssay

Sequences producing significant alignments:

Accession	Description	Max score	Total score	Query coverage	E value	Max ident	Links
AY239968.1	Dendrobium fulgidum 18S ribosomal RNA gene, partial sequence; internal transcribed spacer 1, 5.8S ribosomal RNA gene and internal transcribed spacer 2, complete sequence; and 26S ribosomal RNA gene, partial sequence	1227	1227	100%	0.0	100%	
AY239980.1	Dendrobium mohlianum 18S ribosomal RNA gene, partial sequence; internal transcribed spacer 1, 5.8S ribosomal RNA gene and internal transcribed spacer 2, complete sequence; and 26S ribosomal RNA gene, partial sequence	965	965	100%	0.0	92%	
AY239986.1	Dendrobium nothofagicola 18S ribosomal RNA gene, partial sequence; internal transcribed spacer 1, 5.8S ribosomal RNA gene and internal transcribed spacer 2, complete sequence; and 26S ribosomal RNA gene, partial sequence	941	941	100%	0.0	92%	
AY239977.1	Dendrobium lawesii 18S ribosomal RNA gene, partial sequence; internal transcribed spacer 1, 5.8S ribosomal RNA gene and internal transcribed spacer 2, complete sequence; and 26S ribosomal RNA gene, partial sequence	920	920	100%	0.0	91%	
AY239976.1	Dendrobium lancifolium 18S ribosomal RNA gene, partial sequence; internal transcribed spacer 1, 5.8S ribosomal RNA gene and internal transcribed spacer 2, complete sequence; and 26S ribosomal RNA gene, partial sequence	920	920	100%	0.0	91%	
AY239954.1	Dendrobium bracteosum 18S ribosomal RNA gene, partial sequence; internal transcribed spacer 1, 5.8S ribosomal RNA gene and internal transcribed spacer 2, complete sequence; and 26S ribosomal RNA gene, partial sequence	917	917	100%	0.0	91%	
AY239994.1	Dendrobium serratilabium 18S ribosomal RNA gene, partial sequence; internal transcribed spacer 1, 5.8S ribosomal RNA gene and internal transcribed spacer 2, complete sequence; and 26S ribosomal RNA gene, partial sequence	909	909	100%	0.0	91%	
AY239960.1	Dendrobium chameleon 18S ribosomal RNA gene, partial sequence; internal transcribed spacer 1, 5.8S ribosomal RNA gene and internal transcribed spacer 2, complete sequence; and 26S ribosomal RNA gene, partial sequence	904	904	100%	0.0	91%	
HM590385.1	Dendrobium chameleon voucher NCHU-D89331201-1019 18S ribosomal RNA gene, partial sequence; internal transcribed spacer 1, 5.8S ribosomal RNA gene, and internal transcribed spacer 2, complete sequence; and 26S ribosomal RNA gene, partial sequence	900	900	100%	0.0	91%	
AY240006.1	Dendrobium yeageri 18S ribosomal RNA gene, partial sequence; internal transcribed spacer 1, 5.8S ribosomal RNA gene and internal transcribed spacer 2, complete sequence; and 26S ribosomal RNA gene, partial sequence	898	898	100%	0.0	91%	
AY239958.1	Dendrobium ceraula 18S ribosomal RNA gene, partial sequence; internal transcribed spacer 1, 5.8S ribosomal RNA gene and internal transcribed spacer 2, complete sequence; and 26S ribosomal RNA gene, partial sequence	893	893	100%	0.0	91%	
AY240004.1	Dendrobium victoriae-reginae 18S ribosomal RNA gene, partial sequence; internal transcribed spacer 1, 5.8S ribosomal RNA gene and internal transcribed spacer 2, complete sequence; and 26S ribosomal RNA gene, partial sequence	887	887	100%	0.0	90%	
HM590386.1	Dendrobium miyakei voucher NCHU-D89331201-1020 18S ribosomal RNA gene, partial sequence; internal transcribed spacer 1, 5.8S ribosomal RNA gene, and internal transcribed spacer 2, complete sequence; and 26S ribosomal RNA gene, partial sequence	867	867	100%	0.0	90%	

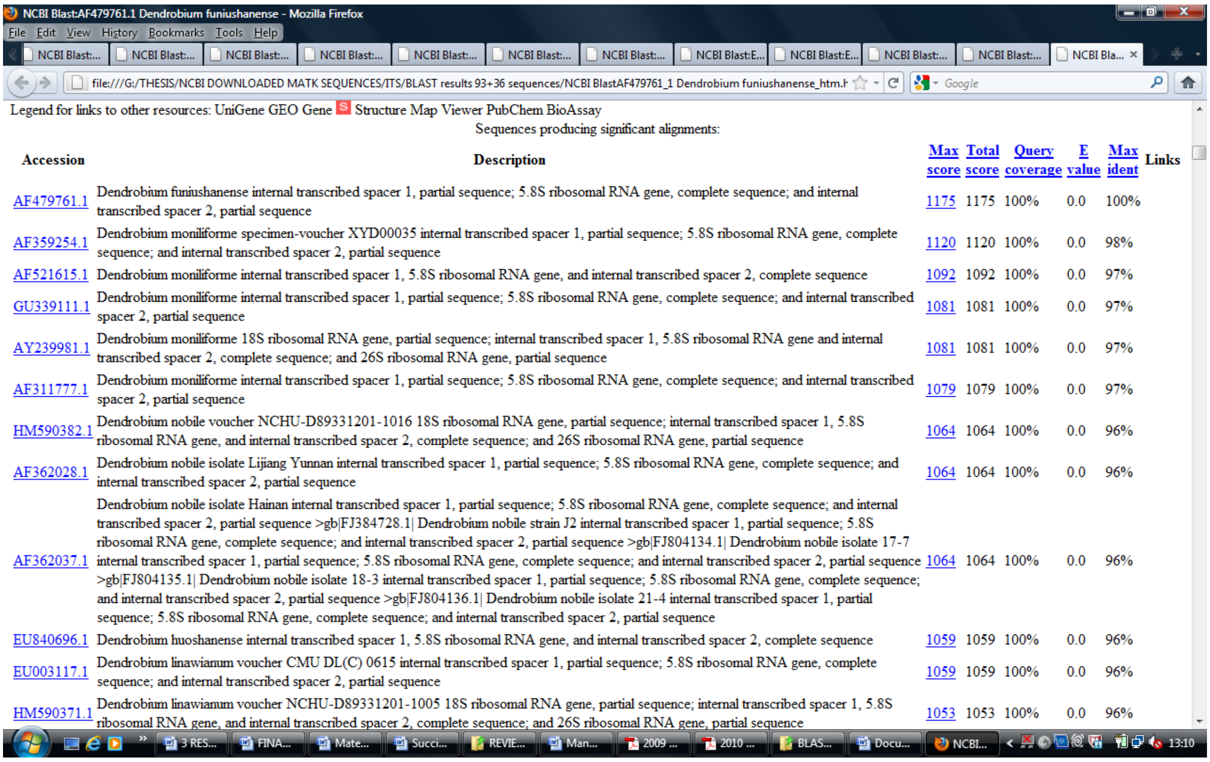

NCBI Blast:AF479761.1 Dendrobium funiushanense - Mozilla Firefox

Legend for links to other resources: UniGene GEO Gene Structure Map Viewer PubChem BioAssay

Sequences producing significant alignments:

Accession	Description	Max score	Total score	Query coverage	E value	Max ident	Links
AF479761.1	Dendrobium funiushanense internal transcribed spacer 1, partial sequence; 5.8S ribosomal RNA gene, complete sequence; and internal transcribed spacer 2, partial sequence	1175	1175	100%	0.0	100%	
AF359254.1	Dendrobium moniliforme specimen-voucher XYD00035 internal transcribed spacer 1, partial sequence; 5.8S ribosomal RNA gene, complete sequence; and internal transcribed spacer 2, partial sequence	1120	1120	100%	0.0	98%	
AF521615.1	Dendrobium moniliforme internal transcribed spacer 1, 5.8S ribosomal RNA gene, and internal transcribed spacer 2, complete sequence	1092	1092	100%	0.0	97%	
GU339111.1	Dendrobium moniliforme internal transcribed spacer 1, partial sequence; 5.8S ribosomal RNA gene, complete sequence; and internal transcribed spacer 2, partial sequence	1081	1081	100%	0.0	97%	
AY239981.1	Dendrobium moniliforme 18S ribosomal RNA gene, partial sequence; internal transcribed spacer 1, 5.8S ribosomal RNA gene and internal transcribed spacer 2, complete sequence; and 26S ribosomal RNA gene, partial sequence	1081	1081	100%	0.0	97%	
AF311777.1	Dendrobium moniliforme internal transcribed spacer 1, partial sequence; 5.8S ribosomal RNA gene, complete sequence; and internal transcribed spacer 2, partial sequence	1079	1079	100%	0.0	97%	
HM590382.1	Dendrobium nobile voucher NCHU-D89331201-1016 18S ribosomal RNA gene, partial sequence; internal transcribed spacer 1, 5.8S ribosomal RNA gene, and internal transcribed spacer 2, complete sequence; and 26S ribosomal RNA gene, partial sequence	1064	1064	100%	0.0	96%	
AF362028.1	Dendrobium nobile isolate Lijiang Yunnan internal transcribed spacer 1, partial sequence; 5.8S ribosomal RNA gene, complete sequence; and internal transcribed spacer 2, partial sequence	1064	1064	100%	0.0	96%	
AF362037.1	Dendrobium nobile isolate Hainan internal transcribed spacer 1, partial sequence; 5.8S ribosomal RNA gene, complete sequence; and internal transcribed spacer 2, partial sequence >gb|FJ384728.1| Dendrobium nobile strain J2 internal transcribed spacer 1, partial sequence; 5.8S ribosomal RNA gene, complete sequence; and internal transcribed spacer 2, partial sequence >gb|FJ804134.1| Dendrobium nobile isolate 17-7 internal transcribed spacer 1, partial sequence; 5.8S ribosomal RNA gene, complete sequence; and internal transcribed spacer 2, partial sequence >gb|FJ804135.1| Dendrobium nobile isolate 18-3 internal transcribed spacer 1, partial sequence; 5.8S ribosomal RNA gene, complete sequence; and internal transcribed spacer 2, partial sequence >gb|FJ804136.1| Dendrobium nobile isolate 21-4 internal transcribed spacer 1, partial sequence; 5.8S ribosomal RNA gene, complete sequence; and internal transcribed spacer 2, partial sequence	1064	1064	100%	0.0	96%	
EU840696.1	Dendrobium huoshanense internal transcribed spacer 1, 5.8S ribosomal RNA gene, and internal transcribed spacer 2, complete sequence	1059	1059	100%	0.0	96%	
EU003117.1	Dendrobium linawianum voucher CMU DL(C) 0615 internal transcribed spacer 1, partial sequence; 5.8S ribosomal RNA gene, complete sequence; and internal transcribed spacer 2, partial sequence	1059	1059	100%	0.0	96%	
HM590371.1	Dendrobium linawianum voucher NCHU-D89331201-1005 18S ribosomal RNA gene, partial sequence; internal transcribed spacer 1, 5.8S ribosomal RNA gene, and internal transcribed spacer 2, complete sequence; and 26S ribosomal RNA gene, partial sequence	1053	1053	100%	0.0	96%	

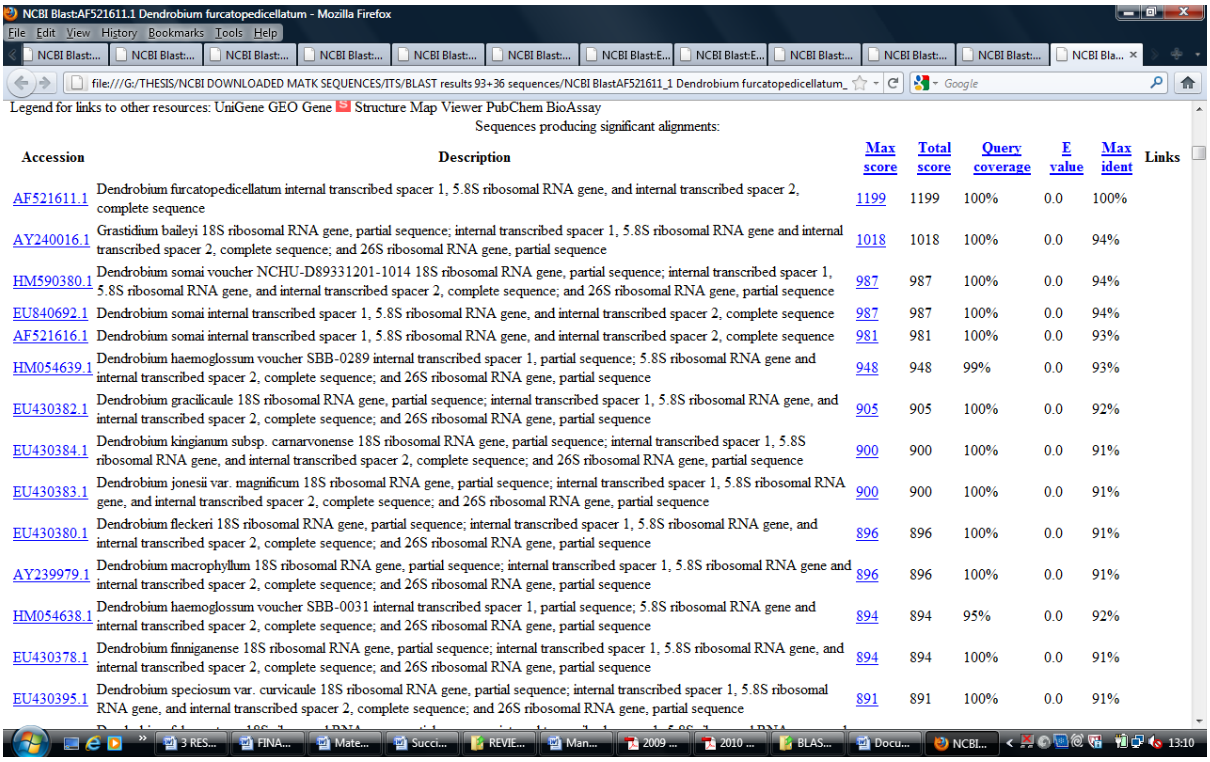

NCBI Blast:AF521611.1 Dendrobium furcatopedicellatum - Mozilla Firefox

Legend for links to other resources: UniGene GEO Gene Structure Map Viewer PubChem BioAssay

Sequences producing significant alignments:

Accession	Description	Max score	Total score	Query coverage	E value	Max ident	Links
AF521611.1	Dendrobium furcatopedicellatum internal transcribed spacer 1, 5.8S ribosomal RNA gene, and internal transcribed spacer 2, complete sequence	1199	1199	100%	0.0	100%	
AY240016.1	Grastidium baileyi 18S ribosomal RNA gene, partial sequence; internal transcribed spacer 1, 5.8S ribosomal RNA gene and internal transcribed spacer 2, complete sequence; and 26S ribosomal RNA gene, partial sequence	1018	1018	100%	0.0	94%	
HM590380.1	Dendrobium somai voucher NCHU-D89331201-1014 18S ribosomal RNA gene, partial sequence; internal transcribed spacer 1, 5.8S ribosomal RNA gene, and internal transcribed spacer 2, complete sequence; and 26S ribosomal RNA gene, partial sequence	987	987	100%	0.0	94%	
EU840692.1	Dendrobium somai internal transcribed spacer 1, 5.8S ribosomal RNA gene, and internal transcribed spacer 2, complete sequence	987	987	100%	0.0	94%	
AF521616.1	Dendrobium somai internal transcribed spacer 1, 5.8S ribosomal RNA gene, and internal transcribed spacer 2, complete sequence	981	981	100%	0.0	93%	
HM054639.1	Dendrobium haemoglossum voucher SBB-0289 internal transcribed spacer 1, partial sequence; 5.8S ribosomal RNA gene and internal transcribed spacer 2, complete sequence; and 26S ribosomal RNA gene, partial sequence	948	948	99%	0.0	93%	
EU430382.1	Dendrobium gracilicaule 18S ribosomal RNA gene, partial sequence; internal transcribed spacer 1, 5.8S ribosomal RNA gene, and internal transcribed spacer 2, complete sequence; and 26S ribosomal RNA gene, partial sequence	905	905	100%	0.0	92%	
EU430384.1	Dendrobium kingianum subsp. carnarvonense 18S ribosomal RNA gene, partial sequence; internal transcribed spacer 1, 5.8S ribosomal RNA gene, and internal transcribed spacer 2, complete sequence; and 26S ribosomal RNA gene, partial sequence	900	900	100%	0.0	91%	
EU430383.1	Dendrobium jonesii var. magnificum 18S ribosomal RNA gene, partial sequence; internal transcribed spacer 1, 5.8S ribosomal RNA gene, and internal transcribed spacer 2, complete sequence; and 26S ribosomal RNA gene, partial sequence	900	900	100%	0.0	91%	
EU430380.1	Dendrobium fleckeri 18S ribosomal RNA gene, partial sequence; internal transcribed spacer 1, 5.8S ribosomal RNA gene, and internal transcribed spacer 2, complete sequence; and 26S ribosomal RNA gene, partial sequence	896	896	100%	0.0	91%	
AY239979.1	Dendrobium macrophyllum 18S ribosomal RNA gene, partial sequence; internal transcribed spacer 1, 5.8S ribosomal RNA gene and internal transcribed spacer 2, complete sequence; and 26S ribosomal RNA gene, partial sequence	896	896	100%	0.0	91%	
HM054638.1	Dendrobium haemoglossum voucher SBB-0031 internal transcribed spacer 1, partial sequence; 5.8S ribosomal RNA gene and internal transcribed spacer 2, complete sequence; and 26S ribosomal RNA gene, partial sequence	894	894	95%	0.0	92%	
EU430378.1	Dendrobium finniganense 18S ribosomal RNA gene, partial sequence; internal transcribed spacer 1, 5.8S ribosomal RNA gene, and internal transcribed spacer 2, complete sequence; and 26S ribosomal RNA gene, partial sequence	894	894	100%	0.0	91%	
EU430395.1	Dendrobium speciosum var. curvicaule 18S ribosomal RNA gene, partial sequence; internal transcribed spacer 1, 5.8S ribosomal RNA gene, and internal transcribed spacer 2, complete sequence; and 26S ribosomal RNA gene, partial sequence	891	891	100%	0.0	91%	

NCBI Blast:GU339105.1 Dendrobium gibsonii - Mozilla Firefox

file:///G:/THESIS/NCBI DOWNLOADED MATK SEQUENCES/ITS/BLAST results 93+36 sequences/NCBI BlastGU339105_1 Dendrobium gibsonii_htm.htm

Legend for links to other resources: UniGene GEO Gene Structure Map Viewer PubChem BioAssay

Sequences producing significant alignments:

Accession	Description	Max score	Total score	Query coverage	E value	Max ident	Links
GU339105.1	Dendrobium gibsonii internal transcribed spacer 1, partial sequence; 5.8S ribosomal RNA gene, complete sequence; and internal transcribed spacer 2, partial sequence	1175	1175	100%	0.0	100%	
AF362041.1	Dendrobium fimbriatum isolate Yunnan internal transcribed spacer 1, partial sequence; 5.8S ribosomal RNA gene, complete sequence; and internal transcribed spacer 2, partial sequence	1162	1162	100%	0.0	99%	
GU339106.1	Dendrobium christyanum internal transcribed spacer 1, partial sequence; 5.8S ribosomal RNA gene, complete sequence; and internal transcribed spacer 2, partial sequence	830	830	100%	0.0	90%	
EF629325.1	Dendrobium christyanum internal transcribed spacer 1, partial sequence; 5.8S ribosomal RNA gene, complete sequence; and internal transcribed spacer 2, partial sequence	822	822	100%	0.0	90%	
AY239967.1	Dendrobium formosum 18S ribosomal RNA gene, partial sequence; internal transcribed spacer 1, 5.8S ribosomal RNA gene and internal transcribed spacer 2, complete sequence; and 26S ribosomal RNA gene, partial sequence	821	821	100%	0.0	90%	
AF362027.1	Dendrobium cariniferum internal transcribed spacer 1, partial sequence; 5.8S ribosomal RNA gene, complete sequence; and internal transcribed spacer 2, partial sequence	804	804	100%	0.0	89%	
AF362030.1	Dendrobium williamsonii internal transcribed spacer 1, partial sequence; 5.8S ribosomal RNA gene, complete sequence; and internal transcribed spacer 2, partial sequence	804	804	100%	0.0	89%	
EU592015.1	Dendrobium bellatulum internal transcribed spacer 1, partial sequence; 5.8S ribosomal RNA gene, complete sequence; and internal transcribed spacer 2, partial sequence	802	802	100%	0.0	89%	
FJ428220.1	Dendrobium williamsonii internal transcribed spacer 1, partial sequence; 5.8S ribosomal RNA gene, complete sequence; and internal transcribed spacer 2, partial sequence	797	797	100%	0.0	89%	
GU339112.1	Dendrobium longicornu internal transcribed spacer 1, partial sequence; 5.8S ribosomal RNA gene, complete sequence; and internal transcribed spacer 2, partial sequence	791	791	100%	0.0	89%	
HM590377.1	Dendrobium hancockii voucher NCHU-D89331201-1011 18S ribosomal RNA gene, partial sequence; internal transcribed spacer 1, 5.8S ribosomal RNA gene, and internal transcribed spacer 2, complete sequence; and 26S ribosomal RNA gene, partial sequence	785	785	100%	0.0	89%	
DQ058796.1	Dendrobium longicornu voucher CJ-SC02-1 internal transcribed spacer 1, 5.8S ribosomal RNA gene, and internal transcribed spacer 2, complete sequence	785	785	100%	0.0	88%	
DQ058788.1	Dendrobium dixanthum voucher HH-JL03-1 internal transcribed spacer 1, 5.8S ribosomal RNA gene, and internal transcribed spacer 2, complete sequence	785	785	100%	0.0	89%	

NCBI Blast:AY239969.1 Dendrobium goldfinchii - Mozilla Firefox

file:///G:/THESIS/NCBI DOWNLOADED MATK SEQUENCES/ITS/BLAST results 93+36 sequences/NCBI BlastAY239969_1 Dendrobium goldfinchii_htm.htm

Legend for links to other resources: UniGene GEO Gene Structure Map Viewer PubChem BioAssay

Sequences producing significant alignments:

Accession	Description	Max score	Total score	Query coverage	E value	Max ident	Links
AY239969.1	Dendrobium goldfinchii 18S ribosomal RNA gene, partial sequence; internal transcribed spacer 1, 5.8S ribosomal RNA gene and internal transcribed spacer 2, complete sequence; and 26S ribosomal RNA gene, partial sequence	1223	1223	100%	0.0	100%	
AY239988.1	Dendrobium philippinense 18S ribosomal RNA gene, partial sequence; internal transcribed spacer 1, 5.8S ribosomal RNA gene and internal transcribed spacer 2, complete sequence; and 26S ribosomal RNA gene, partial sequence	1064	1064	100%	0.0	95%	
AY239975.1	Dendrobium junceum 18S ribosomal RNA gene, partial sequence; internal transcribed spacer 1, 5.8S ribosomal RNA gene and internal transcribed spacer 2, complete sequence; and 26S ribosomal RNA gene, partial sequence	977	977	100%	0.0	93%	
AY239961.1	Dendrobium confusum 18S ribosomal RNA gene, partial sequence; internal transcribed spacer 1, 5.8S ribosomal RNA gene and internal transcribed spacer 2, complete sequence; and 26S ribosomal RNA gene, partial sequence	972	972	100%	0.0	93%	
AY239951.1	Dendrobium aloifolium 18S ribosomal RNA gene, partial sequence; internal transcribed spacer 1, 5.8S ribosomal RNA gene and internal transcribed spacer 2, complete sequence; and 26S ribosomal RNA gene, partial sequence	950	950	100%	0.0	92%	
AY240003.1	Dendrobium usterioides 18S ribosomal RNA gene, partial sequence; internal transcribed spacer 1, 5.8S ribosomal RNA gene and internal transcribed spacer 2, complete sequence; and 26S ribosomal RNA gene, partial sequence	941	941	100%	0.0	92%	
AY239989.1	Dendrobium quadrangulare 18S ribosomal RNA gene, partial sequence; internal transcribed spacer 1, 5.8S ribosomal RNA gene and internal transcribed spacer 2, complete sequence; and 26S ribosomal RNA gene, partial sequence	939	939	100%	0.0	92%	
AY239972.1	Dendrobium indivisum 18S ribosomal RNA gene, partial sequence; internal transcribed spacer 1, 5.8S ribosomal RNA gene and internal transcribed spacer 2, complete sequence; and 26S ribosomal RNA gene, partial sequence	933	933	100%	0.0	92%	
AF521609.1	Dendrobium equitans internal transcribed spacer 1, 5.8S ribosomal RNA gene, and internal transcribed spacer 2, complete sequence >gb|EU840701.1| Dendrobium equitans internal transcribed spacer 1, 5.8S ribosomal RNA gene, and internal transcribed spacer 2, complete sequence	933	933	96%	0.0	93%	
AY239953.1	Dendrobium anceps 18S ribosomal RNA gene, partial sequence; internal transcribed spacer 1, 5.8S ribosomal RNA gene and internal transcribed spacer 2, complete sequence; and 26S ribosomal RNA gene, partial sequence	928	928	100%	0.0	91%	
EU840698.1	Dendrobium quadrangulare internal transcribed spacer 1, 5.8S ribosomal RNA gene, and internal transcribed spacer 2, complete sequence	913	913	95%	0.0	92%	
AY239963.1	Dendrobium crumenatum 18S ribosomal RNA gene, partial sequence; internal transcribed spacer 1, 5.8S ribosomal RNA gene and internal transcribed spacer 2, complete sequence; and 26S ribosomal RNA gene, partial sequence	911	911	100%	0.0	91%	
HM054548.1	Dendrobium anceps voucher SBB-0301 18S ribosomal RNA gene, partial sequence; internal transcribed spacer 1, 5.8S ribosomal RNA gene, and internal transcribed spacer 2, complete sequence; and 26S ribosomal RNA gene, partial sequence	909	909	97%	0.0	92%	

NCBI Blast:AY239970.1 Dendrobium goldschmidtianum - Mozilla Firefox

file:///G:/THESIS/NCBI DOWNLOADED MATK SEQUENCES/ITS/BLAST results 93+36 sequences/NCBI BlastAY239970_1 Dendrobium goldschmidtianum_h

Legend for links to other resources: UniGene GEO Gene Structure Map Viewer PubChem BioAssay

Sequences producing significant alignments:

Accession	Description	Max score	Total score	Query coverage	E value	Max ident	Links
HM590386.1	Dendrobium miyakei voucher NCHU-D89331201-1020 18S ribosomal RNA gene, partial sequence; internal transcribed spacer 1, 5.8S ribosomal RNA gene, and internal transcribed spacer 2, complete sequence; and 26S ribosomal RNA gene, partial sequence	1229	1229	100%	0.0	100%	
AY239970.1	Dendrobium goldschmidtianum 18S ribosomal RNA gene, partial sequence; internal transcribed spacer 1, 5.8S ribosomal RNA gene and internal transcribed spacer 2, complete sequence; and 26S ribosomal RNA gene, partial sequence	1229	1229	100%	0.0	100%	
AF521614.1	Dendrobium miyakei internal transcribed spacer 1, 5.8S ribosomal RNA gene, and internal transcribed spacer 2, complete sequence	1184	1184	96%	0.0	100%	
EU840695.1	Dendrobium goldschmidtianum internal transcribed spacer 1, 5.8S ribosomal RNA gene, and internal transcribed spacer 2, complete sequence	1179	1179	96%	0.0	99%	
EU840694.1	Dendrobium victoriae-reginae internal transcribed spacer 1, 5.8S ribosomal RNA gene, and internal transcribed spacer 2, complete sequence	1146	1146	96%	0.0	98%	
AY239994.1	Dendrobium serratilabium 18S ribosomal RNA gene, partial sequence; internal transcribed spacer 1, 5.8S ribosomal RNA gene and internal transcribed spacer 2, complete sequence; and 26S ribosomal RNA gene, partial sequence	1088	1088	100%	0.0	96%	
HM590385.1	Dendrobium chameleon voucher NCHU-D89331201-1019 18S ribosomal RNA gene, partial sequence; internal transcribed spacer 1, 5.8S ribosomal RNA gene, and internal transcribed spacer 2, complete sequence; and 26S ribosomal RNA gene, partial sequence	1083	1083	100%	0.0	96%	
AY239960.1	Dendrobium chameleon 18S ribosomal RNA gene, partial sequence; internal transcribed spacer 1, 5.8S ribosomal RNA gene and internal transcribed spacer 2, complete sequence; and 26S ribosomal RNA gene, partial sequence	1061	1061	100%	0.0	95%	
AY240006.1	Dendrobium yeageri 18S ribosomal RNA gene, partial sequence; internal transcribed spacer 1, 5.8S ribosomal RNA gene and internal transcribed spacer 2, complete sequence; and 26S ribosomal RNA gene, partial sequence	1055	1055	100%	0.0	95%	
AY239958.1	Dendrobium cerauia 18S ribosomal RNA gene, partial sequence; internal transcribed spacer 1, 5.8S ribosomal RNA gene and internal transcribed spacer 2, complete sequence; and 26S ribosomal RNA gene, partial sequence	1042	1042	100%	0.0	95%	
AY240004.1	Dendrobium victoriae-reginae 18S ribosomal RNA gene, partial sequence; internal transcribed spacer 1, 5.8S ribosomal RNA gene and internal transcribed spacer 2, complete sequence; and 26S ribosomal RNA gene, partial sequence	1037	1037	100%	0.0	94%	
AF521607.1	Dendrobium chameleon internal transcribed spacer 1, 5.8S ribosomal RNA gene, and internal transcribed spacer 2, complete sequence	1005	1005	96%	0.0	95%	
AY239976.1	Dendrobium lancifolium 18S ribosomal RNA gene, partial sequence; internal transcribed spacer 1, 5.8S ribosomal RNA gene and internal transcribed spacer 2, complete sequence; and 26S ribosomal RNA gene, partial sequence	933	933	100%	0.0	92%	
AY239966.1	Dendrobium fairchildae 18S ribosomal RNA gene, partial sequence; internal transcribed spacer 1, 5.8S ribosomal RNA gene and internal transcribed spacer 2, complete sequence; and 26S ribosomal RNA gene, partial sequence	878	878	100%	0.0	90%	

NCBI Blast:AY239971.1 Dendrobium govidjoae - Mozilla Firefox

file:///G:/THESIS/NCBI DOWNLOADED MATK SEQUENCES/ITS/BLAST results 93+36 sequences/NCBI BlastAY239971_1 Dendrobium govidjoae_htm.htm

Legend for links to other resources: UniGene GEO Gene Structure Map Viewer PubChem BioAssay

Sequences producing significant alignments:

Accession	Description	Max score	Total score	Query coverage	E value	Max ident	Links
AY239971.1	Dendrobium govidjoae 18S ribosomal RNA gene, partial sequence; internal transcribed spacer 1, 5.8S ribosomal RNA gene and internal transcribed spacer 2, complete sequence; and 26S ribosomal RNA gene, partial sequence	1240	1240	100%	0.0	100%	
AY239995.1	Dendrobium sinuatum 18S ribosomal RNA gene, partial sequence; internal transcribed spacer 1, 5.8S ribosomal RNA gene and internal transcribed spacer 2, complete sequence; and 26S ribosomal RNA gene, partial sequence	928	928	97%	0.0	92%	
AY239946.1	Bryobium pubescens 18S ribosomal RNA gene, partial sequence; internal transcribed spacer 1, 5.8S ribosomal RNA gene and internal transcribed spacer 2, complete sequence; and 26S ribosomal RNA gene, partial sequence	785	785	97%	0.0	88%	
AF521071.1	Eria ferruginea internal transcribed spacer 1, 5.8S ribosomal RNA gene, and internal transcribed spacer 2, complete sequence	728	728	94%	0.0	87%	
EF670387.1	Collabium simplex 18S ribosomal RNA gene, partial sequence; internal transcribed spacer 1, 5.8S ribosomal RNA gene, and internal transcribed spacer 2, complete sequence; and 28S ribosomal RNA gene, partial sequence	483	483	90%	8e-133	81%	
AF521073.1	Appendicula cornuta internal transcribed spacer 1, 5.8S ribosomal RNA gene, and internal transcribed spacer 2, complete sequence	472	472	79%	2e-129	83%	
AY240014.1	Eria aff. javanica CBG 740854 18S ribosomal RNA gene, partial sequence; internal transcribed spacer 1, 5.8S ribosomal RNA gene and internal transcribed spacer 2, complete sequence; and 26S ribosomal RNA gene, partial sequence	470	470	92%	7e-129	81%	
AF521072.1	Podochilus cultratus internal transcribed spacer 1, partial sequence; 5.8S ribosomal RNA gene, and internal transcribed spacer 2, complete sequence	466	466	91%	9e-128	81%	
AF284727.1	Eulophia graminea internal transcribed spacer 1, 5.8S ribosomal RNA gene, and internal transcribed spacer 2, complete sequence	442	442	62%	1e-120	86%	
AB239261.1	Gastrorchis humblotii genes for ITS1, 5.8S rRNA, ITS2, complete sequence, clone: 5	440	440	76%	5e-120	82%	
AB222024.1	Gastrorchis humblotii genes for ITS1, 5.8S rRNA, ITS2, complete sequence >dbj|AB239260.1| Gastrorchis humblotii genes for ITS1, 5.8S rRNA, ITS2, complete sequence, clone: 4	440	440	76%	5e-120	82%	
AB239259.1	Gastrorchis humblotii genes for ITS1, 5.8S rRNA, ITS2, complete sequence, clone: 3	440	440	76%	5e-120	82%	
AB239258.1	Gastrorchis humblotii genes for ITS1, 5.8S rRNA, ITS2, complete sequence, clone: 2	440	440	76%	5e-120	82%	
AF521052.1	Collabium sp. Chase O-821 internal transcribed spacer 1, 5.8S ribosomal RNA gene, and internal transcribed spacer 2, complete sequence	440	440	71%	5e-120	83%	
AB222033.1	Phaius graeffei genes for ITS1, 5.8S rRNA, ITS2, complete sequence	438	438	76%	2e-119	82%	
AF506297.1	Campylocentrum lansbergii isolate 4 18S ribosomal RNA gene, partial sequence; internal transcribed spacer 1, 5.8S ribosomal RNA gene and internal transcribed spacer 2, complete sequence; and 26S ribosomal RNA gene, partial sequence	436	436	83%	7e-119	81%	
EF079363.1	Chysis bractescens 18S ribosomal RNA gene, partial sequence; internal transcribed spacer 1, 5.8S ribosomal RNA gene, and internal transcribed spacer 2, complete sequence; and 26S ribosomal RNA gene, partial sequence	435	435	83%	2e-118	81%	

NCBI Blast:EU430382.1 Dendrobium gracilicaule - Mozilla Firefox

file:///G:/THESIS/NCBI DOWNLOADED MATK SEQUENCES/ITS/BLAST results 93+36 sequences/NCBI BlastEU430382_1 Dendrobium gracilicaule_htm.htm

Legend for links to other resources: UniGene GEO Gene Structure Map Viewer PubChem BioAssay

Sequences producing significant alignments:

Accession	Description	Max score	Total score	Query coverage	E value	Max ident	Links
EU430382.1	Dendrobium gracilicaule 18S ribosomal RNA gene, partial sequence; internal transcribed spacer 1, 5.8S ribosomal RNA gene, and internal transcribed spacer 2, complete sequence; and 26S ribosomal RNA gene, partial sequence	1319	1319	100%	0.0	100%	
EU430384.1	Dendrobium kingianum subsp. carnarvonense 18S ribosomal RNA gene, partial sequence; internal transcribed spacer 1, 5.8S ribosomal RNA gene, and internal transcribed spacer 2, complete sequence; and 26S ribosomal RNA gene, partial sequence	1236	1236	100%	0.0	97%	
EU430383.1	Dendrobium jonesii var. magnificum 18S ribosomal RNA gene, partial sequence; internal transcribed spacer 1, 5.8S ribosomal RNA gene, and internal transcribed spacer 2, complete sequence; and 26S ribosomal RNA gene, partial sequence	1221	1221	99%	0.0	97%	
EU430395.1	Dendrobium speciosum var. curvicaule 18S ribosomal RNA gene, partial sequence; internal transcribed spacer 1, 5.8S ribosomal RNA gene, and internal transcribed spacer 2, complete sequence; and 26S ribosomal RNA gene, partial sequence	1216	1216	100%	0.0	97%	
EU430398.1	Dendrobium speciosum var. pedunculatum 18S ribosomal RNA gene, partial sequence; internal transcribed spacer 1, 5.8S ribosomal RNA gene, and internal transcribed spacer 2, complete sequence; and 26S ribosomal RNA gene, partial sequence	1190	1190	98%	0.0	96%	
EU430385.1	Dendrobium kingianum var. pulcherrimum 18S ribosomal RNA gene, partial sequence; internal transcribed spacer 1, 5.8S ribosomal RNA gene, and internal transcribed spacer 2, complete sequence; and 26S ribosomal RNA gene, partial sequence	1188	1188	99%	0.0	96%	
EU430397.1	Dendrobium speciosum var. hillii 18S ribosomal RNA gene, partial sequence; internal transcribed spacer 1, 5.8S ribosomal RNA gene, and internal transcribed spacer 2, complete sequence; and 26S ribosomal RNA gene, partial sequence	1182	1182	100%	0.0	96%	
EU430391.1	Dendrobium speciosum var. blackdownense 18S ribosomal RNA gene, partial sequence; internal transcribed spacer 1, 5.8S ribosomal RNA gene, and internal transcribed spacer 2, complete sequence; and 26S ribosomal RNA gene, partial sequence	1182	1182	100%	0.0	96%	
EU430377.1	Dendrobium falcorostrum 18S ribosomal RNA gene, partial sequence; internal transcribed spacer 1, 5.8S ribosomal RNA gene, and internal transcribed spacer 2, complete sequence; and 26S ribosomal RNA gene, partial sequence	1182	1182	93%	0.0	98%	
EU430393.1	Dendrobium speciosum var. capricornicum 18S ribosomal RNA gene, partial sequence; internal transcribed spacer 1, 5.8S ribosomal RNA gene, and internal transcribed spacer 2, complete sequence; and 26S ribosomal RNA gene, partial sequence	1179	1179	100%	0.0	96%	
EU430394.1	Dendrobium speciosum var. carnarvonense 18S ribosomal RNA gene, partial sequence; internal transcribed spacer 1, 5.8S ribosomal RNA gene, and internal transcribed spacer 2, complete sequence; and 26S ribosomal RNA gene, partial sequence	1175	1175	99%	0.0	96%	
EU430396.1	Dendrobium speciosum var. grandiflorum 18S ribosomal RNA gene, partial sequence; internal transcribed spacer 1, 5.8S ribosomal RNA gene, and internal transcribed spacer 2, complete sequence; and 26S ribosomal RNA gene, partial sequence	1170	1170	99%	0.0	96%	
EU430399.1	Dendrobium speciosum var. speciosum 18S ribosomal RNA gene, partial sequence; internal transcribed spacer 1, 5.8S ribosomal RNA gene, and internal transcribed spacer 2, complete sequence; and 26S ribosomal RNA gene, partial sequence	1166	1166	98%	0.0	96%	
[illegible]	[illegible]	[illegible]	[illegible]	[illegible]	[illegible]	[illegible]	

NCBI Blast:HM590377.1 Dendrobium hancockii - Mozilla Firefox

file:///G:/THESIS/NCBI DOWNLOADED MATK SEQUENCES/ITS/BLAST results 93+36 sequences/NCBI BlastHM590377_1 Dendrobium hancockii_htm.htm

Legend for links to other resources: UniGene GEO Gene Structure Map Viewer PubChem BioAssay

Sequences producing significant alignments:

Accession	Description	Max score	Total score	Query coverage	E value	Max ident	Links
HM590377.1	Dendrobium hancockii voucher NCHU-D89331201-1011 18S ribosomal RNA gene, partial sequence; internal transcribed spacer 1, 5.8S ribosomal RNA gene, and internal transcribed spacer 2, complete sequence; and 26S ribosomal RNA gene, partial sequence	1639	1639	100%	0.0	100%	
HM590375.1	Dendrobium aurantiacum voucher NCHU-D89331201-1009 18S ribosomal RNA gene, partial sequence; internal transcribed spacer 1, 5.8S ribosomal RNA gene, and internal transcribed spacer 2, complete sequence; and 26S ribosomal RNA gene, partial sequence	1205	1205	100%	0.0	91%	
HM590392.1	Dendrobium fimbriatum voucher NCHU-D89331201-1029 18S ribosomal RNA gene, partial sequence; internal transcribed spacer 1, 5.8S ribosomal RNA gene, and internal transcribed spacer 2, complete sequence; and 26S ribosomal RNA gene, partial sequence	1194	1194	100%	0.0	91%	
HM590387.1	Dendrobium clavatum voucher NCHU-D89331201-1021 18S ribosomal RNA gene, partial sequence; internal transcribed spacer 1, 5.8S ribosomal RNA gene, and internal transcribed spacer 2, complete sequence; and 26S ribosomal RNA gene, partial sequence	1186	1186	100%	0.0	90%	
AF362025.1	Dendrobium hancockii internal transcribed spacer 1, partial sequence; 5.8S ribosomal RNA gene, complete sequence; and internal transcribed spacer 2, partial sequence	1177	1177	72%	0.0	99%	
DQ058787.1	Dendrobium hancockii voucher XY-GZ03-1 internal transcribed spacer 1, 5.8S ribosomal RNA gene, and internal transcribed spacer 2, complete sequence	1175	1175	72%	0.0	99%	
HM590383.1	Dendrobium chrysotoxum voucher NCHU-D89331201-1017 18S ribosomal RNA gene, partial sequence; internal transcribed spacer 1, 5.8S ribosomal RNA gene, and internal transcribed spacer 2, complete sequence; and 26S ribosomal RNA gene, partial sequence	1147	1147	100%	0.0	90%	
HM590376.1	Dendrobium chrysanthum voucher NCHU-D89331201-1010 18S ribosomal RNA gene, partial sequence; internal transcribed spacer 1, 5.8S ribosomal RNA gene, and internal transcribed spacer 2, complete sequence; and 26S ribosomal RNA gene, partial sequence	1136	1136	100%	0.0	89%	
HM590378.1	Dendrobium parishii voucher NCHU-D89331201-1012 18S ribosomal RNA gene, partial sequence; internal transcribed spacer 1, 5.8S ribosomal RNA gene, and internal transcribed spacer 2, complete sequence; and 26S ribosomal RNA gene, partial sequence	1110	1110	99%	0.0	89%	
HM590369.1	Dendrobium moniliforme voucher NCHU-D89331201-1003 18S ribosomal RNA gene, partial sequence; internal transcribed spacer 1, 5.8S ribosomal RNA gene, and internal transcribed spacer 2, complete sequence; and 26S ribosomal RNA gene, partial sequence	1109	1109	99%	0.0	89%	
HM590379.1	Dendrobium capillipes voucher NCHU-D89331201-1013 18S ribosomal RNA gene, partial sequence; internal transcribed spacer 1, 5.8S ribosomal RNA gene, and internal transcribed spacer 2, complete sequence; and 26S ribosomal RNA gene, partial sequence	1105	1105	96%	0.0	90%	
HM590371.1	Dendrobium linawianum voucher NCHU-D89331201-1005 18S ribosomal RNA gene, partial sequence; internal transcribed spacer 1, 5.8S ribosomal RNA gene, and internal transcribed spacer 2, complete sequence; and 26S ribosomal RNA gene, partial sequence	1101	1101	100%	0.0	89%	
HM590384.1	Dendrobium aphyllum voucher NCHU-D89331201-1018 18S ribosomal RNA gene, partial sequence; internal transcribed spacer 1, 5.8S ribosomal RNA gene, and internal transcribed spacer 2, complete sequence; and 26S ribosomal RNA gene, partial sequence	1088	1088	98%	0.0	89%	
[illegible]	[illegible]	[illegible]	[illegible]	[illegible]	[illegible]	[illegible]	

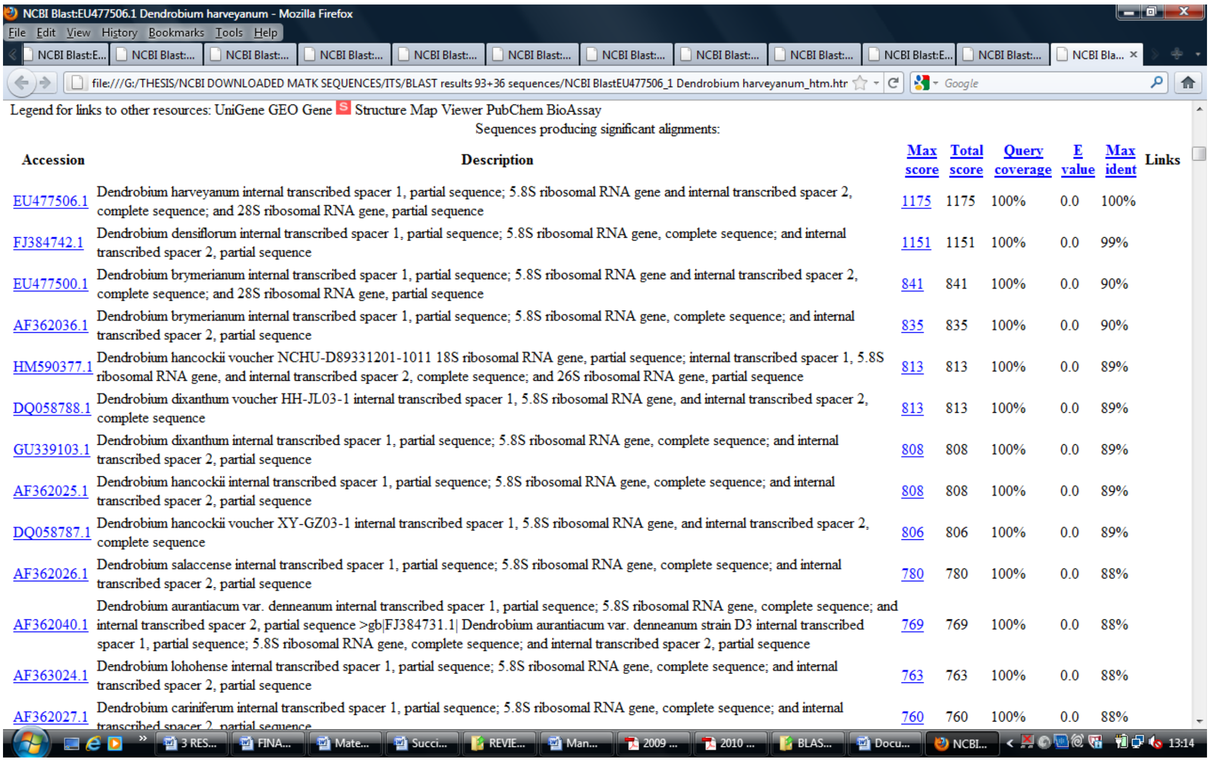

Legend for links to other resources: UniGene GEO Gene S Structure Map Viewer PubChem BioAssay

Sequences producing significant alignments:

Accession	Description	Max score	Total score	Query coverage	E value	Max ident	Links
EU477506.1	Dendrobium harveyanum internal transcribed spacer 1, partial sequence; 5.8S ribosomal RNA gene and internal transcribed spacer 2, complete sequence; and 28S ribosomal RNA gene, partial sequence	1175	1175	100%	0.0	100%	
FJ384742.1	Dendrobium densiflorum internal transcribed spacer 1, partial sequence; 5.8S ribosomal RNA gene, complete sequence; and internal transcribed spacer 2, partial sequence	1151	1151	100%	0.0	99%	
EU477500.1	Dendrobium brymerianum internal transcribed spacer 1, partial sequence; 5.8S ribosomal RNA gene and internal transcribed spacer 2, complete sequence; and 28S ribosomal RNA gene, partial sequence	841	841	100%	0.0	90%	
AF362036.1	Dendrobium brymerianum internal transcribed spacer 1, partial sequence; 5.8S ribosomal RNA gene, complete sequence; and internal transcribed spacer 2, partial sequence	835	835	100%	0.0	90%	
HM590377.1	Dendrobium hancockii voucher NCHU-D89331201-1011 18S ribosomal RNA gene, partial sequence; internal transcribed spacer 1, 5.8S ribosomal RNA gene, and internal transcribed spacer 2, complete sequence; and 26S ribosomal RNA gene, partial sequence	813	813	100%	0.0	89%	
DQ058788.1	Dendrobium dixanthum voucher HH-JL03-1 internal transcribed spacer 1, 5.8S ribosomal RNA gene, and internal transcribed spacer 2, complete sequence	813	813	100%	0.0	89%	
GU339103.1	Dendrobium dixanthum internal transcribed spacer 1, partial sequence; 5.8S ribosomal RNA gene, complete sequence; and internal transcribed spacer 2, partial sequence	808	808	100%	0.0	89%	
AF362025.1	Dendrobium hancockii internal transcribed spacer 1, partial sequence; 5.8S ribosomal RNA gene, complete sequence; and internal transcribed spacer 2, partial sequence	808	808	100%	0.0	89%	
DQ058787.1	Dendrobium hancockii voucher XY-GZ03-1 internal transcribed spacer 1, 5.8S ribosomal RNA gene, and internal transcribed spacer 2, complete sequence	806	806	100%	0.0	89%	
AF362026.1	Dendrobium salaccense internal transcribed spacer 1, partial sequence; 5.8S ribosomal RNA gene, complete sequence; and internal transcribed spacer 2, partial sequence	780	780	100%	0.0	88%	
AF362040.1	Dendrobium aurantiacum var. denneanum internal transcribed spacer 1, partial sequence; 5.8S ribosomal RNA gene, complete sequence; and internal transcribed spacer 2, partial sequence >gb|FJ384731.1| Dendrobium aurantiacum var. denneanum strain D3 internal transcribed spacer 1, partial sequence; 5.8S ribosomal RNA gene, complete sequence; and internal transcribed spacer 2, partial sequence	769	769	100%	0.0	88%	
AF363024.1	Dendrobium lohohense internal transcribed spacer 1, partial sequence; 5.8S ribosomal RNA gene, complete sequence; and internal transcribed spacer 2, partial sequence	763	763	100%	0.0	88%	
AF362027.1	Dendrobium cariniferum internal transcribed spacer 1, partial sequence; 5.8S ribosomal RNA gene, complete sequence; and internal transcribed spacer 2, partial sequence	760	760	100%	0.0	88%	

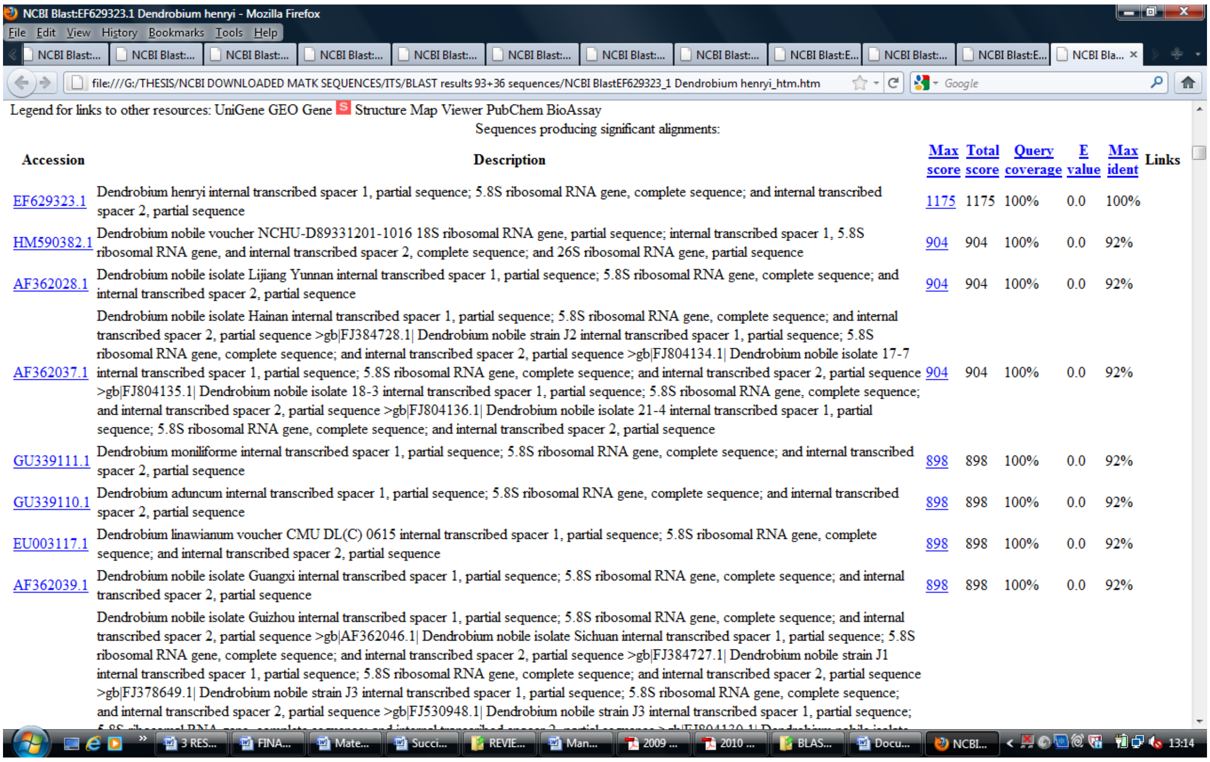

Legend for links to other resources: UniGene GEO Gene S Structure Map Viewer PubChem BioAssay

Sequences producing significant alignments:

Accession	Description	Max score	Total score	Query coverage	E value	Max ident	Links
EF629323.1	Dendrobium henryi internal transcribed spacer 1, partial sequence; 5.8S ribosomal RNA gene, complete sequence; and internal transcribed spacer 2, partial sequence	1175	1175	100%	0.0	100%	
HM590382.1	Dendrobium nobile voucher NCHU-D89331201-1016 18S ribosomal RNA gene, partial sequence; internal transcribed spacer 1, 5.8S ribosomal RNA gene, and internal transcribed spacer 2, complete sequence; and 26S ribosomal RNA gene, partial sequence	904	904	100%	0.0	92%	
AF362028.1	Dendrobium nobile isolate Lijiang Yunnan internal transcribed spacer 1, partial sequence; 5.8S ribosomal RNA gene, complete sequence; and internal transcribed spacer 2, partial sequence	904	904	100%	0.0	92%	
AF362037.1	Dendrobium nobile isolate Hainan internal transcribed spacer 1, partial sequence; 5.8S ribosomal RNA gene, complete sequence; and internal transcribed spacer 2, partial sequence >gb|FJ384728.1| Dendrobium nobile strain J2 internal transcribed spacer 1, partial sequence; 5.8S ribosomal RNA gene, complete sequence; and internal transcribed spacer 2, partial sequence >gb|FJ804134.1| Dendrobium nobile isolate 17-7 internal transcribed spacer 1, partial sequence; 5.8S ribosomal RNA gene, complete sequence; and internal transcribed spacer 2, partial sequence >gb|FJ804135.1| Dendrobium nobile isolate 18-3 internal transcribed spacer 1, partial sequence; 5.8S ribosomal RNA gene, complete sequence; and internal transcribed spacer 2, partial sequence >gb|FJ804136.1| Dendrobium nobile isolate 21-4 internal transcribed spacer 1, partial sequence; 5.8S ribosomal RNA gene, complete sequence; and internal transcribed spacer 2, partial sequence	904	904	100%	0.0	92%	
GU339111.1	Dendrobium moniliforme internal transcribed spacer 1, partial sequence; 5.8S ribosomal RNA gene, complete sequence; and internal transcribed spacer 2, partial sequence	898	898	100%	0.0	92%	
GU339110.1	Dendrobium aduncum internal transcribed spacer 1, partial sequence; 5.8S ribosomal RNA gene, complete sequence; and internal transcribed spacer 2, partial sequence	898	898	100%	0.0	92%	
EU003117.1	Dendrobium linawianum voucher CMU DL(C) 0615 internal transcribed spacer 1, partial sequence; 5.8S ribosomal RNA gene, complete sequence; and internal transcribed spacer 2, partial sequence	898	898	100%	0.0	92%	
AF362039.1	Dendrobium nobile isolate Guangxi internal transcribed spacer 1, partial sequence; 5.8S ribosomal RNA gene, complete sequence; and internal transcribed spacer 2, partial sequence	898	898	100%	0.0	92%	
	Dendrobium nobile isolate Guizhou internal transcribed spacer 1, partial sequence; 5.8S ribosomal RNA gene, complete sequence; and internal transcribed spacer 2, partial sequence >gb|AF362046.1| Dendrobium nobile isolate Sichuan internal transcribed spacer 1, partial sequence; 5.8S ribosomal RNA gene, complete sequence; and internal transcribed spacer 2, partial sequence >gb|FJ384727.1| Dendrobium nobile strain J1 internal transcribed spacer 1, partial sequence; 5.8S ribosomal RNA gene, complete sequence; and internal transcribed spacer 2, partial sequence >gb|FJ378649.1| Dendrobium nobile strain J3 internal transcribed spacer 1, partial sequence; 5.8S ribosomal RNA gene, complete sequence; and internal transcribed spacer 2, partial sequence >gb|FJ530948.1| Dendrobium nobile strain J3 internal transcribed spacer 1, partial sequence; [illegible]						

NCBI Blast:HM590368.1 Dendrobium huoshanense - Mozilla Firefox

file:///G:/THESIS/NCBI DOWNLOADED MATK SEQUENCES/ITS/BLAST results 93+36 sequences/NCBI BlastHM590368_1 Dendrobium huoshanense_htm.h

Legend for links to other resources: UniGene GEO Gene Structure Map Viewer PubChem BioAssay

Sequences producing significant alignments:

Accession	Description	Max score	Total score	Query coverage	E value	Max ident	Links
HM590368.1	Dendrobium huoshanense voucher NCHU-D89331201-1002 18S ribosomal RNA gene, partial sequence; internal transcribed spacer 1, 5.8S ribosomal RNA gene, and internal transcribed spacer 2, complete sequence; and 26S ribosomal RNA gene, partial sequence	1633	1633	100%	0.0	100%	
HM590371.1	Dendrobium linawianum voucher NCHU-D89331201-1005 18S ribosomal RNA gene, partial sequence; internal transcribed spacer 1, 5.8S ribosomal RNA gene, and internal transcribed spacer 2, complete sequence; and 26S ribosomal RNA gene, partial sequence	1293	1293	98%	0.0	93%	
HM590382.1	Dendrobium nobile voucher NCHU-D89331201-1016 18S ribosomal RNA gene, partial sequence; internal transcribed spacer 1, 5.8S ribosomal RNA gene, and internal transcribed spacer 2, complete sequence; and 26S ribosomal RNA gene, partial sequence	1275	1275	96%	0.0	93%	
HM590381.1	Dendrobium hercoglossum voucher NCHU-D89331201-1015 18S ribosomal RNA gene, partial sequence; internal transcribed spacer 1, 5.8S ribosomal RNA gene, and internal transcribed spacer 2, complete sequence; and 26S ribosomal RNA gene, partial sequence	1247	1247	95%	0.0	93%	
HM590372.1	Dendrobium aduncum voucher NCHU-D89331201-1006 18S ribosomal RNA gene, partial sequence; internal transcribed spacer 1, 5.8S ribosomal RNA gene, and internal transcribed spacer 2, complete sequence; and 26S ribosomal RNA gene, partial sequence	1245	1245	94%	0.0	93%	
HM590391.1	Dendrobium candidum voucher NCHU-D89331201-1028 18S ribosomal RNA gene, partial sequence; internal transcribed spacer 1, 5.8S ribosomal RNA gene, and internal transcribed spacer 2, complete sequence; and 26S ribosomal RNA gene, partial sequence	1171	1171	97%	0.0	91%	
HM590367.1	Dendrobium tosaense voucher NCHU-D89331201-1001 18S ribosomal RNA gene, partial sequence; internal transcribed spacer 1, 5.8S ribosomal RNA gene, and internal transcribed spacer 2, complete sequence; and 26S ribosomal RNA gene, partial sequence	1116	1116	94%	0.0	90%	
HM590373.1	Dendrobium leptocladum voucher NCHU-D89331201-1007 18S ribosomal RNA gene, partial sequence; internal transcribed spacer 1, 5.8S ribosomal RNA gene, and internal transcribed spacer 2, complete sequence; and 26S ribosomal RNA gene, partial sequence	1064	1064	98%	0.0	88%	
GU339111.1	Dendrobium moniliforme internal transcribed spacer 1, partial sequence; 5.8S ribosomal RNA gene, complete sequence; and internal transcribed spacer 2, partial sequence	946	946	70%	0.0	94%	
HM590378.1	Dendrobium parishii voucher NCHU-D89331201-1012 18S ribosomal RNA gene, partial sequence; internal transcribed spacer 1, 5.8S ribosomal RNA gene, and internal transcribed spacer 2, complete sequence; and 26S ribosomal RNA gene, partial sequence	924	924	86%	0.0	88%	
HM590369.1	Dendrobium moniliforme voucher NCHU-D89331201-1003 18S ribosomal RNA gene, partial sequence; internal transcribed spacer 1, 5.8S ribosomal RNA gene, and internal transcribed spacer 2, complete sequence; and 26S ribosomal RNA gene, partial sequence	924	924	86%	0.0	88%	
EU840696.1	Dendrobium huoshanense internal transcribed spacer 1, 5.8S ribosomal RNA gene, and internal transcribed spacer 2, complete sequence	924	924	70%	0.0	93%	
AF311777.1	Dendrobium moniliforme internal transcribed spacer 1, partial sequence; 5.8S ribosomal RNA gene, complete sequence; and internal transcribed spacer 2, partial sequence	922	922	70%	0.0	93%	
EU003114.1	Dendrobium moniliforme voucher CMC DM 0302 internal transcribed spacer 1, partial sequence; 5.8S ribosomal RNA gene, complete sequence; and internal transcribed spacer 2, partial sequence	918	918	70%	0.0	93%	

NCBI Blast:AY239972.1 Dendrobium indivisum - Mozilla Firefox

file:///G:/THESIS/NCBI DOWNLOADED MATK SEQUENCES/ITS/BLAST results 93+36 sequences/NCBI BlastAY239972_1 Dendrobium indivisum_htm.htm

Legend for links to other resources: UniGene GEO Gene Structure Map Viewer PubChem BioAssay

Sequences producing significant alignments:

Accession	Description	Max score	Total score	Query coverage	E value	Max ident	Links
AY239972.1	Dendrobium indivisum 18S ribosomal RNA gene, partial sequence; internal transcribed spacer 1, 5.8S ribosomal RNA gene and internal transcribed spacer 2, complete sequence; and 26S ribosomal RNA gene, partial sequence	1232	1232	100%	0.0	100%	
AY239951.1	Dendrobium aloifolium 18S ribosomal RNA gene, partial sequence; internal transcribed spacer 1, 5.8S ribosomal RNA gene and internal transcribed spacer 2, complete sequence; and 26S ribosomal RNA gene, partial sequence	1171	1171	100%	0.0	98%	
AY239953.1	Dendrobium anceps 18S ribosomal RNA gene, partial sequence; internal transcribed spacer 1, 5.8S ribosomal RNA gene and internal transcribed spacer 2, complete sequence; and 26S ribosomal RNA gene, partial sequence	1149	1149	100%	0.0	97%	
HM054548.1	Dendrobium anceps voucher SBB-0301 18S ribosomal RNA gene, partial sequence; internal transcribed spacer 1, 5.8S ribosomal RNA gene, and internal transcribed spacer 2, complete sequence; and 26S ribosomal RNA gene, partial sequence	1123	1123	97%	0.0	97%	
AY239961.1	Dendrobium confusum 18S ribosomal RNA gene, partial sequence; internal transcribed spacer 1, 5.8S ribosomal RNA gene and internal transcribed spacer 2, complete sequence; and 26S ribosomal RNA gene, partial sequence	1120	1120	100%	0.0	97%	
HM054547.1	Dendrobium anceps voucher SBB-0119 internal transcribed spacer 1, partial sequence; 5.8S ribosomal RNA gene, complete sequence; and internal transcribed spacer 2, partial sequence	1068	1068	92%	0.0	97%	
AY239978.1	Dendrobium leonis 18S ribosomal RNA gene, partial sequence; internal transcribed spacer 1, 5.8S ribosomal RNA gene and internal transcribed spacer 2, complete sequence; and 26S ribosomal RNA gene, partial sequence	1046	1046	100%	0.0	94%	
DQ058801.1	Dendrobium terminale voucher DY-ML03-1 internal transcribed spacer 1, 5.8S ribosomal RNA gene, and internal transcribed spacer 2, complete sequence	994	994	96%	0.0	94%	
AF362034.1	Dendrobium acinaciforme internal transcribed spacer 1, partial sequence; 5.8S ribosomal RNA gene, complete sequence; and internal transcribed spacer 2, partial sequence >gb|EF629321.1| Dendrobium jenkinsii internal transcribed spacer 1, partial sequence; 5.8S ribosomal RNA gene, complete sequence; and internal transcribed spacer 2, partial sequence	985	985	96%	0.0	94%	
AY239988.1	Dendrobium philippinense 18S ribosomal RNA gene, partial sequence; internal transcribed spacer 1, 5.8S ribosomal RNA gene and internal transcribed spacer 2, complete sequence; and 26S ribosomal RNA gene, partial sequence	948	948	100%	0.0	92%	
AY239975.1	Dendrobium junceum 18S ribosomal RNA gene, partial sequence; internal transcribed spacer 1, 5.8S ribosomal RNA gene and internal transcribed spacer 2, complete sequence; and 26S ribosomal RNA gene, partial sequence	939	939	100%	0.0	91%	
AY239969.1	Dendrobium goldfinchii 18S ribosomal RNA gene, partial sequence; internal transcribed spacer 1, 5.8S ribosomal RNA gene and internal transcribed spacer 2, complete sequence; and 26S ribosomal RNA gene, partial sequence	933	933	100%	0.0	92%	
HM590370.1	Dendrobium crumenatum voucher NCHU-D89331201-1004 18S ribosomal RNA gene, partial sequence; internal transcribed spacer 1, [illegible]	911	911	100%	0.0	91%	

NCBI Blast:AY239973.1 Dendrobium inflatum - Mozilla Firefox

file:///G:/THESIS/NCBI DOWNLOADED MATK SEQUENCES/ITS/BLAST results 93+36 sequences/NCBI BlastAY239973_1 Dendrobium inflatum_htm.htm

Legend for links to other resources: UniGene GEO Gene Structure Map Viewer PubChem BioAssay

Sequences producing significant alignments:

Accession	Description	Max score	Total score	Query coverage	E value	Max ident	Links
AY239973.1	Dendrobium inflatum 18S ribosomal RNA gene, partial sequence; internal transcribed spacer 1, 5.8S ribosomal RNA gene and internal transcribed spacer 2, complete sequence; and 26S ribosomal RNA gene, partial sequence	1214	1214	100%	0.0	100%	
AY239984.1	Dendrobium mutabile 18S ribosomal RNA gene, partial sequence; internal transcribed spacer 1, 5.8S ribosomal RNA gene and internal transcribed spacer 2, complete sequence; and 26S ribosomal RNA gene, partial sequence	1040	1040	100%	0.0	95%	
AY239992.1	Dendrobium sanguinolentum 18S ribosomal RNA gene, partial sequence; internal transcribed spacer 1, 5.8S ribosomal RNA gene and internal transcribed spacer 2, complete sequence; and 26S ribosomal RNA gene, partial sequence	1003	1003	100%	0.0	94%	
AY239974.1	Dendrobium ionopus 18S ribosomal RNA gene, partial sequence; internal transcribed spacer 1, 5.8S ribosomal RNA gene and internal transcribed spacer 2, complete sequence; and 26S ribosomal RNA gene, partial sequence	857	857	100%	0.0	90%	
AY239993.1	Dendrobium secundum 18S ribosomal RNA gene, partial sequence; internal transcribed spacer 1, 5.8S ribosomal RNA gene and internal transcribed spacer 2, complete sequence; and 26S ribosomal RNA gene, partial sequence	824	824	100%	0.0	89%	
AY239952.1	Dendrobium amethystoglossum 18S ribosomal RNA gene, partial sequence; internal transcribed spacer 1, 5.8S ribosomal RNA gene and internal transcribed spacer 2, complete sequence; and 26S ribosomal RNA gene, partial sequence	817	817	100%	0.0	89%	
AY239959.1	Dendrobium cerinum 18S ribosomal RNA gene, partial sequence; internal transcribed spacer 1, 5.8S ribosomal RNA gene and internal transcribed spacer 2, complete sequence; and 26S ribosomal RNA gene, partial sequence	813	813	100%	0.0	89%	
AY239962.1	Dendrobium aff. crocatum Smedley s.n. 18S ribosomal RNA gene, partial sequence; internal transcribed spacer 1, 5.8S ribosomal RNA gene and internal transcribed spacer 2, complete sequence; and 26S ribosomal RNA gene, partial sequence	784	784	100%	0.0	88%	
AY239955.1	Dendrobium camptocentrum 18S ribosomal RNA gene, partial sequence; internal transcribed spacer 1, 5.8S ribosomal RNA gene and internal transcribed spacer 2, complete sequence; and 26S ribosomal RNA gene, partial sequence	767	767	100%	0.0	88%	
AY239967.1	Dendrobium formosum 18S ribosomal RNA gene, partial sequence; internal transcribed spacer 1, 5.8S ribosomal RNA gene and internal transcribed spacer 2, complete sequence; and 26S ribosomal RNA gene, partial sequence	701	701	100%	0.0	86%	
AY240001.1	Dendrobium thyrsiflorum 18S ribosomal RNA gene, partial sequence; internal transcribed spacer 1, 5.8S ribosomal RNA gene and internal transcribed spacer 2, complete sequence; and 26S ribosomal RNA gene, partial sequence	688	688	100%	0.0	85%	
HM054758.1	Dendrobium thyrsiflorum voucher SBB-0518 internal transcribed spacer 1, partial sequence; 5.8S ribosomal RNA gene and internal transcribed spacer 2, complete sequence; and 26S ribosomal RNA gene, partial sequence	680	680	98%	0.0	85%	
HM054626.1	Dendrobium densiflorum voucher SBB-0120 internal transcribed spacer 1, partial sequence; 5.8S ribosomal RNA gene and internal transcribed spacer 2, complete sequence; and 26S ribosomal RNA gene, partial sequence	678	678	97%	0.0	85%	
	Dendrobium christyanum internal transcribed spacer 1, partial sequence; 5.8S ribosomal RNA gene, complete sequence; and internal [illegible]						

NCBI Blast:AY239974.1 Dendrobium ionopus - Mozilla Firefox

file:///G:/THESIS/NCBI DOWNLOADED MATK SEQUENCES/ITS/BLAST results 93+36 sequences/NCBI BlastAY239974_1 Dendrobium ionopus_htm.htm

Legend for links to other resources: UniGene GEO Gene Structure Map Viewer PubChem BioAssay

Sequences producing significant alignments:

Accession	Description	Max score	Total score	Query coverage	E value	Max ident	Links
AY239974.1	Dendrobium ionopus 18S ribosomal RNA gene, partial sequence; internal transcribed spacer 1, 5.8S ribosomal RNA gene and internal transcribed spacer 2, complete sequence; and 26S ribosomal RNA gene, partial sequence	1210	1210	100%	0.0	100%	
AY239959.1	Dendrobium cerinum 18S ribosomal RNA gene, partial sequence; internal transcribed spacer 1, 5.8S ribosomal RNA gene and internal transcribed spacer 2, complete sequence; and 26S ribosomal RNA gene, partial sequence	1116	1116	100%	0.0	97%	
AY239962.1	Dendrobium aff. crocatum Smedley s.n. 18S ribosomal RNA gene, partial sequence; internal transcribed spacer 1, 5.8S ribosomal RNA gene and internal transcribed spacer 2, complete sequence; and 26S ribosomal RNA gene, partial sequence	1031	1031	100%	0.0	95%	
AY239955.1	Dendrobium camptocentrum 18S ribosomal RNA gene, partial sequence; internal transcribed spacer 1, 5.8S ribosomal RNA gene and internal transcribed spacer 2, complete sequence; and 26S ribosomal RNA gene, partial sequence	976	976	100%	0.0	93%	
AY239993.1	Dendrobium secundum 18S ribosomal RNA gene, partial sequence; internal transcribed spacer 1, 5.8S ribosomal RNA gene and internal transcribed spacer 2, complete sequence; and 26S ribosomal RNA gene, partial sequence	928	928	100%	0.0	92%	
AY239984.1	Dendrobium mutabile 18S ribosomal RNA gene, partial sequence; internal transcribed spacer 1, 5.8S ribosomal RNA gene and internal transcribed spacer 2, complete sequence; and 26S ribosomal RNA gene, partial sequence	861	861	100%	0.0	90%	
AY239952.1	Dendrobium amethystoglossum 18S ribosomal RNA gene, partial sequence; internal transcribed spacer 1, 5.8S ribosomal RNA gene and internal transcribed spacer 2, complete sequence; and 26S ribosomal RNA gene, partial sequence	859	859	100%	0.0	90%	
AY239973.1	Dendrobium inflatum 18S ribosomal RNA gene, partial sequence; internal transcribed spacer 1, 5.8S ribosomal RNA gene and internal transcribed spacer 2, complete sequence; and 26S ribosomal RNA gene, partial sequence	857	857	100%	0.0	90%	
AY239992.1	Dendrobium sanguinolentum 18S ribosomal RNA gene, partial sequence; internal transcribed spacer 1, 5.8S ribosomal RNA gene and internal transcribed spacer 2, complete sequence; and 26S ribosomal RNA gene, partial sequence	852	852	100%	0.0	90%	
AY239967.1	Dendrobium formosum 18S ribosomal RNA gene, partial sequence; internal transcribed spacer 1, 5.8S ribosomal RNA gene and internal transcribed spacer 2, complete sequence; and 26S ribosomal RNA gene, partial sequence	726	726	100%	0.0	86%	
GU339106.1	Dendrobium christyanum internal transcribed spacer 1, partial sequence; 5.8S ribosomal RNA gene, complete sequence; and internal transcribed spacer 2, partial sequence	725	725	90%	0.0	88%	
AY240001.1	Dendrobium thyrsiflorum 18S ribosomal RNA gene, partial sequence; internal transcribed spacer 1, 5.8S ribosomal RNA gene and internal transcribed spacer 2, complete sequence; and 26S ribosomal RNA gene, partial sequence	725	725	100%	0.0	86%	
HM054758.1	Dendrobium thyrsiflorum voucher SBB-0518 internal transcribed spacer 1, partial sequence; 5.8S ribosomal RNA gene and internal transcribed spacer 2, complete sequence; and 26S ribosomal RNA gene, partial sequence	723	723	98%	0.0	86%	
	[illegible]						

NCBI Blast:EU430383.1 Dendrobium jonesii - Mozilla Firefox

file:///G:/THESIS/NCBI DOWNLOADED MATK SEQUENCES/ITS/BLAST results 93+36 sequences/NCBI BlastEU430383_1 Dendrobium jonesii_htm.htm

Legend for links to other resources: UniGene GEO Gene Structure Map Viewer PubChem BioAssay

Sequences producing significant alignments:

Accession	Description	Max score	Total score	Query coverage	E value	Max ident	Links
EU430383.1	Dendrobium jonesii var. magnificum 18S ribosomal RNA gene, partial sequence; internal transcribed spacer 1, 5.8S ribosomal RNA gene, and internal transcribed spacer 2, complete sequence; and 26S ribosomal RNA gene, partial sequence	1339	1339	100%	0.0	100%	
EU430384.1	Dendrobium kingianum subsp. carnarvonense 18S ribosomal RNA gene, partial sequence; internal transcribed spacer 1, 5.8S ribosomal RNA gene, and internal transcribed spacer 2, complete sequence; and 26S ribosomal RNA gene, partial sequence	1232	1232	100%	0.0	97%	
EU430382.1	Dendrobium gracilicaule 18S ribosomal RNA gene, partial sequence; internal transcribed spacer 1, 5.8S ribosomal RNA gene, and internal transcribed spacer 2, complete sequence; and 26S ribosomal RNA gene, partial sequence	1221	1221	98%	0.0	97%	
EU430395.1	Dendrobium speciosum var. curvicaule 18S ribosomal RNA gene, partial sequence; internal transcribed spacer 1, 5.8S ribosomal RNA gene, and internal transcribed spacer 2, complete sequence; and 26S ribosomal RNA gene, partial sequence	1218	1218	100%	0.0	96%	
EU430398.1	Dendrobium speciosum var. pedunculatum 18S ribosomal RNA gene, partial sequence; internal transcribed spacer 1, 5.8S ribosomal RNA gene, and internal transcribed spacer 2, complete sequence; and 26S ribosomal RNA gene, partial sequence	1190	1190	98%	0.0	96%	
EU430385.1	Dendrobium kingianum var. pulcherrimum 18S ribosomal RNA gene, partial sequence; internal transcribed spacer 1, 5.8S ribosomal RNA gene, and internal transcribed spacer 2, complete sequence; and 26S ribosomal RNA gene, partial sequence	1186	1186	100%	0.0	96%	
AF321594.1	Dockrillia pugioniformis internal transcribed spacer 1, 5.8S ribosomal RNA gene, and internal transcribed spacer 2, complete sequence	1181	1181	100%	0.0	96%	
EU430380.1	Dendrobium fleckeri 18S ribosomal RNA gene, partial sequence; internal transcribed spacer 1, 5.8S ribosomal RNA gene, and internal transcribed spacer 2, complete sequence; and 26S ribosomal RNA gene, partial sequence	1166	1166	93%	0.0	97%	
EU430397.1	Dendrobium speciosum var. hillii 18S ribosomal RNA gene, partial sequence; internal transcribed spacer 1, 5.8S ribosomal RNA gene, and internal transcribed spacer 2, complete sequence; and 26S ribosomal RNA gene, partial sequence	1158	1158	100%	0.0	95%	
EU430391.1	Dendrobium speciosum var. blackdownense 18S ribosomal RNA gene, partial sequence; internal transcribed spacer 1, 5.8S ribosomal RNA gene, and internal transcribed spacer 2, complete sequence; and 26S ribosomal RNA gene, partial sequence	1158	1158	100%	0.0	95%	
AF321593.1	Dockrillia linguiforme internal transcribed spacer 1, 5.8S ribosomal RNA gene, and internal transcribed spacer 2, complete sequence	1158	1158	100%	0.0	95%	
EU430396.1	Dendrobium speciosum var. grandiflorum 18S ribosomal RNA gene, partial sequence; internal transcribed spacer 1, 5.8S ribosomal RNA gene, and internal transcribed spacer 2, complete sequence; and 26S ribosomal RNA gene, partial sequence	1155	1155	99%	0.0	95%	
EU430394.1	Dendrobium speciosum var. carnarvonense 18S ribosomal RNA gene, partial sequence; internal transcribed spacer 1, 5.8S ribosomal RNA gene, and internal transcribed spacer 2, complete sequence; and 26S ribosomal RNA gene, partial sequence	1155	1155	99%	0.0	95%	
EU430393.1	Dendrobium speciosum var. capricornicum 18S ribosomal RNA gene, partial sequence; internal transcribed spacer 1, 5.8S ribosomal [illegible]	1155	1155	100%	0.0	95%	

NCBI Blast:AY239975.1 Dendrobium junceum - Mozilla Firefox

file:///G:/THESIS/NCBI DOWNLOADED MATK SEQUENCES/ITS/BLAST results 93+36 sequences/NCBI BlastAY239975_1 Dendrobium junceum_htm.htm

Legend for links to other resources: UniGene GEO Gene Structure Map Viewer PubChem BioAssay

Sequences producing significant alignments:

Accession	Description	Max score	Total score	Query coverage	E value	Max ident	Links
AY239975.1	Dendrobium junceum 18S ribosomal RNA gene, partial sequence; internal transcribed spacer 1, 5.8S ribosomal RNA gene and internal transcribed spacer 2, complete sequence; and 26S ribosomal RNA gene, partial sequence	1254	1254	100%	0.0	100%	
AY239988.1	Dendrobium philippinense 18S ribosomal RNA gene, partial sequence; internal transcribed spacer 1, 5.8S ribosomal RNA gene and internal transcribed spacer 2, complete sequence; and 26S ribosomal RNA gene, partial sequence	981	981	100%	0.0	93%	
AY239969.1	Dendrobium goldfinchii 18S ribosomal RNA gene, partial sequence; internal transcribed spacer 1, 5.8S ribosomal RNA gene and internal transcribed spacer 2, complete sequence; and 26S ribosomal RNA gene, partial sequence	977	977	100%	0.0	93%	
AF521609.1	Dendrobium equitans internal transcribed spacer 1, 5.8S ribosomal RNA gene, and internal transcribed spacer 2, complete sequence >gb|EU840701.1| Dendrobium equitans internal transcribed spacer 1, 5.8S ribosomal RNA gene, and internal transcribed spacer 2, complete sequence	976	976	96%	0.0	93%	
AY240003.1	Dendrobium usterioides 18S ribosomal RNA gene, partial sequence; internal transcribed spacer 1, 5.8S ribosomal RNA gene and internal transcribed spacer 2, complete sequence; and 26S ribosomal RNA gene, partial sequence	974	974	100%	0.0	92%	
AY239961.1	Dendrobium confusum 18S ribosomal RNA gene, partial sequence; internal transcribed spacer 1, 5.8S ribosomal RNA gene and internal transcribed spacer 2, complete sequence; and 26S ribosomal RNA gene, partial sequence	948	948	100%	0.0	92%	
AY239951.1	Dendrobium aloifolium 18S ribosomal RNA gene, partial sequence; internal transcribed spacer 1, 5.8S ribosomal RNA gene and internal transcribed spacer 2, complete sequence; and 26S ribosomal RNA gene, partial sequence	944	944	100%	0.0	92%	
AY239972.1	Dendrobium indivisum 18S ribosomal RNA gene, partial sequence; internal transcribed spacer 1, 5.8S ribosomal RNA gene and internal transcribed spacer 2, complete sequence; and 26S ribosomal RNA gene, partial sequence	939	939	100%	0.0	91%	
AY239953.1	Dendrobium anceps 18S ribosomal RNA gene, partial sequence; internal transcribed spacer 1, 5.8S ribosomal RNA gene and internal transcribed spacer 2, complete sequence; and 26S ribosomal RNA gene, partial sequence	928	928	100%	0.0	91%	
AY239989.1	Dendrobium quadrangulare 18S ribosomal RNA gene, partial sequence; internal transcribed spacer 1, 5.8S ribosomal RNA gene and internal transcribed spacer 2, complete sequence; and 26S ribosomal RNA gene, partial sequence	926	926	100%	0.0	91%	
HM054548.1	Dendrobium anceps voucher SBB-0301 18S ribosomal RNA gene, partial sequence; internal transcribed spacer 1, 5.8S ribosomal RNA gene, and internal transcribed spacer 2, complete sequence; and 26S ribosomal RNA gene, partial sequence	913	913	97%	0.0	91%	
AY239978.1	Dendrobium leonis 18S ribosomal RNA gene, partial sequence; internal transcribed spacer 1, 5.8S ribosomal RNA gene and internal transcribed spacer 2, complete sequence; and 26S ribosomal RNA gene, partial sequence	902	902	100%	0.0	90%	
EU840698.1	Dendrobium quadrangulare internal transcribed spacer 1, 5.8S ribosomal RNA gene, and internal transcribed spacer 2, complete sequence	900	900	96%	0.0	91%	
[illegible]	Dendrobium crumenatum voucher NCHU-D89331201-1004 18S ribosomal RNA gene, partial sequence; internal transcribed spacer 1 [illegible]	[illegible]	[illegible]	[illegible]	[illegible]	[illegible]	

NCBI Blast:EU430385.1 Dendrobium kingianum - Mozilla Firefox

Legend for links to other resources: UniGene GEO Gene S Structure Map Viewer PubChem BioAssay

Sequences producing significant alignments:

Accession	Description	Max score	Total score	Query coverage	E value	Max ident	Links
EU430385.1	Dendrobium kingianum var. pulcherrimum 18S ribosomal RNA gene, partial sequence; internal transcribed spacer 1, 5.8S ribosomal RNA gene, and internal transcribed spacer 2, complete sequence; and 26S ribosomal RNA gene, partial sequence	1371	1371	100%	0.0	100%	
EU430384.1	Dendrobium kingianum subsp. carnarvonense 18S ribosomal RNA gene, partial sequence; internal transcribed spacer 1, 5.8S ribosomal RNA gene, and internal transcribed spacer 2, complete sequence; and 26S ribosomal RNA gene, partial sequence	1271	1271	98%	0.0	97%	
EU430386.1	Dendrobium kingianum var. kingianum 18S ribosomal RNA gene, partial sequence; internal transcribed spacer 1, 5.8S ribosomal RNA gene, and internal transcribed spacer 2, complete sequence; and 26S ribosomal RNA gene, partial sequence	1234	1234	96%	0.0	97%	
EU430395.1	Dendrobium speciosum var. curvicaule 18S ribosomal RNA gene, partial sequence; internal transcribed spacer 1, 5.8S ribosomal RNA gene, and internal transcribed spacer 2, complete sequence; and 26S ribosomal RNA gene, partial sequence	1206	1206	98%	0.0	96%	
EU430398.1	Dendrobium speciosum var. pedunculatum 18S ribosomal RNA gene, partial sequence; internal transcribed spacer 1, 5.8S ribosomal RNA gene, and internal transcribed spacer 2, complete sequence; and 26S ribosomal RNA gene, partial sequence	1201	1201	97%	0.0	96%	
EU430382.1	Dendrobium gracilicaule 18S ribosomal RNA gene, partial sequence; internal transcribed spacer 1, 5.8S ribosomal RNA gene, and internal transcribed spacer 2, complete sequence; and 26S ribosomal RNA gene, partial sequence	1188	1188	96%	0.0	96%	
EU430383.1	Dendrobium jonesii var. magnificum 18S ribosomal RNA gene, partial sequence; internal transcribed spacer 1, 5.8S ribosomal RNA gene, and internal transcribed spacer 2, complete sequence; and 26S ribosomal RNA gene, partial sequence	1186	1186	97%	0.0	96%	
EU430393.1	Dendrobium speciosum var. capricornicum 18S ribosomal RNA gene, partial sequence; internal transcribed spacer 1, 5.8S ribosomal RNA gene, and internal transcribed spacer 2, complete sequence; and 26S ribosomal RNA gene, partial sequence	1171	1171	99%	0.0	95%	
EU430391.1	Dendrobium speciosum var. blackdownense 18S ribosomal RNA gene, partial sequence; internal transcribed spacer 1, 5.8S ribosomal RNA gene, and internal transcribed spacer 2, complete sequence; and 26S ribosomal RNA gene, partial sequence	1162	1162	98%	0.0	95%	
EU430397.1	Dendrobium speciosum var. hillii 18S ribosomal RNA gene, partial sequence; internal transcribed spacer 1, 5.8S ribosomal RNA gene, and internal transcribed spacer 2, complete sequence; and 26S ribosomal RNA gene, partial sequence	1160	1160	98%	0.0	95%	
EU430396.1	Dendrobium speciosum var. grandiflorum 18S ribosomal RNA gene, partial sequence; internal transcribed spacer 1, 5.8S ribosomal RNA gene, and internal transcribed spacer 2, complete sequence; and 26S ribosomal RNA gene, partial sequence	1160	1160	98%	0.0	95%	
EU430392.1	Dendrobium speciosum var. boreale 18S ribosomal RNA gene, partial sequence; internal transcribed spacer 1, 5.8S ribosomal RNA gene, and internal transcribed spacer 2, complete sequence; and 26S ribosomal RNA gene, partial sequence	1160	1160	93%	0.0	96%	
EU430388.1	Dendrobium moorei 18S ribosomal RNA gene, partial sequence; internal transcribed spacer 1 and 5.8S ribosomal RNA gene, complete sequence; and internal transcribed spacer 2, partial sequence	1157	1157	90%	0.0	97%	

NCBI Blast:AY239976.1 Dendrobium lancifolium - Mozilla Firefox

Legend for links to other resources: UniGene GEO Gene S Structure Map Viewer PubChem BioAssay

Sequences producing significant alignments:

Accession	Description	Max score	Total score	Query coverage	E value	Max ident	Links
AY239976.1	Dendrobium lancifolium 18S ribosomal RNA gene, partial sequence; internal transcribed spacer 1, 5.8S ribosomal RNA gene and internal transcribed spacer 2, complete sequence; and 26S ribosomal RNA gene, partial sequence	1225	1225	100%	0.0	100%	
AY239994.1	Dendrobium serratilabium 18S ribosomal RNA gene, partial sequence; internal transcribed spacer 1, 5.8S ribosomal RNA gene and internal transcribed spacer 2, complete sequence; and 26S ribosomal RNA gene, partial sequence	998	998	100%	0.0	93%	
AY239960.1	Dendrobium chameleon 18S ribosomal RNA gene, partial sequence; internal transcribed spacer 1, 5.8S ribosomal RNA gene and internal transcribed spacer 2, complete sequence; and 26S ribosomal RNA gene, partial sequence	998	998	100%	0.0	93%	
AY240006.1	Dendrobium yeageri 18S ribosomal RNA gene, partial sequence; internal transcribed spacer 1, 5.8S ribosomal RNA gene and internal transcribed spacer 2, complete sequence; and 26S ribosomal RNA gene, partial sequence	992	992	100%	0.0	93%	
AY239958.1	Dendrobium ceraula 18S ribosomal RNA gene, partial sequence; internal transcribed spacer 1, 5.8S ribosomal RNA gene and internal transcribed spacer 2, complete sequence; and 26S ribosomal RNA gene, partial sequence	990	990	100%	0.0	93%	
AY240004.1	Dendrobium victoriae-reginae 18S ribosomal RNA gene, partial sequence; internal transcribed spacer 1, 5.8S ribosomal RNA gene and internal transcribed spacer 2, complete sequence; and 26S ribosomal RNA gene, partial sequence	985	985	100%	0.0	93%	
HM590385.1	Dendrobium chameleon voucher NCHU-D89331201-1019 18S ribosomal RNA gene, partial sequence; internal transcribed spacer 1, 5.8S ribosomal RNA gene, and internal transcribed spacer 2, complete sequence; and 26S ribosomal RNA gene, partial sequence	981	981	100%	0.0	93%	
AF521607.1	Dendrobium chameleon internal transcribed spacer 1, 5.8S ribosomal RNA gene, and internal transcribed spacer 2, complete sequence	942	942	96%	0.0	93%	
HM590386.1	Dendrobium miyakei voucher NCHU-D89331201-1020 18S ribosomal RNA gene, partial sequence; internal transcribed spacer 1, 5.8S ribosomal RNA gene, and internal transcribed spacer 2, complete sequence; and 26S ribosomal RNA gene, partial sequence	933	933	100%	0.0	92%	
AY239970.1	Dendrobium goldschmidtianum 18S ribosomal RNA gene, partial sequence; internal transcribed spacer 1, 5.8S ribosomal RNA gene and internal transcribed spacer 2, complete sequence; and 26S ribosomal RNA gene, partial sequence	933	933	100%	0.0	92%	
AY239980.1	Dendrobium mohlianum 18S ribosomal RNA gene, partial sequence; internal transcribed spacer 1, 5.8S ribosomal RNA gene and internal transcribed spacer 2, complete sequence; and 26S ribosomal RNA gene, partial sequence	926	926	100%	0.0	91%	
AY239968.1	Dendrobium fulgidum 18S ribosomal RNA gene, partial sequence; internal transcribed spacer 1, 5.8S ribosomal RNA gene and internal transcribed spacer 2, complete sequence; and 26S ribosomal RNA gene, partial sequence	920	920	100%	0.0	91%	
AY239986.1	Dendrobium nothofagicola 18S ribosomal RNA gene, partial sequence; internal transcribed spacer 1, 5.8S ribosomal RNA gene and internal transcribed spacer 2, complete sequence; and 26S ribosomal RNA gene, partial sequence	907	907	100%	0.0	91%	
AY239957.1	Dendrobium caluculimentum 18S ribosomal RNA gene, partial sequence; internal transcribed spacer 1, 5.8S ribosomal RNA gene and [illegible]	904	904	100%	0.0	91%	

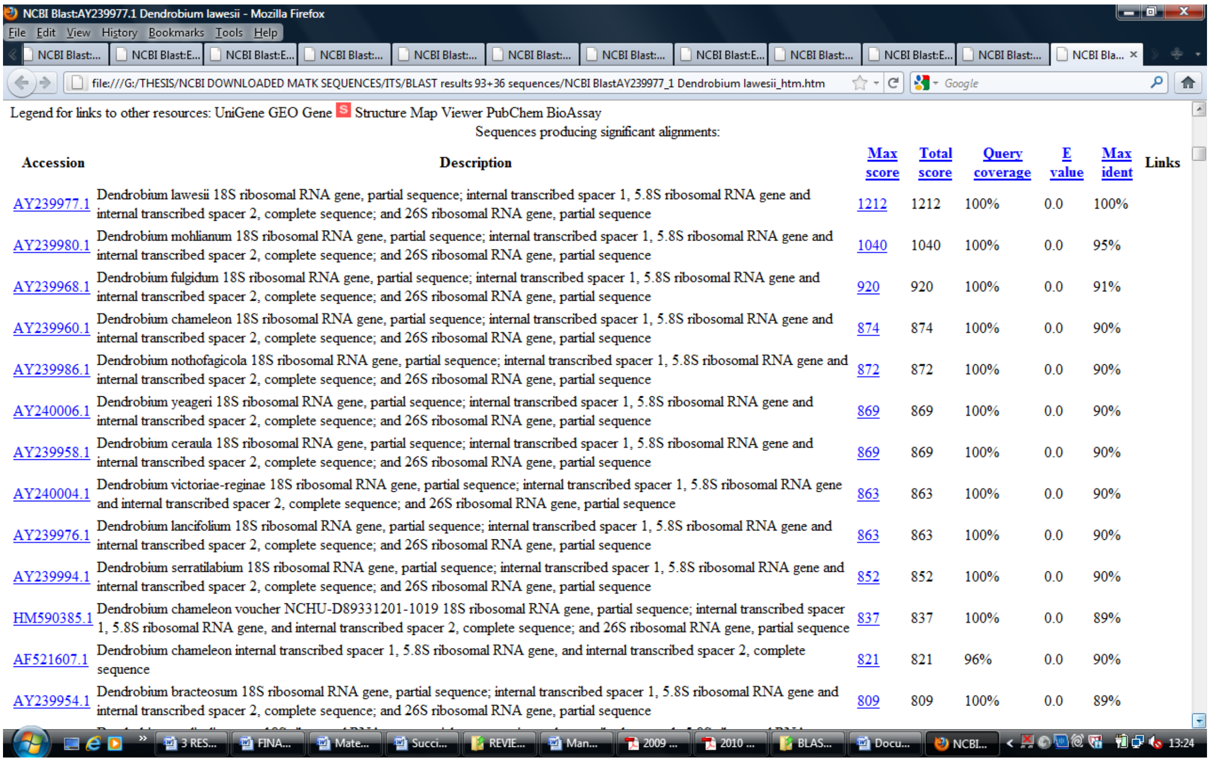

NCBI Blast:AY239977.1 Dendrobium lawesii - Mozilla Firefox

file:///G:/THESIS/NCBI DOWNLOADED MATK SEQUENCES/ITS/BLAST results 93+36 sequences/NCBI BlastAY239977_1 Dendrobium lawesii_htm.htm

Legend for links to other resources: UniGene GEO Gene Structure Map Viewer PubChem BioAssay

Sequences producing significant alignments:

Accession	Description	Max score	Total score	Query coverage	E value	Max ident	Links
AY239977.1	Dendrobium lawesii 18S ribosomal RNA gene, partial sequence; internal transcribed spacer 1, 5.8S ribosomal RNA gene and internal transcribed spacer 2, complete sequence; and 26S ribosomal RNA gene, partial sequence	1212	1212	100%	0.0	100%	
AY239980.1	Dendrobium mohlianum 18S ribosomal RNA gene, partial sequence; internal transcribed spacer 1, 5.8S ribosomal RNA gene and internal transcribed spacer 2, complete sequence; and 26S ribosomal RNA gene, partial sequence	1040	1040	100%	0.0	95%	
AY239968.1	Dendrobium fulgidum 18S ribosomal RNA gene, partial sequence; internal transcribed spacer 1, 5.8S ribosomal RNA gene and internal transcribed spacer 2, complete sequence; and 26S ribosomal RNA gene, partial sequence	920	920	100%	0.0	91%	
AY239960.1	Dendrobium chameleon 18S ribosomal RNA gene, partial sequence; internal transcribed spacer 1, 5.8S ribosomal RNA gene and internal transcribed spacer 2, complete sequence; and 26S ribosomal RNA gene, partial sequence	874	874	100%	0.0	90%	
AY239986.1	Dendrobium nothofagicola 18S ribosomal RNA gene, partial sequence; internal transcribed spacer 1, 5.8S ribosomal RNA gene and internal transcribed spacer 2, complete sequence; and 26S ribosomal RNA gene, partial sequence	872	872	100%	0.0	90%	
AY240006.1	Dendrobium yeageri 18S ribosomal RNA gene, partial sequence; internal transcribed spacer 1, 5.8S ribosomal RNA gene and internal transcribed spacer 2, complete sequence; and 26S ribosomal RNA gene, partial sequence	869	869	100%	0.0	90%	
AY239958.1	Dendrobium ceraula 18S ribosomal RNA gene, partial sequence; internal transcribed spacer 1, 5.8S ribosomal RNA gene and internal transcribed spacer 2, complete sequence; and 26S ribosomal RNA gene, partial sequence	869	869	100%	0.0	90%	
AY240004.1	Dendrobium victoriae-reginae 18S ribosomal RNA gene, partial sequence; internal transcribed spacer 1, 5.8S ribosomal RNA gene and internal transcribed spacer 2, complete sequence; and 26S ribosomal RNA gene, partial sequence	863	863	100%	0.0	90%	
AY239976.1	Dendrobium lancifolium 18S ribosomal RNA gene, partial sequence; internal transcribed spacer 1, 5.8S ribosomal RNA gene and internal transcribed spacer 2, complete sequence; and 26S ribosomal RNA gene, partial sequence	863	863	100%	0.0	90%	
AY239994.1	Dendrobium serratilabium 18S ribosomal RNA gene, partial sequence; internal transcribed spacer 1, 5.8S ribosomal RNA gene and internal transcribed spacer 2, complete sequence; and 26S ribosomal RNA gene, partial sequence	852	852	100%	0.0	90%	
HM590385.1	Dendrobium chameleon voucher NCHU-D89331201-1019 18S ribosomal RNA gene, partial sequence; internal transcribed spacer 1, 5.8S ribosomal RNA gene, and internal transcribed spacer 2, complete sequence; and 26S ribosomal RNA gene, partial sequence	837	837	100%	0.0	89%	
AF521607.1	Dendrobium chameleon internal transcribed spacer 1, 5.8S ribosomal RNA gene, and internal transcribed spacer 2, complete sequence	821	821	96%	0.0	90%	
AY239954.1	Dendrobium bracteosum 18S ribosomal RNA gene, partial sequence; internal transcribed spacer 1, 5.8S ribosomal RNA gene and internal transcribed spacer 2, complete sequence; and 26S ribosomal RNA gene, partial sequence	809	809	100%	0.0	89%	
[illegible]	[illegible]						

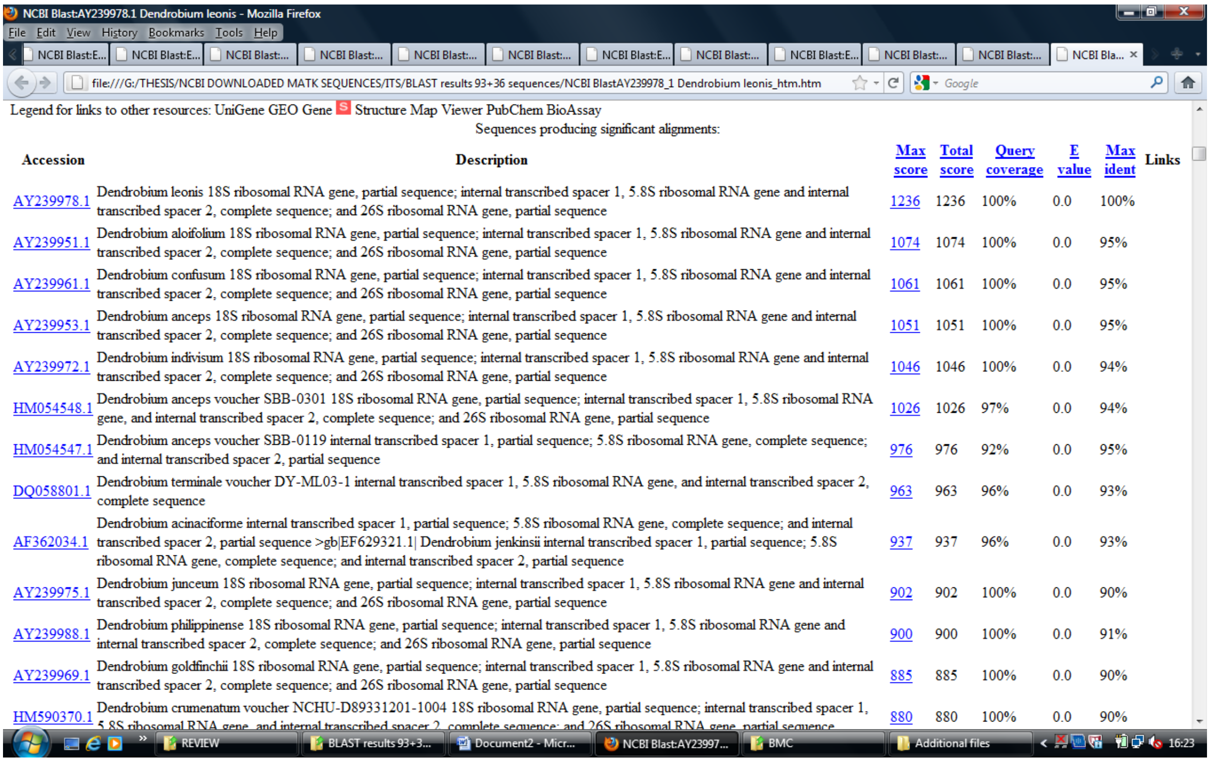

NCBI Blast:AY239978.1 Dendrobium leonis - Mozilla Firefox

file:///G:/THESIS/NCBI DOWNLOADED MATK SEQUENCES/ITS/BLAST results 93+36 sequences/NCBI BlastAY239978_1 Dendrobium leonis_htm.htm

Legend for links to other resources: UniGene GEO Gene Structure Map Viewer PubChem BioAssay

Sequences producing significant alignments:

Accession	Description	Max score	Total score	Query coverage	E value	Max ident	Links
AY239978.1	Dendrobium leonis 18S ribosomal RNA gene, partial sequence; internal transcribed spacer 1, 5.8S ribosomal RNA gene and internal transcribed spacer 2, complete sequence; and 26S ribosomal RNA gene, partial sequence	1236	1236	100%	0.0	100%	
AY239951.1	Dendrobium aloifolium 18S ribosomal RNA gene, partial sequence; internal transcribed spacer 1, 5.8S ribosomal RNA gene and internal transcribed spacer 2, complete sequence; and 26S ribosomal RNA gene, partial sequence	1074	1074	100%	0.0	95%	
AY239961.1	Dendrobium confusum 18S ribosomal RNA gene, partial sequence; internal transcribed spacer 1, 5.8S ribosomal RNA gene and internal transcribed spacer 2, complete sequence; and 26S ribosomal RNA gene, partial sequence	1061	1061	100%	0.0	95%	
AY239953.1	Dendrobium anceps 18S ribosomal RNA gene, partial sequence; internal transcribed spacer 1, 5.8S ribosomal RNA gene and internal transcribed spacer 2, complete sequence; and 26S ribosomal RNA gene, partial sequence	1051	1051	100%	0.0	95%	
AY239972.1	Dendrobium indivisum 18S ribosomal RNA gene, partial sequence; internal transcribed spacer 1, 5.8S ribosomal RNA gene and internal transcribed spacer 2, complete sequence; and 26S ribosomal RNA gene, partial sequence	1046	1046	100%	0.0	94%	
HM054548.1	Dendrobium anceps voucher SBB-0301 18S ribosomal RNA gene, partial sequence; internal transcribed spacer 1, 5.8S ribosomal RNA gene, and internal transcribed spacer 2, complete sequence; and 26S ribosomal RNA gene, partial sequence	1026	1026	97%	0.0	94%	
HM054547.1	Dendrobium anceps voucher SBB-0119 internal transcribed spacer 1, partial sequence; 5.8S ribosomal RNA gene, complete sequence; and internal transcribed spacer 2, partial sequence	976	976	92%	0.0	95%	
DQ058801.1	Dendrobium terminale voucher DY-ML03-1 internal transcribed spacer 1, 5.8S ribosomal RNA gene, and internal transcribed spacer 2, complete sequence	963	963	96%	0.0	93%	
AF362034.1	Dendrobium acinaciforme internal transcribed spacer 1, partial sequence; 5.8S ribosomal RNA gene, complete sequence; and internal transcribed spacer 2, partial sequence >gb|EF629321.1| Dendrobium jenkinsii internal transcribed spacer 1, partial sequence; 5.8S ribosomal RNA gene, complete sequence; and internal transcribed spacer 2, partial sequence	937	937	96%	0.0	93%	
AY239975.1	Dendrobium junceum 18S ribosomal RNA gene, partial sequence; internal transcribed spacer 1, 5.8S ribosomal RNA gene and internal transcribed spacer 2, complete sequence; and 26S ribosomal RNA gene, partial sequence	902	902	100%	0.0	90%	
AY239988.1	Dendrobium philippinense 18S ribosomal RNA gene, partial sequence; internal transcribed spacer 1, 5.8S ribosomal RNA gene and internal transcribed spacer 2, complete sequence; and 26S ribosomal RNA gene, partial sequence	900	900	100%	0.0	91%	
AY239969.1	Dendrobium goldfinchii 18S ribosomal RNA gene, partial sequence; internal transcribed spacer 1, 5.8S ribosomal RNA gene and internal transcribed spacer 2, complete sequence; and 26S ribosomal RNA gene, partial sequence	885	885	100%	0.0	90%	
HM590370.1	Dendrobium crumenatum voucher NCHU-D89331201-1004 18S ribosomal RNA gene, partial sequence; internal transcribed spacer 1, 5.8S ribosomal RNA gene, and internal transcribed spacer 2, complete sequence; and 26S ribosomal RNA gene, partial sequence	880	880	100%	0.0	90%	

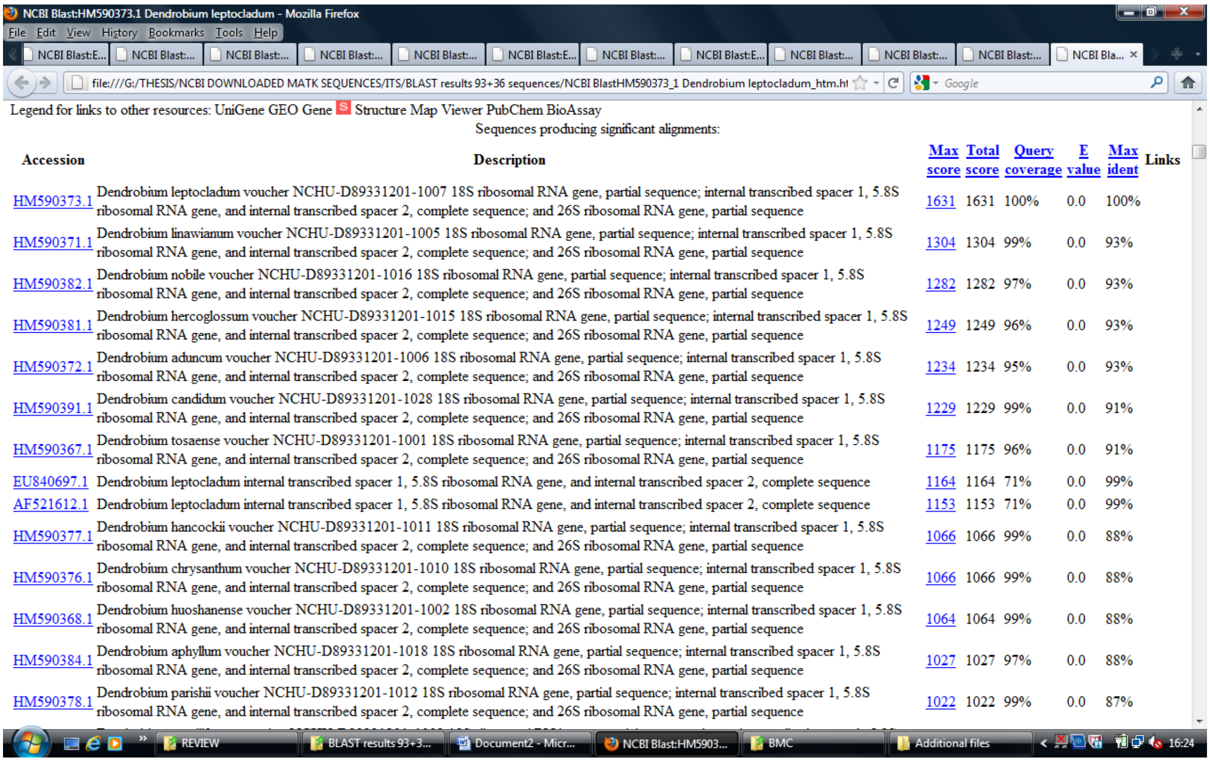

NCBI Blast:HM590373.1 Dendrobium leptocladum - Mozilla Firefox

Legend for links to other resources: UniGene GEO Gene Structure Map Viewer PubChem BioAssay

Sequences producing significant alignments:

Accession	Description	Max score	Total score	Query coverage	E value	Max ident	Links
HM590373.1	Dendrobium leptocladum voucher NCHU-D89331201-1007 18S ribosomal RNA gene, partial sequence; internal transcribed spacer 1, 5.8S ribosomal RNA gene, and internal transcribed spacer 2, complete sequence; and 26S ribosomal RNA gene, partial sequence	1631	1631	100%	0.0	100%	
HM590371.1	Dendrobium linawianum voucher NCHU-D89331201-1005 18S ribosomal RNA gene, partial sequence; internal transcribed spacer 1, 5.8S ribosomal RNA gene, and internal transcribed spacer 2, complete sequence; and 26S ribosomal RNA gene, partial sequence	1304	1304	99%	0.0	93%	
HM590382.1	Dendrobium nobile voucher NCHU-D89331201-1016 18S ribosomal RNA gene, partial sequence; internal transcribed spacer 1, 5.8S ribosomal RNA gene, and internal transcribed spacer 2, complete sequence; and 26S ribosomal RNA gene, partial sequence	1282	1282	97%	0.0	93%	
HM590381.1	Dendrobium hercoglossum voucher NCHU-D89331201-1015 18S ribosomal RNA gene, partial sequence; internal transcribed spacer 1, 5.8S ribosomal RNA gene, and internal transcribed spacer 2, complete sequence; and 26S ribosomal RNA gene, partial sequence	1249	1249	96%	0.0	93%	
HM590372.1	Dendrobium aduncum voucher NCHU-D89331201-1006 18S ribosomal RNA gene, partial sequence; internal transcribed spacer 1, 5.8S ribosomal RNA gene, and internal transcribed spacer 2, complete sequence; and 26S ribosomal RNA gene, partial sequence	1234	1234	95%	0.0	93%	
HM590391.1	Dendrobium candidum voucher NCHU-D89331201-1028 18S ribosomal RNA gene, partial sequence; internal transcribed spacer 1, 5.8S ribosomal RNA gene, and internal transcribed spacer 2, complete sequence; and 26S ribosomal RNA gene, partial sequence	1229	1229	99%	0.0	91%	
HM590367.1	Dendrobium tosaense voucher NCHU-D89331201-1001 18S ribosomal RNA gene, partial sequence; internal transcribed spacer 1, 5.8S ribosomal RNA gene, and internal transcribed spacer 2, complete sequence; and 26S ribosomal RNA gene, partial sequence	1175	1175	96%	0.0	91%	
EU840697.1	Dendrobium leptocladum internal transcribed spacer 1, 5.8S ribosomal RNA gene, and internal transcribed spacer 2, complete sequence	1164	1164	71%	0.0	99%	
AF521612.1	Dendrobium leptocladum internal transcribed spacer 1, 5.8S ribosomal RNA gene, and internal transcribed spacer 2, complete sequence	1153	1153	71%	0.0	99%	
HM590377.1	Dendrobium hancockii voucher NCHU-D89331201-1011 18S ribosomal RNA gene, partial sequence; internal transcribed spacer 1, 5.8S ribosomal RNA gene, and internal transcribed spacer 2, complete sequence; and 26S ribosomal RNA gene, partial sequence	1066	1066	99%	0.0	88%	
HM590376.1	Dendrobium chrysanthum voucher NCHU-D89331201-1010 18S ribosomal RNA gene, partial sequence; internal transcribed spacer 1, 5.8S ribosomal RNA gene, and internal transcribed spacer 2, complete sequence; and 26S ribosomal RNA gene, partial sequence	1066	1066	99%	0.0	88%	
HM590368.1	Dendrobium huoshanense voucher NCHU-D89331201-1002 18S ribosomal RNA gene, partial sequence; internal transcribed spacer 1, 5.8S ribosomal RNA gene, and internal transcribed spacer 2, complete sequence; and 26S ribosomal RNA gene, partial sequence	1064	1064	99%	0.0	88%	
HM590384.1	Dendrobium aphyllum voucher NCHU-D89331201-1018 18S ribosomal RNA gene, partial sequence; internal transcribed spacer 1, 5.8S ribosomal RNA gene, and internal transcribed spacer 2, complete sequence; and 26S ribosomal RNA gene, partial sequence	1027	1027	97%	0.0	88%	
HM590378.1	Dendrobium parishii voucher NCHU-D89331201-1012 18S ribosomal RNA gene, partial sequence; internal transcribed spacer 1, 5.8S ribosomal RNA gene, and internal transcribed spacer 2, complete sequence; and 26S ribosomal RNA gene, partial sequence	1022	1022	99%	0.0	87%	

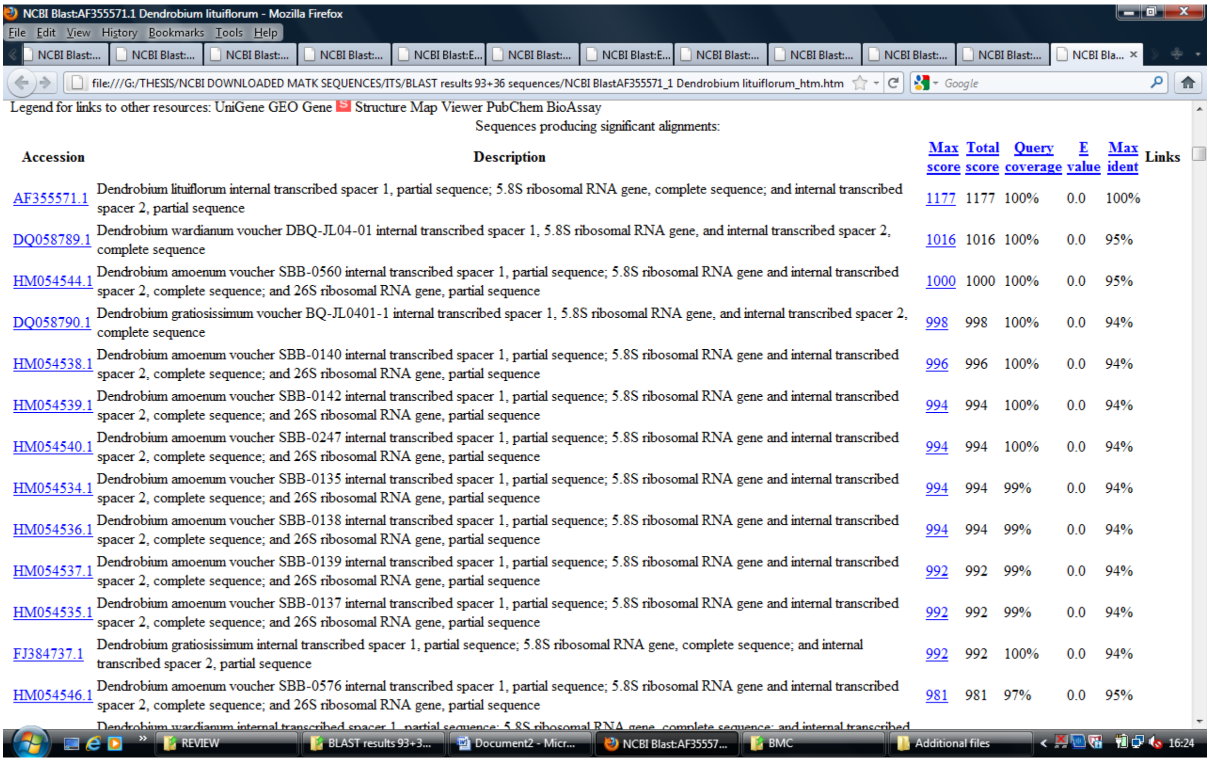

NCBI Blast:AF355571.1 Dendrobium lituiflorum - Mozilla Firefox

Legend for links to other resources: UniGene GEO Gene Structure Map Viewer PubChem BioAssay

Sequences producing significant alignments:

Accession	Description	Max score	Total score	Query coverage	E value	Max ident	Links
AF355571.1	Dendrobium lituiflorum internal transcribed spacer 1, partial sequence; 5.8S ribosomal RNA gene, complete sequence; and internal transcribed spacer 2, partial sequence	1177	1177	100%	0.0	100%	
DQ058789.1	Dendrobium wardianum voucher DBQ-JL04-01 internal transcribed spacer 1, 5.8S ribosomal RNA gene, and internal transcribed spacer 2, complete sequence	1016	1016	100%	0.0	95%	
HM054544.1	Dendrobium amoenum voucher SBB-0560 internal transcribed spacer 1, partial sequence; 5.8S ribosomal RNA gene and internal transcribed spacer 2, complete sequence; and 26S ribosomal RNA gene, partial sequence	1000	1000	100%	0.0	95%	
DQ058790.1	Dendrobium gratiosissimum voucher BQ-JL0401-1 internal transcribed spacer 1, 5.8S ribosomal RNA gene, and internal transcribed spacer 2, complete sequence	998	998	100%	0.0	94%	
HM054538.1	Dendrobium amoenum voucher SBB-0140 internal transcribed spacer 1, partial sequence; 5.8S ribosomal RNA gene and internal transcribed spacer 2, complete sequence; and 26S ribosomal RNA gene, partial sequence	996	996	100%	0.0	94%	
HM054539.1	Dendrobium amoenum voucher SBB-0142 internal transcribed spacer 1, partial sequence; 5.8S ribosomal RNA gene and internal transcribed spacer 2, complete sequence; and 26S ribosomal RNA gene, partial sequence	994	994	100%	0.0	94%	
HM054540.1	Dendrobium amoenum voucher SBB-0247 internal transcribed spacer 1, partial sequence; 5.8S ribosomal RNA gene and internal transcribed spacer 2, complete sequence; and 26S ribosomal RNA gene, partial sequence	994	994	100%	0.0	94%	
HM054534.1	Dendrobium amoenum voucher SBB-0135 internal transcribed spacer 1, partial sequence; 5.8S ribosomal RNA gene and internal transcribed spacer 2, complete sequence; and 26S ribosomal RNA gene, partial sequence	994	994	99%	0.0	94%	
HM054536.1	Dendrobium amoenum voucher SBB-0138 internal transcribed spacer 1, partial sequence; 5.8S ribosomal RNA gene and internal transcribed spacer 2, complete sequence; and 26S ribosomal RNA gene, partial sequence	994	994	99%	0.0	94%	
HM054537.1	Dendrobium amoenum voucher SBB-0139 internal transcribed spacer 1, partial sequence; 5.8S ribosomal RNA gene and internal transcribed spacer 2, complete sequence; and 26S ribosomal RNA gene, partial sequence	992	992	99%	0.0	94%	
HM054535.1	Dendrobium amoenum voucher SBB-0137 internal transcribed spacer 1, partial sequence; 5.8S ribosomal RNA gene and internal transcribed spacer 2, complete sequence; and 26S ribosomal RNA gene, partial sequence	992	992	99%	0.0	94%	
FJ384737.1	Dendrobium gratiosissimum internal transcribed spacer 1, partial sequence; 5.8S ribosomal RNA gene, complete sequence; and internal transcribed spacer 2, partial sequence	992	992	100%	0.0	94%	
HM054546.1	Dendrobium amoenum voucher SBB-0576 internal transcribed spacer 1, partial sequence; 5.8S ribosomal RNA gene and internal transcribed spacer 2, complete sequence; and 26S ribosomal RNA gene, partial sequence	981	981	97%	0.0	95%	

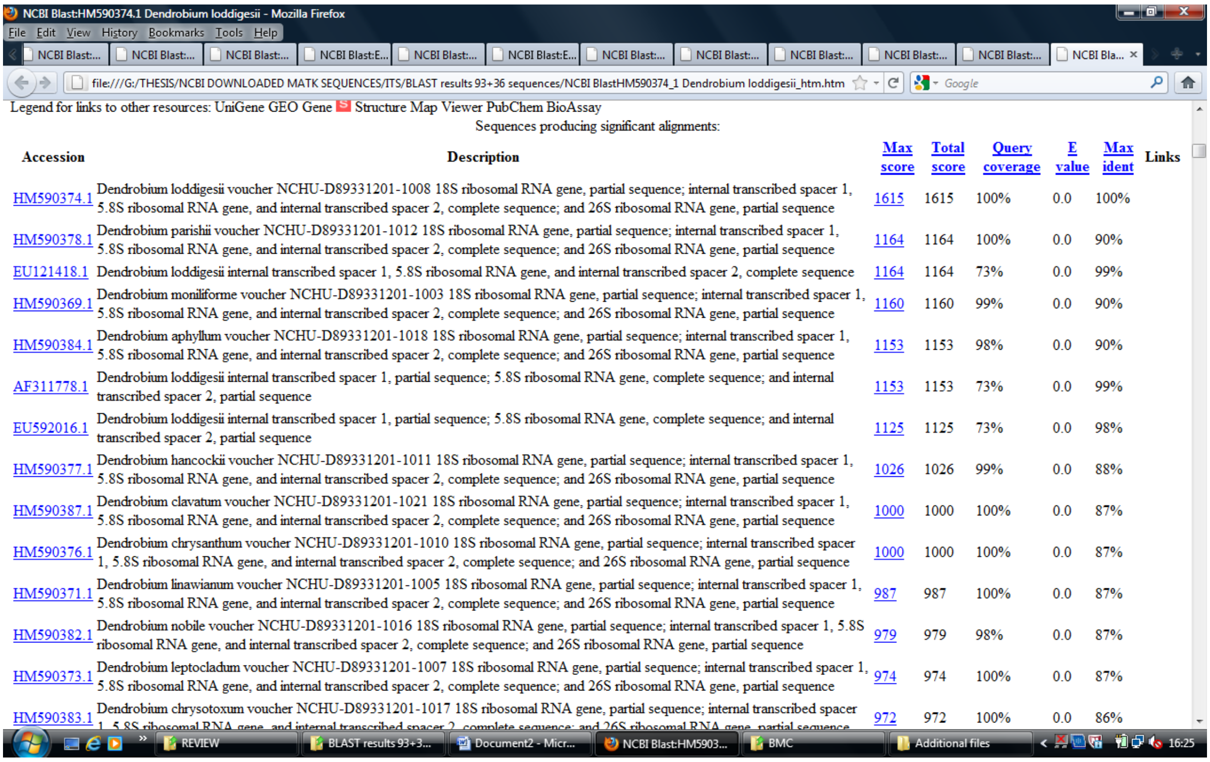

Legend for links to other resources: UniGene GEO Gene Structure Map Viewer PubChem BioAssay

Sequences producing significant alignments:

Accession	Description	Max score	Total score	Query coverage	E value	Max ident	Links
HM590374.1	Dendrobium loddigesii voucher NCHU-D89331201-1008 18S ribosomal RNA gene, partial sequence; internal transcribed spacer 1, 5.8S ribosomal RNA gene, and internal transcribed spacer 2, complete sequence; and 26S ribosomal RNA gene, partial sequence	1615	1615	100%	0.0	100%	
HM590378.1	Dendrobium parishii voucher NCHU-D89331201-1012 18S ribosomal RNA gene, partial sequence; internal transcribed spacer 1, 5.8S ribosomal RNA gene, and internal transcribed spacer 2, complete sequence; and 26S ribosomal RNA gene, partial sequence	1164	1164	100%	0.0	90%	
EU121418.1	Dendrobium loddigesii internal transcribed spacer 1, 5.8S ribosomal RNA gene, and internal transcribed spacer 2, complete sequence	1164	1164	73%	0.0	99%	
HM590369.1	Dendrobium moniliforme voucher NCHU-D89331201-1003 18S ribosomal RNA gene, partial sequence; internal transcribed spacer 1, 5.8S ribosomal RNA gene, and internal transcribed spacer 2, complete sequence; and 26S ribosomal RNA gene, partial sequence	1160	1160	99%	0.0	90%	
HM590384.1	Dendrobium aphyllum voucher NCHU-D89331201-1018 18S ribosomal RNA gene, partial sequence; internal transcribed spacer 1, 5.8S ribosomal RNA gene, and internal transcribed spacer 2, complete sequence; and 26S ribosomal RNA gene, partial sequence	1153	1153	98%	0.0	90%	
AF311778.1	Dendrobium loddigesii internal transcribed spacer 1, partial sequence; 5.8S ribosomal RNA gene, complete sequence; and internal transcribed spacer 2, partial sequence	1153	1153	73%	0.0	99%	
EU592016.1	Dendrobium loddigesii internal transcribed spacer 1, partial sequence; 5.8S ribosomal RNA gene, complete sequence; and internal transcribed spacer 2, partial sequence	1125	1125	73%	0.0	98%	
HM590377.1	Dendrobium hancockii voucher NCHU-D89331201-1011 18S ribosomal RNA gene, partial sequence; internal transcribed spacer 1, 5.8S ribosomal RNA gene, and internal transcribed spacer 2, complete sequence; and 26S ribosomal RNA gene, partial sequence	1026	1026	99%	0.0	88%	
HM590387.1	Dendrobium clavatum voucher NCHU-D89331201-1021 18S ribosomal RNA gene, partial sequence; internal transcribed spacer 1, 5.8S ribosomal RNA gene, and internal transcribed spacer 2, complete sequence; and 26S ribosomal RNA gene, partial sequence	1000	1000	100%	0.0	87%	
HM590376.1	Dendrobium chrysanthum voucher NCHU-D89331201-1010 18S ribosomal RNA gene, partial sequence; internal transcribed spacer 1, 5.8S ribosomal RNA gene, and internal transcribed spacer 2, complete sequence; and 26S ribosomal RNA gene, partial sequence	1000	1000	100%	0.0	87%	
HM590371.1	Dendrobium linawianum voucher NCHU-D89331201-1005 18S ribosomal RNA gene, partial sequence; internal transcribed spacer 1, 5.8S ribosomal RNA gene, and internal transcribed spacer 2, complete sequence; and 26S ribosomal RNA gene, partial sequence	987	987	100%	0.0	87%	
HM590382.1	Dendrobium nobile voucher NCHU-D89331201-1016 18S ribosomal RNA gene, partial sequence; internal transcribed spacer 1, 5.8S ribosomal RNA gene, and internal transcribed spacer 2, complete sequence; and 26S ribosomal RNA gene, partial sequence	979	979	98%	0.0	87%	
HM590373.1	Dendrobium leptocladum voucher NCHU-D89331201-1007 18S ribosomal RNA gene, partial sequence; internal transcribed spacer 1, 5.8S ribosomal RNA gene, and internal transcribed spacer 2, complete sequence; and 26S ribosomal RNA gene, partial sequence	974	974	100%	0.0	87%	
HM590383.1	Dendrobium chrysotoxum voucher NCHU-D89331201-1017 18S ribosomal RNA gene, partial sequence; internal transcribed spacer 1, 5.8S ribosomal RNA gene, and internal transcribed spacer 2, complete sequence; and 26S ribosomal RNA gene, partial sequence	972	972	100%	0.0	86%	

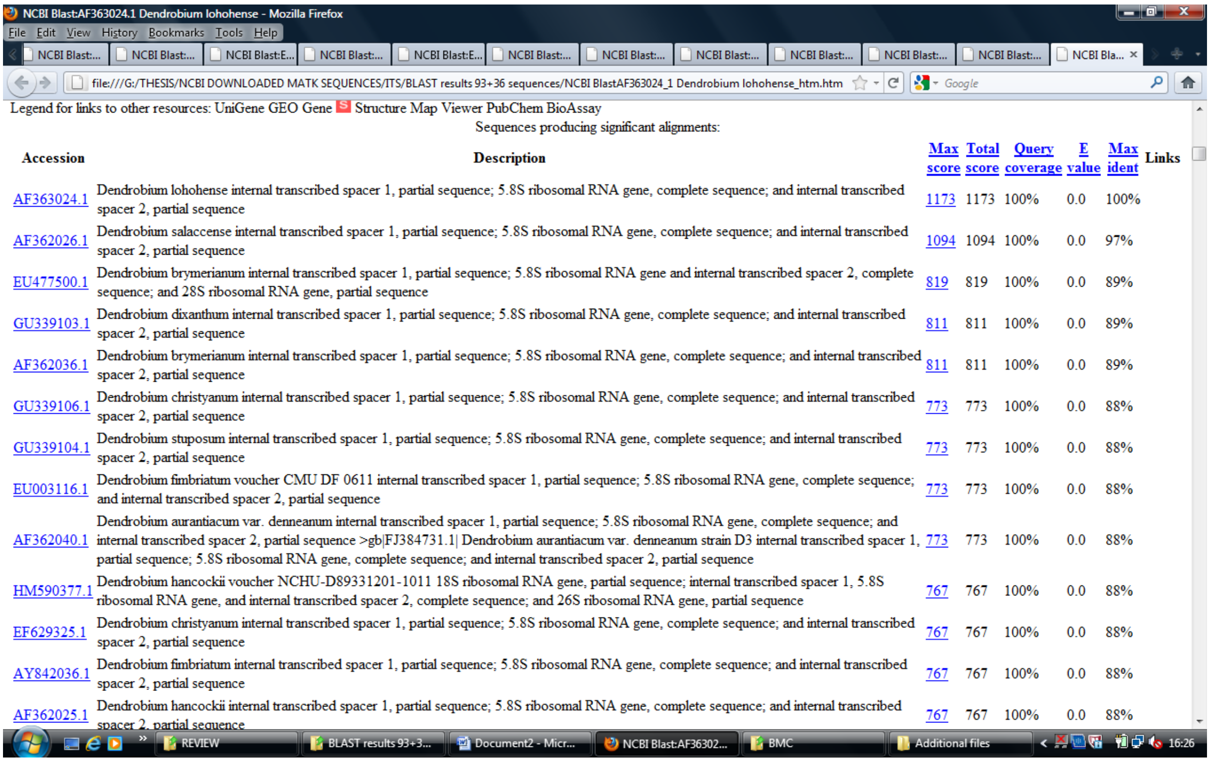

Legend for links to other resources: UniGene GEO Gene Structure Map Viewer PubChem BioAssay

Sequences producing significant alignments:

Accession	Description	Max score	Total score	Query coverage	E value	Max ident	Links
AF363024.1	Dendrobium lohohense internal transcribed spacer 1, partial sequence; 5.8S ribosomal RNA gene, complete sequence; and internal transcribed spacer 2, partial sequence	1173	1173	100%	0.0	100%	
AF362026.1	Dendrobium salaccense internal transcribed spacer 1, partial sequence; 5.8S ribosomal RNA gene, complete sequence; and internal transcribed spacer 2, partial sequence	1094	1094	100%	0.0	97%	
EU477500.1	Dendrobium brymerianum internal transcribed spacer 1, partial sequence; 5.8S ribosomal RNA gene and internal transcribed spacer 2, complete sequence; and 28S ribosomal RNA gene, partial sequence	819	819	100%	0.0	89%	
GU339103.1	Dendrobium dixanthum internal transcribed spacer 1, partial sequence; 5.8S ribosomal RNA gene, complete sequence; and internal transcribed spacer 2, partial sequence	811	811	100%	0.0	89%	
AF362036.1	Dendrobium brymerianum internal transcribed spacer 1, partial sequence; 5.8S ribosomal RNA gene, complete sequence; and internal transcribed spacer 2, partial sequence	811	811	100%	0.0	89%	
GU339106.1	Dendrobium christyanum internal transcribed spacer 1, partial sequence; 5.8S ribosomal RNA gene, complete sequence; and internal transcribed spacer 2, partial sequence	773	773	100%	0.0	88%	
GU339104.1	Dendrobium stuposum internal transcribed spacer 1, partial sequence; 5.8S ribosomal RNA gene, complete sequence; and internal transcribed spacer 2, partial sequence	773	773	100%	0.0	88%	
EU003116.1	Dendrobium fimbriatum voucher CMU DF 0611 internal transcribed spacer 1, partial sequence; 5.8S ribosomal RNA gene, complete sequence; and internal transcribed spacer 2, partial sequence	773	773	100%	0.0	88%	
AF362040.1	Dendrobium aurantiacum var. denneanum internal transcribed spacer 1, partial sequence; 5.8S ribosomal RNA gene, complete sequence; and internal transcribed spacer 2, partial sequence >gb|FJ384731.1| Dendrobium aurantiacum var. denneanum strain D3 internal transcribed spacer 1, partial sequence; 5.8S ribosomal RNA gene, complete sequence; and internal transcribed spacer 2, partial sequence	773	773	100%	0.0	88%	
HM590377.1	Dendrobium hancockii voucher NCHU-D89331201-1011 18S ribosomal RNA gene, partial sequence; internal transcribed spacer 1, 5.8S ribosomal RNA gene, and internal transcribed spacer 2, complete sequence; and 26S ribosomal RNA gene, partial sequence	767	767	100%	0.0	88%	
EF629325.1	Dendrobium christyanum internal transcribed spacer 1, partial sequence; 5.8S ribosomal RNA gene, complete sequence; and internal transcribed spacer 2, partial sequence	767	767	100%	0.0	88%	
AY842036.1	Dendrobium fimbriatum internal transcribed spacer 1, partial sequence; 5.8S ribosomal RNA gene, complete sequence; and internal transcribed spacer 2, partial sequence	767	767	100%	0.0	88%	
AF362025.1	Dendrobium hancockii internal transcribed spacer 1, partial sequence; 5.8S ribosomal RNA gene, complete sequence; and internal transcribed spacer 2, partial sequence	767	767	100%	0.0	88%	

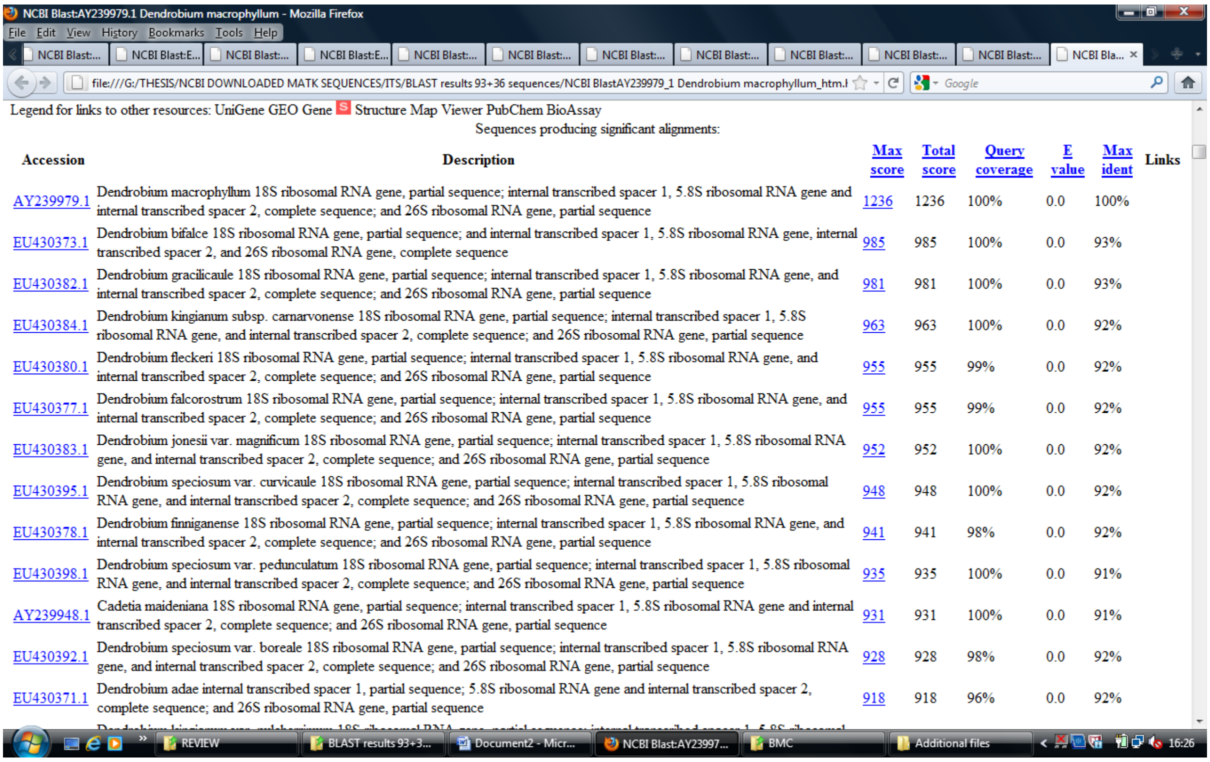

NCBI Blast:AY239979.1 Dendrobium macrophyllum - Mozilla Firefox

Legend for links to other resources: UniGene GEO Gene S Structure Map Viewer PubChem BioAssay

Sequences producing significant alignments:

Accession	Description	Max score	Total score	Query coverage	E value	Max ident	Links
AY239979.1	Dendrobium macrophyllum 18S ribosomal RNA gene, partial sequence; internal transcribed spacer 1, 5.8S ribosomal RNA gene and internal transcribed spacer 2, complete sequence; and 26S ribosomal RNA gene, partial sequence	1236	1236	100%	0.0	100%	
EU430373.1	Dendrobium bifalce 18S ribosomal RNA gene, partial sequence; and internal transcribed spacer 1, 5.8S ribosomal RNA gene, internal transcribed spacer 2, and 26S ribosomal RNA gene, complete sequence	985	985	100%	0.0	93%	
EU430382.1	Dendrobium gracilicaule 18S ribosomal RNA gene, partial sequence; internal transcribed spacer 1, 5.8S ribosomal RNA gene, and internal transcribed spacer 2, complete sequence; and 26S ribosomal RNA gene, partial sequence	981	981	100%	0.0	93%	
EU430384.1	Dendrobium kingianum subsp. carnarvonense 18S ribosomal RNA gene, partial sequence; internal transcribed spacer 1, 5.8S ribosomal RNA gene, and internal transcribed spacer 2, complete sequence; and 26S ribosomal RNA gene, partial sequence	963	963	100%	0.0	92%	
EU430380.1	Dendrobium fleckeri 18S ribosomal RNA gene, partial sequence; internal transcribed spacer 1, 5.8S ribosomal RNA gene, and internal transcribed spacer 2, complete sequence; and 26S ribosomal RNA gene, partial sequence	955	955	99%	0.0	92%	
EU430377.1	Dendrobium falcorostrum 18S ribosomal RNA gene, partial sequence; internal transcribed spacer 1, 5.8S ribosomal RNA gene, and internal transcribed spacer 2, complete sequence; and 26S ribosomal RNA gene, partial sequence	955	955	99%	0.0	92%	
EU430383.1	Dendrobium jonesii var. magnificum 18S ribosomal RNA gene, partial sequence; internal transcribed spacer 1, 5.8S ribosomal RNA gene, and internal transcribed spacer 2, complete sequence; and 26S ribosomal RNA gene, partial sequence	952	952	100%	0.0	92%	
EU430395.1	Dendrobium speciosum var. curvicaule 18S ribosomal RNA gene, partial sequence; internal transcribed spacer 1, 5.8S ribosomal RNA gene, and internal transcribed spacer 2, complete sequence; and 26S ribosomal RNA gene, partial sequence	948	948	100%	0.0	92%	
EU430378.1	Dendrobium finniganense 18S ribosomal RNA gene, partial sequence; internal transcribed spacer 1, 5.8S ribosomal RNA gene, and internal transcribed spacer 2, complete sequence; and 26S ribosomal RNA gene, partial sequence	941	941	98%	0.0	92%	
EU430398.1	Dendrobium speciosum var. pedunculatum 18S ribosomal RNA gene, partial sequence; internal transcribed spacer 1, 5.8S ribosomal RNA gene, and internal transcribed spacer 2, complete sequence; and 26S ribosomal RNA gene, partial sequence	935	935	100%	0.0	91%	
AY239948.1	Cadetia maideniana 18S ribosomal RNA gene, partial sequence; internal transcribed spacer 1, 5.8S ribosomal RNA gene and internal transcribed spacer 2, complete sequence; and 26S ribosomal RNA gene, partial sequence	931	931	100%	0.0	91%	
EU430392.1	Dendrobium speciosum var. boreale 18S ribosomal RNA gene, partial sequence; internal transcribed spacer 1, 5.8S ribosomal RNA gene, and internal transcribed spacer 2, complete sequence; and 26S ribosomal RNA gene, partial sequence	928	928	98%	0.0	92%	
EU430371.1	Dendrobium adae internal transcribed spacer 1, partial sequence; 5.8S ribosomal RNA gene and internal transcribed spacer 2, complete sequence; and 26S ribosomal RNA gene, partial sequence	918	918	96%	0.0	92%	
[illegible]	Dendrobium kingianum var. [illegible] 18S ribosomal RNA gene, partial sequence; internal transcribed spacer 1, 5.8S ribosomal [illegible]	[illegible]	[illegible]	[illegible]	[illegible]	[illegible]	

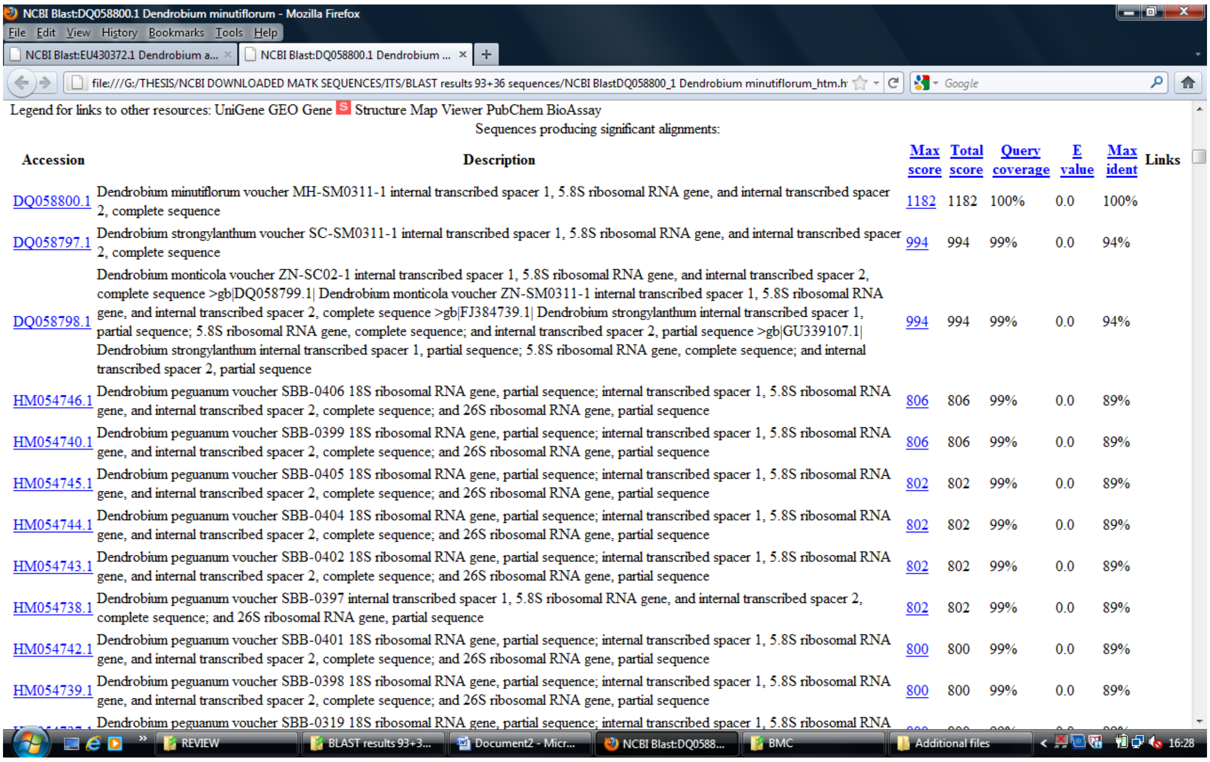

NCBI Blast:DQ058800.1 Dendrobium minutiflorum - Mozilla Firefox

Legend for links to other resources: UniGene GEO Gene S Structure Map Viewer PubChem BioAssay

Sequences producing significant alignments:

Accession	Description	Max score	Total score	Query coverage	E value	Max ident	Links
DQ058800.1	Dendrobium minutiflorum voucher MH-SM0311-1 internal transcribed spacer 1, 5.8S ribosomal RNA gene, and internal transcribed spacer 2, complete sequence	1182	1182	100%	0.0	100%	
DQ058797.1	Dendrobium strongylanthum voucher SC-SM0311-1 internal transcribed spacer 1, 5.8S ribosomal RNA gene, and internal transcribed spacer 2, complete sequence	994	994	99%	0.0	94%	
DQ058798.1	Dendrobium monticola voucher ZN-SC02-1 internal transcribed spacer 1, 5.8S ribosomal RNA gene, and internal transcribed spacer 2, complete sequence >gb|DQ058799.1| Dendrobium monticola voucher ZN-SM0311-1 internal transcribed spacer 1, 5.8S ribosomal RNA gene, and internal transcribed spacer 2, complete sequence >gb|FJ384739.1| Dendrobium strongylanthum internal transcribed spacer 1, partial sequence; 5.8S ribosomal RNA gene, complete sequence; and internal transcribed spacer 2, partial sequence >gb|GU339107.1| Dendrobium strongylanthum internal transcribed spacer 1, partial sequence; 5.8S ribosomal RNA gene, complete sequence; and internal transcribed spacer 2, partial sequence	994	994	99%	0.0	94%	
HM054746.1	Dendrobium peguanum voucher SBB-0406 18S ribosomal RNA gene, partial sequence; internal transcribed spacer 1, 5.8S ribosomal RNA gene, and internal transcribed spacer 2, complete sequence; and 26S ribosomal RNA gene, partial sequence	806	806	99%	0.0	89%	
HM054740.1	Dendrobium peguanum voucher SBB-0399 18S ribosomal RNA gene, partial sequence; internal transcribed spacer 1, 5.8S ribosomal RNA gene, and internal transcribed spacer 2, complete sequence; and 26S ribosomal RNA gene, partial sequence	806	806	99%	0.0	89%	
HM054745.1	Dendrobium peguanum voucher SBB-0405 18S ribosomal RNA gene, partial sequence; internal transcribed spacer 1, 5.8S ribosomal RNA gene, and internal transcribed spacer 2, complete sequence; and 26S ribosomal RNA gene, partial sequence	802	802	99%	0.0	89%	
HM054744.1	Dendrobium peguanum voucher SBB-0404 18S ribosomal RNA gene, partial sequence; internal transcribed spacer 1, 5.8S ribosomal RNA gene, and internal transcribed spacer 2, complete sequence; and 26S ribosomal RNA gene, partial sequence	802	802	99%	0.0	89%	
HM054743.1	Dendrobium peguanum voucher SBB-0402 18S ribosomal RNA gene, partial sequence; internal transcribed spacer 1, 5.8S ribosomal RNA gene, and internal transcribed spacer 2, complete sequence; and 26S ribosomal RNA gene, partial sequence	802	802	99%	0.0	89%	
HM054738.1	Dendrobium peguanum voucher SBB-0397 internal transcribed spacer 1, 5.8S ribosomal RNA gene, and internal transcribed spacer 2, complete sequence; and 26S ribosomal RNA gene, partial sequence	802	802	99%	0.0	89%	
HM054742.1	Dendrobium peguanum voucher SBB-0401 18S ribosomal RNA gene, partial sequence; internal transcribed spacer 1, 5.8S ribosomal RNA gene, and internal transcribed spacer 2, complete sequence; and 26S ribosomal RNA gene, partial sequence	800	800	99%	0.0	89%	
HM054739.1	Dendrobium peguanum voucher SBB-0398 18S ribosomal RNA gene, partial sequence; internal transcribed spacer 1, 5.8S ribosomal RNA gene, and internal transcribed spacer 2, complete sequence; and 26S ribosomal RNA gene, partial sequence	800	800	99%	0.0	89%	
[illegible]	Dendrobium peguanum voucher SBB-0319 18S ribosomal RNA gene, partial sequence; internal transcribed spacer 1, 5.8S ribosomal RNA [illegible]	[illegible]	[illegible]	[illegible]	[illegible]	[illegible]	

NCBI Blast:HM590386.1 Dendrobium miyakei - Mozilla Firefox

file:///G:/THESIS/NCBI DOWNLOADED MATK SEQUENCES/ITS/BLAST results 93+36 sequences/NCBI BlastHM590386_1 Dendrobium miyakei_htm.htm

Legend for links to other resources: UniGene GEO Gene Structure Map Viewer PubChem BioAssay

Sequences producing significant alignments:

Accession	Description	Max score	Total score	Query coverage	E value	Max ident	Links
HM590386.1	Dendrobium miyakei voucher NCHU-D89331201-1020 18S ribosomal RNA gene, partial sequence; internal transcribed spacer 1, 5.8S ribosomal RNA gene, and internal transcribed spacer 2, complete sequence; and 26S ribosomal RNA gene, partial sequence	1640	1640	100%	0.0	100%	
HM590385.1	Dendrobium chameleon voucher NCHU-D89331201-1019 18S ribosomal RNA gene, partial sequence; internal transcribed spacer 1, 5.8S ribosomal RNA gene, and internal transcribed spacer 2, complete sequence; and 26S ribosomal RNA gene, partial sequence	1471	1471	100%	0.0	96%	
AY239970.1	Dendrobium goldschmidtianum 18S ribosomal RNA gene, partial sequence; internal transcribed spacer 1, 5.8S ribosomal RNA gene and internal transcribed spacer 2, complete sequence; and 26S ribosomal RNA gene, partial sequence	1229	1229	74%	0.0	100%	
AF521614.1	Dendrobium miyakei internal transcribed spacer 1, 5.8S ribosomal RNA gene, and internal transcribed spacer 2, complete sequence	1184	1184	72%	0.0	100%	
EU840695.1	Dendrobium goldschmidtianum internal transcribed spacer 1, 5.8S ribosomal RNA gene, and internal transcribed spacer 2, complete sequence	1179	1179	72%	0.0	99%	
EU840694.1	Dendrobium victoriae-reginae internal transcribed spacer 1, 5.8S ribosomal RNA gene, and internal transcribed spacer 2, complete sequence	1146	1146	72%	0.0	98%	
AY239994.1	Dendrobium serratilabium 18S ribosomal RNA gene, partial sequence; internal transcribed spacer 1, 5.8S ribosomal RNA gene and internal transcribed spacer 2, complete sequence; and 26S ribosomal RNA gene, partial sequence	1088	1088	74%	0.0	96%	
AY239960.1	Dendrobium chameleon 18S ribosomal RNA gene, partial sequence; internal transcribed spacer 1, 5.8S ribosomal RNA gene and internal transcribed spacer 2, complete sequence; and 26S ribosomal RNA gene, partial sequence	1061	1061	74%	0.0	95%	
AY240006.1	Dendrobium yeageri 18S ribosomal RNA gene, partial sequence; internal transcribed spacer 1, 5.8S ribosomal RNA gene and internal transcribed spacer 2, complete sequence; and 26S ribosomal RNA gene, partial sequence	1055	1055	74%	0.0	95%	
AY239958.1	Dendrobium ceraula 18S ribosomal RNA gene, partial sequence; internal transcribed spacer 1, 5.8S ribosomal RNA gene and internal transcribed spacer 2, complete sequence; and 26S ribosomal RNA gene, partial sequence	1042	1042	74%	0.0	95%	
AY240004.1	Dendrobium victoriae-reginae 18S ribosomal RNA gene, partial sequence; internal transcribed spacer 1, 5.8S ribosomal RNA gene and internal transcribed spacer 2, complete sequence; and 26S ribosomal RNA gene, partial sequence	1037	1037	74%	0.0	94%	
AF521607.1	Dendrobium chameleon internal transcribed spacer 1, 5.8S ribosomal RNA gene, and internal transcribed spacer 2, complete sequence	1005	1005	72%	0.0	95%	
HM590377.1	Dendrobium hancockii voucher NCHU-D89331201-1011 18S ribosomal RNA gene, partial sequence; internal transcribed spacer 1, 5.8S ribosomal RNA gene, and internal transcribed spacer 2, complete sequence; and 26S ribosomal RNA gene, partial sequence	987	987	99%	0.0	87%	
HM590370.1	Dendrobium crumenatum voucher NCHU-D89331201-1004 18S ribosomal RNA gene, partial sequence; internal transcribed spacer 1, 5.8S ribosomal RNA gene, and internal transcribed spacer 2, complete sequence; and 26S ribosomal RNA gene, partial sequence	961	961	100%	0.0	86%	

NCBI Blast:AY239980.1 Dendrobium mohlianum - Mozilla Firefox

file:///G:/THESIS/NCBI DOWNLOADED MATK SEQUENCES/ITS/BLAST results 93+36 sequences/NCBI BlastAY239980_1 Dendrobium mohlianum_htm.htm

Legend for links to other resources: UniGene GEO Gene Structure Map Viewer PubChem BioAssay

Sequences producing significant alignments:

Accession	Description	Max score	Total score	Query coverage	E value	Max ident	Links
AY239980.1	Dendrobium mohlianum 18S ribosomal RNA gene, partial sequence; internal transcribed spacer 1, 5.8S ribosomal RNA gene and internal transcribed spacer 2, complete sequence; and 26S ribosomal RNA gene, partial sequence	1225	1225	100%	0.0	100%	
AY239977.1	Dendrobium lawesii 18S ribosomal RNA gene, partial sequence; internal transcribed spacer 1, 5.8S ribosomal RNA gene and internal transcribed spacer 2, complete sequence; and 26S ribosomal RNA gene, partial sequence	1040	1040	100%	0.0	95%	
AY239968.1	Dendrobium fulgidum 18S ribosomal RNA gene, partial sequence; internal transcribed spacer 1, 5.8S ribosomal RNA gene and internal transcribed spacer 2, complete sequence; and 26S ribosomal RNA gene, partial sequence	965	965	100%	0.0	92%	
AY239976.1	Dendrobium lancifolium 18S ribosomal RNA gene, partial sequence; internal transcribed spacer 1, 5.8S ribosomal RNA gene and internal transcribed spacer 2, complete sequence; and 26S ribosomal RNA gene, partial sequence	926	926	100%	0.0	91%	
AY239960.1	Dendrobium chameleon 18S ribosomal RNA gene, partial sequence; internal transcribed spacer 1, 5.8S ribosomal RNA gene and internal transcribed spacer 2, complete sequence; and 26S ribosomal RNA gene, partial sequence	920	920	100%	0.0	91%	
AY240006.1	Dendrobium yeageri 18S ribosomal RNA gene, partial sequence; internal transcribed spacer 1, 5.8S ribosomal RNA gene and internal transcribed spacer 2, complete sequence; and 26S ribosomal RNA gene, partial sequence	915	915	100%	0.0	91%	
AY239986.1	Dendrobium nothofagicola 18S ribosomal RNA gene, partial sequence; internal transcribed spacer 1, 5.8S ribosomal RNA gene and internal transcribed spacer 2, complete sequence; and 26S ribosomal RNA gene, partial sequence	913	913	100%	0.0	91%	
AY239958.1	Dendrobium ceraula 18S ribosomal RNA gene, partial sequence; internal transcribed spacer 1, 5.8S ribosomal RNA gene and internal transcribed spacer 2, complete sequence; and 26S ribosomal RNA gene, partial sequence	907	907	100%	0.0	91%	
AY240004.1	Dendrobium victoriae-reginae 18S ribosomal RNA gene, partial sequence; internal transcribed spacer 1, 5.8S ribosomal RNA gene and internal transcribed spacer 2, complete sequence; and 26S ribosomal RNA gene, partial sequence	902	902	100%	0.0	91%	
AY239994.1	Dendrobium serratilabium 18S ribosomal RNA gene, partial sequence; internal transcribed spacer 1, 5.8S ribosomal RNA gene and internal transcribed spacer 2, complete sequence; and 26S ribosomal RNA gene, partial sequence	893	893	100%	0.0	91%	
HM590385.1	Dendrobium chameleon voucher NCHU-D89331201-1019 18S ribosomal RNA gene, partial sequence; internal transcribed spacer 1, 5.8S ribosomal RNA gene, and internal transcribed spacer 2, complete sequence; and 26S ribosomal RNA gene, partial sequence	881	881	100%	0.0	90%	
AF521607.1	Dendrobium chameleon internal transcribed spacer 1, 5.8S ribosomal RNA gene, and internal transcribed spacer 2, complete sequence	865	865	96%	0.0	91%	
AY239950.1	Dendrobium agathodaemonis 18S ribosomal RNA gene, partial sequence; internal transcribed spacer 1, 5.8S ribosomal RNA gene and internal transcribed spacer 2, complete sequence; and 26S ribosomal RNA gene, partial sequence	863	863	100%	0.0	90%	
AY239997.1	Dendrobium sophronites 18S ribosomal RNA gene, partial sequence; internal transcribed spacer 1, 5.8S ribosomal RNA gene and internal transcribed spacer 2, complete sequence; and 26S ribosomal RNA gene, partial sequence	857	857	100%	0.0	90%	

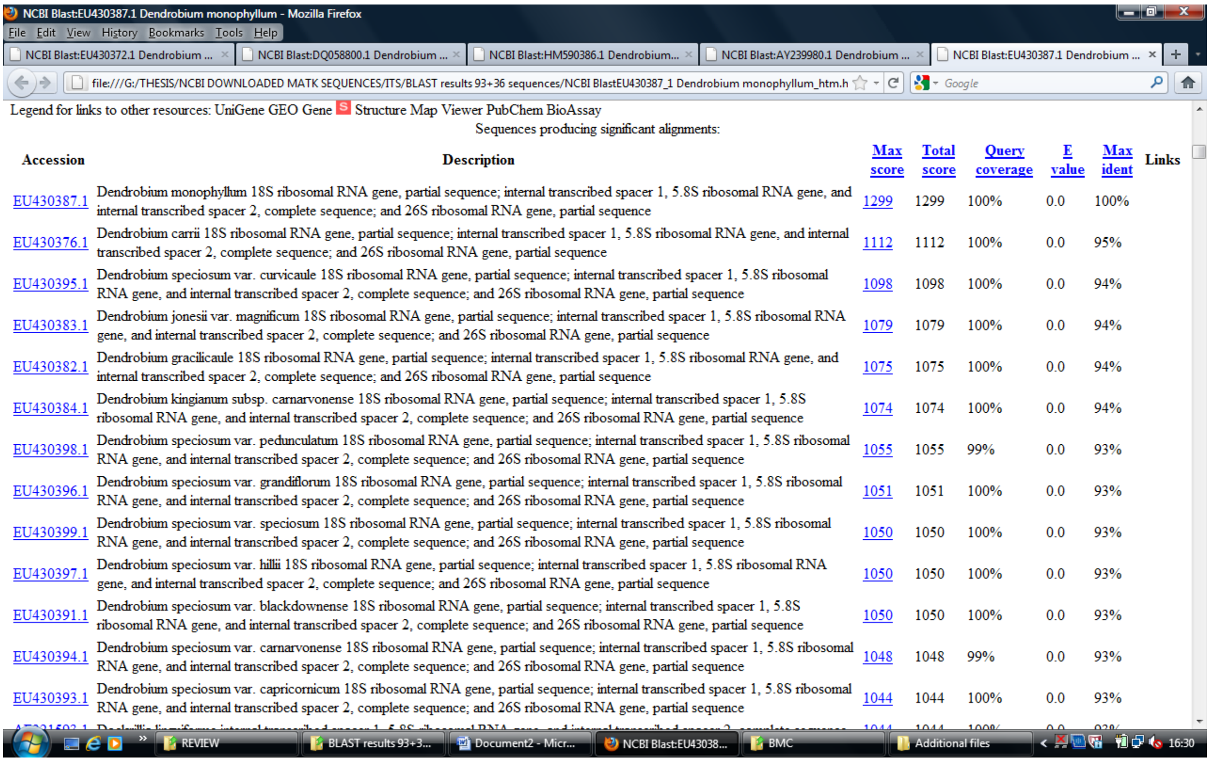

Legend for links to other resources: UniGene GEO Gene Structure Map Viewer PubChem BioAssay

Sequences producing significant alignments:

Accession	Description	Max score	Total score	Query coverage	E value	Max ident	Links
EU430387.1	Dendrobium monophyllum 18S ribosomal RNA gene, partial sequence; internal transcribed spacer 1, 5.8S ribosomal RNA gene, and internal transcribed spacer 2, complete sequence; and 26S ribosomal RNA gene, partial sequence	1299	1299	100%	0.0	100%	
EU430376.1	Dendrobium carrii 18S ribosomal RNA gene, partial sequence; internal transcribed spacer 1, 5.8S ribosomal RNA gene, and internal transcribed spacer 2, complete sequence; and 26S ribosomal RNA gene, partial sequence	1112	1112	100%	0.0	95%	
EU430395.1	Dendrobium speciosum var. curvicaule 18S ribosomal RNA gene, partial sequence; internal transcribed spacer 1, 5.8S ribosomal RNA gene, and internal transcribed spacer 2, complete sequence; and 26S ribosomal RNA gene, partial sequence	1098	1098	100%	0.0	94%	
EU430383.1	Dendrobium jonesii var. magnificum 18S ribosomal RNA gene, partial sequence; internal transcribed spacer 1, 5.8S ribosomal RNA gene, and internal transcribed spacer 2, complete sequence; and 26S ribosomal RNA gene, partial sequence	1079	1079	100%	0.0	94%	
EU430382.1	Dendrobium gracilicaule 18S ribosomal RNA gene, partial sequence; internal transcribed spacer 1, 5.8S ribosomal RNA gene, and internal transcribed spacer 2, complete sequence; and 26S ribosomal RNA gene, partial sequence	1075	1075	100%	0.0	94%	
EU430384.1	Dendrobium kingianum subsp. carnarvonense 18S ribosomal RNA gene, partial sequence; internal transcribed spacer 1, 5.8S ribosomal RNA gene, and internal transcribed spacer 2, complete sequence; and 26S ribosomal RNA gene, partial sequence	1074	1074	100%	0.0	94%	
EU430398.1	Dendrobium speciosum var. pedunculatum 18S ribosomal RNA gene, partial sequence; internal transcribed spacer 1, 5.8S ribosomal RNA gene, and internal transcribed spacer 2, complete sequence; and 26S ribosomal RNA gene, partial sequence	1055	1055	99%	0.0	93%	
EU430396.1	Dendrobium speciosum var. grandiflorum 18S ribosomal RNA gene, partial sequence; internal transcribed spacer 1, 5.8S ribosomal RNA gene, and internal transcribed spacer 2, complete sequence; and 26S ribosomal RNA gene, partial sequence	1051	1051	100%	0.0	93%	
EU430399.1	Dendrobium speciosum var. speciosum 18S ribosomal RNA gene, partial sequence; internal transcribed spacer 1, 5.8S ribosomal RNA gene, and internal transcribed spacer 2, complete sequence; and 26S ribosomal RNA gene, partial sequence	1050	1050	100%	0.0	93%	
EU430397.1	Dendrobium speciosum var. hillii 18S ribosomal RNA gene, partial sequence; internal transcribed spacer 1, 5.8S ribosomal RNA gene, and internal transcribed spacer 2, complete sequence; and 26S ribosomal RNA gene, partial sequence	1050	1050	100%	0.0	93%	
EU430391.1	Dendrobium speciosum var. blackdownense 18S ribosomal RNA gene, partial sequence; internal transcribed spacer 1, 5.8S ribosomal RNA gene, and internal transcribed spacer 2, complete sequence; and 26S ribosomal RNA gene, partial sequence	1050	1050	100%	0.0	93%	
EU430394.1	Dendrobium speciosum var. carnarvonense 18S ribosomal RNA gene, partial sequence; internal transcribed spacer 1, 5.8S ribosomal RNA gene, and internal transcribed spacer 2, complete sequence; and 26S ribosomal RNA gene, partial sequence	1048	1048	99%	0.0	93%	
EU430393.1	Dendrobium speciosum var. capricornicum 18S ribosomal RNA gene, partial sequence; internal transcribed spacer 1, 5.8S ribosomal RNA gene, and internal transcribed spacer 2, complete sequence; and 26S ribosomal RNA gene, partial sequence	1044	1044	100%	0.0	93%	
[illegible]	[illegible]	[illegible]	[illegible]	[illegible]	[illegible]	[illegible]	

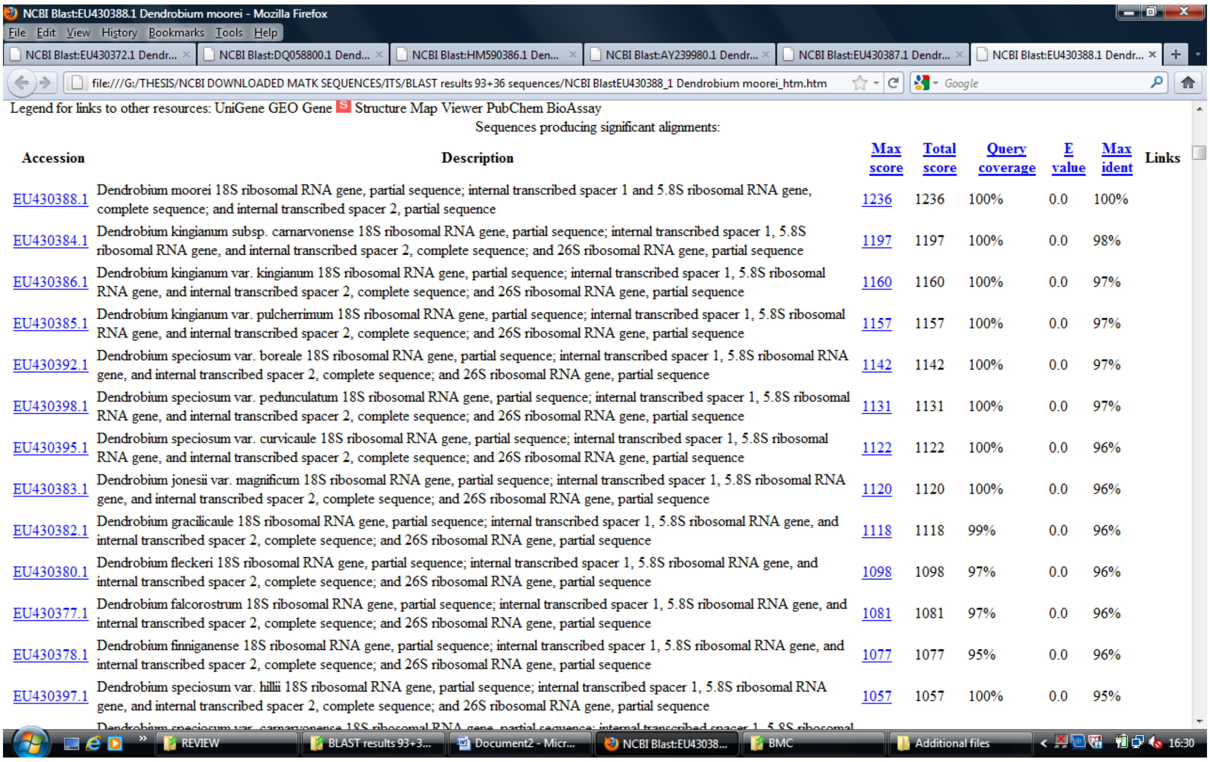

Legend for links to other resources: UniGene GEO Gene Structure Map Viewer PubChem BioAssay

Sequences producing significant alignments:

Accession	Description	Max score	Total score	Query coverage	E value	Max ident	Links
EU430388.1	Dendrobium moorei 18S ribosomal RNA gene, partial sequence; internal transcribed spacer 1 and 5.8S ribosomal RNA gene, complete sequence; and internal transcribed spacer 2, partial sequence	1236	1236	100%	0.0	100%	
EU430384.1	Dendrobium kingianum subsp. carnarvonense 18S ribosomal RNA gene, partial sequence; internal transcribed spacer 1, 5.8S ribosomal RNA gene, and internal transcribed spacer 2, complete sequence; and 26S ribosomal RNA gene, partial sequence	1197	1197	100%	0.0	98%	
EU430386.1	Dendrobium kingianum var. kingianum 18S ribosomal RNA gene, partial sequence; internal transcribed spacer 1, 5.8S ribosomal RNA gene, and internal transcribed spacer 2, complete sequence; and 26S ribosomal RNA gene, partial sequence	1160	1160	100%	0.0	97%	
EU430385.1	Dendrobium kingianum var. pulcherrimum 18S ribosomal RNA gene, partial sequence; internal transcribed spacer 1, 5.8S ribosomal RNA gene, and internal transcribed spacer 2, complete sequence; and 26S ribosomal RNA gene, partial sequence	1157	1157	100%	0.0	97%	
EU430392.1	Dendrobium speciosum var. boreale 18S ribosomal RNA gene, partial sequence; internal transcribed spacer 1, 5.8S ribosomal RNA gene, and internal transcribed spacer 2, complete sequence; and 26S ribosomal RNA gene, partial sequence	1142	1142	100%	0.0	97%	
EU430398.1	Dendrobium speciosum var. pedunculatum 18S ribosomal RNA gene, partial sequence; internal transcribed spacer 1, 5.8S ribosomal RNA gene, and internal transcribed spacer 2, complete sequence; and 26S ribosomal RNA gene, partial sequence	1131	1131	100%	0.0	97%	
EU430395.1	Dendrobium speciosum var. curvicaule 18S ribosomal RNA gene, partial sequence; internal transcribed spacer 1, 5.8S ribosomal RNA gene, and internal transcribed spacer 2, complete sequence; and 26S ribosomal RNA gene, partial sequence	1122	1122	100%	0.0	96%	
EU430383.1	Dendrobium jonesii var. magnificum 18S ribosomal RNA gene, partial sequence; internal transcribed spacer 1, 5.8S ribosomal RNA gene, and internal transcribed spacer 2, complete sequence; and 26S ribosomal RNA gene, partial sequence	1120	1120	100%	0.0	96%	
EU430382.1	Dendrobium gracilicaule 18S ribosomal RNA gene, partial sequence; internal transcribed spacer 1, 5.8S ribosomal RNA gene, and internal transcribed spacer 2, complete sequence; and 26S ribosomal RNA gene, partial sequence	1118	1118	99%	0.0	96%	
EU430380.1	Dendrobium fleckeri 18S ribosomal RNA gene, partial sequence; internal transcribed spacer 1, 5.8S ribosomal RNA gene, and internal transcribed spacer 2, complete sequence; and 26S ribosomal RNA gene, partial sequence	1098	1098	97%	0.0	96%	
EU430377.1	Dendrobium falcorostrum 18S ribosomal RNA gene, partial sequence; internal transcribed spacer 1, 5.8S ribosomal RNA gene, and internal transcribed spacer 2, complete sequence; and 26S ribosomal RNA gene, partial sequence	1081	1081	97%	0.0	96%	
EU430378.1	Dendrobium finniganense 18S ribosomal RNA gene, partial sequence; internal transcribed spacer 1, 5.8S ribosomal RNA gene, and internal transcribed spacer 2, complete sequence; and 26S ribosomal RNA gene, partial sequence	1077	1077	95%	0.0	96%	
EU430397.1	Dendrobium speciosum var. hillii 18S ribosomal RNA gene, partial sequence; internal transcribed spacer 1, 5.8S ribosomal RNA gene, and internal transcribed spacer 2, complete sequence; and 26S ribosomal RNA gene, partial sequence	1057	1057	100%	0.0	95%	
[illegible]	[illegible]	[illegible]	[illegible]	[illegible]	[illegible]	[illegible]	

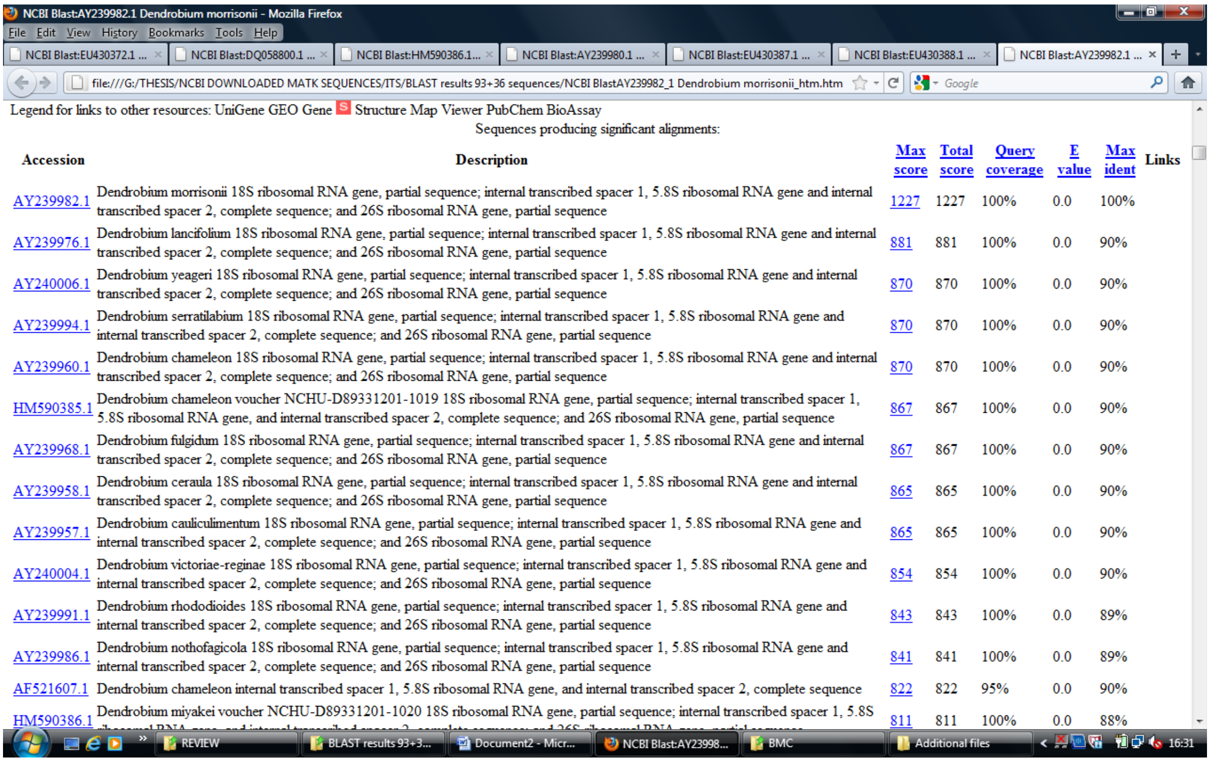

Legend for links to other resources: UniGene GEO Gene Structure Map Viewer PubChem BioAssay

Sequences producing significant alignments:

Accession	Description	Max score	Total score	Query coverage	E value	Max ident	Links
AY239982.1	Dendrobium morrisonii 18S ribosomal RNA gene, partial sequence; internal transcribed spacer 1, 5.8S ribosomal RNA gene and internal transcribed spacer 2, complete sequence; and 26S ribosomal RNA gene, partial sequence	1227	1227	100%	0.0	100%	
AY239976.1	Dendrobium lancifolium 18S ribosomal RNA gene, partial sequence; internal transcribed spacer 1, 5.8S ribosomal RNA gene and internal transcribed spacer 2, complete sequence; and 26S ribosomal RNA gene, partial sequence	881	881	100%	0.0	90%	
AY240006.1	Dendrobium yeageri 18S ribosomal RNA gene, partial sequence; internal transcribed spacer 1, 5.8S ribosomal RNA gene and internal transcribed spacer 2, complete sequence; and 26S ribosomal RNA gene, partial sequence	870	870	100%	0.0	90%	
AY239994.1	Dendrobium serratilabium 18S ribosomal RNA gene, partial sequence; internal transcribed spacer 1, 5.8S ribosomal RNA gene and internal transcribed spacer 2, complete sequence; and 26S ribosomal RNA gene, partial sequence	870	870	100%	0.0	90%	
AY239960.1	Dendrobium chameleon 18S ribosomal RNA gene, partial sequence; internal transcribed spacer 1, 5.8S ribosomal RNA gene and internal transcribed spacer 2, complete sequence; and 26S ribosomal RNA gene, partial sequence	870	870	100%	0.0	90%	
HM590385.1	Dendrobium chameleon voucher NCHU-D89331201-1019 18S ribosomal RNA gene, partial sequence; internal transcribed spacer 1, 5.8S ribosomal RNA gene, and internal transcribed spacer 2, complete sequence; and 26S ribosomal RNA gene, partial sequence	867	867	100%	0.0	90%	
AY239968.1	Dendrobium fulgidum 18S ribosomal RNA gene, partial sequence; internal transcribed spacer 1, 5.8S ribosomal RNA gene and internal transcribed spacer 2, complete sequence; and 26S ribosomal RNA gene, partial sequence	867	867	100%	0.0	90%	
AY239958.1	Dendrobium ceraula 18S ribosomal RNA gene, partial sequence; internal transcribed spacer 1, 5.8S ribosomal RNA gene and internal transcribed spacer 2, complete sequence; and 26S ribosomal RNA gene, partial sequence	865	865	100%	0.0	90%	
AY239957.1	Dendrobium cauliculimentum 18S ribosomal RNA gene, partial sequence; internal transcribed spacer 1, 5.8S ribosomal RNA gene and internal transcribed spacer 2, complete sequence; and 26S ribosomal RNA gene, partial sequence	865	865	100%	0.0	90%	
AY240004.1	Dendrobium victoriae-reginae 18S ribosomal RNA gene, partial sequence; internal transcribed spacer 1, 5.8S ribosomal RNA gene and internal transcribed spacer 2, complete sequence; and 26S ribosomal RNA gene, partial sequence	854	854	100%	0.0	90%	
AY239991.1	Dendrobium rhododioides 18S ribosomal RNA gene, partial sequence; internal transcribed spacer 1, 5.8S ribosomal RNA gene and internal transcribed spacer 2, complete sequence; and 26S ribosomal RNA gene, partial sequence	843	843	100%	0.0	89%	
AY239986.1	Dendrobium nothofagicola 18S ribosomal RNA gene, partial sequence; internal transcribed spacer 1, 5.8S ribosomal RNA gene and internal transcribed spacer 2, complete sequence; and 26S ribosomal RNA gene, partial sequence	841	841	100%	0.0	89%	
AF521607.1	Dendrobium chameleon internal transcribed spacer 1, 5.8S ribosomal RNA gene, and internal transcribed spacer 2, complete sequence	822	822	95%	0.0	90%	
HM590386.1	Dendrobium miyakei voucher NCHU-D89331201-1020 18S ribosomal RNA gene, partial sequence; internal transcribed spacer 1, 5.8S [illegible]	811	811	100%	0.0	88%	

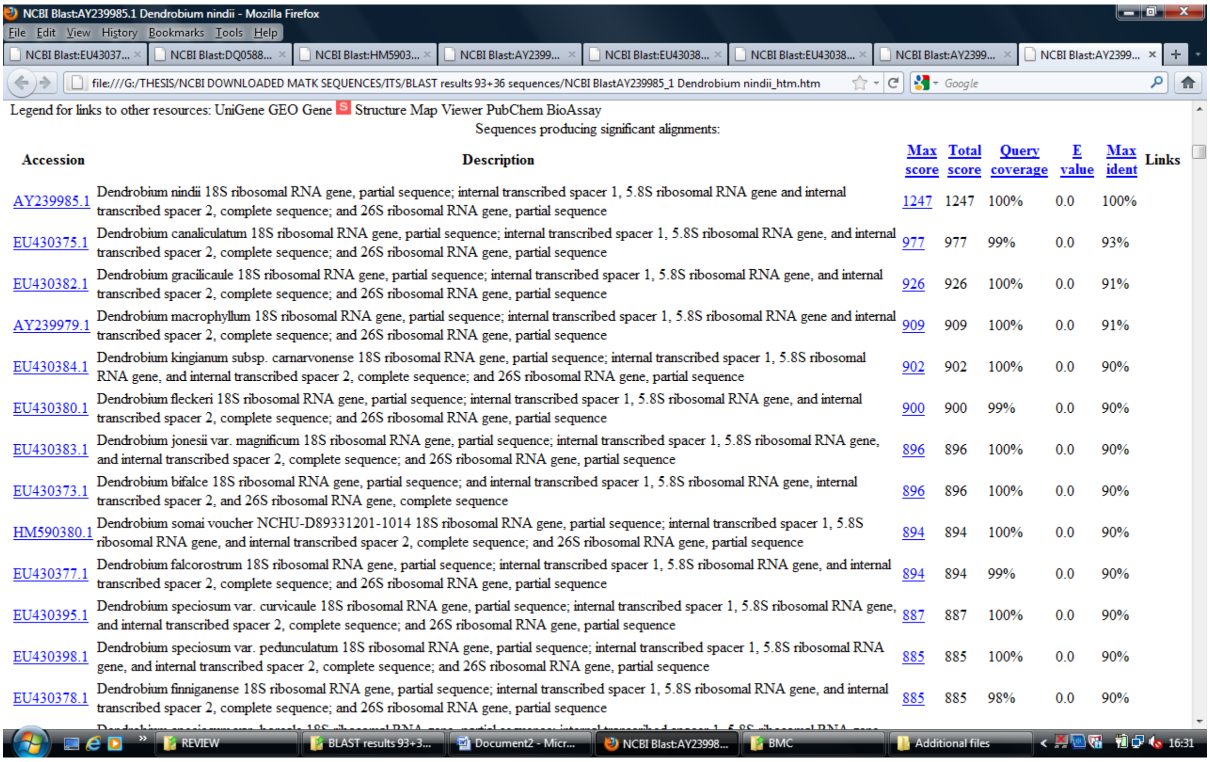

Legend for links to other resources: UniGene GEO Gene Structure Map Viewer PubChem BioAssay

Sequences producing significant alignments:

Accession	Description	Max score	Total score	Query coverage	E value	Max ident	Links
AY239985.1	Dendrobium nindii 18S ribosomal RNA gene, partial sequence; internal transcribed spacer 1, 5.8S ribosomal RNA gene and internal transcribed spacer 2, complete sequence; and 26S ribosomal RNA gene, partial sequence	1247	1247	100%	0.0	100%	
EU430375.1	Dendrobium canaliculatum 18S ribosomal RNA gene, partial sequence; internal transcribed spacer 1, 5.8S ribosomal RNA gene, and internal transcribed spacer 2, complete sequence; and 26S ribosomal RNA gene, partial sequence	977	977	99%	0.0	93%	
EU430382.1	Dendrobium gracilicaule 18S ribosomal RNA gene, partial sequence; internal transcribed spacer 1, 5.8S ribosomal RNA gene, and internal transcribed spacer 2, complete sequence; and 26S ribosomal RNA gene, partial sequence	926	926	100%	0.0	91%	
AY239979.1	Dendrobium macrophyllum 18S ribosomal RNA gene, partial sequence; internal transcribed spacer 1, 5.8S ribosomal RNA gene and internal transcribed spacer 2, complete sequence; and 26S ribosomal RNA gene, partial sequence	909	909	100%	0.0	91%	
EU430384.1	Dendrobium kingianum subsp. carnarvonense 18S ribosomal RNA gene, partial sequence; internal transcribed spacer 1, 5.8S ribosomal RNA gene, and internal transcribed spacer 2, complete sequence; and 26S ribosomal RNA gene, partial sequence	902	902	100%	0.0	90%	
EU430380.1	Dendrobium fleckeri 18S ribosomal RNA gene, partial sequence; internal transcribed spacer 1, 5.8S ribosomal RNA gene, and internal transcribed spacer 2, complete sequence; and 26S ribosomal RNA gene, partial sequence	900	900	99%	0.0	90%	
EU430383.1	Dendrobium jonesii var. magnificum 18S ribosomal RNA gene, partial sequence; internal transcribed spacer 1, 5.8S ribosomal RNA gene, and internal transcribed spacer 2, complete sequence; and 26S ribosomal RNA gene, partial sequence	896	896	100%	0.0	90%	
EU430373.1	Dendrobium bifalce 18S ribosomal RNA gene, partial sequence; and internal transcribed spacer 1, 5.8S ribosomal RNA gene, internal transcribed spacer 2, and 26S ribosomal RNA gene, complete sequence	896	896	100%	0.0	90%	
HM590380.1	Dendrobium somai voucher NCHU-D89331201-1014 18S ribosomal RNA gene, partial sequence; internal transcribed spacer 1, 5.8S ribosomal RNA gene, and internal transcribed spacer 2, complete sequence; and 26S ribosomal RNA gene, partial sequence	894	894	100%	0.0	90%	
EU430377.1	Dendrobium falcorostrum 18S ribosomal RNA gene, partial sequence; internal transcribed spacer 1, 5.8S ribosomal RNA gene, and internal transcribed spacer 2, complete sequence; and 26S ribosomal RNA gene, partial sequence	894	894	99%	0.0	90%	
EU430395.1	Dendrobium speciosum var. curvicaule 18S ribosomal RNA gene, partial sequence; internal transcribed spacer 1, 5.8S ribosomal RNA gene, and internal transcribed spacer 2, complete sequence; and 26S ribosomal RNA gene, partial sequence	887	887	100%	0.0	90%	
EU430398.1	Dendrobium speciosum var. pedunculatum 18S ribosomal RNA gene, partial sequence; internal transcribed spacer 1, 5.8S ribosomal RNA gene, and internal transcribed spacer 2, complete sequence; and 26S ribosomal RNA gene, partial sequence	885	885	100%	0.0	90%	
EU430378.1	Dendrobium finniganense 18S ribosomal RNA gene, partial sequence; internal transcribed spacer 1, 5.8S ribosomal RNA gene, and internal transcribed spacer 2, complete sequence; and 26S ribosomal RNA gene, partial sequence	885	885	98%	0.0	90%	

NCBI Blast:AY239986.1 Dendrobium nothofagicola - Mozilla Firefox

file:///G:/THESIS/NCBI DOWNLOADED MATK SEQUENCES/ITS/BLAST results 93+36 sequences/NCBI BlastAY239986_1 Dendrobium nothofagicola_htm.h

Legend for links to other resources: UniGene GEO Gene S Structure Map Viewer PubChem BioAssay

Sequences producing significant alignments:

Accession	Description	Max score	Total score	Query coverage	E value	Max ident	Links
AY239986.1	Dendrobium nothofagicola 18S ribosomal RNA gene, partial sequence; internal transcribed spacer 1, 5.8S ribosomal RNA gene and internal transcribed spacer 2, complete sequence; and 26S ribosomal RNA gene, partial sequence	1221	1221	100%	0.0	100%	
AY239968.1	Dendrobium fulgidum 18S ribosomal RNA gene, partial sequence; internal transcribed spacer 1, 5.8S ribosomal RNA gene and internal transcribed spacer 2, complete sequence; and 26S ribosomal RNA gene, partial sequence	941	941	100%	0.0	92%	
AY239980.1	Dendrobium mohlianum 18S ribosomal RNA gene, partial sequence; internal transcribed spacer 1, 5.8S ribosomal RNA gene and internal transcribed spacer 2, complete sequence; and 26S ribosomal RNA gene, partial sequence	913	913	100%	0.0	91%	
AY239976.1	Dendrobium lancifolium 18S ribosomal RNA gene, partial sequence; internal transcribed spacer 1, 5.8S ribosomal RNA gene and internal transcribed spacer 2, complete sequence; and 26S ribosomal RNA gene, partial sequence	907	907	100%	0.0	91%	
AY239960.1	Dendrobium chameleon 18S ribosomal RNA gene, partial sequence; internal transcribed spacer 1, 5.8S ribosomal RNA gene and internal transcribed spacer 2, complete sequence; and 26S ribosomal RNA gene, partial sequence	896	896	100%	0.0	91%	
AY240006.1	Dendrobium yeageri 18S ribosomal RNA gene, partial sequence; internal transcribed spacer 1, 5.8S ribosomal RNA gene and internal transcribed spacer 2, complete sequence; and 26S ribosomal RNA gene, partial sequence	891	891	100%	0.0	91%	
AY239994.1	Dendrobium serratilabium 18S ribosomal RNA gene, partial sequence; internal transcribed spacer 1, 5.8S ribosomal RNA gene and internal transcribed spacer 2, complete sequence; and 26S ribosomal RNA gene, partial sequence	885	885	100%	0.0	90%	
AY239954.1	Dendrobium bracteosum 18S ribosomal RNA gene, partial sequence; internal transcribed spacer 1, 5.8S ribosomal RNA gene and internal transcribed spacer 2, complete sequence; and 26S ribosomal RNA gene, partial sequence	885	885	100%	0.0	90%	
HM590385.1	Dendrobium chameleon voucher NCHU-D89331201-1019 18S ribosomal RNA gene, partial sequence; internal transcribed spacer 1, 5.8S ribosomal RNA gene, and internal transcribed spacer 2, complete sequence; and 26S ribosomal RNA gene, partial sequence	880	880	100%	0.0	90%	
AY239958.1	Dendrobium ceraula 18S ribosomal RNA gene, partial sequence; internal transcribed spacer 1, 5.8S ribosomal RNA gene and internal transcribed spacer 2, complete sequence; and 26S ribosomal RNA gene, partial sequence	878	878	100%	0.0	90%	
AY240004.1	Dendrobium victoriae-reginae 18S ribosomal RNA gene, partial sequence; internal transcribed spacer 1, 5.8S ribosomal RNA gene and internal transcribed spacer 2, complete sequence; and 26S ribosomal RNA gene, partial sequence	872	872	100%	0.0	90%	
AY239977.1	Dendrobium lawesii 18S ribosomal RNA gene, partial sequence; internal transcribed spacer 1, 5.8S ribosomal RNA gene and internal transcribed spacer 2, complete sequence; and 26S ribosomal RNA gene, partial sequence	872	872	100%	0.0	90%	
AY239950.1	Dendrobium agathodaemonis 18S ribosomal RNA gene, partial sequence; internal transcribed spacer 1, 5.8S ribosomal RNA gene and internal transcribed spacer 2, complete sequence; and 26S ribosomal RNA gene, partial sequence	872	872	100%	0.0	90%	
[illegible]	[illegible]	[illegible]	[illegible]	[illegible]	[illegible]	[illegible]	

NCBI Blast:AY239987.1 Dendrobium papilio - Mozilla Firefox

file:///G:/THESIS/NCBI DOWNLOADED MATK SEQUENCES/ITS/BLAST results 93+36 sequences/NCBI BlastAY239987_1 Dendrobium papilio_htm.htm

Legend for links to other resources: UniGene GEO Gene S Structure Map Viewer PubChem BioAssay

Sequences producing significant alignments:

Accession	Description	Max score	Total score	Query coverage	E value	Max ident	Links
AY239987.1	Dendrobium papilio 18S ribosomal RNA gene, partial sequence; internal transcribed spacer 1, 5.8S ribosomal RNA gene and internal transcribed spacer 2, complete sequence; and 26S ribosomal RNA gene, partial sequence	1227	1227	100%	0.0	100%	
AY239966.1	Dendrobium fairchildae 18S ribosomal RNA gene, partial sequence; internal transcribed spacer 1, 5.8S ribosomal RNA gene and internal transcribed spacer 2, complete sequence; and 26S ribosomal RNA gene, partial sequence	922	922	100%	0.0	91%	
AY239994.1	Dendrobium serratilabium 18S ribosomal RNA gene, partial sequence; internal transcribed spacer 1, 5.8S ribosomal RNA gene and internal transcribed spacer 2, complete sequence; and 26S ribosomal RNA gene, partial sequence	881	881	100%	0.0	90%	
HM590385.1	Dendrobium chameleon voucher NCHU-D89331201-1019 18S ribosomal RNA gene, partial sequence; internal transcribed spacer 1, 5.8S ribosomal RNA gene, and internal transcribed spacer 2, complete sequence; and 26S ribosomal RNA gene, partial sequence	867	867	100%	0.0	90%	
AY240006.1	Dendrobium yeageri 18S ribosomal RNA gene, partial sequence; internal transcribed spacer 1, 5.8S ribosomal RNA gene and internal transcribed spacer 2, complete sequence; and 26S ribosomal RNA gene, partial sequence	859	859	100%	0.0	90%	
AY239960.1	Dendrobium chameleon 18S ribosomal RNA gene, partial sequence; internal transcribed spacer 1, 5.8S ribosomal RNA gene and internal transcribed spacer 2, complete sequence; and 26S ribosomal RNA gene, partial sequence	859	859	100%	0.0	90%	
AY239958.1	Dendrobium ceraula 18S ribosomal RNA gene, partial sequence; internal transcribed spacer 1, 5.8S ribosomal RNA gene and internal transcribed spacer 2, complete sequence; and 26S ribosomal RNA gene, partial sequence	859	859	100%	0.0	90%	
AY240004.1	Dendrobium victoriae-reginae 18S ribosomal RNA gene, partial sequence; internal transcribed spacer 1, 5.8S ribosomal RNA gene and internal transcribed spacer 2, complete sequence; and 26S ribosomal RNA gene, partial sequence	854	854	100%	0.0	90%	
HM590386.1	Dendrobium miyakei voucher NCHU-D89331201-1020 18S ribosomal RNA gene, partial sequence; internal transcribed spacer 1, 5.8S ribosomal RNA gene, and internal transcribed spacer 2, complete sequence; and 26S ribosomal RNA gene, partial sequence	828	828	100%	0.0	89%	
AY239970.1	Dendrobium goldschmidtianum 18S ribosomal RNA gene, partial sequence; internal transcribed spacer 1, 5.8S ribosomal RNA gene and internal transcribed spacer 2, complete sequence; and 26S ribosomal RNA gene, partial sequence	828	828	100%	0.0	89%	
AY239976.1	Dendrobium lancifolium 18S ribosomal RNA gene, partial sequence; internal transcribed spacer 1, 5.8S ribosomal RNA gene and internal transcribed spacer 2, complete sequence; and 26S ribosomal RNA gene, partial sequence	815	815	100%	0.0	88%	
AF521607.1	Dendrobium chameleon internal transcribed spacer 1, 5.8S ribosomal RNA gene, and internal transcribed spacer 2, complete sequence	815	815	94%	0.0	90%	
AF521614.1	Dendrobium miyakei internal transcribed spacer 1, 5.8S ribosomal RNA gene, and internal transcribed spacer 2, complete sequence	793	793	93%	0.0	89%	
EU840695.1	Dendrobium goldschmidtianum internal transcribed spacer 1, 5.8S ribosomal RNA gene, and internal transcribed spacer 2, complete sequence	787	787	93%	0.0	89%	

NCBI Blast:EF629324.1 Dendrobium parciflorum - Mozilla Firefox

file:///G:/THESIS/NCBI DOWNLOADED MATK SEQUENCES/ITS/BLAST results 93+36 sequences/NCBI BlastEF629324_1 Dendrobium parciflorum_htm.htm

Legend for links to other resources: UniGene GEO Gene Structure Map Viewer PubChem BioAssay

Sequences producing significant alignments:

Accession	Description	Max score	Total score	Query coverage	E value	Max ident	Links
EF629324.1	Dendrobium parciflorum internal transcribed spacer 1, partial sequence; 5.8S ribosomal RNA gene, complete sequence; and internal transcribed spacer 2, partial sequence	1175	1175	100%	0.0	100%	
AF362038.1	Dendrobium hancockii isolate Henan internal transcribed spacer 1, partial sequence; 5.8S ribosomal RNA gene, complete sequence; and internal transcribed spacer 2, partial sequence >gb|FJ384726.1| Dendrobium hancockii strain X2 internal transcribed spacer 1, partial sequence; 5.8S ribosomal RNA gene, complete sequence; and internal transcribed spacer 2, partial sequence	1166	1166	100%	0.0	99%	
FJ384725.1	Dendrobium hancockii strain X1 internal transcribed spacer 1, partial sequence; 5.8S ribosomal RNA gene, complete sequence; and internal transcribed spacer 2, partial sequence	1160	1160	100%	0.0	99%	
AF362025.1	Dendrobium hancockii internal transcribed spacer 1, partial sequence; 5.8S ribosomal RNA gene, complete sequence; and internal transcribed spacer 2, partial sequence	985	985	100%	0.0	94%	
DQ058787.1	Dendrobium hancockii voucher XY-GZ03-1 internal transcribed spacer 1, 5.8S ribosomal RNA gene, and internal transcribed spacer 2, complete sequence	983	983	100%	0.0	94%	
HM590377.1	Dendrobium hancockii voucher NCHU-D89331201-1011 18S ribosomal RNA gene, partial sequence; internal transcribed spacer 1, 5.8S ribosomal RNA gene, and internal transcribed spacer 2, complete sequence; and 26S ribosomal RNA gene, partial sequence	979	979	100%	0.0	94%	
EU003120.1	Dendrobium hancockii voucher CMU DH 0613 internal transcribed spacer 1, partial sequence; 5.8S ribosomal RNA gene, complete sequence; and internal transcribed spacer 2, partial sequence	883	883	100%	0.0	91%	
DQ058788.1	Dendrobium dixanthum voucher HH-JL03-1 internal transcribed spacer 1, 5.8S ribosomal RNA gene, and internal transcribed spacer 2, complete sequence	802	802	100%	0.0	89%	
GU339103.1	Dendrobium dixanthum internal transcribed spacer 1, partial sequence; 5.8S ribosomal RNA gene, complete sequence; and internal transcribed spacer 2, partial sequence	797	797	100%	0.0	89%	
EU477500.1	Dendrobium brymerianum internal transcribed spacer 1, partial sequence; 5.8S ribosomal RNA gene and internal transcribed spacer 2, complete sequence; and 28S ribosomal RNA gene, partial sequence	769	769	100%	0.0	88%	
AF362036.1	Dendrobium brymerianum internal transcribed spacer 1, partial sequence; 5.8S ribosomal RNA gene, complete sequence; and internal transcribed spacer 2, partial sequence	763	763	100%	0.0	88%	
AF362040.1	Dendrobium aurantiacum var. denneanum internal transcribed spacer 1, partial sequence; 5.8S ribosomal RNA gene, complete sequence; and internal transcribed spacer 2, partial sequence >gb|FJ384731.1| Dendrobium aurantiacum var. denneanum strain D3 internal transcribed spacer 1, partial sequence; 5.8S ribosomal RNA gene, complete sequence; and internal transcribed spacer 2, partial sequence	752	752	100%	0.0	88%	
[illegible]	Dendrobium densiflorum internal transcribed spacer 1, partial sequence; 5.8S ribosomal RNA gene, complete sequence; and internal [illegible]	[illegible]	[illegible]	[illegible]	[illegible]	[illegible]	

NCBI Blast:GU339115.1 Dendrobium pendulum - Mozilla Firefox

file:///G:/THESIS/NCBI DOWNLOADED MATK SEQUENCES/ITS/BLAST results 93+36 sequences/NCBI BlastGU339115_1 Dendrobium pendulum_htm.htm

Legend for links to other resources: UniGene GEO Gene Structure Map Viewer PubChem BioAssay

Sequences producing significant alignments:

Accession	Description	Max score	Total score	Query coverage	E value	Max ident	Links
GU339115.1	Dendrobium pendulum internal transcribed spacer 1, partial sequence; 5.8S ribosomal RNA gene, complete sequence; and internal transcribed spacer 2, partial sequence	1177	1177	100%	0.0	100%	
DQ058791.1	Dendrobium pendulum voucher ZJ-0312-1 internal transcribed spacer 1, 5.8S ribosomal RNA gene, and internal transcribed spacer 2, complete sequence	1160	1160	100%	0.0	99%	
EU477508.1	Dendrobium pendulum internal transcribed spacer 1, partial sequence; 5.8S ribosomal RNA gene and internal transcribed spacer 2, complete sequence; and 28S ribosomal RNA gene, partial sequence	1099	1099	100%	0.0	97%	
AF362912.1	Dendrobium pendulum internal transcribed spacer 1, partial sequence; 5.8S ribosomal RNA gene, complete sequence; and internal transcribed spacer 2, partial sequence	1062	1062	100%	0.0	96%	
HM054544.1	Dendrobium amoenum voucher SBB-0560 internal transcribed spacer 1, partial sequence; 5.8S ribosomal RNA gene and internal transcribed spacer 2, complete sequence; and 26S ribosomal RNA gene, partial sequence	917	917	100%	0.0	92%	
DQ058789.1	Dendrobium wardianum voucher DBQ-JL04-01 internal transcribed spacer 1, 5.8S ribosomal RNA gene, and internal transcribed spacer 2, complete sequence	917	917	100%	0.0	92%	
HM054538.1	Dendrobium amoenum voucher SBB-0140 internal transcribed spacer 1, partial sequence; 5.8S ribosomal RNA gene and internal transcribed spacer 2, complete sequence; and 26S ribosomal RNA gene, partial sequence	913	913	100%	0.0	92%	
HM054539.1	Dendrobium amoenum voucher SBB-0142 internal transcribed spacer 1, partial sequence; 5.8S ribosomal RNA gene and internal transcribed spacer 2, complete sequence; and 26S ribosomal RNA gene, partial sequence	911	911	100%	0.0	92%	
HM054540.1	Dendrobium amoenum voucher SBB-0247 internal transcribed spacer 1, partial sequence; 5.8S ribosomal RNA gene and internal transcribed spacer 2, complete sequence; and 26S ribosomal RNA gene, partial sequence	911	911	100%	0.0	92%	
HM054534.1	Dendrobium amoenum voucher SBB-0135 internal transcribed spacer 1, partial sequence; 5.8S ribosomal RNA gene and internal transcribed spacer 2, complete sequence; and 26S ribosomal RNA gene, partial sequence	911	911	99%	0.0	92%	
HM054536.1	Dendrobium amoenum voucher SBB-0138 internal transcribed spacer 1, partial sequence; 5.8S ribosomal RNA gene and internal transcribed spacer 2, complete sequence; and 26S ribosomal RNA gene, partial sequence	911	911	99%	0.0	92%	
HM054537.1	Dendrobium amoenum voucher SBB-0139 internal transcribed spacer 1, partial sequence; 5.8S ribosomal RNA gene and internal transcribed spacer 2, complete sequence; and 26S ribosomal RNA gene, partial sequence	909	909	99%	0.0	92%	
HM054535.1	Dendrobium amoenum voucher SBB-0137 internal transcribed spacer 1, partial sequence; 5.8S ribosomal RNA gene and internal transcribed spacer 2, complete sequence; and 26S ribosomal RNA gene, partial sequence	909	909	99%	0.0	92%	
[illegible]	Dendrobium gratiosissimum voucher BQ-JL0401-1 internal transcribed spacer 1, 5.8S ribosomal RNA gene, and internal transcribed spacer 2, [illegible]	[illegible]	[illegible]	[illegible]	[illegible]	[illegible]	

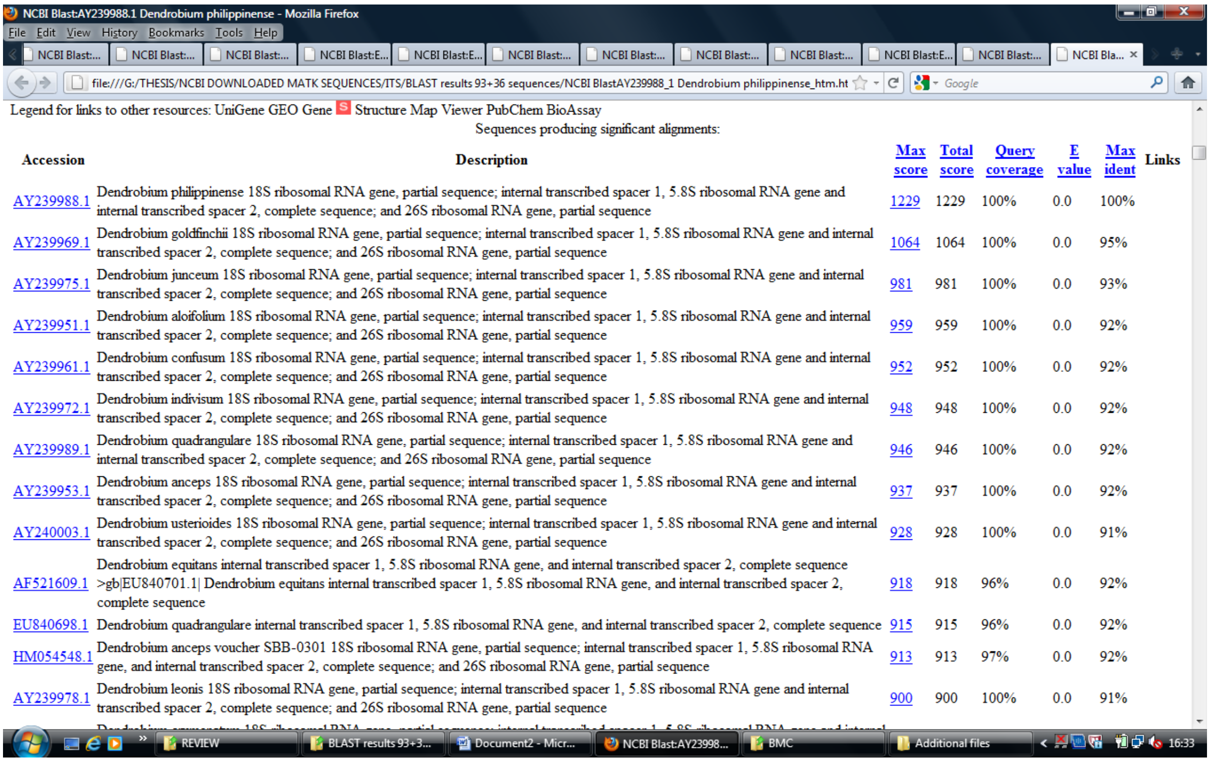

Legend for links to other resources: UniGene GEO Gene S Structure Map Viewer PubChem BioAssay

Sequences producing significant alignments:

Accession	Description	Max score	Total score	Query coverage	E value	Max ident	Links
AY239988.1	Dendrobium philippinense 18S ribosomal RNA gene, partial sequence; internal transcribed spacer 1, 5.8S ribosomal RNA gene and internal transcribed spacer 2, complete sequence; and 26S ribosomal RNA gene, partial sequence	1229	1229	100%	0.0	100%	
AY239969.1	Dendrobium goldfinchii 18S ribosomal RNA gene, partial sequence; internal transcribed spacer 1, 5.8S ribosomal RNA gene and internal transcribed spacer 2, complete sequence; and 26S ribosomal RNA gene, partial sequence	1064	1064	100%	0.0	95%	
AY239975.1	Dendrobium junceum 18S ribosomal RNA gene, partial sequence; internal transcribed spacer 1, 5.8S ribosomal RNA gene and internal transcribed spacer 2, complete sequence; and 26S ribosomal RNA gene, partial sequence	981	981	100%	0.0	93%	
AY239951.1	Dendrobium aloifolium 18S ribosomal RNA gene, partial sequence; internal transcribed spacer 1, 5.8S ribosomal RNA gene and internal transcribed spacer 2, complete sequence; and 26S ribosomal RNA gene, partial sequence	959	959	100%	0.0	92%	
AY239961.1	Dendrobium confusum 18S ribosomal RNA gene, partial sequence; internal transcribed spacer 1, 5.8S ribosomal RNA gene and internal transcribed spacer 2, complete sequence; and 26S ribosomal RNA gene, partial sequence	952	952	100%	0.0	92%	
AY239972.1	Dendrobium indivisum 18S ribosomal RNA gene, partial sequence; internal transcribed spacer 1, 5.8S ribosomal RNA gene and internal transcribed spacer 2, complete sequence; and 26S ribosomal RNA gene, partial sequence	948	948	100%	0.0	92%	
AY239989.1	Dendrobium quadrangulare 18S ribosomal RNA gene, partial sequence; internal transcribed spacer 1, 5.8S ribosomal RNA gene and internal transcribed spacer 2, complete sequence; and 26S ribosomal RNA gene, partial sequence	946	946	100%	0.0	92%	
AY239953.1	Dendrobium anceps 18S ribosomal RNA gene, partial sequence; internal transcribed spacer 1, 5.8S ribosomal RNA gene and internal transcribed spacer 2, complete sequence; and 26S ribosomal RNA gene, partial sequence	937	937	100%	0.0	92%	
AY240003.1	Dendrobium usterioides 18S ribosomal RNA gene, partial sequence; internal transcribed spacer 1, 5.8S ribosomal RNA gene and internal transcribed spacer 2, complete sequence; and 26S ribosomal RNA gene, partial sequence	928	928	100%	0.0	91%	
AF521609.1	Dendrobium equitans internal transcribed spacer 1, 5.8S ribosomal RNA gene, and internal transcribed spacer 2, complete sequence >gb|EU840701.1| Dendrobium equitans internal transcribed spacer 1, 5.8S ribosomal RNA gene, and internal transcribed spacer 2, complete sequence	918	918	96%	0.0	92%	
EU840698.1	Dendrobium quadrangulare internal transcribed spacer 1, 5.8S ribosomal RNA gene, and internal transcribed spacer 2, complete sequence	915	915	96%	0.0	92%	
HM054548.1	Dendrobium anceps voucher SBB-0301 18S ribosomal RNA gene, partial sequence; internal transcribed spacer 1, 5.8S ribosomal RNA gene, and internal transcribed spacer 2, complete sequence; and 26S ribosomal RNA gene, partial sequence	913	913	97%	0.0	92%	
AY239978.1	Dendrobium leonis 18S ribosomal RNA gene, partial sequence; internal transcribed spacer 1, 5.8S ribosomal RNA gene and internal transcribed spacer 2, complete sequence; and 26S ribosomal RNA gene, partial sequence	900	900	100%	0.0	91%	

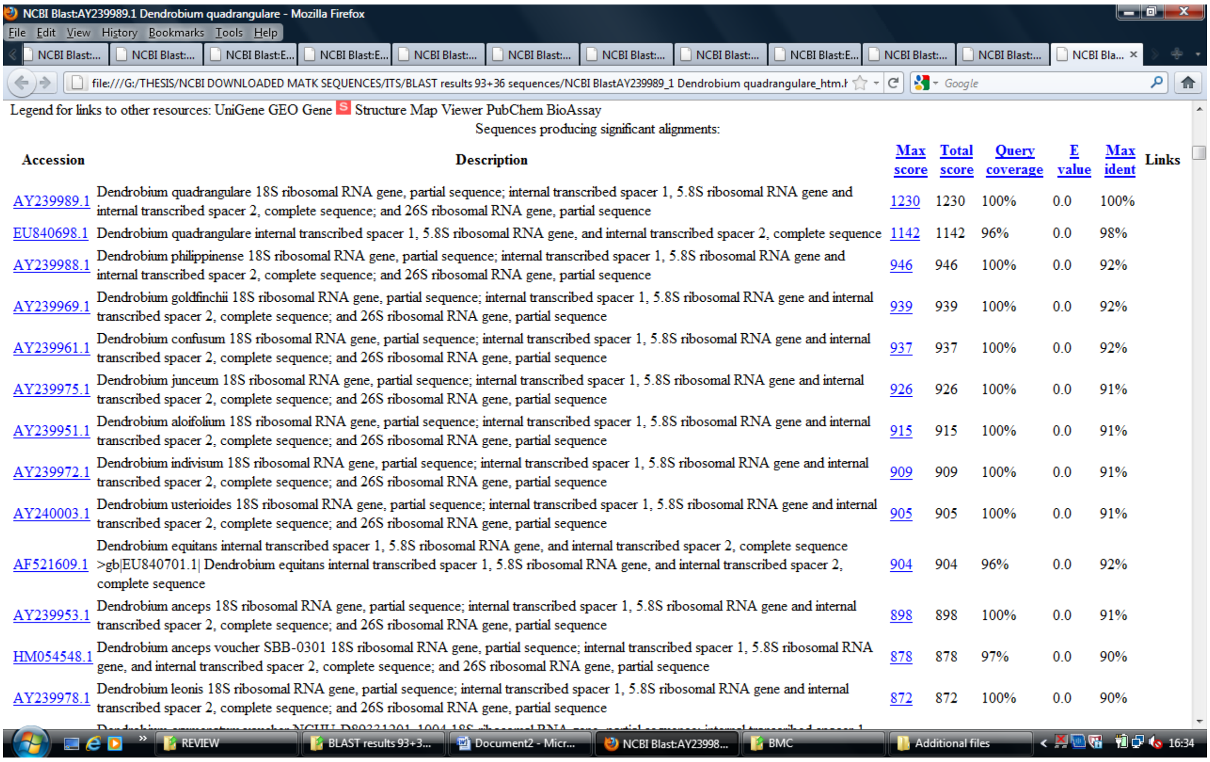

Legend for links to other resources: UniGene GEO Gene S Structure Map Viewer PubChem BioAssay

Sequences producing significant alignments:

Accession	Description	Max score	Total score	Query coverage	E value	Max ident	Links
AY239989.1	Dendrobium quadrangulare 18S ribosomal RNA gene, partial sequence; internal transcribed spacer 1, 5.8S ribosomal RNA gene and internal transcribed spacer 2, complete sequence; and 26S ribosomal RNA gene, partial sequence	1230	1230	100%	0.0	100%	
EU840698.1	Dendrobium quadrangulare internal transcribed spacer 1, 5.8S ribosomal RNA gene, and internal transcribed spacer 2, complete sequence	1142	1142	96%	0.0	98%	
AY239988.1	Dendrobium philippinense 18S ribosomal RNA gene, partial sequence; internal transcribed spacer 1, 5.8S ribosomal RNA gene and internal transcribed spacer 2, complete sequence; and 26S ribosomal RNA gene, partial sequence	946	946	100%	0.0	92%	
AY239969.1	Dendrobium goldfinchii 18S ribosomal RNA gene, partial sequence; internal transcribed spacer 1, 5.8S ribosomal RNA gene and internal transcribed spacer 2, complete sequence; and 26S ribosomal RNA gene, partial sequence	939	939	100%	0.0	92%	
AY239961.1	Dendrobium confusum 18S ribosomal RNA gene, partial sequence; internal transcribed spacer 1, 5.8S ribosomal RNA gene and internal transcribed spacer 2, complete sequence; and 26S ribosomal RNA gene, partial sequence	937	937	100%	0.0	92%	
AY239975.1	Dendrobium junceum 18S ribosomal RNA gene, partial sequence; internal transcribed spacer 1, 5.8S ribosomal RNA gene and internal transcribed spacer 2, complete sequence; and 26S ribosomal RNA gene, partial sequence	926	926	100%	0.0	91%	
AY239951.1	Dendrobium aloifolium 18S ribosomal RNA gene, partial sequence; internal transcribed spacer 1, 5.8S ribosomal RNA gene and internal transcribed spacer 2, complete sequence; and 26S ribosomal RNA gene, partial sequence	915	915	100%	0.0	91%	
AY239972.1	Dendrobium indivisum 18S ribosomal RNA gene, partial sequence; internal transcribed spacer 1, 5.8S ribosomal RNA gene and internal transcribed spacer 2, complete sequence; and 26S ribosomal RNA gene, partial sequence	909	909	100%	0.0	91%	
AY240003.1	Dendrobium usterioides 18S ribosomal RNA gene, partial sequence; internal transcribed spacer 1, 5.8S ribosomal RNA gene and internal transcribed spacer 2, complete sequence; and 26S ribosomal RNA gene, partial sequence	905	905	100%	0.0	91%	
AF521609.1	Dendrobium equitans internal transcribed spacer 1, 5.8S ribosomal RNA gene, and internal transcribed spacer 2, complete sequence >gb|EU840701.1| Dendrobium equitans internal transcribed spacer 1, 5.8S ribosomal RNA gene, and internal transcribed spacer 2, complete sequence	904	904	96%	0.0	92%	
AY239953.1	Dendrobium anceps 18S ribosomal RNA gene, partial sequence; internal transcribed spacer 1, 5.8S ribosomal RNA gene and internal transcribed spacer 2, complete sequence; and 26S ribosomal RNA gene, partial sequence	898	898	100%	0.0	91%	
HM054548.1	Dendrobium anceps voucher SBB-0301 18S ribosomal RNA gene, partial sequence; internal transcribed spacer 1, 5.8S ribosomal RNA gene, and internal transcribed spacer 2, complete sequence; and 26S ribosomal RNA gene, partial sequence	878	878	97%	0.0	90%	
AY239978.1	Dendrobium leonis 18S ribosomal RNA gene, partial sequence; internal transcribed spacer 1, 5.8S ribosomal RNA gene and internal transcribed spacer 2, complete sequence; and 26S ribosomal RNA gene, partial sequence	872	872	100%	0.0	90%	

NCBI Blast:EU430389.1 Dendrobium racemosum - Mozilla Firefox

file:///G:/THESIS/NCBI DOWNLOADED MATK SEQUENCES/ITS/BLAST results 93+36 sequences/NCBI BlastEU430389_1 Dendrobium racemosum_htm.htm

Legend for links to other resources: UniGene GEO Gene Structure Map Viewer PubChem BioAssay

Sequences producing significant alignments:

Accession	Description	Max score	Total score	Query coverage	E value	Max ident	Links
EU430389.1	Dendrobium racemosum 18S ribosomal RNA gene, partial sequence; internal transcribed spacer 1, 5.8S ribosomal RNA gene, and internal transcribed spacer 2, complete sequence; and 26S ribosomal RNA gene, partial sequence	1304	1304	100%	0.0	100%	
AF321593.1	Dockrillia linguiforme internal transcribed spacer 1, 5.8S ribosomal RNA gene, and internal transcribed spacer 2, complete sequence	1138	1138	100%	0.0	95%	
EU430382.1	Dendrobium gracilicaule 18S ribosomal RNA gene, partial sequence; internal transcribed spacer 1, 5.8S ribosomal RNA gene, and internal transcribed spacer 2, complete sequence; and 26S ribosomal RNA gene, partial sequence	1092	1092	99%	0.0	94%	
EU430384.1	Dendrobium kingianum subsp. carnarvonense 18S ribosomal RNA gene, partial sequence; internal transcribed spacer 1, 5.8S ribosomal RNA gene, and internal transcribed spacer 2, complete sequence; and 26S ribosomal RNA gene, partial sequence	1079	1079	100%	0.0	94%	
EU430395.1	Dendrobium speciosum var. curvicaule 18S ribosomal RNA gene, partial sequence; internal transcribed spacer 1, 5.8S ribosomal RNA gene, and internal transcribed spacer 2, complete sequence; and 26S ribosomal RNA gene, partial sequence	1070	1070	100%	0.0	94%	
EU430383.1	Dendrobium jonesii var. magnificum 18S ribosomal RNA gene, partial sequence; internal transcribed spacer 1, 5.8S ribosomal RNA gene, and internal transcribed spacer 2, complete sequence; and 26S ribosomal RNA gene, partial sequence	1062	1062	100%	0.0	93%	
EU430398.1	Dendrobium speciosum var. pedunculatum 18S ribosomal RNA gene, partial sequence; internal transcribed spacer 1, 5.8S ribosomal RNA gene, and internal transcribed spacer 2, complete sequence; and 26S ribosomal RNA gene, partial sequence	1057	1057	100%	0.0	93%	
EU430377.1	Dendrobium falcorostrum 18S ribosomal RNA gene, partial sequence; internal transcribed spacer 1, 5.8S ribosomal RNA gene, and internal transcribed spacer 2, complete sequence; and 26S ribosomal RNA gene, partial sequence	1051	1051	95%	0.0	94%	
EU430385.1	Dendrobium kingianum var. pulcherrimum 18S ribosomal RNA gene, partial sequence; internal transcribed spacer 1, 5.8S ribosomal RNA gene, and internal transcribed spacer 2, complete sequence; and 26S ribosomal RNA gene, partial sequence	1033	1033	100%	0.0	93%	
AF321594.1	Dockrillia pugioniformis internal transcribed spacer 1, 5.8S ribosomal RNA gene, and internal transcribed spacer 2, complete sequence	1031	1031	100%	0.0	93%	
EU430392.1	Dendrobium speciosum var. boreale 18S ribosomal RNA gene, partial sequence; internal transcribed spacer 1, 5.8S ribosomal RNA gene, and internal transcribed spacer 2, complete sequence; and 26S ribosomal RNA gene, partial sequence	1027	1027	96%	0.0	93%	
EU430386.1	Dendrobium kingianum var. kingianum 18S ribosomal RNA gene, partial sequence; internal transcribed spacer 1, 5.8S ribosomal RNA gene, and internal transcribed spacer 2, complete sequence; and 26S ribosomal RNA gene, partial sequence	1020	1020	98%	0.0	93%	
EU430380.1	Dendrobium fleckeri 18S ribosomal RNA gene, partial sequence; internal transcribed spacer 1, 5.8S ribosomal RNA gene, and internal transcribed spacer 2, complete sequence; and 26S ribosomal RNA gene, partial sequence	1013	1013	96%	0.0	93%	
EU430397.1	Dendrobium speciosum var. hillii 18S ribosomal RNA gene, partial sequence; internal transcribed spacer 1, 5.8S ribosomal RNA gene, and internal transcribed spacer 2, complete sequence; and 26S ribosomal RNA gene, partial sequence	1005	1005	100%	0.0	92%	

NCBI Blast:AY239991.1 Dendrobium rhododioides - Mozilla Firefox

file:///G:/THESIS/NCBI DOWNLOADED MATK SEQUENCES/ITS/BLAST results 93+36 sequences/NCBI BlastAY239991_1 Dendrobium rhododioides_htm.ht

Legend for links to other resources: UniGene GEO Gene Structure Map Viewer PubChem BioAssay

Sequences producing significant alignments:

Accession	Description	Max score	Total score	Query coverage	E value	Max ident	Links
AY239991.1	Dendrobium rhododioides 18S ribosomal RNA gene, partial sequence; internal transcribed spacer 1, 5.8S ribosomal RNA gene and internal transcribed spacer 2, complete sequence; and 26S ribosomal RNA gene, partial sequence	1225	1225	100%	0.0	100%	
AY239957.1	Dendrobium cauliculimentum 18S ribosomal RNA gene, partial sequence; internal transcribed spacer 1, 5.8S ribosomal RNA gene and internal transcribed spacer 2, complete sequence; and 26S ribosomal RNA gene, partial sequence	1203	1203	100%	0.0	99%	
AY239994.1	Dendrobium serratilabium 18S ribosomal RNA gene, partial sequence; internal transcribed spacer 1, 5.8S ribosomal RNA gene and internal transcribed spacer 2, complete sequence; and 26S ribosomal RNA gene, partial sequence	904	904	100%	0.0	91%	
AY240006.1	Dendrobium yeageri 18S ribosomal RNA gene, partial sequence; internal transcribed spacer 1, 5.8S ribosomal RNA gene and internal transcribed spacer 2, complete sequence; and 26S ribosomal RNA gene, partial sequence	893	893	100%	0.0	91%	
AY239960.1	Dendrobium chameleon 18S ribosomal RNA gene, partial sequence; internal transcribed spacer 1, 5.8S ribosomal RNA gene and internal transcribed spacer 2, complete sequence; and 26S ribosomal RNA gene, partial sequence	893	893	100%	0.0	91%	
AY239976.1	Dendrobium lancifolium 18S ribosomal RNA gene, partial sequence; internal transcribed spacer 1, 5.8S ribosomal RNA gene and internal transcribed spacer 2, complete sequence; and 26S ribosomal RNA gene, partial sequence	881	881	100%	0.0	90%	
AY239958.1	Dendrobium ceraula 18S ribosomal RNA gene, partial sequence; internal transcribed spacer 1, 5.8S ribosomal RNA gene and internal transcribed spacer 2, complete sequence; and 26S ribosomal RNA gene, partial sequence	880	880	100%	0.0	90%	
AY240004.1	Dendrobium victoriae-reginae 18S ribosomal RNA gene, partial sequence; internal transcribed spacer 1, 5.8S ribosomal RNA gene and internal transcribed spacer 2, complete sequence; and 26S ribosomal RNA gene, partial sequence	874	874	100%	0.0	90%	
AF521607.1	Dendrobium chameleon internal transcribed spacer 1, 5.8S ribosomal RNA gene, and internal transcribed spacer 2, complete sequence	856	856	96%	0.0	90%	
AY239970.1	Dendrobium goldschmidtianum 18S ribosomal RNA gene, partial sequence; internal transcribed spacer 1, 5.8S ribosomal RNA gene and internal transcribed spacer 2, complete sequence; and 26S ribosomal RNA gene, partial sequence	845	845	100%	0.0	89%	
AY239982.1	Dendrobium morrisonii 18S ribosomal RNA gene, partial sequence; internal transcribed spacer 1, 5.8S ribosomal RNA gene and internal transcribed spacer 2, complete sequence; and 26S ribosomal RNA gene, partial sequence	843	843	100%	0.0	89%	
AY239968.1	Dendrobium fulgidum 18S ribosomal RNA gene, partial sequence; internal transcribed spacer 1, 5.8S ribosomal RNA gene and internal transcribed spacer 2, complete sequence; and 26S ribosomal RNA gene, partial sequence	826	826	100%	0.0	89%	
AY239980.1	Dendrobium mohlianum 18S ribosomal RNA gene, partial sequence; internal transcribed spacer 1, 5.8S ribosomal RNA gene and internal transcribed spacer 2, complete sequence; and 26S ribosomal RNA gene, partial sequence	821	821	100%	0.0	89%	
AF521614.1	Dendrobium miyakei internal transcribed spacer 1, 5.8S ribosomal RNA gene, and internal transcribed spacer 2, complete sequence	819	819	94%	0.0	90%	
[illegible]	[illegible]						

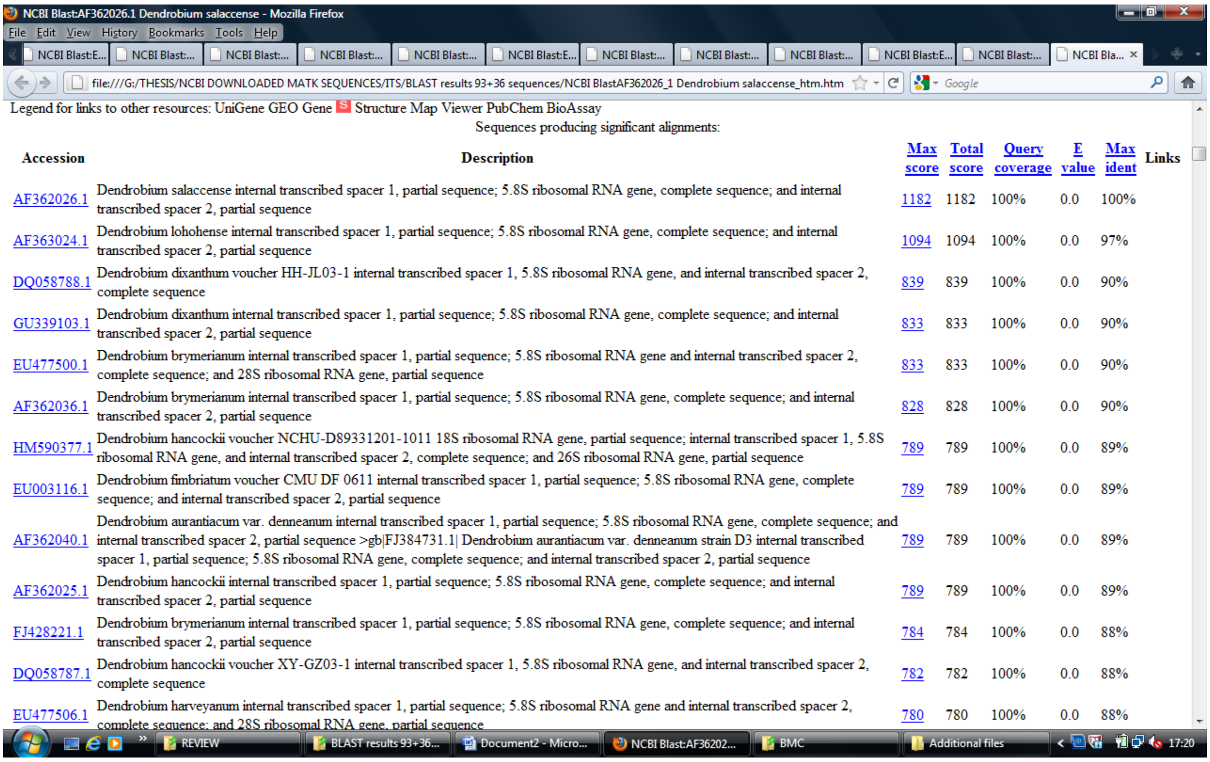

Legend for links to other resources: UniGene GEO Gene Structure Map Viewer PubChem BioAssay

Sequences producing significant alignments:

Accession	Description	Max score	Total score	Query coverage	E value	Max ident	Links
AF362026.1	Dendrobium salaccense internal transcribed spacer 1, partial sequence; 5.8S ribosomal RNA gene, complete sequence; and internal transcribed spacer 2, partial sequence	1182	1182	100%	0.0	100%	
AF363024.1	Dendrobium lohohense internal transcribed spacer 1, partial sequence; 5.8S ribosomal RNA gene, complete sequence; and internal transcribed spacer 2, partial sequence	1094	1094	100%	0.0	97%	
DQ058788.1	Dendrobium dixanthum voucher HH-JL03-1 internal transcribed spacer 1, 5.8S ribosomal RNA gene, and internal transcribed spacer 2, complete sequence	839	839	100%	0.0	90%	
GU339103.1	Dendrobium dixanthum internal transcribed spacer 1, partial sequence; 5.8S ribosomal RNA gene, complete sequence; and internal transcribed spacer 2, partial sequence	833	833	100%	0.0	90%	
EU477500.1	Dendrobium brymerianum internal transcribed spacer 1, partial sequence; 5.8S ribosomal RNA gene and internal transcribed spacer 2, complete sequence; and 28S ribosomal RNA gene, partial sequence	833	833	100%	0.0	90%	
AF362036.1	Dendrobium brymerianum internal transcribed spacer 1, partial sequence; 5.8S ribosomal RNA gene, complete sequence; and internal transcribed spacer 2, partial sequence	828	828	100%	0.0	90%	
HM590377.1	Dendrobium hancockii voucher NCHU-D89331201-1011 18S ribosomal RNA gene, partial sequence; internal transcribed spacer 1, 5.8S ribosomal RNA gene, and internal transcribed spacer 2, complete sequence; and 26S ribosomal RNA gene, partial sequence	789	789	100%	0.0	89%	
EU003116.1	Dendrobium fimbriatum voucher CMU DF 0611 internal transcribed spacer 1, partial sequence; 5.8S ribosomal RNA gene, complete sequence; and internal transcribed spacer 2, partial sequence	789	789	100%	0.0	89%	
AF362040.1	Dendrobium aurantiacum var. denneanum internal transcribed spacer 1, partial sequence; 5.8S ribosomal RNA gene, complete sequence; and internal transcribed spacer 2, partial sequence >gb|FJ384731.1| Dendrobium aurantiacum var. denneanum strain D3 internal transcribed spacer 1, partial sequence; 5.8S ribosomal RNA gene, complete sequence; and internal transcribed spacer 2, partial sequence	789	789	100%	0.0	89%	
AF362025.1	Dendrobium hancockii internal transcribed spacer 1, partial sequence; 5.8S ribosomal RNA gene, complete sequence; and internal transcribed spacer 2, partial sequence	789	789	100%	0.0	89%	
FJ428221.1	Dendrobium brymerianum internal transcribed spacer 1, partial sequence; 5.8S ribosomal RNA gene, complete sequence; and internal transcribed spacer 2, partial sequence	784	784	100%	0.0	88%	
DQ058787.1	Dendrobium hancockii voucher XY-GZ03-1 internal transcribed spacer 1, 5.8S ribosomal RNA gene, and internal transcribed spacer 2, complete sequence	782	782	100%	0.0	88%	
EU477506.1	Dendrobium harveyanum internal transcribed spacer 1, partial sequence; 5.8S ribosomal RNA gene and internal transcribed spacer 2, complete sequence; and 28S ribosomal RNA gene, partial sequence	780	780	100%	0.0	88%	

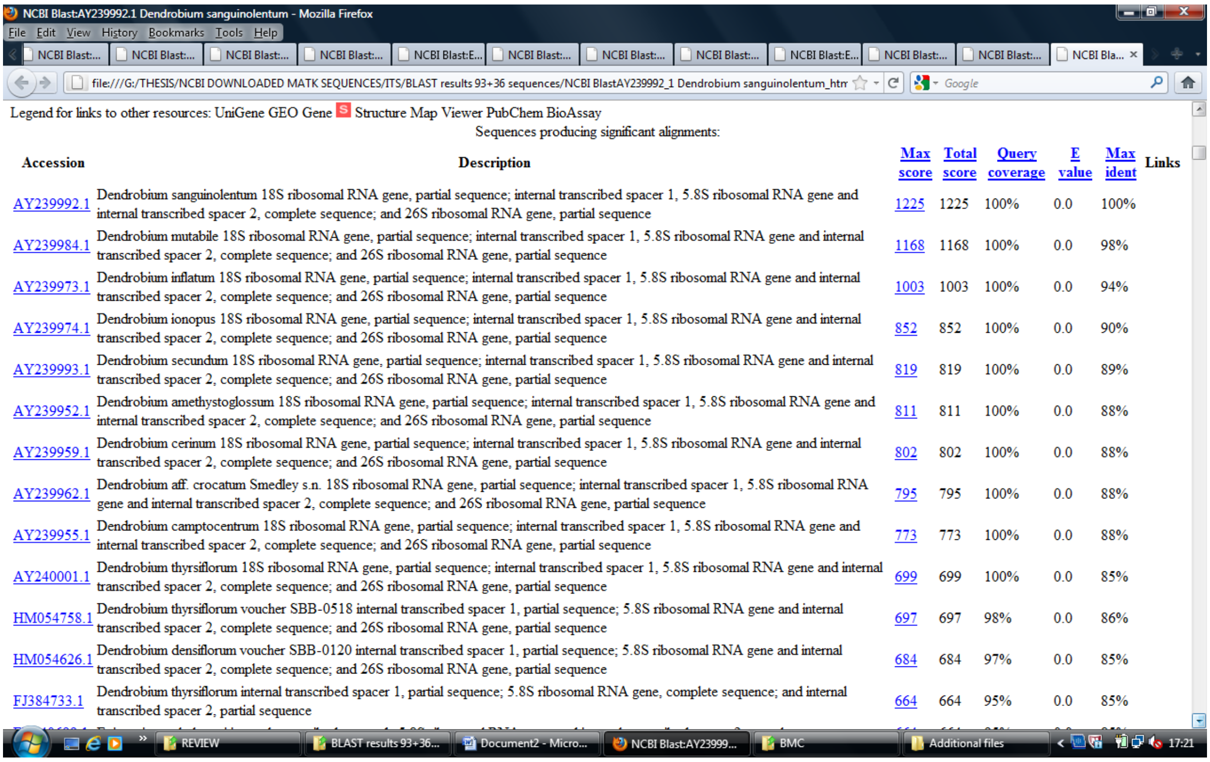

Legend for links to other resources: UniGene GEO Gene Structure Map Viewer PubChem BioAssay

Sequences producing significant alignments:

Accession	Description	Max score	Total score	Query coverage	E value	Max ident	Links
AY239992.1	Dendrobium sanguinolentum 18S ribosomal RNA gene, partial sequence; internal transcribed spacer 1, 5.8S ribosomal RNA gene and internal transcribed spacer 2, complete sequence; and 26S ribosomal RNA gene, partial sequence	1225	1225	100%	0.0	100%	
AY239984.1	Dendrobium mutabile 18S ribosomal RNA gene, partial sequence; internal transcribed spacer 1, 5.8S ribosomal RNA gene and internal transcribed spacer 2, complete sequence; and 26S ribosomal RNA gene, partial sequence	1168	1168	100%	0.0	98%	
AY239973.1	Dendrobium inflatum 18S ribosomal RNA gene, partial sequence; internal transcribed spacer 1, 5.8S ribosomal RNA gene and internal transcribed spacer 2, complete sequence; and 26S ribosomal RNA gene, partial sequence	1003	1003	100%	0.0	94%	
AY239974.1	Dendrobium ionopus 18S ribosomal RNA gene, partial sequence; internal transcribed spacer 1, 5.8S ribosomal RNA gene and internal transcribed spacer 2, complete sequence; and 26S ribosomal RNA gene, partial sequence	852	852	100%	0.0	90%	
AY239993.1	Dendrobium secundum 18S ribosomal RNA gene, partial sequence; internal transcribed spacer 1, 5.8S ribosomal RNA gene and internal transcribed spacer 2, complete sequence; and 26S ribosomal RNA gene, partial sequence	819	819	100%	0.0	89%	
AY239952.1	Dendrobium amethystoglossum 18S ribosomal RNA gene, partial sequence; internal transcribed spacer 1, 5.8S ribosomal RNA gene and internal transcribed spacer 2, complete sequence; and 26S ribosomal RNA gene, partial sequence	811	811	100%	0.0	88%	
AY239959.1	Dendrobium cerinum 18S ribosomal RNA gene, partial sequence; internal transcribed spacer 1, 5.8S ribosomal RNA gene and internal transcribed spacer 2, complete sequence; and 26S ribosomal RNA gene, partial sequence	802	802	100%	0.0	88%	
AY239962.1	Dendrobium aff. crocatum Smedley s.n. 18S ribosomal RNA gene, partial sequence; internal transcribed spacer 1, 5.8S ribosomal RNA gene and internal transcribed spacer 2, complete sequence; and 26S ribosomal RNA gene, partial sequence	795	795	100%	0.0	88%	
AY239955.1	Dendrobium camptocentrum 18S ribosomal RNA gene, partial sequence; internal transcribed spacer 1, 5.8S ribosomal RNA gene and internal transcribed spacer 2, complete sequence; and 26S ribosomal RNA gene, partial sequence	773	773	100%	0.0	88%	
AY240001.1	Dendrobium thyrsiflorum 18S ribosomal RNA gene, partial sequence; internal transcribed spacer 1, 5.8S ribosomal RNA gene and internal transcribed spacer 2, complete sequence; and 26S ribosomal RNA gene, partial sequence	699	699	100%	0.0	85%	
HM054758.1	Dendrobium thyrsiflorum voucher SBB-0518 internal transcribed spacer 1, partial sequence; 5.8S ribosomal RNA gene and internal transcribed spacer 2, complete sequence; and 26S ribosomal RNA gene, partial sequence	697	697	98%	0.0	86%	
HM054626.1	Dendrobium densiflorum voucher SBB-0120 internal transcribed spacer 1, partial sequence; 5.8S ribosomal RNA gene and internal transcribed spacer 2, complete sequence; and 26S ribosomal RNA gene, partial sequence	684	684	97%	0.0	85%	
FJ384733.1	Dendrobium thyrsiflorum internal transcribed spacer 1, partial sequence; 5.8S ribosomal RNA gene, complete sequence; and internal transcribed spacer 2, partial sequence	664	664	95%	0.0	85%	

NCBI Blast:EU430390.1 Dendrobium schoeninum - Mozilla Firefox

file:///G:/THESIS/NCBI DOWNLOADED MATK SEQUENCES/ITS/BLAST results 93+36 sequences/NCBI BlastEU430390_1 Dendrobium schoeninum_htm.htm

Legend for links to other resources: UniGene GEO Gene S Structure Map Viewer PubChem BioAssay

Sequences producing significant alignments:

Accession	Description	Max score	Total score	Query coverage	E value	Max ident	Links
EU430390.1	Dendrobium schoeninum 18S ribosomal RNA gene, partial sequence; internal transcribed spacer 1, 5.8S ribosomal RNA gene, and internal transcribed spacer 2, complete sequence; and 26S ribosomal RNA gene, partial sequence	1223	1223	100%	0.0	100%	
AY240008.1	Dockrillia calamiformis 18S ribosomal RNA gene, partial sequence; internal transcribed spacer 1, 5.8S ribosomal RNA gene and internal transcribed spacer 2, complete sequence; and 26S ribosomal RNA gene, partial sequence	1116	1116	100%	0.0	96%	
EU430382.1	Dendrobium gracilicaule 18S ribosomal RNA gene, partial sequence; internal transcribed spacer 1, 5.8S ribosomal RNA gene, and internal transcribed spacer 2, complete sequence; and 26S ribosomal RNA gene, partial sequence	1085	1085	100%	0.0	96%	
EU430377.1	Dendrobium falcorostrum 18S ribosomal RNA gene, partial sequence; internal transcribed spacer 1, 5.8S ribosomal RNA gene, and internal transcribed spacer 2, complete sequence; and 26S ribosomal RNA gene, partial sequence	1075	1075	100%	0.0	95%	
EU430384.1	Dendrobium kingianum subsp. carnarvonense 18S ribosomal RNA gene, partial sequence; internal transcribed spacer 1, 5.8S ribosomal RNA gene, and internal transcribed spacer 2, complete sequence; and 26S ribosomal RNA gene, partial sequence	1074	1074	100%	0.0	95%	
EU430398.1	Dendrobium speciosum var. pedunculatum 18S ribosomal RNA gene, partial sequence; internal transcribed spacer 1, 5.8S ribosomal RNA gene, and internal transcribed spacer 2, complete sequence; and 26S ribosomal RNA gene, partial sequence	1068	1068	100%	0.0	95%	
EU430395.1	Dendrobium speciosum var. curvicaule 18S ribosomal RNA gene, partial sequence; internal transcribed spacer 1, 5.8S ribosomal RNA gene, and internal transcribed spacer 2, complete sequence; and 26S ribosomal RNA gene, partial sequence	1068	1068	100%	0.0	95%	
EU430378.1	Dendrobium finniganense 18S ribosomal RNA gene, partial sequence; internal transcribed spacer 1, 5.8S ribosomal RNA gene, and internal transcribed spacer 2, complete sequence; and 26S ribosomal RNA gene, partial sequence	1068	1068	99%	0.0	95%	
EU430392.1	Dendrobium speciosum var. boreale 18S ribosomal RNA gene, partial sequence; internal transcribed spacer 1, 5.8S ribosomal RNA gene, and internal transcribed spacer 2, complete sequence; and 26S ribosomal RNA gene, partial sequence	1064	1064	98%	0.0	95%	
EU430380.1	Dendrobium fleckeri 18S ribosomal RNA gene, partial sequence; internal transcribed spacer 1, 5.8S ribosomal RNA gene, and internal transcribed spacer 2, complete sequence; and 26S ribosomal RNA gene, partial sequence	1064	1064	100%	0.0	95%	
AF321594.1	Dockrillia pugioniformis internal transcribed spacer 1, 5.8S ribosomal RNA gene, and internal transcribed spacer 2, complete sequence	1059	1059	100%	0.0	95%	
EU430383.1	Dendrobium jonesii var. magnificum 18S ribosomal RNA gene, partial sequence; internal transcribed spacer 1, 5.8S ribosomal RNA gene, and internal transcribed spacer 2, complete sequence; and 26S ribosomal RNA gene, partial sequence	1057	1057	100%	0.0	95%	
EU430385.1	Dendrobium kingianum var. pulcherrimum 18S ribosomal RNA gene, partial sequence; internal transcribed spacer 1, 5.8S ribosomal RNA gene, and internal transcribed spacer 2, complete sequence; and 26S ribosomal RNA gene, partial sequence	1050	1050	100%	0.0	95%	

REVIEW | BLAST results 93+36... | Document2 - Micro... | NCBI Blast:EU43039... | BMC | Additional files | 17:21

NCBI Blast:AY239993.1 Dendrobium secundum - Mozilla Firefox

file:///G:/THESIS/NCBI DOWNLOADED MATK SEQUENCES/ITS/BLAST results 93+36 sequences/NCBI BlastAY239993_1 Dendrobium secundum_htm.htm

Legend for links to other resources: UniGene GEO Gene S Structure Map Viewer PubChem BioAssay

Sequences producing significant alignments:

Accession	Description	Max score	Total score	Query coverage	E value	Max ident	Links
AY239993.1	Dendrobium secundum 18S ribosomal RNA gene, partial sequence; internal transcribed spacer 1, 5.8S ribosomal RNA gene and internal transcribed spacer 2, complete sequence; and 26S ribosomal RNA gene, partial sequence	1210	1210	100%	0.0	100%	
AY239974.1	Dendrobium ionopus 18S ribosomal RNA gene, partial sequence; internal transcribed spacer 1, 5.8S ribosomal RNA gene and internal transcribed spacer 2, complete sequence; and 26S ribosomal RNA gene, partial sequence	928	928	100%	0.0	92%	
AY239952.1	Dendrobium amethystoglossum 18S ribosomal RNA gene, partial sequence; internal transcribed spacer 1, 5.8S ribosomal RNA gene and internal transcribed spacer 2, complete sequence; and 26S ribosomal RNA gene, partial sequence	915	915	100%	0.0	91%	
AY239959.1	Dendrobium cerinum 18S ribosomal RNA gene, partial sequence; internal transcribed spacer 1, 5.8S ribosomal RNA gene and internal transcribed spacer 2, complete sequence; and 26S ribosomal RNA gene, partial sequence	894	894	100%	0.0	91%	
AY239962.1	Dendrobium aff. crocatum Smedley s.n. 18S ribosomal RNA gene, partial sequence; internal transcribed spacer 1, 5.8S ribosomal RNA gene and internal transcribed spacer 2, complete sequence; and 26S ribosomal RNA gene, partial sequence	832	832	100%	0.0	89%	
AY239955.1	Dendrobium camptocentrum 18S ribosomal RNA gene, partial sequence; internal transcribed spacer 1, 5.8S ribosomal RNA gene and internal transcribed spacer 2, complete sequence; and 26S ribosomal RNA gene, partial sequence	832	832	100%	0.0	89%	
AY239973.1	Dendrobium inflatum 18S ribosomal RNA gene, partial sequence; internal transcribed spacer 1, 5.8S ribosomal RNA gene and internal transcribed spacer 2, complete sequence; and 26S ribosomal RNA gene, partial sequence	824	824	100%	0.0	89%	
AY239992.1	Dendrobium sanguinolentum 18S ribosomal RNA gene, partial sequence; internal transcribed spacer 1, 5.8S ribosomal RNA gene and internal transcribed spacer 2, complete sequence; and 26S ribosomal RNA gene, partial sequence	819	819	100%	0.0	89%	
AY239984.1	Dendrobium mutabile 18S ribosomal RNA gene, partial sequence; internal transcribed spacer 1, 5.8S ribosomal RNA gene and internal transcribed spacer 2, complete sequence; and 26S ribosomal RNA gene, partial sequence	817	817	100%	0.0	89%	
AY240001.1	Dendrobium thyrsiflorum 18S ribosomal RNA gene, partial sequence; internal transcribed spacer 1, 5.8S ribosomal RNA gene and internal transcribed spacer 2, complete sequence; and 26S ribosomal RNA gene, partial sequence	730	730	100%	0.0	86%	
AY239967.1	Dendrobium formosum 18S ribosomal RNA gene, partial sequence; internal transcribed spacer 1, 5.8S ribosomal RNA gene and internal transcribed spacer 2, complete sequence; and 26S ribosomal RNA gene, partial sequence	726	726	100%	0.0	86%	
HM054758.1	Dendrobium thyrsiflorum voucher SBB-0518 internal transcribed spacer 1, partial sequence; 5.8S ribosomal RNA gene and internal transcribed spacer 2, complete sequence; and 26S ribosomal RNA gene, partial sequence	717	717	98%	0.0	86%	
HM054760.1	Dendrobium thyrsiflorum voucher SBB-0520 internal transcribed spacer 1, partial sequence; 5.8S ribosomal RNA gene and internal transcribed spacer 2, complete sequence; and 26S ribosomal RNA gene, partial sequence	715	715	95%	0.0	87%	

REVIEW | BLAST results 93+36... | Document2 - Micro... | NCBI Blast:AY23999... | BMC | Additional files | 17:21

NCBI Blast:EU477509.1 Dendrobium senile - Mozilla Firefox

file:///G:/THESIS/NCBI DOWNLOADED MATK SEQUENCES/ITS/BLAST results 93+36 sequences/NCBI BlastEU477509_1 Dendrobium senile_htm.htm

Legend for links to other resources: UniGene GEO Gene S Structure Map Viewer PubChem BioAssay

Sequences producing significant alignments:

Accession	Description	Max score	Total score	Query coverage	E value	Max ident	Links
EU477509.1	Dendrobium senile internal transcribed spacer 1, partial sequence; 5.8S ribosomal RNA gene and internal transcribed spacer 2, complete sequence; and 28S ribosomal RNA gene, partial sequence	1190	1190	100%	0.0	100%	
GU339106.1	Dendrobium christyanum internal transcribed spacer 1, partial sequence; 5.8S ribosomal RNA gene, complete sequence; and internal transcribed spacer 2, partial sequence	776	776	100%	0.0	88%	
EF629325.1	Dendrobium christyanum internal transcribed spacer 1, partial sequence; 5.8S ribosomal RNA gene, complete sequence; and internal transcribed spacer 2, partial sequence	774	774	100%	0.0	88%	
GU339105.1	Dendrobium gibsonii internal transcribed spacer 1, partial sequence; 5.8S ribosomal RNA gene, complete sequence; and internal transcribed spacer 2, partial sequence	750	750	100%	0.0	87%	
EU592015.1	Dendrobium bellatulum internal transcribed spacer 1, partial sequence; 5.8S ribosomal RNA gene, complete sequence; and internal transcribed spacer 2, partial sequence	749	749	100%	0.0	87%	
AF362041.1	Dendrobium fimbriatum isolate Yunnan internal transcribed spacer 1, partial sequence; 5.8S ribosomal RNA gene, complete sequence; and internal transcribed spacer 2, partial sequence	749	749	100%	0.0	87%	
AY239967.1	Dendrobium formosum 18S ribosomal RNA gene, partial sequence; internal transcribed spacer 1, 5.8S ribosomal RNA gene and internal transcribed spacer 2, complete sequence; and 26S ribosomal RNA gene, partial sequence	739	739	100%	0.0	87%	
FJ428220.1	Dendrobium williamsonii internal transcribed spacer 1, partial sequence; 5.8S ribosomal RNA gene, complete sequence; and internal transcribed spacer 2, partial sequence	737	737	100%	0.0	87%	
DQ058793.1	Dendrobium trigonopus voucher CG-NB-0401 internal transcribed spacer 1, 5.8S ribosomal RNA gene, and internal transcribed spacer 2, complete sequence	736	736	100%	0.0	87%	
DQ058794.1	Dendrobium trigonopus voucher CG-031010 internal transcribed spacer 1, 5.8S ribosomal RNA gene, and internal transcribed spacer 2, complete sequence >gb|DQ058795.1| Dendrobium trigonopus voucher CG-ML-0401 internal transcribed spacer 1, 5.8S ribosomal RNA gene, and internal transcribed spacer 2, complete sequence >gb|FJ384741.1| Dendrobium trigonopus internal transcribed spacer 1, partial sequence; 5.8S ribosomal RNA gene, complete sequence; and internal transcribed spacer 2, partial sequence	736	736	100%	0.0	87%	
DQ058788.1	Dendrobium dixanthum voucher HH-JL03-1 internal transcribed spacer 1, 5.8S ribosomal RNA gene, and internal transcribed spacer 2, complete sequence	732	732	98%	0.0	87%	
GU339112.1	Dendrobium longicornu internal transcribed spacer 1, partial sequence; 5.8S ribosomal RNA gene, complete sequence; and internal transcribed spacer 2, partial sequence	726	726	100%	0.0	87%	
	Dendrobium dixanthum internal transcribed spacer 1, partial sequence; 5.8S ribosomal RNA gene, complete sequence; and internal						

REVIEW | BLAST results 93+36... | Document2 - Micro... | NCBI Blast:EU47750... | BMC | Additional files | 17:22

NCBI Blast:AY239995.1 Dendrobium sinuatum - Mozilla Firefox

file:///G:/THESIS/NCBI DOWNLOADED MATK SEQUENCES/ITS/BLAST results 93+36 sequences/NCBI BlastAY239995_1 Dendrobium sinuatum_htm.htm

Legend for links to other resources: UniGene GEO Gene S Structure Map Viewer PubChem BioAssay

Sequences producing significant alignments:

Accession	Description	Max score	Total score	Query coverage	E value	Max ident	Links
AY239995.1	Dendrobium sinuatum 18S ribosomal RNA gene, partial sequence; internal transcribed spacer 1, 5.8S ribosomal RNA gene and internal transcribed spacer 2, complete sequence; and 26S ribosomal RNA gene, partial sequence	1269	1269	100%	0.0	100%	
AY239971.1	Dendrobium govidjoae 18S ribosomal RNA gene, partial sequence; internal transcribed spacer 1, 5.8S ribosomal RNA gene and internal transcribed spacer 2, complete sequence; and 26S ribosomal RNA gene, partial sequence	928	928	95%	0.0	92%	
AY239946.1	Bryobium pubescens 18S ribosomal RNA gene, partial sequence; internal transcribed spacer 1, 5.8S ribosomal RNA gene and internal transcribed spacer 2, complete sequence; and 26S ribosomal RNA gene, partial sequence	850	850	100%	0.0	89%	
AF521071.1	Eria ferruginea internal transcribed spacer 1, 5.8S ribosomal RNA gene, and internal transcribed spacer 2, complete sequence	809	809	92%	0.0	90%	
AF521073.1	Appendicula cornuta internal transcribed spacer 1, 5.8S ribosomal RNA gene, and internal transcribed spacer 2, complete sequence	558	558	91%	1e-155	83%	
AY240014.1	Eria aff. javanica CBG 740854 18S ribosomal RNA gene, partial sequence; internal transcribed spacer 1, 5.8S ribosomal RNA gene and internal transcribed spacer 2, complete sequence; and 26S ribosomal RNA gene, partial sequence	536	536	100%	7e-149	82%	
AF521072.1	Podochilus cultratus internal transcribed spacer 1, partial sequence; 5.8S ribosomal RNA gene, and internal transcribed spacer 2, complete sequence	512	512	86%	1e-141	82%	
EF670387.1	Collabium simplex 18S ribosomal RNA gene, partial sequence; internal transcribed spacer 1, 5.8S ribosomal RNA gene, and internal transcribed spacer 2, complete sequence; and 28S ribosomal RNA gene, partial sequence	494	494	80%	4e-136	83%	
EF079363.1	Chysis bractescens 18S ribosomal RNA gene, partial sequence; internal transcribed spacer 1, 5.8S ribosomal RNA gene, and internal transcribed spacer 2, complete sequence; and 26S ribosomal RNA gene, partial sequence	484	484	83%	2e-133	82%	
AF260150.1	Chysis bractescens internal transcribed spacer 1, partial sequence; 5.8S ribosomal RNA gene, complete sequence; and internal transcribed spacer 2, partial sequence	484	484	77%	2e-133	83%	
AF521052.1	Collabium sp. Chase O-821 internal transcribed spacer 1, 5.8S ribosomal RNA gene, and internal transcribed spacer 2, complete sequence	483	483	73%	9e-133	84%	
AF284727.1	Eulophia graminea internal transcribed spacer 1, 5.8S ribosomal RNA gene, and internal transcribed spacer 2, complete sequence	481	481	74%	3e-132	84%	
AY240011.1	Epigeneium cymbidioides 18S ribosomal RNA gene, partial sequence; internal transcribed spacer 1, 5.8S ribosomal RNA gene and internal transcribed spacer 2, complete sequence; and 26S ribosomal RNA gene, partial sequence	466	466	79%	9e-128	82%	
EU100765.1	Frankliniella intonsa isolate FI-1 18S ribosomal RNA gene, partial sequence; internal transcribed spacer 1, 5.8S ribosomal RNA gene, and internal transcribed spacer 2, complete sequence; and 28S ribosomal RNA gene, partial sequence	464	464	80%	3e-127	82%	
AF521069.1	Ridleyella paniculata internal transcribed spacer 1, 5.8S ribosomal RNA gene, and internal transcribed spacer 2, complete sequence	464	464	63%	3e-127	85%	
AB222033.1	Phaius graeffei genes for ITS1, 5.8S rRNA, ITS2, complete sequence	462	462	74%	1e-126	83%	

REVIEW | BLAST results 93+36... | Document2 - Micro... | NCBI Blast:AY23999... | BMC | Additional files | 17:22

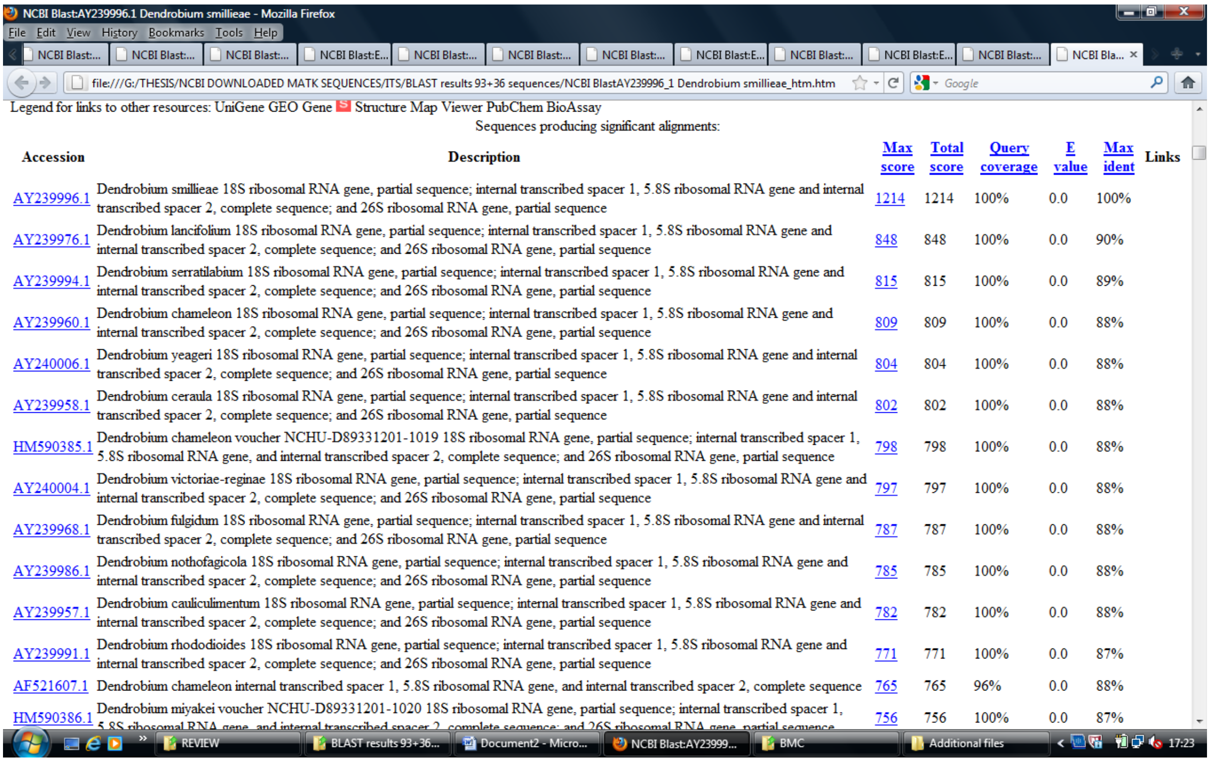

Accession	Description	Max score	Total score	Query coverage	E value	Max ident	Links
AY239996.1	Dendrobium smillieae 18S ribosomal RNA gene, partial sequence; internal transcribed spacer 1, 5.8S ribosomal RNA gene and internal transcribed spacer 2, complete sequence; and 26S ribosomal RNA gene, partial sequence	1214	1214	100%	0.0	100%	
AY239976.1	Dendrobium lancifolium 18S ribosomal RNA gene, partial sequence; internal transcribed spacer 1, 5.8S ribosomal RNA gene and internal transcribed spacer 2, complete sequence; and 26S ribosomal RNA gene, partial sequence	848	848	100%	0.0	90%	
AY239994.1	Dendrobium serratilabium 18S ribosomal RNA gene, partial sequence; internal transcribed spacer 1, 5.8S ribosomal RNA gene and internal transcribed spacer 2, complete sequence; and 26S ribosomal RNA gene, partial sequence	815	815	100%	0.0	89%	
AY239960.1	Dendrobium chameleon 18S ribosomal RNA gene, partial sequence; internal transcribed spacer 1, 5.8S ribosomal RNA gene and internal transcribed spacer 2, complete sequence; and 26S ribosomal RNA gene, partial sequence	809	809	100%	0.0	88%	
AY240006.1	Dendrobium yeageri 18S ribosomal RNA gene, partial sequence; internal transcribed spacer 1, 5.8S ribosomal RNA gene and internal transcribed spacer 2, complete sequence; and 26S ribosomal RNA gene, partial sequence	804	804	100%	0.0	88%	
AY239958.1	Dendrobium ceraula 18S ribosomal RNA gene, partial sequence; internal transcribed spacer 1, 5.8S ribosomal RNA gene and internal transcribed spacer 2, complete sequence; and 26S ribosomal RNA gene, partial sequence	802	802	100%	0.0	88%	
HM590385.1	Dendrobium chameleon voucher NCHU-D89331201-1019 18S ribosomal RNA gene, partial sequence; internal transcribed spacer 1, 5.8S ribosomal RNA gene, and internal transcribed spacer 2, complete sequence; and 26S ribosomal RNA gene, partial sequence	798	798	100%	0.0	88%	
AY240004.1	Dendrobium victoriae-reginae 18S ribosomal RNA gene, partial sequence; internal transcribed spacer 1, 5.8S ribosomal RNA gene and internal transcribed spacer 2, complete sequence; and 26S ribosomal RNA gene, partial sequence	797	797	100%	0.0	88%	
AY239968.1	Dendrobium fulgidum 18S ribosomal RNA gene, partial sequence; internal transcribed spacer 1, 5.8S ribosomal RNA gene and internal transcribed spacer 2, complete sequence; and 26S ribosomal RNA gene, partial sequence	787	787	100%	0.0	88%	
AY239986.1	Dendrobium nothofagicola 18S ribosomal RNA gene, partial sequence; internal transcribed spacer 1, 5.8S ribosomal RNA gene and internal transcribed spacer 2, complete sequence; and 26S ribosomal RNA gene, partial sequence	785	785	100%	0.0	88%	
AY239957.1	Dendrobium cauliculimentum 18S ribosomal RNA gene, partial sequence; internal transcribed spacer 1, 5.8S ribosomal RNA gene and internal transcribed spacer 2, complete sequence; and 26S ribosomal RNA gene, partial sequence	782	782	100%	0.0	88%	
AY239991.1	Dendrobium rhododioides 18S ribosomal RNA gene, partial sequence; internal transcribed spacer 1, 5.8S ribosomal RNA gene and internal transcribed spacer 2, complete sequence; and 26S ribosomal RNA gene, partial sequence	771	771	100%	0.0	87%	
AF521607.1	Dendrobium chameleon internal transcribed spacer 1, 5.8S ribosomal RNA gene, and internal transcribed spacer 2, complete sequence	765	765	96%	0.0	88%	
HM590386.1	Dendrobium miyakei voucher NCHU-D89331201-1020 18S ribosomal RNA gene, partial sequence; internal transcribed spacer 1, 5.8S ribosomal RNA gene, and internal transcribed spacer 2, complete sequence; and 26S ribosomal RNA gene, partial sequence	756	756	100%	0.0	87%	

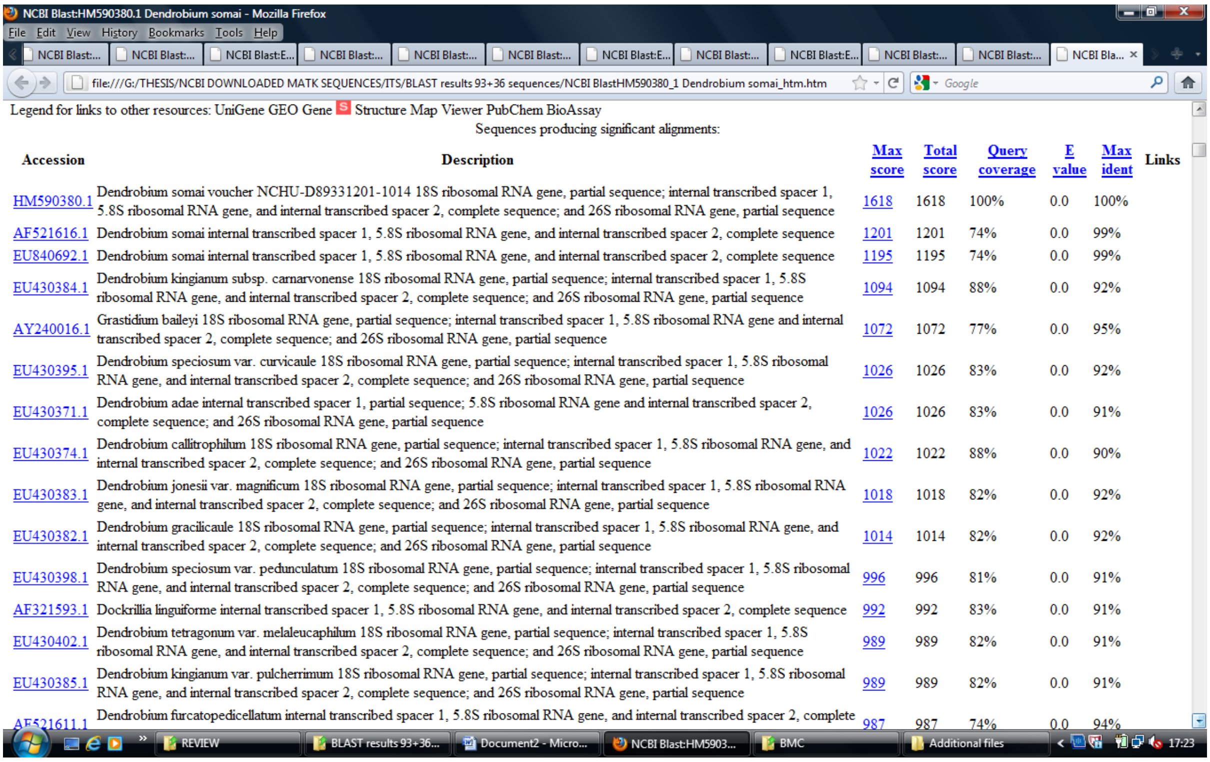

Accession	Description	Max score	Total score	Query coverage	E value	Max ident	Links
HM590380.1	Dendrobium somai voucher NCHU-D89331201-1014 18S ribosomal RNA gene, partial sequence; internal transcribed spacer 1, 5.8S ribosomal RNA gene, and internal transcribed spacer 2, complete sequence; and 26S ribosomal RNA gene, partial sequence	1618	1618	100%	0.0	100%	
AF521616.1	Dendrobium somai internal transcribed spacer 1, 5.8S ribosomal RNA gene, and internal transcribed spacer 2, complete sequence	1201	1201	74%	0.0	99%	
EU840692.1	Dendrobium somai internal transcribed spacer 1, 5.8S ribosomal RNA gene, and internal transcribed spacer 2, complete sequence	1195	1195	74%	0.0	99%	
EU430384.1	Dendrobium kingianum subsp. carnarvonense 18S ribosomal RNA gene, partial sequence; internal transcribed spacer 1, 5.8S ribosomal RNA gene, and internal transcribed spacer 2, complete sequence; and 26S ribosomal RNA gene, partial sequence	1094	1094	88%	0.0	92%	
AY240016.1	Grastidium baileyi 18S ribosomal RNA gene, partial sequence; internal transcribed spacer 1, 5.8S ribosomal RNA gene and internal transcribed spacer 2, complete sequence; and 26S ribosomal RNA gene, partial sequence	1072	1072	77%	0.0	95%	
EU430395.1	Dendrobium speciosum var. curvicaule 18S ribosomal RNA gene, partial sequence; internal transcribed spacer 1, 5.8S ribosomal RNA gene, and internal transcribed spacer 2, complete sequence; and 26S ribosomal RNA gene, partial sequence	1026	1026	83%	0.0	92%	
EU430371.1	Dendrobium adae internal transcribed spacer 1, partial sequence; 5.8S ribosomal RNA gene and internal transcribed spacer 2, complete sequence; and 26S ribosomal RNA gene, partial sequence	1026	1026	83%	0.0	91%	
EU430374.1	Dendrobium callitrophilum 18S ribosomal RNA gene, partial sequence; internal transcribed spacer 1, 5.8S ribosomal RNA gene, and internal transcribed spacer 2, complete sequence; and 26S ribosomal RNA gene, partial sequence	1022	1022	88%	0.0	90%	
EU430383.1	Dendrobium jonesii var. magnificum 18S ribosomal RNA gene, partial sequence; internal transcribed spacer 1, 5.8S ribosomal RNA gene, and internal transcribed spacer 2, complete sequence; and 26S ribosomal RNA gene, partial sequence	1018	1018	82%	0.0	92%	
EU430382.1	Dendrobium gracilicaule 18S ribosomal RNA gene, partial sequence; internal transcribed spacer 1, 5.8S ribosomal RNA gene, and internal transcribed spacer 2, complete sequence; and 26S ribosomal RNA gene, partial sequence	1014	1014	82%	0.0	92%	
EU430398.1	Dendrobium speciosum var. pedunculatum 18S ribosomal RNA gene, partial sequence; internal transcribed spacer 1, 5.8S ribosomal RNA gene, and internal transcribed spacer 2, complete sequence; and 26S ribosomal RNA gene, partial sequence	996	996	81%	0.0	91%	
AF321593.1	Dockrillia linguiforme internal transcribed spacer 1, 5.8S ribosomal RNA gene, and internal transcribed spacer 2, complete sequence	992	992	83%	0.0	91%	
EU430402.1	Dendrobium tetragonum var. melaleucaphilum 18S ribosomal RNA gene, partial sequence; internal transcribed spacer 1, 5.8S ribosomal RNA gene, and internal transcribed spacer 2, complete sequence; and 26S ribosomal RNA gene, partial sequence	989	989	82%	0.0	91%	
EU430385.1	Dendrobium kingianum var. pulcherrimum 18S ribosomal RNA gene, partial sequence; internal transcribed spacer 1, 5.8S ribosomal RNA gene, and internal transcribed spacer 2, complete sequence; and 26S ribosomal RNA gene, partial sequence	989	989	82%	0.0	91%	
AF521611.1	Dendrobium furcatopedicellatum internal transcribed spacer 1, 5.8S ribosomal RNA gene, and internal transcribed spacer 2, complete	987	987	74%	0.0	94%	

NCBI Blast:AY239998.1 Dendrobium speciosum - Mozilla Firefox

file:///G:/THESIS/NCBI DOWNLOADED MATK SEQUENCES/ITS/BLAST results 93+36 sequences/NCBI BlastAY239998_1 Dendrobium speciosum_htm.htm

Legend for links to other resources: UniGene GEO Gene Structure Map Viewer PubChem BioAssay

Sequences producing significant alignments:

Accession	Description	Max score	Total score	Query coverage	E value	Max ident	Links
AY239998.1	Dendrobium speciosum 18S ribosomal RNA gene, partial sequence; internal transcribed spacer 1, 5.8S ribosomal RNA gene and internal transcribed spacer 2, complete sequence; and 26S ribosomal RNA gene, partial sequence	1216	1216	100%	0.0	100%	
EU430399.1	Dendrobium speciosum var. speciosum 18S ribosomal RNA gene, partial sequence; internal transcribed spacer 1, 5.8S ribosomal RNA gene, and internal transcribed spacer 2, complete sequence; and 26S ribosomal RNA gene, partial sequence	1205	1205	100%	0.0	99%	
EU430397.1	Dendrobium speciosum var. hillii 18S ribosomal RNA gene, partial sequence; internal transcribed spacer 1, 5.8S ribosomal RNA gene, and internal transcribed spacer 2, complete sequence; and 26S ribosomal RNA gene, partial sequence	1205	1205	100%	0.0	99%	
EU430394.1	Dendrobium speciosum var. carnarvonense 18S ribosomal RNA gene, partial sequence; internal transcribed spacer 1, 5.8S ribosomal RNA gene, and internal transcribed spacer 2, complete sequence; and 26S ribosomal RNA gene, partial sequence	1205	1205	100%	0.0	99%	
EU430391.1	Dendrobium speciosum var. blackdownense 18S ribosomal RNA gene, partial sequence; internal transcribed spacer 1, 5.8S ribosomal RNA gene, and internal transcribed spacer 2, complete sequence; and 26S ribosomal RNA gene, partial sequence	1205	1205	100%	0.0	99%	
EU430396.1	Dendrobium speciosum var. grandiflorum 18S ribosomal RNA gene, partial sequence; internal transcribed spacer 1, 5.8S ribosomal RNA gene, and internal transcribed spacer 2, complete sequence; and 26S ribosomal RNA gene, partial sequence	1201	1201	100%	0.0	99%	
EU430393.1	Dendrobium speciosum var. capricornicum 18S ribosomal RNA gene, partial sequence; internal transcribed spacer 1, 5.8S ribosomal RNA gene, and internal transcribed spacer 2, complete sequence; and 26S ribosomal RNA gene, partial sequence	1201	1201	100%	0.0	99%	
EU430395.1	Dendrobium speciosum var. curvicaule 18S ribosomal RNA gene, partial sequence; internal transcribed spacer 1, 5.8S ribosomal RNA gene, and internal transcribed spacer 2, complete sequence; and 26S ribosomal RNA gene, partial sequence	1114	1114	100%	0.0	97%	
EU430382.1	Dendrobium gracilicaule 18S ribosomal RNA gene, partial sequence; internal transcribed spacer 1, 5.8S ribosomal RNA gene, and internal transcribed spacer 2, complete sequence; and 26S ribosomal RNA gene, partial sequence	1079	1079	100%	0.0	96%	
EU430377.1	Dendrobium falcorostrum 18S ribosomal RNA gene, partial sequence; internal transcribed spacer 1, 5.8S ribosomal RNA gene, and internal transcribed spacer 2, complete sequence; and 26S ribosomal RNA gene, partial sequence	1066	1066	99%	0.0	95%	
EU430398.1	Dendrobium speciosum var. pedunculatum 18S ribosomal RNA gene, partial sequence; internal transcribed spacer 1, 5.8S ribosomal RNA gene, and internal transcribed spacer 2, complete sequence; and 26S ribosomal RNA gene, partial sequence	1062	1062	100%	0.0	95%	
EU430392.1	Dendrobium speciosum var. boreale 18S ribosomal RNA gene, partial sequence; internal transcribed spacer 1, 5.8S ribosomal RNA gene, and internal transcribed spacer 2, complete sequence; and 26S ribosomal RNA gene, partial sequence	1055	1055	98%	0.0	95%	
EU430384.1	Dendrobium kingianum subsp. carnarvonense 18S ribosomal RNA gene, partial sequence; internal transcribed spacer 1, 5.8S ribosomal RNA gene, and internal transcribed spacer 2, complete sequence; and 26S ribosomal RNA gene, partial sequence	1051	1051	100%	0.0	95%	

Dendrobium jonesii var. magnificum 18S ribosomal RNA gene, partial sequence; internal transcribed spacer 1, 5.8S ribosomal RNA

NCBI Blast:AY239999.1 Dendrobium stuartii - Mozilla Firefox

file:///G:/THESIS/NCBI DOWNLOADED MATK SEQUENCES/ITS/BLAST results 93+36 sequences/NCBI BlastAY239999_1 Dendrobium stuartii_htm.htm

Legend for links to other resources: UniGene GEO Gene Structure Map Viewer PubChem BioAssay

Sequences producing significant alignments:

Accession	Description	Max score	Total score	Query coverage	E value	Max ident	Links
AY239999.1	Dendrobium stuartii 18S ribosomal RNA gene, partial sequence; internal transcribed spacer 1, 5.8S ribosomal RNA gene and internal transcribed spacer 2, complete sequence; and 26S ribosomal RNA gene, partial sequence	1225	1225	100%	0.0	100%	
HM054696.1	Dendrobium macrostachyum voucher SBB-0287 18S ribosomal RNA gene, partial sequence; internal transcribed spacer 1, 5.8S ribosomal RNA gene, and internal transcribed spacer 2, complete sequence; and 26S ribosomal RNA gene, partial sequence	1219	1219	100%	0.0	99%	
HM054678.1	Dendrobium macrostachyum voucher SBB-0127 18S ribosomal RNA gene, partial sequence; internal transcribed spacer 1, 5.8S ribosomal RNA gene, and internal transcribed spacer 2, complete sequence; and 26S ribosomal RNA gene, partial sequence	1219	1219	100%	0.0	99%	
HM054695.1	Dendrobium macrostachyum voucher SBB-0286 internal transcribed spacer 1, partial sequence; 5.8S ribosomal RNA gene and internal transcribed spacer 2, complete sequence; and 26S ribosomal RNA gene, partial sequence	1205	1205	98%	0.0	99%	
HM054681.1	Dendrobium macrostachyum voucher SBB-0130 internal transcribed spacer 1, partial sequence; 5.8S ribosomal RNA gene and internal transcribed spacer 2, complete sequence; and 26S ribosomal RNA gene, partial sequence	1184	1184	97%	0.0	99%	
HM054692.1	Dendrobium macrostachyum voucher SBB-0022 internal transcribed spacer 1, partial sequence; 5.8S ribosomal RNA gene and internal transcribed spacer 2, complete sequence; and 26S ribosomal RNA gene, partial sequence	1182	1182	96%	0.0	99%	
HM054694.1	Dendrobium macrostachyum voucher SBB-0024 internal transcribed spacer 1, partial sequence; 5.8S ribosomal RNA gene and internal transcribed spacer 2, complete sequence; and 26S ribosomal RNA gene, partial sequence	1181	1181	96%	0.0	99%	
HM054693.1	Dendrobium macrostachyum voucher SBB-0023 internal transcribed spacer 1, partial sequence; 5.8S ribosomal RNA gene and internal transcribed spacer 2, complete sequence; and 26S ribosomal RNA gene, partial sequence	1181	1181	96%	0.0	99%	
HM054689.1	Dendrobium macrostachyum voucher SBB-0019 internal transcribed spacer 1, partial sequence; 5.8S ribosomal RNA gene and internal transcribed spacer 2, complete sequence; and 26S ribosomal RNA gene, partial sequence	1181	1181	96%	0.0	99%	
HM054682.1	Dendrobium macrostachyum voucher SBB-0131 internal transcribed spacer 1, partial sequence; 5.8S ribosomal RNA gene and internal transcribed spacer 2, complete sequence; and 26S ribosomal RNA gene, partial sequence	1181	1181	96%	0.0	99%	
HM054698.1	Dendrobium macrostachyum voucher SBB-0008 internal transcribed spacer 1, partial sequence; 5.8S ribosomal RNA gene and internal transcribed spacer 2, complete sequence; and 26S ribosomal RNA gene, partial sequence	1179	1179	97%	0.0	99%	
HM054680.1	Dendrobium macrostachyum voucher SBB-0012 internal transcribed spacer 1, partial sequence; 5.8S ribosomal RNA gene and internal transcribed spacer 2, complete sequence; and 26S ribosomal RNA gene, partial sequence	1177	1177	96%	0.0	99%	
HM054699.1	Dendrobium macrostachyum voucher SBB-0009 internal transcribed spacer 1, partial sequence; 5.8S ribosomal RNA gene and internal transcribed spacer 2, complete sequence; and 26S ribosomal RNA gene, partial sequence	1175	1175	96%	0.0	99%	

Dendrobium macrostachyum voucher SBB-0007 internal transcribed spacer 1, partial sequence; 5.8S ribosomal RNA gene and internal

NCBI Blast:GU339104.1 Dendrobium stuposum - Mozilla Firefox

file:///G:/THESIS/NCBI DOWNLOADED MATK SEQUENCES/ITS/BLAST results 93+36 sequences/NCBI BlastGU339104_1 Dendrobium stuposum_htm.htm

Legend for links to other resources: UniGene GEO Gene Structure Map Viewer PubChem BioAssay

Sequences producing significant alignments:

Accession	Description	Max score	Total score	Query coverage	E value	Max ident	Links
GU339104.1	Dendrobium stuposum internal transcribed spacer 1, partial sequence; 5.8S ribosomal RNA gene, complete sequence; and internal transcribed spacer 2, partial sequence	1182	1182	100%	0.0	100%	
AY842036.1	Dendrobium fimbriatum internal transcribed spacer 1, partial sequence; 5.8S ribosomal RNA gene, complete sequence; and internal transcribed spacer 2, partial sequence	972	972	100%	0.0	94%	
HM054581.1	Dendrobium bicameratum voucher SBB-0213 internal transcribed spacer 1, partial sequence; 5.8S ribosomal RNA gene and internal transcribed spacer 2, complete sequence; and 26S ribosomal RNA gene, partial sequence	968	968	99%	0.0	94%	
HM054590.1	Dendrobium bicameratum voucher SBB-0239 internal transcribed spacer 1, partial sequence; 5.8S ribosomal RNA gene and internal transcribed spacer 2, complete sequence; and 26S ribosomal RNA gene, partial sequence	965	965	99%	0.0	93%	
HM054589.1	Dendrobium bicameratum voucher SBB-0228 internal transcribed spacer 1, partial sequence; 5.8S ribosomal RNA gene and internal transcribed spacer 2, complete sequence; and 26S ribosomal RNA gene, partial sequence	965	965	99%	0.0	93%	
HM054584.1	Dendrobium bicameratum voucher SBB-0217 internal transcribed spacer 1, partial sequence; 5.8S ribosomal RNA gene and internal transcribed spacer 2, complete sequence; and 26S ribosomal RNA gene, partial sequence	963	963	99%	0.0	93%	
HM054586.1	Dendrobium bicameratum voucher SBB-0222 internal transcribed spacer 1, partial sequence; 5.8S ribosomal RNA gene and internal transcribed spacer 2, complete sequence; and 26S ribosomal RNA gene, partial sequence	955	955	98%	0.0	94%	
HM054587.1	Dendrobium bicameratum voucher SBB-0223 internal transcribed spacer 1, partial sequence; 5.8S ribosomal RNA gene and internal transcribed spacer 2, complete sequence; and 26S ribosomal RNA gene, partial sequence	955	955	98%	0.0	94%	
HM054591.1	Dendrobium bicameratum voucher SBB-0028 internal transcribed spacer 1, partial sequence; 5.8S ribosomal RNA gene and internal transcribed spacer 2, complete sequence; and 26S ribosomal RNA gene, partial sequence	953	953	98%	0.0	94%	
HM054585.1	Dendrobium bicameratum voucher SBB-0221 internal transcribed spacer 1, partial sequence; 5.8S ribosomal RNA gene and internal transcribed spacer 2, complete sequence; and 26S ribosomal RNA gene, partial sequence	950	950	98%	0.0	93%	
HM054583.1	Dendrobium bicameratum voucher SBB-0216 internal transcribed spacer 1, partial sequence; 5.8S ribosomal RNA gene and internal transcribed spacer 2, complete sequence; and 26S ribosomal RNA gene, partial sequence	946	946	97%	0.0	93%	
HM054582.1	Dendrobium bicameratum voucher SBB-0215 internal transcribed spacer 1, partial sequence; 5.8S ribosomal RNA gene, complete sequence; and internal transcribed spacer 2, partial sequence	944	944	97%	0.0	93%	
HM054588.1	Dendrobium bicameratum voucher SBB-0226 internal transcribed spacer 1, partial sequence; 5.8S ribosomal RNA gene and internal transcribed spacer 2, complete sequence; and 26S ribosomal RNA gene, partial sequence	942	942	97%	0.0	94%	
[illegible]	[illegible]						

NCBI Blast:AY240000.1 Dendrobium subuliferum - Mozilla Firefox

file:///G:/THESIS/NCBI DOWNLOADED MATK SEQUENCES/ITS/BLAST results 93+36 sequences/NCBI BlastAY240000_1 Dendrobium subuliferum_htm.htm

Legend for links to other resources: UniGene GEO Gene Structure Map Viewer PubChem BioAssay

Sequences producing significant alignments:

Accession	Description	Max score	Total score	Query coverage	E value	Max ident	Links
AY240000.1	Dendrobium subuliferum 18S ribosomal RNA gene, partial sequence; internal transcribed spacer 1, 5.8S ribosomal RNA gene and internal transcribed spacer 2, complete sequence; and 26S ribosomal RNA gene, partial sequence	1229	1229	100%	0.0	100%	
AY239964.1	Dendrobium cyanocentrum 18S ribosomal RNA gene, partial sequence; internal transcribed spacer 1, 5.8S ribosomal RNA gene and internal transcribed spacer 2, complete sequence; and 26S ribosomal RNA gene, partial sequence	1155	1155	100%	0.0	98%	
AY240006.1	Dendrobium yeageri 18S ribosomal RNA gene, partial sequence; internal transcribed spacer 1, 5.8S ribosomal RNA gene and internal transcribed spacer 2, complete sequence; and 26S ribosomal RNA gene, partial sequence	776	776	99%	0.0	88%	
AY239976.1	Dendrobium lancifolium 18S ribosomal RNA gene, partial sequence; internal transcribed spacer 1, 5.8S ribosomal RNA gene and internal transcribed spacer 2, complete sequence; and 26S ribosomal RNA gene, partial sequence	776	776	99%	0.0	87%	
AY239960.1	Dendrobium chameleon 18S ribosomal RNA gene, partial sequence; internal transcribed spacer 1, 5.8S ribosomal RNA gene and internal transcribed spacer 2, complete sequence; and 26S ribosomal RNA gene, partial sequence	776	776	99%	0.0	88%	
AY239958.1	Dendrobium ceraula 18S ribosomal RNA gene, partial sequence; internal transcribed spacer 1, 5.8S ribosomal RNA gene and internal transcribed spacer 2, complete sequence; and 26S ribosomal RNA gene, partial sequence	769	769	99%	0.0	87%	
AY240004.1	Dendrobium victoriae-reginae 18S ribosomal RNA gene, partial sequence; internal transcribed spacer 1, 5.8S ribosomal RNA gene and internal transcribed spacer 2, complete sequence; and 26S ribosomal RNA gene, partial sequence	763	763	99%	0.0	87%	
AY239994.1	Dendrobium serratilabium 18S ribosomal RNA gene, partial sequence; internal transcribed spacer 1, 5.8S ribosomal RNA gene and internal transcribed spacer 2, complete sequence; and 26S ribosomal RNA gene, partial sequence	760	760	99%	0.0	87%	
HM590385.1	Dendrobium chameleon voucher NCHU-D89331201-1019 18S ribosomal RNA gene, partial sequence; internal transcribed spacer 1, 5.8S ribosomal RNA gene, and internal transcribed spacer 2, complete sequence; and 26S ribosomal RNA gene, partial sequence	749	749	99%	0.0	87%	
AY239968.1	Dendrobium fulgidum 18S ribosomal RNA gene, partial sequence; internal transcribed spacer 1, 5.8S ribosomal RNA gene and internal transcribed spacer 2, complete sequence; and 26S ribosomal RNA gene, partial sequence	743	743	99%	0.0	87%	
HM590386.1	Dendrobium miyakei voucher NCHU-D89331201-1020 18S ribosomal RNA gene, partial sequence; internal transcribed spacer 1, 5.8S ribosomal RNA gene, and internal transcribed spacer 2, complete sequence; and 26S ribosomal RNA gene, partial sequence	734	734	99%	0.0	86%	
AY239970.1	Dendrobium goldschmidtianum 18S ribosomal RNA gene, partial sequence; internal transcribed spacer 1, 5.8S ribosomal RNA gene and internal transcribed spacer 2, complete sequence; and 26S ribosomal RNA gene, partial sequence	734	734	99%	0.0	86%	
AF521607.1	Dendrobium chameleon internal transcribed spacer 1, 5.8S ribosomal RNA gene, and internal transcribed spacer 2, complete sequence	734	734	96%	0.0	87%	
AY239990.1	Dendrobium aff. rarum Clements 5613 18S ribosomal RNA gene, partial sequence; internal transcribed spacer 1, 5.8S ribosomal RNA [illegible]	726	726	99%	0.0	86%	

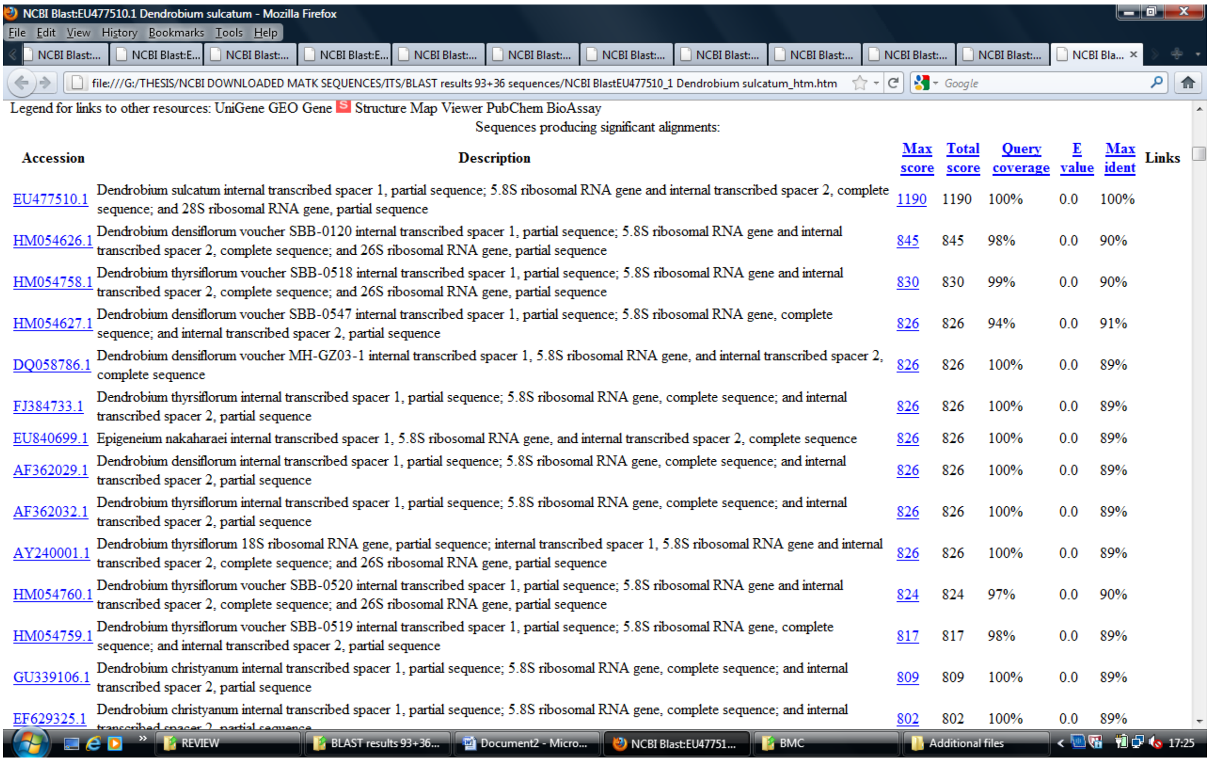

Legend for links to other resources: UniGene GEO Gene Structure Map Viewer PubChem BioAssay

Sequences producing significant alignments:

Accession	Description	Max score	Total score	Query coverage	E value	Max ident	Links
EU477510.1	Dendrobium sulcatum internal transcribed spacer 1, partial sequence; 5.8S ribosomal RNA gene and internal transcribed spacer 2, complete sequence; and 28S ribosomal RNA gene, partial sequence	1190	1190	100%	0.0	100%	
HM054626.1	Dendrobium densiflorum voucher SBB-0120 internal transcribed spacer 1, partial sequence; 5.8S ribosomal RNA gene and internal transcribed spacer 2, complete sequence; and 26S ribosomal RNA gene, partial sequence	845	845	98%	0.0	90%	
HM054758.1	Dendrobium thyrsiflorum voucher SBB-0518 internal transcribed spacer 1, partial sequence; 5.8S ribosomal RNA gene and internal transcribed spacer 2, complete sequence; and 26S ribosomal RNA gene, partial sequence	830	830	99%	0.0	90%	
HM054627.1	Dendrobium densiflorum voucher SBB-0547 internal transcribed spacer 1, partial sequence; 5.8S ribosomal RNA gene, complete sequence; and internal transcribed spacer 2, partial sequence	826	826	94%	0.0	91%	
DQ058786.1	Dendrobium densiflorum voucher MH-GZ03-1 internal transcribed spacer 1, 5.8S ribosomal RNA gene, and internal transcribed spacer 2, complete sequence	826	826	100%	0.0	89%	
FJ384733.1	Dendrobium thyrsiflorum internal transcribed spacer 1, partial sequence; 5.8S ribosomal RNA gene, complete sequence; and internal transcribed spacer 2, partial sequence	826	826	100%	0.0	89%	
EU840699.1	Epigeneium nakaharaei internal transcribed spacer 1, 5.8S ribosomal RNA gene, and internal transcribed spacer 2, complete sequence	826	826	100%	0.0	89%	
AF362029.1	Dendrobium densiflorum internal transcribed spacer 1, partial sequence; 5.8S ribosomal RNA gene, complete sequence; and internal transcribed spacer 2, partial sequence	826	826	100%	0.0	89%	
AF362032.1	Dendrobium thyrsiflorum internal transcribed spacer 1, partial sequence; 5.8S ribosomal RNA gene, complete sequence; and internal transcribed spacer 2, partial sequence	826	826	100%	0.0	89%	
AY240001.1	Dendrobium thyrsiflorum 18S ribosomal RNA gene, partial sequence; internal transcribed spacer 1, 5.8S ribosomal RNA gene and internal transcribed spacer 2, complete sequence; and 26S ribosomal RNA gene, partial sequence	826	826	100%	0.0	89%	
HM054760.1	Dendrobium thyrsiflorum voucher SBB-0520 internal transcribed spacer 1, partial sequence; 5.8S ribosomal RNA gene and internal transcribed spacer 2, complete sequence; and 26S ribosomal RNA gene, partial sequence	824	824	97%	0.0	90%	
HM054759.1	Dendrobium thyrsiflorum voucher SBB-0519 internal transcribed spacer 1, partial sequence; 5.8S ribosomal RNA gene, complete sequence; and internal transcribed spacer 2, partial sequence	817	817	98%	0.0	89%	
GU339106.1	Dendrobium christyanum internal transcribed spacer 1, partial sequence; 5.8S ribosomal RNA gene, complete sequence; and internal transcribed spacer 2, partial sequence	809	809	100%	0.0	89%	
EF629325.1	Dendrobium christyanum internal transcribed spacer 1, partial sequence; 5.8S ribosomal RNA gene, complete sequence; and internal transcribed spacer 2, partial sequence	802	802	100%	0.0	89%	

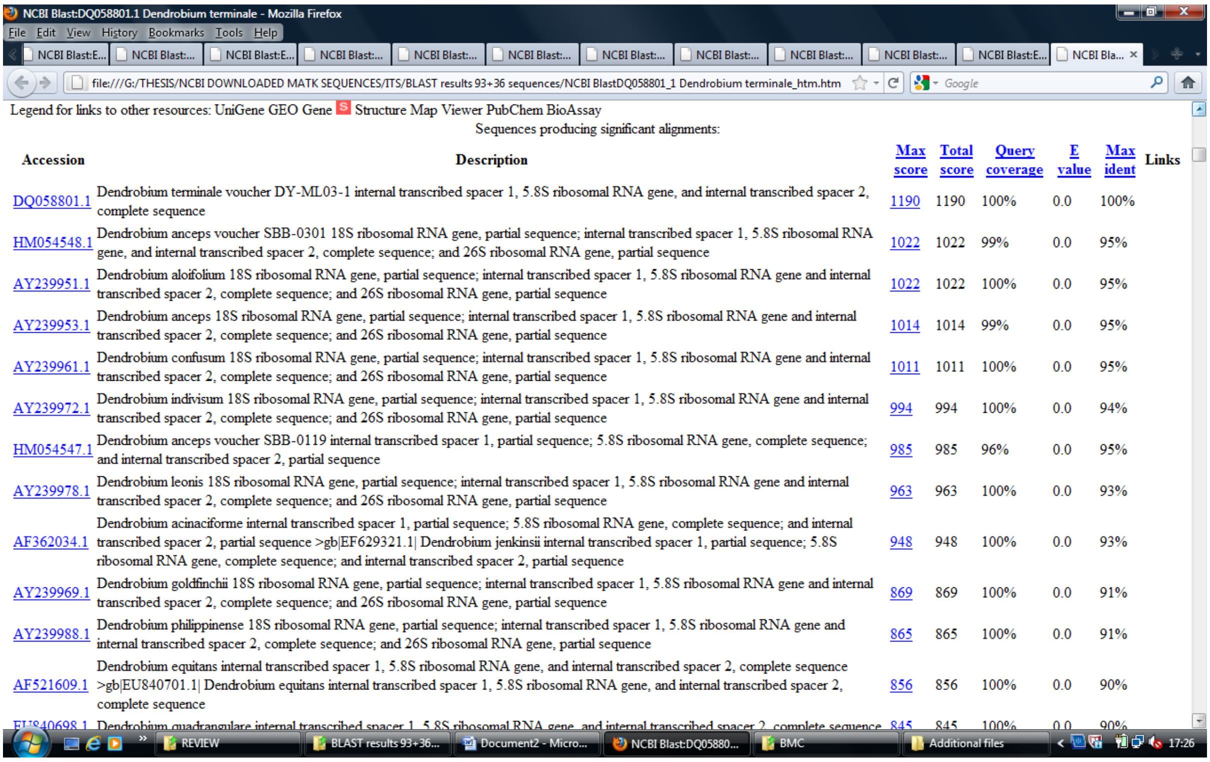

Legend for links to other resources: UniGene GEO Gene Structure Map Viewer PubChem BioAssay

Sequences producing significant alignments:

Accession	Description	Max score	Total score	Query coverage	E value	Max ident	Links
DQ058801.1	Dendrobium terminale voucher DY-ML03-1 internal transcribed spacer 1, 5.8S ribosomal RNA gene, and internal transcribed spacer 2, complete sequence	1190	1190	100%	0.0	100%	
HM054548.1	Dendrobium anceps voucher SBB-0301 18S ribosomal RNA gene, partial sequence; internal transcribed spacer 1, 5.8S ribosomal RNA gene, and internal transcribed spacer 2, complete sequence; and 26S ribosomal RNA gene, partial sequence	1022	1022	99%	0.0	95%	
AY239951.1	Dendrobium aloifolium 18S ribosomal RNA gene, partial sequence; internal transcribed spacer 1, 5.8S ribosomal RNA gene and internal transcribed spacer 2, complete sequence; and 26S ribosomal RNA gene, partial sequence	1022	1022	100%	0.0	95%	
AY239953.1	Dendrobium anceps 18S ribosomal RNA gene, partial sequence; internal transcribed spacer 1, 5.8S ribosomal RNA gene and internal transcribed spacer 2, complete sequence; and 26S ribosomal RNA gene, partial sequence	1014	1014	99%	0.0	95%	
AY239961.1	Dendrobium confusum 18S ribosomal RNA gene, partial sequence; internal transcribed spacer 1, 5.8S ribosomal RNA gene and internal transcribed spacer 2, complete sequence; and 26S ribosomal RNA gene, partial sequence	1011	1011	100%	0.0	95%	
AY239972.1	Dendrobium indivisum 18S ribosomal RNA gene, partial sequence; internal transcribed spacer 1, 5.8S ribosomal RNA gene and internal transcribed spacer 2, complete sequence; and 26S ribosomal RNA gene, partial sequence	994	994	100%	0.0	94%	
HM054547.1	Dendrobium anceps voucher SBB-0119 internal transcribed spacer 1, partial sequence; 5.8S ribosomal RNA gene, complete sequence; and internal transcribed spacer 2, partial sequence	985	985	96%	0.0	95%	
AY239978.1	Dendrobium leonis 18S ribosomal RNA gene, partial sequence; internal transcribed spacer 1, 5.8S ribosomal RNA gene and internal transcribed spacer 2, complete sequence; and 26S ribosomal RNA gene, partial sequence	963	963	100%	0.0	93%	
AF362034.1	Dendrobium acinaciforme internal transcribed spacer 1, partial sequence; 5.8S ribosomal RNA gene, complete sequence; and internal transcribed spacer 2, partial sequence >gb|EF629321.1| Dendrobium jenkinsii internal transcribed spacer 1, partial sequence; 5.8S ribosomal RNA gene, complete sequence; and internal transcribed spacer 2, partial sequence	948	948	100%	0.0	93%	
AY239969.1	Dendrobium goldfinchii 18S ribosomal RNA gene, partial sequence; internal transcribed spacer 1, 5.8S ribosomal RNA gene and internal transcribed spacer 2, complete sequence; and 26S ribosomal RNA gene, partial sequence	869	869	100%	0.0	91%	
AY239988.1	Dendrobium philippinense 18S ribosomal RNA gene, partial sequence; internal transcribed spacer 1, 5.8S ribosomal RNA gene and internal transcribed spacer 2, complete sequence; and 26S ribosomal RNA gene, partial sequence	865	865	100%	0.0	91%	
AF521609.1	Dendrobium equitans internal transcribed spacer 1, 5.8S ribosomal RNA gene, and internal transcribed spacer 2, complete sequence >gb|EU840701.1| Dendrobium equitans internal transcribed spacer 1, 5.8S ribosomal RNA gene, and internal transcribed spacer 2, complete sequence	856	856	100%	0.0	90%	
EU840698.1	Dendrobium quadrangulare internal transcribed spacer 1, 5.8S ribosomal RNA gene, and internal transcribed spacer 2, complete sequence	845	845	100%	0.0	90%	

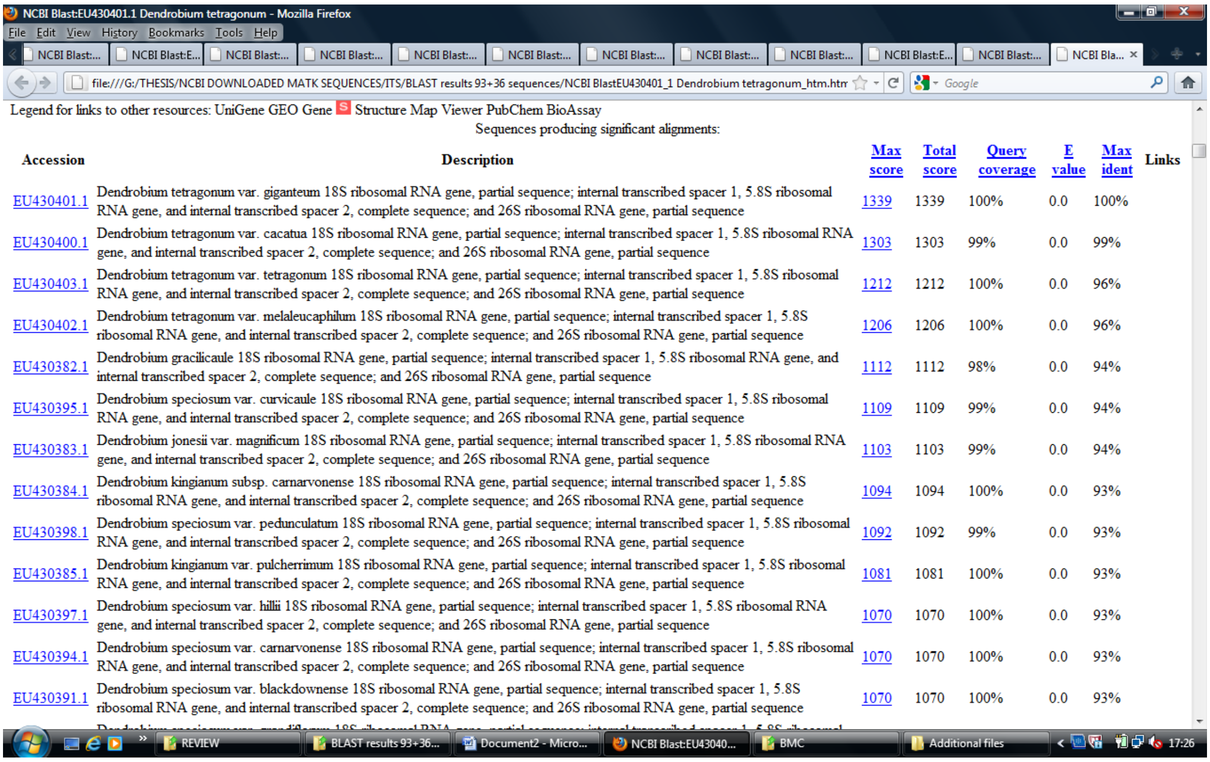

Legend for links to other resources: UniGene GEO Gene S Structure Map Viewer PubChem BioAssay

Sequences producing significant alignments:

Accession	Description	Max score	Total score	Query coverage	E value	Max ident	Links
EU430401.1	Dendrobium tetragonum var. giganteum 18S ribosomal RNA gene, partial sequence; internal transcribed spacer 1, 5.8S ribosomal RNA gene, and internal transcribed spacer 2, complete sequence; and 26S ribosomal RNA gene, partial sequence	1339	1339	100%	0.0	100%	
EU430400.1	Dendrobium tetragonum var. cacatua 18S ribosomal RNA gene, partial sequence; internal transcribed spacer 1, 5.8S ribosomal RNA gene, and internal transcribed spacer 2, complete sequence; and 26S ribosomal RNA gene, partial sequence	1303	1303	99%	0.0	99%	
EU430403.1	Dendrobium tetragonum var. tetragonum 18S ribosomal RNA gene, partial sequence; internal transcribed spacer 1, 5.8S ribosomal RNA gene, and internal transcribed spacer 2, complete sequence; and 26S ribosomal RNA gene, partial sequence	1212	1212	100%	0.0	96%	
EU430402.1	Dendrobium tetragonum var. melaleucaphilum 18S ribosomal RNA gene, partial sequence; internal transcribed spacer 1, 5.8S ribosomal RNA gene, and internal transcribed spacer 2, complete sequence; and 26S ribosomal RNA gene, partial sequence	1206	1206	100%	0.0	96%	
EU430382.1	Dendrobium gracilicaule 18S ribosomal RNA gene, partial sequence; internal transcribed spacer 1, 5.8S ribosomal RNA gene, and internal transcribed spacer 2, complete sequence; and 26S ribosomal RNA gene, partial sequence	1112	1112	98%	0.0	94%	
EU430395.1	Dendrobium speciosum var. curvicaule 18S ribosomal RNA gene, partial sequence; internal transcribed spacer 1, 5.8S ribosomal RNA gene, and internal transcribed spacer 2, complete sequence; and 26S ribosomal RNA gene, partial sequence	1109	1109	99%	0.0	94%	
EU430383.1	Dendrobium jonesii var. magnificum 18S ribosomal RNA gene, partial sequence; internal transcribed spacer 1, 5.8S ribosomal RNA gene, and internal transcribed spacer 2, complete sequence; and 26S ribosomal RNA gene, partial sequence	1103	1103	99%	0.0	94%	
EU430384.1	Dendrobium kingianum subsp. carnarvonense 18S ribosomal RNA gene, partial sequence; internal transcribed spacer 1, 5.8S ribosomal RNA gene, and internal transcribed spacer 2, complete sequence; and 26S ribosomal RNA gene, partial sequence	1094	1094	100%	0.0	93%	
EU430398.1	Dendrobium speciosum var. pedunculatum 18S ribosomal RNA gene, partial sequence; internal transcribed spacer 1, 5.8S ribosomal RNA gene, and internal transcribed spacer 2, complete sequence; and 26S ribosomal RNA gene, partial sequence	1092	1092	99%	0.0	93%	
EU430385.1	Dendrobium kingianum var. pulcherrimum 18S ribosomal RNA gene, partial sequence; internal transcribed spacer 1, 5.8S ribosomal RNA gene, and internal transcribed spacer 2, complete sequence; and 26S ribosomal RNA gene, partial sequence	1081	1081	100%	0.0	93%	
EU430397.1	Dendrobium speciosum var. hillii 18S ribosomal RNA gene, partial sequence; internal transcribed spacer 1, 5.8S ribosomal RNA gene, and internal transcribed spacer 2, complete sequence; and 26S ribosomal RNA gene, partial sequence	1070	1070	100%	0.0	93%	
EU430394.1	Dendrobium speciosum var. carnarvonense 18S ribosomal RNA gene, partial sequence; internal transcribed spacer 1, 5.8S ribosomal RNA gene, and internal transcribed spacer 2, complete sequence; and 26S ribosomal RNA gene, partial sequence	1070	1070	100%	0.0	93%	
EU430391.1	Dendrobium speciosum var. blackdownense 18S ribosomal RNA gene, partial sequence; internal transcribed spacer 1, 5.8S ribosomal RNA gene, and internal transcribed spacer 2, complete sequence; and 26S ribosomal RNA gene, partial sequence	1070	1070	100%	0.0	93%	
[illegible]	[illegible]						

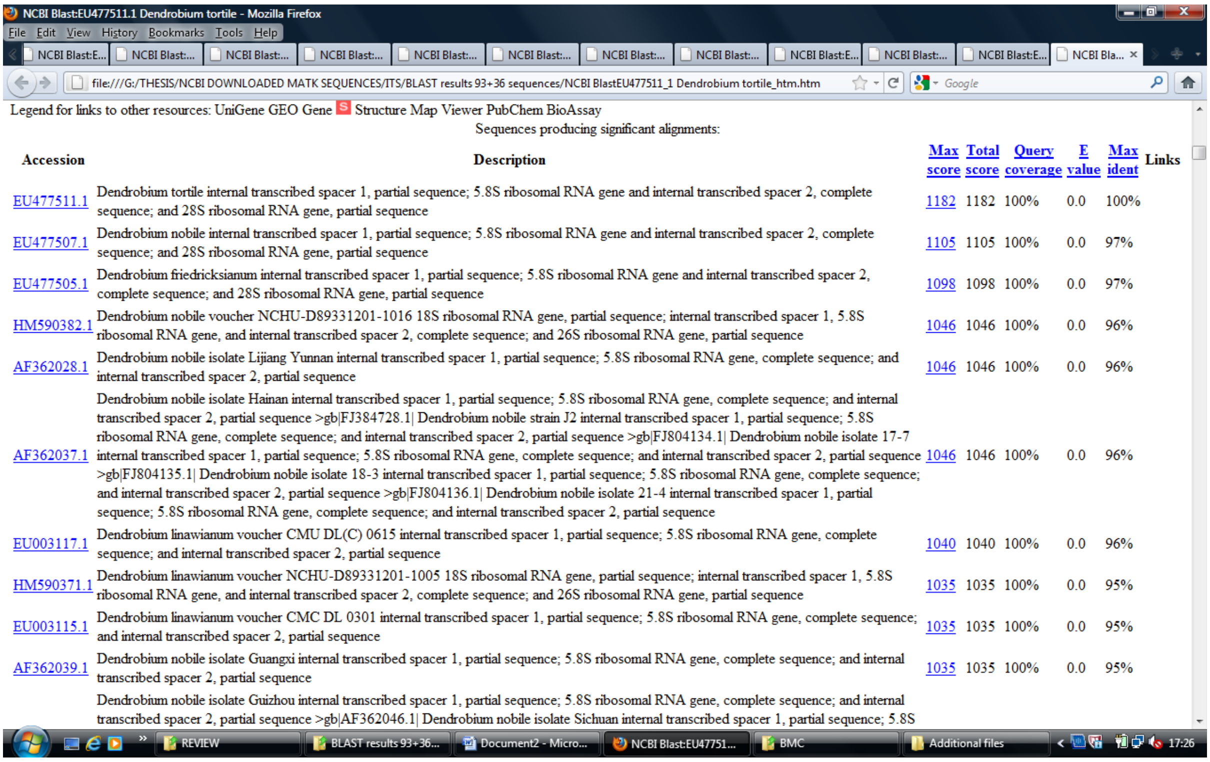

Legend for links to other resources: UniGene GEO Gene S Structure Map Viewer PubChem BioAssay

Sequences producing significant alignments:

Accession	Description	Max score	Total score	Query coverage	E value	Max ident	Links
EU477511.1	Dendrobium tortile internal transcribed spacer 1, partial sequence; 5.8S ribosomal RNA gene and internal transcribed spacer 2, complete sequence; and 28S ribosomal RNA gene, partial sequence	1182	1182	100%	0.0	100%	
EU477507.1	Dendrobium nobile internal transcribed spacer 1, partial sequence; 5.8S ribosomal RNA gene and internal transcribed spacer 2, complete sequence; and 28S ribosomal RNA gene, partial sequence	1105	1105	100%	0.0	97%	
EU477505.1	Dendrobium friedricksianum internal transcribed spacer 1, partial sequence; 5.8S ribosomal RNA gene and internal transcribed spacer 2, complete sequence; and 28S ribosomal RNA gene, partial sequence	1098	1098	100%	0.0	97%	
HM590382.1	Dendrobium nobile voucher NCHU-D89331201-1016 18S ribosomal RNA gene, partial sequence; internal transcribed spacer 1, 5.8S ribosomal RNA gene, and internal transcribed spacer 2, complete sequence; and 26S ribosomal RNA gene, partial sequence	1046	1046	100%	0.0	96%	
AF362028.1	Dendrobium nobile isolate Lijiang Yunnan internal transcribed spacer 1, partial sequence; 5.8S ribosomal RNA gene, complete sequence; and internal transcribed spacer 2, partial sequence	1046	1046	100%	0.0	96%	
AF362037.1	Dendrobium nobile isolate Hainan internal transcribed spacer 1, partial sequence; 5.8S ribosomal RNA gene, complete sequence; and internal transcribed spacer 2, partial sequence >gb|FJ384728.1| Dendrobium nobile strain J2 internal transcribed spacer 1, partial sequence; 5.8S ribosomal RNA gene, complete sequence; and internal transcribed spacer 2, partial sequence >gb|FJ804134.1| Dendrobium nobile isolate 17-7 internal transcribed spacer 1, partial sequence; 5.8S ribosomal RNA gene, complete sequence; and internal transcribed spacer 2, partial sequence >gb|FJ804135.1| Dendrobium nobile isolate 18-3 internal transcribed spacer 1, partial sequence; 5.8S ribosomal RNA gene, complete sequence; and internal transcribed spacer 2, partial sequence >gb|FJ804136.1| Dendrobium nobile isolate 21-4 internal transcribed spacer 1, partial sequence; 5.8S ribosomal RNA gene, complete sequence; and internal transcribed spacer 2, partial sequence	1046	1046	100%	0.0	96%	
EU003117.1	Dendrobium linawianum voucher CMU DL(C) 0615 internal transcribed spacer 1, partial sequence; 5.8S ribosomal RNA gene, complete sequence; and internal transcribed spacer 2, partial sequence	1040	1040	100%	0.0	96%	
HM590371.1	Dendrobium linawianum voucher NCHU-D89331201-1005 18S ribosomal RNA gene, partial sequence; internal transcribed spacer 1, 5.8S ribosomal RNA gene, and internal transcribed spacer 2, complete sequence; and 26S ribosomal RNA gene, partial sequence	1035	1035	100%	0.0	95%	
EU003115.1	Dendrobium linawianum voucher CMC DL 0301 internal transcribed spacer 1, partial sequence; 5.8S ribosomal RNA gene, complete sequence; and internal transcribed spacer 2, partial sequence	1035	1035	100%	0.0	95%	
AF362039.1	Dendrobium nobile isolate Guangxi internal transcribed spacer 1, partial sequence; 5.8S ribosomal RNA gene, complete sequence; and internal transcribed spacer 2, partial sequence	1035	1035	100%	0.0	95%	
	Dendrobium nobile isolate Guizhou internal transcribed spacer 1, partial sequence; 5.8S ribosomal RNA gene, complete sequence; and internal transcribed spacer 2, partial sequence >gb|AF362046.1| Dendrobium nobile isolate Sichuan internal transcribed spacer 1, partial sequence; 5.8S						

NCBI Blast:HM590367.1 Dendrobium tosaense - Mozilla Firefox

file:///G:/THESIS/NCBI DOWNLOADED MATK SEQUENCES/ITS/BLAST results 93+36 sequences/NCBI BlastHM590367_1 Dendrobium tosaense_htm.htm

Legend for links to other resources: UniGene GEO Gene S Structure Map Viewer PubChem BioAssay

Sequences producing significant alignments:

Accession	Description	Max score	Total score	Query coverage	E value	Max ident	Links
HM590367.1	Dendrobium tosaense voucher NCHU-D89331201-1001 18S ribosomal RNA gene, partial sequence; internal transcribed spacer 1, 5.8S ribosomal RNA gene, and internal transcribed spacer 2, complete sequence; and 26S ribosomal RNA gene, partial sequence	1570	1570	100%	0.0	100%	
HM590391.1	Dendrobium candidum voucher NCHU-D89331201-1028 18S ribosomal RNA gene, partial sequence; internal transcribed spacer 1, 5.8S ribosomal RNA gene, and internal transcribed spacer 2, complete sequence; and 26S ribosomal RNA gene, partial sequence	1555	1555	100%	0.0	99%	
HM590382.1	Dendrobium nobile voucher NCHU-D89331201-1016 18S ribosomal RNA gene, partial sequence; internal transcribed spacer 1, 5.8S ribosomal RNA gene, and internal transcribed spacer 2, complete sequence; and 26S ribosomal RNA gene, partial sequence	1315	1315	100%	0.0	94%	
HM590371.1	Dendrobium linawianum voucher NCHU-D89331201-1005 18S ribosomal RNA gene, partial sequence; internal transcribed spacer 1, 5.8S ribosomal RNA gene, and internal transcribed spacer 2, complete sequence; and 26S ribosomal RNA gene, partial sequence	1310	1310	100%	0.0	94%	
HM590381.1	Dendrobium hercoglossum voucher NCHU-D89331201-1015 18S ribosomal RNA gene, partial sequence; internal transcribed spacer 1, 5.8S ribosomal RNA gene, and internal transcribed spacer 2, complete sequence; and 26S ribosomal RNA gene, partial sequence	1293	1293	100%	0.0	94%	
HM590372.1	Dendrobium aduncum voucher NCHU-D89331201-1006 18S ribosomal RNA gene, partial sequence; internal transcribed spacer 1, 5.8S ribosomal RNA gene, and internal transcribed spacer 2, complete sequence; and 26S ribosomal RNA gene, partial sequence	1288	1288	99%	0.0	94%	
HM590373.1	Dendrobium leptocladum voucher NCHU-D89331201-1007 18S ribosomal RNA gene, partial sequence; internal transcribed spacer 1, 5.8S ribosomal RNA gene, and internal transcribed spacer 2, complete sequence; and 26S ribosomal RNA gene, partial sequence	1175	1175	100%	0.0	91%	
AF521617.1	Dendrobium tosaense internal transcribed spacer 1, 5.8S ribosomal RNA gene, and internal transcribed spacer 2, complete sequence >gb|EU003113.1| Dendrobium tosaense voucher CMC DT 0303 internal transcribed spacer 1, partial sequence; 5.8S ribosomal RNA gene, complete sequence; and internal transcribed spacer 2, partial sequence	1175	1175	74%	0.0	100%	
FJ384723.1	Dendrobium officinale strain T1 internal transcribed spacer 1, partial sequence; 5.8S ribosomal RNA gene, complete sequence; and internal transcribed spacer 2, partial sequence	1170	1170	74%	0.0	99%	
FJ384724.1	Dendrobium officinale strain T2 internal transcribed spacer 1, partial sequence; 5.8S ribosomal RNA gene, complete sequence; and internal transcribed spacer 2, partial sequence >gb|FJ530944.1| Dendrobium officinale strain T4 internal transcribed spacer 1, partial sequence; 5.8S ribosomal RNA gene, complete sequence; and internal transcribed spacer 2, partial sequence >gb|FJ530945.1| Dendrobium officinale strain T5 internal transcribed spacer 1, partial sequence; 5.8S ribosomal RNA gene, complete sequence; and internal transcribed spacer 2, partial sequence >gb|FJ530946.1| Dendrobium officinale strain T6 internal transcribed spacer 1, partial sequence; 5.8S ribosomal RNA gene, complete sequence; and internal transcribed spacer 2, partial sequence >gb|FJ530947.1| Dendrobium officinale strain T7 internal transcribed spacer 1, partial sequence; 5.8S ribosomal RNA gene, complete sequence; and internal transcribed spacer 2, partial sequence >gb|FJ588871.1| Dendrobium officinale strain T3 internal transcribed spacer 1, partial sequence; 5.8S ribosomal RNA gene, complete sequence; and internal transcribed spacer [illegible]	1170	1170	74%	0.0	99%	

NCBI Blast:FJ384741.1 Dendrobium trigonopus - Mozilla Firefox

file:///G:/THESIS/NCBI DOWNLOADED MATK SEQUENCES/ITS/BLAST results 93+36 sequences/NCBI BlastFJ384741_1 Dendrobium trigonopus_htm.htm

Legend for links to other resources: UniGene GEO Gene S Structure Map Viewer PubChem BioAssay

Sequences producing significant alignments:

Accession	Description	Max score	Total score	Query coverage	E value	Max ident	Links
DQ058793.1	Dendrobium trigonopus voucher CG-NB-0401 internal transcribed spacer 1, 5.8S ribosomal RNA gene, and internal transcribed spacer 2, complete sequence	1190	1190	100%	0.0	100%	
DQ058794.1	Dendrobium trigonopus voucher CG-031010 internal transcribed spacer 1, 5.8S ribosomal RNA gene, and internal transcribed spacer 2, complete sequence >gb|DQ058795.1| Dendrobium trigonopus voucher CG-ML-0401 internal transcribed spacer 1, 5.8S ribosomal RNA gene, and internal transcribed spacer 2, complete sequence >gb|FJ384741.1| Dendrobium trigonopus internal transcribed spacer 1, partial sequence; 5.8S ribosomal RNA gene, complete sequence; and internal transcribed spacer 2, partial sequence	1190	1190	100%	0.0	100%	
DQ058792.1	Dendrobium bellatulum voucher AI-SC02-1 internal transcribed spacer 1, 5.8S ribosomal RNA gene, and internal transcribed spacer 2, complete sequence	1166	1166	100%	0.0	99%	
GU339106.1	Dendrobium christyanum internal transcribed spacer 1, partial sequence; 5.8S ribosomal RNA gene, complete sequence; and internal transcribed spacer 2, partial sequence	815	815	100%	0.0	89%	
EF629325.1	Dendrobium christyanum internal transcribed spacer 1, partial sequence; 5.8S ribosomal RNA gene, complete sequence; and internal transcribed spacer 2, partial sequence	808	808	100%	0.0	89%	
AY239967.1	Dendrobium formosum 18S ribosomal RNA gene, partial sequence; internal transcribed spacer 1, 5.8S ribosomal RNA gene and internal transcribed spacer 2, complete sequence; and 26S ribosomal RNA gene, partial sequence	800	800	100%	0.0	89%	
AF362027.1	Dendrobium cariniferum internal transcribed spacer 1, partial sequence; 5.8S ribosomal RNA gene, complete sequence; and internal transcribed spacer 2, partial sequence	795	795	100%	0.0	89%	
AF362030.1	Dendrobium williamsonii internal transcribed spacer 1, partial sequence; 5.8S ribosomal RNA gene, complete sequence; and internal transcribed spacer 2, partial sequence	795	795	100%	0.0	89%	
EU592015.1	Dendrobium bellatulum internal transcribed spacer 1, partial sequence; 5.8S ribosomal RNA gene, complete sequence; and internal transcribed spacer 2, partial sequence	787	787	100%	0.0	88%	
FJ428220.1	Dendrobium williamsonii internal transcribed spacer 1, partial sequence; 5.8S ribosomal RNA gene, complete sequence; and internal transcribed spacer 2, partial sequence	776	776	100%	0.0	88%	
GU339112.1	Dendrobium longicornu internal transcribed spacer 1, partial sequence; 5.8S ribosomal RNA gene, complete sequence; and internal transcribed spacer 2, partial sequence	771	771	100%	0.0	88%	
HM054668.1	Dendrobium infundibulum voucher SBB-0529 internal transcribed spacer 1, partial sequence; 5.8S ribosomal RNA gene and internal transcribed spacer 2, complete sequence; and 26S ribosomal RNA gene, partial sequence	771	771	94%	0.0	89%	
	Dendrobium infundibulum voucher SBB-0530 internal transcribed spacer 1, partial sequence; 5.8S ribosomal RNA gene and internal [illegible]						

NCBI Blast:AY240002.1 Dendrobium truncatum - Mozilla Firefox

file:///G:/THESIS/NCBI DOWNLOADED MATK SEQUENCES/ITS/BLAST results 93+36 sequences/NCBI BlastAY240002_1 Dendrobium truncatum_htm.htm

Legend for links to other resources: UniGene GEO Gene Structure Map Viewer PubChem BioAssay

Sequences producing significant alignments:

Accession	Description	Max score	Total score	Query coverage	E value	Max ident	Links
AY240002.1	Dendrobium truncatum 18S ribosomal RNA gene, partial sequence; internal transcribed spacer 1, 5.8S ribosomal RNA gene and internal transcribed spacer 2, complete sequence; and 26S ribosomal RNA gene, partial sequence	1216	1216	100%	0.0	100%	
AY239951.1	Dendrobium aloifolium 18S ribosomal RNA gene, partial sequence; internal transcribed spacer 1, 5.8S ribosomal RNA gene and internal transcribed spacer 2, complete sequence; and 26S ribosomal RNA gene, partial sequence	846	846	100%	0.0	89%	
AY239972.1	Dendrobium indivisum 18S ribosomal RNA gene, partial sequence; internal transcribed spacer 1, 5.8S ribosomal RNA gene and internal transcribed spacer 2, complete sequence; and 26S ribosomal RNA gene, partial sequence	824	824	100%	0.0	89%	
AY239961.1	Dendrobium confusum 18S ribosomal RNA gene, partial sequence; internal transcribed spacer 1, 5.8S ribosomal RNA gene and internal transcribed spacer 2, complete sequence; and 26S ribosomal RNA gene, partial sequence	824	824	100%	0.0	89%	
AY239953.1	Dendrobium anceps 18S ribosomal RNA gene, partial sequence; internal transcribed spacer 1, 5.8S ribosomal RNA gene and internal transcribed spacer 2, complete sequence; and 26S ribosomal RNA gene, partial sequence	819	819	100%	0.0	89%	
AY239988.1	Dendrobium philippinense 18S ribosomal RNA gene, partial sequence; internal transcribed spacer 1, 5.8S ribosomal RNA gene and internal transcribed spacer 2, complete sequence; and 26S ribosomal RNA gene, partial sequence	817	817	100%	0.0	89%	
AY239969.1	Dendrobium goldfinchii 18S ribosomal RNA gene, partial sequence; internal transcribed spacer 1, 5.8S ribosomal RNA gene and internal transcribed spacer 2, complete sequence; and 26S ribosomal RNA gene, partial sequence	815	815	100%	0.0	89%	
AY239989.1	Dendrobium quadrangulare 18S ribosomal RNA gene, partial sequence; internal transcribed spacer 1, 5.8S ribosomal RNA gene and internal transcribed spacer 2, complete sequence; and 26S ribosomal RNA gene, partial sequence	813	813	100%	0.0	89%	
AY239975.1	Dendrobium junceum 18S ribosomal RNA gene, partial sequence; internal transcribed spacer 1, 5.8S ribosomal RNA gene and internal transcribed spacer 2, complete sequence; and 26S ribosomal RNA gene, partial sequence	808	808	100%	0.0	88%	
AY239978.1	Dendrobium leonis 18S ribosomal RNA gene, partial sequence; internal transcribed spacer 1, 5.8S ribosomal RNA gene and internal transcribed spacer 2, complete sequence; and 26S ribosomal RNA gene, partial sequence	798	798	100%	0.0	88%	
HM054548.1	Dendrobium anceps voucher SBB-0301 18S ribosomal RNA gene, partial sequence; internal transcribed spacer 1, 5.8S ribosomal RNA gene, and internal transcribed spacer 2, complete sequence; and 26S ribosomal RNA gene, partial sequence	795	795	97%	0.0	88%	
EU840698.1	Dendrobium quadrangulare internal transcribed spacer 1, 5.8S ribosomal RNA gene, and internal transcribed spacer 2, complete sequence	791	791	89%	0.0	91%	
AF521609.1	Dendrobium equitans internal transcribed spacer 1, 5.8S ribosomal RNA gene, and internal transcribed spacer 2, complete sequence >gb|EU840701.1| Dendrobium equitans internal transcribed spacer 1, 5.8S ribosomal RNA gene, and internal transcribed spacer 2, complete sequence	780	780	89%	0.0	90%	
[illegible]	[illegible]	[illegible]	[illegible]	[illegible]	[illegible]	[illegible]	

REVIEW | BLAST results 93+36... | Document2 - Micro... | NCBI Blast:AY24000... | BMC | Additional files | 17:27

NCBI Blast:AY240003.1 Dendrobium usterioides - Mozilla Firefox

file:///G:/THESIS/NCBI DOWNLOADED MATK SEQUENCES/ITS/BLAST results 93+36 sequences/NCBI BlastAY240003_1 Dendrobium usterioides_htm.htm

Legend for links to other resources: UniGene GEO Gene Structure Map Viewer PubChem BioAssay

Sequences producing significant alignments:

Accession	Description	Max score	Total score	Query coverage	E value	Max ident	Links
AY240003.1	Dendrobium usterioides 18S ribosomal RNA gene, partial sequence; internal transcribed spacer 1, 5.8S ribosomal RNA gene and internal transcribed spacer 2, complete sequence; and 26S ribosomal RNA gene, partial sequence	1225	1225	100%	0.0	100%	
AY239975.1	Dendrobium junceum 18S ribosomal RNA gene, partial sequence; internal transcribed spacer 1, 5.8S ribosomal RNA gene and internal transcribed spacer 2, complete sequence; and 26S ribosomal RNA gene, partial sequence	974	974	100%	0.0	92%	
AY239969.1	Dendrobium goldfinchii 18S ribosomal RNA gene, partial sequence; internal transcribed spacer 1, 5.8S ribosomal RNA gene and internal transcribed spacer 2, complete sequence; and 26S ribosomal RNA gene, partial sequence	941	941	100%	0.0	92%	
AY239988.1	Dendrobium philippinense 18S ribosomal RNA gene, partial sequence; internal transcribed spacer 1, 5.8S ribosomal RNA gene and internal transcribed spacer 2, complete sequence; and 26S ribosomal RNA gene, partial sequence	928	928	100%	0.0	91%	
AF521609.1	Dendrobium equitans internal transcribed spacer 1, 5.8S ribosomal RNA gene, and internal transcribed spacer 2, complete sequence >gb|EU840701.1| Dendrobium equitans internal transcribed spacer 1, 5.8S ribosomal RNA gene, and internal transcribed spacer 2, complete sequence	928	928	96%	0.0	92%	
AY239961.1	Dendrobium confusum 18S ribosomal RNA gene, partial sequence; internal transcribed spacer 1, 5.8S ribosomal RNA gene and internal transcribed spacer 2, complete sequence; and 26S ribosomal RNA gene, partial sequence	922	922	100%	0.0	91%	
AY239989.1	Dendrobium quadrangulare 18S ribosomal RNA gene, partial sequence; internal transcribed spacer 1, 5.8S ribosomal RNA gene and internal transcribed spacer 2, complete sequence; and 26S ribosomal RNA gene, partial sequence	905	905	100%	0.0	91%	
AY239951.1	Dendrobium aloifolium 18S ribosomal RNA gene, partial sequence; internal transcribed spacer 1, 5.8S ribosomal RNA gene and internal transcribed spacer 2, complete sequence; and 26S ribosomal RNA gene, partial sequence	902	902	100%	0.0	91%	
AY239972.1	Dendrobium indivisum 18S ribosomal RNA gene, partial sequence; internal transcribed spacer 1, 5.8S ribosomal RNA gene and internal transcribed spacer 2, complete sequence; and 26S ribosomal RNA gene, partial sequence	891	891	100%	0.0	90%	
AY239953.1	Dendrobium anceps 18S ribosomal RNA gene, partial sequence; internal transcribed spacer 1, 5.8S ribosomal RNA gene and internal transcribed spacer 2, complete sequence; and 26S ribosomal RNA gene, partial sequence	880	880	99%	0.0	90%	
EU840698.1	Dendrobium quadrangulare internal transcribed spacer 1, 5.8S ribosomal RNA gene, and internal transcribed spacer 2, complete sequence	869	869	96%	0.0	91%	
HM054548.1	Dendrobium anceps voucher SBB-0301 18S ribosomal RNA gene, partial sequence; internal transcribed spacer 1, 5.8S ribosomal RNA gene, and internal transcribed spacer 2, complete sequence; and 26S ribosomal RNA gene, partial sequence	865	865	97%	0.0	90%	
AY239978.1	Dendrobium leonis 18S ribosomal RNA gene, partial sequence; internal transcribed spacer 1, 5.8S ribosomal RNA gene and internal transcribed spacer 2, complete sequence; and 26S ribosomal RNA gene, partial sequence	848	848	100%	0.0	89%	
[illegible]	[illegible]	[illegible]	[illegible]	[illegible]	[illegible]	[illegible]	

REVIEW | BLAST results 93+36... | Document2 - Micro... | NCBI Blast:AY24000... | BMC | Additional files | 17:28

NCBI Blast:EU840694.1 Dendrobium victoriaereginae - Mozilla Firefox

file:///G:/THESIS/NCBI DOWNLOADED MATK SEQUENCES/ITS/BLAST results 93+36 sequences/NCBI BlastEU840694_1 Dendrobium victoriaereginae_htm

Legend for links to other resources: UniGene GEO Gene Structure Map Viewer PubChem BioAssay

Sequences producing significant alignments:

Accession	Description	Max score	Total score	Query coverage	E value	Max ident	Links
EU840694.1	Dendrobium victoriae-reginae internal transcribed spacer 1, 5.8S ribosomal RNA gene, and internal transcribed spacer 2, complete sequence	1184	1184	100%	0.0	100%	
HM590386.1	Dendrobium miyakei voucher NCHU-D89331201-1020 18S ribosomal RNA gene, partial sequence; internal transcribed spacer 1, 5.8S ribosomal RNA gene, and internal transcribed spacer 2, complete sequence; and 26S ribosomal RNA gene, partial sequence	1146	1146	100%	0.0	98%	
AY239970.1	Dendrobium goldschmidtianum 18S ribosomal RNA gene, partial sequence; internal transcribed spacer 1, 5.8S ribosomal RNA gene and internal transcribed spacer 2, complete sequence; and 26S ribosomal RNA gene, partial sequence	1146	1146	100%	0.0	98%	
AF521614.1	Dendrobium miyakei internal transcribed spacer 1, 5.8S ribosomal RNA gene, and internal transcribed spacer 2, complete sequence	1146	1146	100%	0.0	98%	
EU840695.1	Dendrobium goldschmidtianum internal transcribed spacer 1, 5.8S ribosomal RNA gene, and internal transcribed spacer 2, complete sequence	1140	1140	100%	0.0	98%	
HM590385.1	Dendrobium chameleon voucher NCHU-D89331201-1019 18S ribosomal RNA gene, partial sequence; internal transcribed spacer 1, 5.8S ribosomal RNA gene, and internal transcribed spacer 2, complete sequence; and 26S ribosomal RNA gene, partial sequence	1018	1018	100%	0.0	95%	
AY239994.1	Dendrobium serratilabium 18S ribosomal RNA gene, partial sequence; internal transcribed spacer 1, 5.8S ribosomal RNA gene and internal transcribed spacer 2, complete sequence; and 26S ribosomal RNA gene, partial sequence	1016	1016	100%	0.0	95%	
AY239960.1	Dendrobium chameleon 18S ribosomal RNA gene, partial sequence; internal transcribed spacer 1, 5.8S ribosomal RNA gene and internal transcribed spacer 2, complete sequence; and 26S ribosomal RNA gene, partial sequence	989	989	100%	0.0	94%	
AY240006.1	Dendrobium yeageri 18S ribosomal RNA gene, partial sequence; internal transcribed spacer 1, 5.8S ribosomal RNA gene and internal transcribed spacer 2, complete sequence; and 26S ribosomal RNA gene, partial sequence	983	983	100%	0.0	94%	
AF521607.1	Dendrobium chameleon internal transcribed spacer 1, 5.8S ribosomal RNA gene, and internal transcribed spacer 2, complete sequence	977	977	100%	0.0	94%	
AY239958.1	Dendrobium ceraula 18S ribosomal RNA gene, partial sequence; internal transcribed spacer 1, 5.8S ribosomal RNA gene and internal transcribed spacer 2, complete sequence; and 26S ribosomal RNA gene, partial sequence	970	970	100%	0.0	94%	
AY240004.1	Dendrobium victoriae-reginae 18S ribosomal RNA gene, partial sequence; internal transcribed spacer 1, 5.8S ribosomal RNA gene and internal transcribed spacer 2, complete sequence; and 26S ribosomal RNA gene, partial sequence	965	965	100%	0.0	93%	
AY239976.1	Dendrobium lancifolium 18S ribosomal RNA gene, partial sequence; internal transcribed spacer 1, 5.8S ribosomal RNA gene and internal transcribed spacer 2, complete sequence; and 26S ribosomal RNA gene, partial sequence	861	861	100%	0.0	90%	
AY239957.1	Dendrobium cauliculimentum 18S ribosomal RNA gene, partial sequence; internal transcribed spacer 1, 5.8S ribosomal RNA gene and internal transcribed spacer 2, complete sequence; and 26S ribosomal RNA gene, partial sequence	813	813	99%	0.0	89%	

NCBI Blast:AY240005.1 Dendrobium violaceum - Mozilla Firefox

file:///G:/THESIS/NCBI DOWNLOADED MATK SEQUENCES/ITS/BLAST results 93+36 sequences/NCBI BlastAY240005_1 Dendrobium violaceum_htm.htm

Legend for links to other resources: UniGene GEO Gene Structure Map Viewer PubChem BioAssay

Sequences producing significant alignments:

Accession	Description	Max score	Total score	Query coverage	E value	Max ident	Links
AY240005.1	Dendrobium violaceum 18S ribosomal RNA gene, partial sequence; internal transcribed spacer 1, 5.8S ribosomal RNA gene and internal transcribed spacer 2, complete sequence; and 26S ribosomal RNA gene, partial sequence	1166	1166	100%	0.0	100%	
AY239964.1	Dendrobium cyanocentrum 18S ribosomal RNA gene, partial sequence; internal transcribed spacer 1, 5.8S ribosomal RNA gene and internal transcribed spacer 2, complete sequence; and 26S ribosomal RNA gene, partial sequence	569	569	67%	6e-159	91%	
AY240000.1	Dendrobium subuliferum 18S ribosomal RNA gene, partial sequence; internal transcribed spacer 1, 5.8S ribosomal RNA gene and internal transcribed spacer 2, complete sequence; and 26S ribosomal RNA gene, partial sequence	547	547	67%	3e-152	90%	
AY239976.1	Dendrobium lancifolium 18S ribosomal RNA gene, partial sequence; internal transcribed spacer 1, 5.8S ribosomal RNA gene and internal transcribed spacer 2, complete sequence; and 26S ribosomal RNA gene, partial sequence	547	547	67%	3e-152	89%	
AY240006.1	Dendrobium yeageri 18S ribosomal RNA gene, partial sequence; internal transcribed spacer 1, 5.8S ribosomal RNA gene and internal transcribed spacer 2, complete sequence; and 26S ribosomal RNA gene, partial sequence	536	536	67%	6e-149	89%	
AY239958.1	Dendrobium ceraula 18S ribosomal RNA gene, partial sequence; internal transcribed spacer 1, 5.8S ribosomal RNA gene and internal transcribed spacer 2, complete sequence; and 26S ribosomal RNA gene, partial sequence	536	536	67%	6e-149	89%	
HM590385.1	Dendrobium chameleon voucher NCHU-D89331201-1019 18S ribosomal RNA gene, partial sequence; internal transcribed spacer 1, 5.8S ribosomal RNA gene, and internal transcribed spacer 2, complete sequence; and 26S ribosomal RNA gene, partial sequence	531	531	67%	3e-147	89%	
AY239994.1	Dendrobium serratilabium 18S ribosomal RNA gene, partial sequence; internal transcribed spacer 1, 5.8S ribosomal RNA gene and internal transcribed spacer 2, complete sequence; and 26S ribosomal RNA gene, partial sequence	531	531	67%	3e-147	89%	
AY239990.1	Dendrobium aff. rarum Clements 5613 18S ribosomal RNA gene, partial sequence; internal transcribed spacer 1, 5.8S ribosomal RNA gene and internal transcribed spacer 2, complete sequence; and 26S ribosomal RNA gene, partial sequence	531	531	67%	3e-147	89%	
AY239960.1	Dendrobium chameleon 18S ribosomal RNA gene, partial sequence; internal transcribed spacer 1, 5.8S ribosomal RNA gene and internal transcribed spacer 2, complete sequence; and 26S ribosomal RNA gene, partial sequence	531	531	67%	3e-147	89%	
AY240004.1	Dendrobium victoriae-reginae 18S ribosomal RNA gene, partial sequence; internal transcribed spacer 1, 5.8S ribosomal RNA gene and internal transcribed spacer 2, complete sequence; and 26S ribosomal RNA gene, partial sequence	525	525	67%	1e-145	89%	
AY239950.1	Dendrobium agathodaemonis 18S ribosomal RNA gene, partial sequence; internal transcribed spacer 1, 5.8S ribosomal RNA gene and internal transcribed spacer 2, complete sequence; and 26S ribosomal RNA gene, partial sequence	525	525	67%	1e-145	89%	
AY239954.1	Dendrobium bracteosum 18S ribosomal RNA gene, partial sequence; internal transcribed spacer 1, 5.8S ribosomal RNA gene and internal transcribed spacer 2, complete sequence; and 26S ribosomal RNA gene, partial sequence	523	523	67%	5e-145	89%	

NCBI Blast:AY240006.1 Dendrobium yeageri - Mozilla Firefox

file:///G:/THESIS/NCBI DOWNLOADED MATK SEQUENCES/ITS/BLAST results 93+36 sequences/NCBI BlastAY240006_1 Dendrobium yeageri_htm.htm

Legend for links to other resources: UniGene GEO Gene Structure Map Viewer PubChem BioAssay

Sequences producing significant alignments:

Accession	Description	Max score	Total score	Query coverage	E value	Max ident	Links
AY240006.1	Dendrobium yeageri 18S ribosomal RNA gene, partial sequence; internal transcribed spacer 1, 5.8S ribosomal RNA gene and internal transcribed spacer 2, complete sequence; and 26S ribosomal RNA gene, partial sequence	1225	1225	100%	0.0	100%	
AY239960.1	Dendrobium chameleon 18S ribosomal RNA gene, partial sequence; internal transcribed spacer 1, 5.8S ribosomal RNA gene and internal transcribed spacer 2, complete sequence; and 26S ribosomal RNA gene, partial sequence	1214	1214	100%	0.0	99%	
AY239958.1	Dendrobium ceraula 18S ribosomal RNA gene, partial sequence; internal transcribed spacer 1, 5.8S ribosomal RNA gene and internal transcribed spacer 2, complete sequence; and 26S ribosomal RNA gene, partial sequence	1212	1212	100%	0.0	99%	
AY240004.1	Dendrobium victoriae-reginae 18S ribosomal RNA gene, partial sequence; internal transcribed spacer 1, 5.8S ribosomal RNA gene and internal transcribed spacer 2, complete sequence; and 26S ribosomal RNA gene, partial sequence	1206	1206	100%	0.0	99%	
AF521607.1	Dendrobium chameleon internal transcribed spacer 1, 5.8S ribosomal RNA gene, and internal transcribed spacer 2, complete sequence	1164	1164	96%	0.0	99%	
AY239994.1	Dendrobium serratilabium 18S ribosomal RNA gene, partial sequence; internal transcribed spacer 1, 5.8S ribosomal RNA gene and internal transcribed spacer 2, complete sequence; and 26S ribosomal RNA gene, partial sequence	1120	1120	100%	0.0	97%	
HM590385.1	Dendrobium chameleon voucher NCHU-D89331201-1019 18S ribosomal RNA gene, partial sequence; internal transcribed spacer 1, 5.8S ribosomal RNA gene, and internal transcribed spacer 2, complete sequence; and 26S ribosomal RNA gene, partial sequence	1114	1114	100%	0.0	96%	
HM590386.1	Dendrobium miyakei voucher NCHU-D89331201-1020 18S ribosomal RNA gene, partial sequence; internal transcribed spacer 1, 5.8S ribosomal RNA gene, and internal transcribed spacer 2, complete sequence; and 26S ribosomal RNA gene, partial sequence	1055	1055	100%	0.0	95%	
AY239970.1	Dendrobium goldschmidtianum 18S ribosomal RNA gene, partial sequence; internal transcribed spacer 1, 5.8S ribosomal RNA gene and internal transcribed spacer 2, complete sequence; and 26S ribosomal RNA gene, partial sequence	1055	1055	100%	0.0	95%	
AF521614.1	Dendrobium miyakei internal transcribed spacer 1, 5.8S ribosomal RNA gene, and internal transcribed spacer 2, complete sequence	1011	1011	96%	0.0	95%	
EU840695.1	Dendrobium goldschmidtianum internal transcribed spacer 1, 5.8S ribosomal RNA gene, and internal transcribed spacer 2, complete sequence	1005	1005	96%	0.0	95%	
AY239976.1	Dendrobium lancifolium 18S ribosomal RNA gene, partial sequence; internal transcribed spacer 1, 5.8S ribosomal RNA gene and internal transcribed spacer 2, complete sequence; and 26S ribosomal RNA gene, partial sequence	992	992	100%	0.0	93%	
EU840694.1	Dendrobium victoriae-reginae internal transcribed spacer 1, 5.8S ribosomal RNA gene, and internal transcribed spacer 2, complete sequence	983	983	96%	0.0	94%	
AY239980.1	Dendrobium mohlianum 18S ribosomal RNA gene, partial sequence; internal transcribed spacer 1, 5.8S ribosomal RNA gene and internal transcribed spacer 2, complete sequence; and 26S ribosomal RNA gene, partial sequence	915	915	100%	0.0	91%	

NCBI Blast:D aduncum - Mozilla Firefox

file:///G:/THESIS/NCBI DOWNLOADED MATK SEQUENCES/ITS/BLAST results 93+36 sequences/NCBI BlastD aduncum_htm.htm

Legend for links to other resources: UniGene GEO Gene Structure Map Viewer PubChem BioAssay

Sequences producing significant alignments:

Accession	Description	Max score	Total score	Query coverage	E value	Max ident	Links
GU339110.1	Dendrobium aduncum internal transcribed spacer 1, partial sequence; 5.8S ribosomal RNA gene, complete sequence; and internal transcribed spacer 2, partial sequence	1144	1144	94%	0.0	99%	
HM590391.1	Dendrobium candidum voucher NCHU-D89331201-1028 18S ribosomal RNA gene, partial sequence; internal transcribed spacer 1, 5.8S ribosomal RNA gene, and internal transcribed spacer 2, complete sequence; and 26S ribosomal RNA gene, partial sequence	1077	1077	100%	0.0	95%	
HM590367.1	Dendrobium tosaense voucher NCHU-D89331201-1001 18S ribosomal RNA gene, partial sequence; internal transcribed spacer 1, 5.8S ribosomal RNA gene, and internal transcribed spacer 2, complete sequence; and 26S ribosomal RNA gene, partial sequence	1059	1059	100%	0.0	95%	
HM590382.1	Dendrobium nobile voucher NCHU-D89331201-1016 18S ribosomal RNA gene, partial sequence; internal transcribed spacer 1, 5.8S ribosomal RNA gene, and internal transcribed spacer 2, complete sequence; and 26S ribosomal RNA gene, partial sequence	1014	1014	100%	0.0	94%	
FJ384743.1	Dendrobium flexicaule internal transcribed spacer 1, partial sequence; 5.8S ribosomal RNA gene, complete sequence; and internal transcribed spacer 2, partial sequence	1014	1014	94%	0.0	95%	
FJ384723.1	Dendrobium officinale strain T1 internal transcribed spacer 1, partial sequence; 5.8S ribosomal RNA gene, complete sequence; and internal transcribed spacer 2, partial sequence	1014	1014	94%	0.0	95%	
FJ384724.1	Dendrobium officinale strain T2 internal transcribed spacer 1, partial sequence; 5.8S ribosomal RNA gene, complete sequence; and internal transcribed spacer 2, partial sequence >gb|FJ530944.1| Dendrobium officinale strain T4 internal transcribed spacer 1, partial sequence; 5.8S ribosomal RNA gene, complete sequence; and internal transcribed spacer 2, partial sequence >gb|FJ530945.1| Dendrobium officinale strain T5 internal transcribed spacer 1, partial sequence; 5.8S ribosomal RNA gene, complete sequence; and internal transcribed spacer 2, partial sequence >gb|FJ530946.1| Dendrobium officinale strain T6 internal transcribed spacer 1, partial sequence; 5.8S ribosomal RNA gene, complete sequence; and internal transcribed spacer 2, partial sequence >gb|FJ530947.1| Dendrobium officinale strain T7 internal transcribed spacer 1, partial sequence; 5.8S ribosomal RNA gene, complete sequence; and internal transcribed spacer 2, partial sequence >gb|FJ588871.1| Dendrobium officinale strain T3 internal transcribed spacer 1, partial sequence; 5.8S ribosomal RNA gene, complete sequence; and internal transcribed spacer 2, partial sequence >gb|FJ588872.1| Dendrobium officinale strain T8 internal transcribed spacer 1, partial sequence; 5.8S ribosomal RNA gene, complete sequence; and internal transcribed spacer 2, partial sequence >gb|FJ588873.1| Dendrobium officinale strain T9 internal transcribed spacer 1, partial sequence; 5.8S ribosomal RNA gene, complete sequence; and internal transcribed spacer 2, partial sequence >gb|GU339109.1| Dendrobium officinale internal transcribed spacer 1, partial sequence; 5.8S ribosomal RNA gene, complete sequence; and internal transcribed spacer 2, partial sequence	1014	1014	94%	0.0	95%	
EF221854.1	Dendrobium officinale voucher XYD05006 internal transcribed spacer 1, partial sequence; 5.8S ribosomal RNA gene, complete sequence; and internal transcribed spacer 2, partial sequence	1009	1009	94%	0.0	95%	
	Dendrobium tosaense internal transcribed spacer 1, 5.8S ribosomal RNA gene, and internal transcribed spacer 2, complete sequence						

NCBI Blast:D amoenum - Mozilla Firefox

file:///G:/THESIS/NCBI DOWNLOADED MATK SEQUENCES/ITS/BLAST results 93+36 sequences/NCBI BlastD amoenum_htm.htm

Legend for links to other resources: UniGene GEO Gene Structure Map Viewer PubChem BioAssay

Sequences producing significant alignments:

Accession	Description	Max score	Total score	Query coverage	E value	Max ident	Links
HM054539.1	Dendrobium amoenum voucher SBB-0142 internal transcribed spacer 1, partial sequence; 5.8S ribosomal RNA gene and internal transcribed spacer 2, complete sequence; and 26S ribosomal RNA gene, partial sequence	1242	1242	99%	0.0	100%	
HM054540.1	Dendrobium amoenum voucher SBB-0247 internal transcribed spacer 1, partial sequence; 5.8S ribosomal RNA gene and internal transcribed spacer 2, complete sequence; and 26S ribosomal RNA gene, partial sequence	1242	1242	99%	0.0	100%	
HM054535.1	Dendrobium amoenum voucher SBB-0137 internal transcribed spacer 1, partial sequence; 5.8S ribosomal RNA gene and internal transcribed spacer 2, complete sequence; and 26S ribosomal RNA gene, partial sequence	1240	1240	99%	0.0	100%	
HM054538.1	Dendrobium amoenum voucher SBB-0140 internal transcribed spacer 1, partial sequence; 5.8S ribosomal RNA gene and internal transcribed spacer 2, complete sequence; and 26S ribosomal RNA gene, partial sequence	1238	1238	99%	0.0	99%	
HM054534.1	Dendrobium amoenum voucher SBB-0135 internal transcribed spacer 1, partial sequence; 5.8S ribosomal RNA gene and internal transcribed spacer 2, complete sequence; and 26S ribosomal RNA gene, partial sequence	1236	1236	99%	0.0	99%	
HM054536.1	Dendrobium amoenum voucher SBB-0138 internal transcribed spacer 1, partial sequence; 5.8S ribosomal RNA gene and internal transcribed spacer 2, complete sequence; and 26S ribosomal RNA gene, partial sequence	1236	1236	99%	0.0	99%	
HM054541.1	Dendrobium amoenum voucher SBB-0248 internal transcribed spacer 1, partial sequence; 5.8S ribosomal RNA gene and internal transcribed spacer 2, complete sequence; and 26S ribosomal RNA gene, partial sequence	1223	1223	97%	0.0	100%	
HM054542.1	Dendrobium amoenum voucher SBB-0249 internal transcribed spacer 1, partial sequence; 5.8S ribosomal RNA gene and internal transcribed spacer 2, complete sequence; and 26S ribosomal RNA gene, partial sequence	1221	1221	97%	0.0	100%	
HM054537.1	Dendrobium amoenum voucher SBB-0139 internal transcribed spacer 1, partial sequence; 5.8S ribosomal RNA gene and internal transcribed spacer 2, complete sequence; and 26S ribosomal RNA gene, partial sequence	1212	1212	97%	0.0	99%	
HM054543.1	Dendrobium amoenum voucher SBB-0029 internal transcribed spacer 1, partial sequence; 5.8S ribosomal RNA gene and internal transcribed spacer 2, complete sequence; and 26S ribosomal RNA gene, partial sequence	1208	1208	96%	0.0	99%	
HM054544.1	Dendrobium amoenum voucher SBB-0560 internal transcribed spacer 1, partial sequence; 5.8S ribosomal RNA gene and internal transcribed spacer 2, complete sequence; and 26S ribosomal RNA gene, partial sequence	1195	1195	96%	0.0	99%	
HM054546.1	Dendrobium amoenum voucher SBB-0576 internal transcribed spacer 1, partial sequence; 5.8S ribosomal RNA gene and internal transcribed spacer 2, complete sequence; and 26S ribosomal RNA gene, partial sequence	1190	1190	95%	0.0	99%	
HM054545.1	Dendrobium amoenum voucher SBB-0575 internal transcribed spacer 1, partial sequence; 5.8S ribosomal RNA gene and internal transcribed spacer 2, complete sequence; and 26S ribosomal RNA gene, partial sequence	1190	1190	95%	0.0	99%	

REVIEW | BLAST results 93+36... | Document2 - Micro... | NCBI Blast:D amoen... | BMC | Additional files | 17:29

NCBI Blast:D anceps - Mozilla Firefox

file:///G:/THESIS/NCBI DOWNLOADED MATK SEQUENCES/ITS/BLAST results 93+36 sequences/NCBI BlastD anceps_htm.htm#300250534

Legend for links to other resources: UniGene GEO Gene Structure Map Viewer PubChem BioAssay

Sequences producing significant alignments:

Accession	Description	Max score	Total score	Query coverage	E value	Max ident	Links
HM054548.1	Dendrobium anceps voucher SBB-0301 18S ribosomal RNA gene, partial sequence; internal transcribed spacer 1, 5.8S ribosomal RNA gene, and internal transcribed spacer 2, complete sequence; and 26S ribosomal RNA gene, partial sequence	1218	1218	99%	0.0	100%	
AY239953.1	Dendrobium anceps 18S ribosomal RNA gene, partial sequence; internal transcribed spacer 1, 5.8S ribosomal RNA gene and internal transcribed spacer 2, complete sequence; and 26S ribosomal RNA gene, partial sequence	1205	1205	99%	0.0	99%	
AY239951.1	Dendrobium aloifolium 18S ribosomal RNA gene, partial sequence; internal transcribed spacer 1, 5.8S ribosomal RNA gene and internal transcribed spacer 2, complete sequence; and 26S ribosomal RNA gene, partial sequence	1149	1149	99%	0.0	98%	
HM054547.1	Dendrobium anceps voucher SBB-0119 internal transcribed spacer 1, partial sequence; 5.8S ribosomal RNA gene, complete sequence; and internal transcribed spacer 2, partial sequence	1147	1147	93%	0.0	100%	
AY239972.1	Dendrobium indivisum 18S ribosomal RNA gene, partial sequence; internal transcribed spacer 1, 5.8S ribosomal RNA gene and internal transcribed spacer 2, complete sequence; and 26S ribosomal RNA gene, partial sequence	1127	1127	99%	0.0	97%	
AY239961.1	Dendrobium confusum 18S ribosomal RNA gene, partial sequence; internal transcribed spacer 1, 5.8S ribosomal RNA gene and internal transcribed spacer 2, complete sequence; and 26S ribosomal RNA gene, partial sequence	1110	1110	99%	0.0	97%	
AY239978.1	Dendrobium leonis 18S ribosomal RNA gene, partial sequence; internal transcribed spacer 1, 5.8S ribosomal RNA gene and internal transcribed spacer 2, complete sequence; and 26S ribosomal RNA gene, partial sequence	1029	1029	99%	0.0	95%	
DQ058801.1	Dendrobium terminale voucher DY-ML03-1 internal transcribed spacer 1, 5.8S ribosomal RNA gene, and internal transcribed spacer 2, complete sequence	1022	1022	97%	0.0	95%	
AF362034.1	Dendrobium acinaciforme internal transcribed spacer 1, partial sequence; 5.8S ribosomal RNA gene, complete sequence; and internal transcribed spacer 2, partial sequence >gb|EF629321.1| Dendrobium jenkinsii internal transcribed spacer 1, partial sequence; 5.8S ribosomal RNA gene, complete sequence; and internal transcribed spacer 2, partial sequence	990	990	97%	0.0	94%	
AY239975.1	Dendrobium junceum 18S ribosomal RNA gene, partial sequence; internal transcribed spacer 1, 5.8S ribosomal RNA gene and internal transcribed spacer 2, complete sequence; and 26S ribosomal RNA gene, partial sequence	917	917	99%	0.0	91%	
AY239988.1	Dendrobium philippinense 18S ribosomal RNA gene, partial sequence; internal transcribed spacer 1, 5.8S ribosomal RNA gene and internal transcribed spacer 2, complete sequence; and 26S ribosomal RNA gene, partial sequence	915	915	99%	0.0	91%	
AY239969.1	Dendrobium goldfinchii 18S ribosomal RNA gene, partial sequence; internal transcribed spacer 1, 5.8S ribosomal RNA gene and internal transcribed spacer 2, complete sequence; and 26S ribosomal RNA gene, partial sequence	909	909	98%	0.0	92%	
AF521609.1	Dendrobium equitans internal transcribed spacer 1, 5.8S ribosomal RNA gene, and internal transcribed spacer 2, complete sequence >gb|EU840701.1| Dendrobium equitans internal transcribed spacer 1, 5.8S ribosomal RNA gene, and intern[illegible]	[illegible]8	898	97%	0.0	91%	

REVIEW | BLAST results 93+36... | Document2 - Micro... | NCBI Blast:D anceps... | BMC | Additional files | 17:33

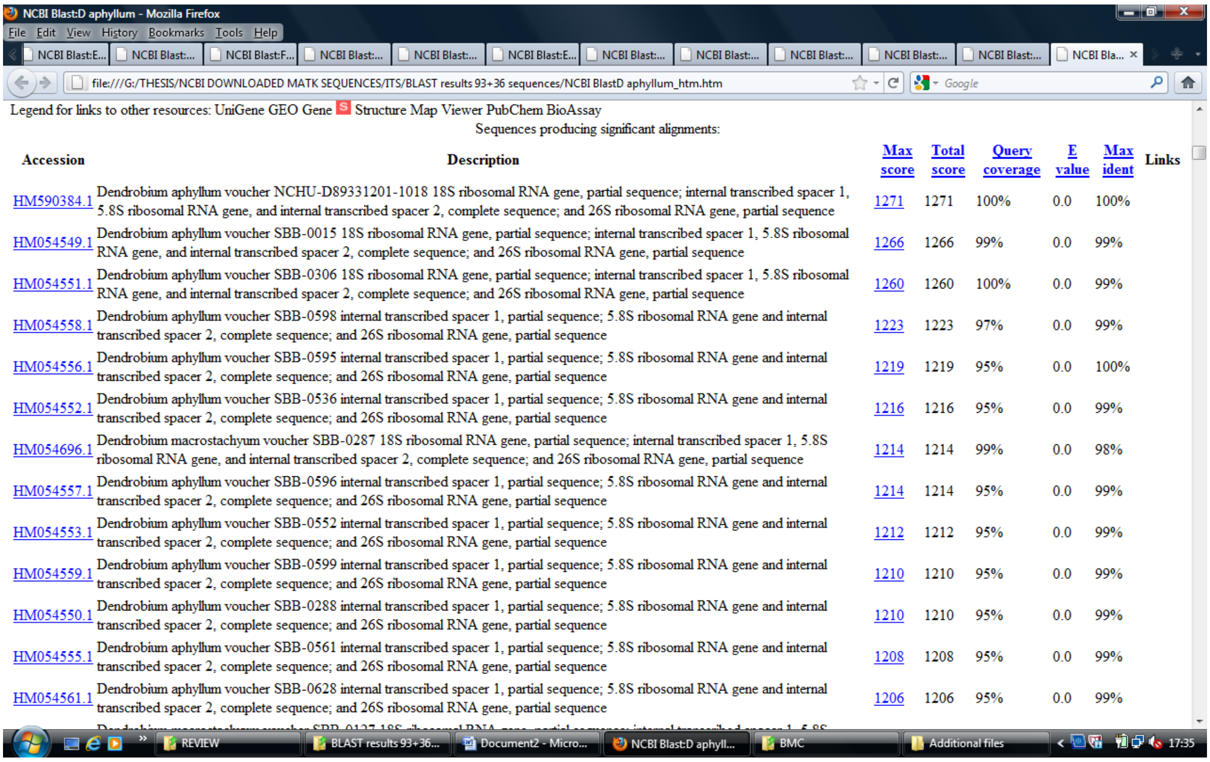

Legend for links to other resources: UniGene GEO Gene Structure Map Viewer PubChem BioAssay

Sequences producing significant alignments:

Accession	Description	Max score	Total score	Query coverage	E value	Max ident	Links
HM590384.1	Dendrobium aphyllum voucher NCHU-D89331201-1018 18S ribosomal RNA gene, partial sequence; internal transcribed spacer 1, 5.8S ribosomal RNA gene, and internal transcribed spacer 2, complete sequence; and 26S ribosomal RNA gene, partial sequence	1271	1271	100%	0.0	100%	
HM054549.1	Dendrobium aphyllum voucher SBB-0015 18S ribosomal RNA gene, partial sequence; internal transcribed spacer 1, 5.8S ribosomal RNA gene, and internal transcribed spacer 2, complete sequence; and 26S ribosomal RNA gene, partial sequence	1266	1266	99%	0.0	99%	
HM054551.1	Dendrobium aphyllum voucher SBB-0306 18S ribosomal RNA gene, partial sequence; internal transcribed spacer 1, 5.8S ribosomal RNA gene, and internal transcribed spacer 2, complete sequence; and 26S ribosomal RNA gene, partial sequence	1260	1260	100%	0.0	99%	
HM054558.1	Dendrobium aphyllum voucher SBB-0598 internal transcribed spacer 1, partial sequence; 5.8S ribosomal RNA gene and internal transcribed spacer 2, complete sequence; and 26S ribosomal RNA gene, partial sequence	1223	1223	97%	0.0	99%	
HM054556.1	Dendrobium aphyllum voucher SBB-0595 internal transcribed spacer 1, partial sequence; 5.8S ribosomal RNA gene and internal transcribed spacer 2, complete sequence; and 26S ribosomal RNA gene, partial sequence	1219	1219	95%	0.0	100%	
HM054552.1	Dendrobium aphyllum voucher SBB-0536 internal transcribed spacer 1, partial sequence; 5.8S ribosomal RNA gene and internal transcribed spacer 2, complete sequence; and 26S ribosomal RNA gene, partial sequence	1216	1216	95%	0.0	99%	
HM054696.1	Dendrobium macrostachyum voucher SBB-0287 18S ribosomal RNA gene, partial sequence; internal transcribed spacer 1, 5.8S ribosomal RNA gene, and internal transcribed spacer 2, complete sequence; and 26S ribosomal RNA gene, partial sequence	1214	1214	99%	0.0	98%	
HM054557.1	Dendrobium aphyllum voucher SBB-0596 internal transcribed spacer 1, partial sequence; 5.8S ribosomal RNA gene and internal transcribed spacer 2, complete sequence; and 26S ribosomal RNA gene, partial sequence	1214	1214	95%	0.0	99%	
HM054553.1	Dendrobium aphyllum voucher SBB-0552 internal transcribed spacer 1, partial sequence; 5.8S ribosomal RNA gene and internal transcribed spacer 2, complete sequence; and 26S ribosomal RNA gene, partial sequence	1212	1212	95%	0.0	99%	
HM054559.1	Dendrobium aphyllum voucher SBB-0599 internal transcribed spacer 1, partial sequence; 5.8S ribosomal RNA gene and internal transcribed spacer 2, complete sequence; and 26S ribosomal RNA gene, partial sequence	1210	1210	95%	0.0	99%	
HM054550.1	Dendrobium aphyllum voucher SBB-0288 internal transcribed spacer 1, partial sequence; 5.8S ribosomal RNA gene and internal transcribed spacer 2, complete sequence; and 26S ribosomal RNA gene, partial sequence	1210	1210	95%	0.0	99%	
HM054555.1	Dendrobium aphyllum voucher SBB-0561 internal transcribed spacer 1, partial sequence; 5.8S ribosomal RNA gene and internal transcribed spacer 2, complete sequence; and 26S ribosomal RNA gene, partial sequence	1208	1208	95%	0.0	99%	
HM054561.1	Dendrobium aphyllum voucher SBB-0628 internal transcribed spacer 1, partial sequence; 5.8S ribosomal RNA gene and internal transcribed spacer 2, complete sequence; and 26S ribosomal RNA gene, partial sequence	1206	1206	95%	0.0	99%	

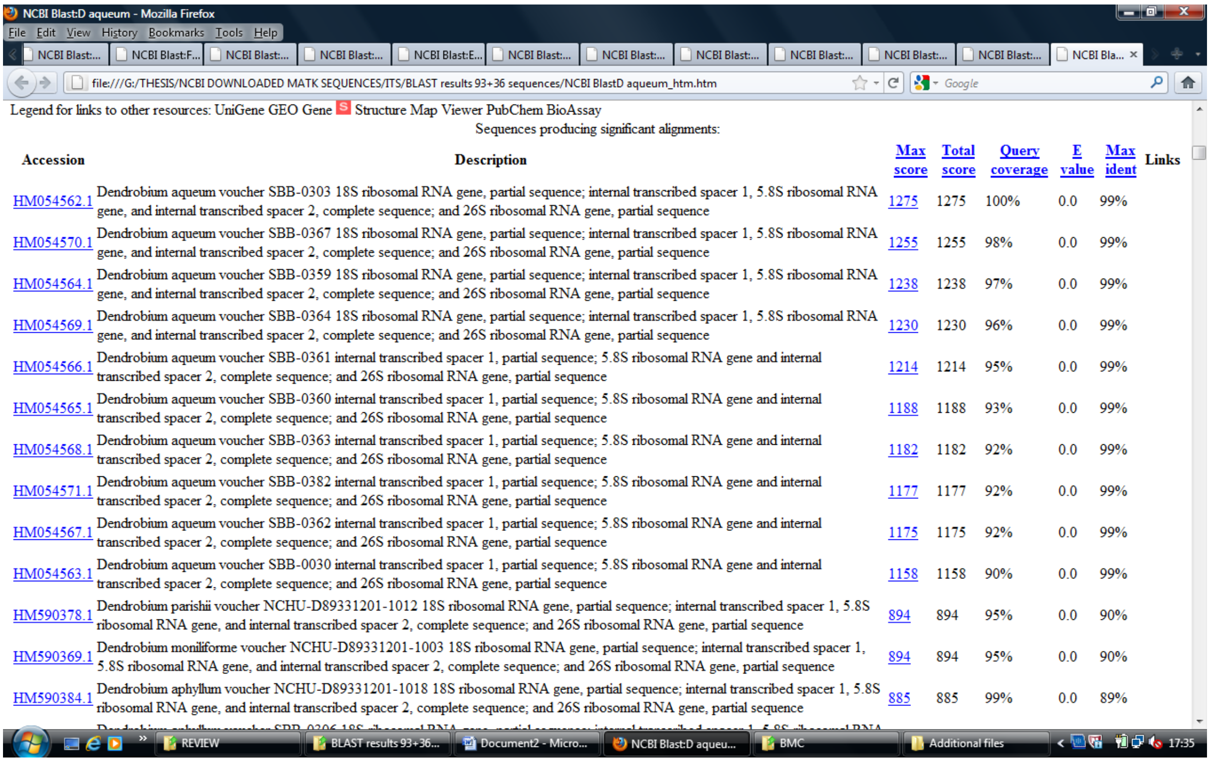

Legend for links to other resources: UniGene GEO Gene Structure Map Viewer PubChem BioAssay

Sequences producing significant alignments:

Accession	Description	Max score	Total score	Query coverage	E value	Max ident	Links
HM054562.1	Dendrobium aqueum voucher SBB-0303 18S ribosomal RNA gene, partial sequence; internal transcribed spacer 1, 5.8S ribosomal RNA gene, and internal transcribed spacer 2, complete sequence; and 26S ribosomal RNA gene, partial sequence	1275	1275	100%	0.0	99%	
HM054570.1	Dendrobium aqueum voucher SBB-0367 18S ribosomal RNA gene, partial sequence; internal transcribed spacer 1, 5.8S ribosomal RNA gene, and internal transcribed spacer 2, complete sequence; and 26S ribosomal RNA gene, partial sequence	1255	1255	98%	0.0	99%	
HM054564.1	Dendrobium aqueum voucher SBB-0359 18S ribosomal RNA gene, partial sequence; internal transcribed spacer 1, 5.8S ribosomal RNA gene, and internal transcribed spacer 2, complete sequence; and 26S ribosomal RNA gene, partial sequence	1238	1238	97%	0.0	99%	
HM054569.1	Dendrobium aqueum voucher SBB-0364 18S ribosomal RNA gene, partial sequence; internal transcribed spacer 1, 5.8S ribosomal RNA gene, and internal transcribed spacer 2, complete sequence; and 26S ribosomal RNA gene, partial sequence	1230	1230	96%	0.0	99%	
HM054566.1	Dendrobium aqueum voucher SBB-0361 internal transcribed spacer 1, partial sequence; 5.8S ribosomal RNA gene and internal transcribed spacer 2, complete sequence; and 26S ribosomal RNA gene, partial sequence	1214	1214	95%	0.0	99%	
HM054565.1	Dendrobium aqueum voucher SBB-0360 internal transcribed spacer 1, partial sequence; 5.8S ribosomal RNA gene and internal transcribed spacer 2, complete sequence; and 26S ribosomal RNA gene, partial sequence	1188	1188	93%	0.0	99%	
HM054568.1	Dendrobium aqueum voucher SBB-0363 internal transcribed spacer 1, partial sequence; 5.8S ribosomal RNA gene and internal transcribed spacer 2, complete sequence; and 26S ribosomal RNA gene, partial sequence	1182	1182	92%	0.0	99%	
HM054571.1	Dendrobium aqueum voucher SBB-0382 internal transcribed spacer 1, partial sequence; 5.8S ribosomal RNA gene and internal transcribed spacer 2, complete sequence; and 26S ribosomal RNA gene, partial sequence	1177	1177	92%	0.0	99%	
HM054567.1	Dendrobium aqueum voucher SBB-0362 internal transcribed spacer 1, partial sequence; 5.8S ribosomal RNA gene and internal transcribed spacer 2, complete sequence; and 26S ribosomal RNA gene, partial sequence	1175	1175	92%	0.0	99%	
HM054563.1	Dendrobium aqueum voucher SBB-0030 internal transcribed spacer 1, partial sequence; 5.8S ribosomal RNA gene and internal transcribed spacer 2, complete sequence; and 26S ribosomal RNA gene, partial sequence	1158	1158	90%	0.0	99%	
HM590378.1	Dendrobium parishii voucher NCHU-D89331201-1012 18S ribosomal RNA gene, partial sequence; internal transcribed spacer 1, 5.8S ribosomal RNA gene, and internal transcribed spacer 2, complete sequence; and 26S ribosomal RNA gene, partial sequence	894	894	95%	0.0	90%	
HM590369.1	Dendrobium moniliforme voucher NCHU-D89331201-1003 18S ribosomal RNA gene, partial sequence; internal transcribed spacer 1, 5.8S ribosomal RNA gene, and internal transcribed spacer 2, complete sequence; and 26S ribosomal RNA gene, partial sequence	894	894	95%	0.0	90%	
HM590384.1	Dendrobium aphyllum voucher NCHU-D89331201-1018 18S ribosomal RNA gene, partial sequence; internal transcribed spacer 1, 5.8S ribosomal RNA gene, and internal transcribed spacer 2, complete sequence; and 26S ribosomal RNA gene, partial sequence	885	885	99%	0.0	89%	

NCBI Blast:D barbatulum - Mozilla Firefox

file:///G:/THESIS/NCBI DOWNLOADED MATK SEQUENCES/ITS/BLAST results 93+36 sequences/NCBI BlastD barbatulum_htm.htm

Legend for links to other resources: UniGene GEO Gene Structure Map Viewer PubChem BioAssay

Sequences producing significant alignments:

Accession	Description	Max score	Total score	Query coverage	E value	Max ident	Links
HM054574.1	Dendrobium barbatulum voucher SBB-0409 internal transcribed spacer 1, partial sequence; 5.8S ribosomal RNA gene and internal transcribed spacer 2, complete sequence; and 26S ribosomal RNA gene, partial sequence	1236	1236	95%	0.0	99%	
HM054578.1	Dendrobium barbatulum voucher SBB-0415 internal transcribed spacer 1, partial sequence; 5.8S ribosomal RNA gene and internal transcribed spacer 2, complete sequence; and 26S ribosomal RNA gene, partial sequence	1234	1234	95%	0.0	99%	
HM054576.1	Dendrobium barbatulum voucher SBB-0411 internal transcribed spacer 1, partial sequence; 5.8S ribosomal RNA gene and internal transcribed spacer 2, complete sequence; and 26S ribosomal RNA gene, partial sequence	1234	1234	95%	0.0	99%	
HM054572.1	Dendrobium barbatulum voucher SBB-0407 internal transcribed spacer 1, partial sequence; 5.8S ribosomal RNA gene and internal transcribed spacer 2, complete sequence; and 26S ribosomal RNA gene, partial sequence	1234	1234	95%	0.0	99%	
HM054575.1	Dendrobium barbatulum voucher SBB-0410 internal transcribed spacer 1, partial sequence; 5.8S ribosomal RNA gene and internal transcribed spacer 2, complete sequence; and 26S ribosomal RNA gene, partial sequence	1234	1234	95%	0.0	99%	
HM054577.1	Dendrobium barbatulum voucher SBB-0413 internal transcribed spacer 1, partial sequence; 5.8S ribosomal RNA gene and internal transcribed spacer 2, complete sequence; and 26S ribosomal RNA gene, partial sequence	1229	1229	94%	0.0	99%	
HM054573.1	Dendrobium barbatulum voucher SBB-0408 internal transcribed spacer 1, partial sequence; 5.8S ribosomal RNA gene and internal transcribed spacer 2, complete sequence; and 26S ribosomal RNA gene, partial sequence	1225	1225	94%	0.0	99%	
HM054721.1	Dendrobium ovatum voucher SBB-0001 18S ribosomal RNA gene, partial sequence; internal transcribed spacer 1, 5.8S ribosomal RNA gene, and internal transcribed spacer 2, complete sequence; and 26S ribosomal RNA gene, partial sequence	1157	1157	96%	0.0	97%	
HM054734.1	Dendrobium ovatum voucher SBB-0005 18S ribosomal RNA gene, partial sequence; internal transcribed spacer 1, 5.8S ribosomal RNA gene, and internal transcribed spacer 2, complete sequence; and 26S ribosomal RNA gene, partial sequence	1142	1142	95%	0.0	97%	
HM054733.1	Dendrobium ovatum voucher SBB-0004 18S ribosomal RNA gene, partial sequence; internal transcribed spacer 1, 5.8S ribosomal RNA gene, and internal transcribed spacer 2, complete sequence; and 26S ribosomal RNA gene, partial sequence	1138	1138	95%	0.0	97%	
HM054728.1	Dendrobium ovatum voucher SBB-0422 18S ribosomal RNA gene, partial sequence; internal transcribed spacer 1, 5.8S ribosomal RNA gene, and internal transcribed spacer 2, complete sequence; and 26S ribosomal RNA gene, partial sequence	1138	1138	95%	0.0	97%	
HM054729.1	Dendrobium ovatum voucher SBB-0423 internal transcribed spacer 1, partial sequence; 5.8S ribosomal RNA gene and internal transcribed spacer 2, complete sequence; and 26S ribosomal RNA gene, partial sequence	1133	1133	95%	0.0	96%	
HM054726.1	Dendrobium ovatum voucher SBB-0420 18S ribosomal RNA gene, partial sequence; internal transcribed spacer 1, 5.8S ribosomal RNA gene, and internal transcribed spacer 2, complete sequence; and 26S ribosomal RNA gene, partial sequence	1133	1133	95%	0.0	96%	

NCBI Blast:D bensoniae - Mozilla Firefox

file:///G:/THESIS/NCBI DOWNLOADED MATK SEQUENCES/ITS/BLAST results 93+36 sequences/NCBI BlastD bensoniae_htm.htm

Legend for links to other resources: UniGene GEO Gene Structure Map Viewer PubChem BioAssay

Sequences producing significant alignments:

Accession	Description	Max score	Total score	Query coverage	E value	Max ident	Links
HM054579.1	Dendrobium bensoniae voucher SBB-0543 internal transcribed spacer 1, partial sequence; 5.8S ribosomal RNA gene and internal transcribed spacer 2, complete sequence; and 26S ribosomal RNA gene, partial sequence	1229	1229	100%	0.0	100%	
HM054580.1	Dendrobium bensoniae voucher SBB-0544 internal transcribed spacer 1, partial sequence; 5.8S ribosomal RNA gene and internal transcribed spacer 2, complete sequence; and 26S ribosomal RNA gene, partial sequence	1199	1199	97%	0.0	100%	
GU339116.1	Dendrobium crystallinum internal transcribed spacer 1, partial sequence; 5.8S ribosomal RNA gene, complete sequence; and internal transcribed spacer 2, partial sequence	1074	1074	95%	0.0	97%	
AF363023.1	Dendrobium crystallinum internal transcribed spacer 1, partial sequence; 5.8S ribosomal RNA gene, complete sequence; and internal transcribed spacer 2, partial sequence	944	944	95%	0.0	93%	
HM054538.1	Dendrobium amoenum voucher SBB-0140 internal transcribed spacer 1, partial sequence; 5.8S ribosomal RNA gene and internal transcribed spacer 2, complete sequence; and 26S ribosomal RNA gene, partial sequence	929	929	98%	0.0	92%	
HM054534.1	Dendrobium amoenum voucher SBB-0135 internal transcribed spacer 1, partial sequence; 5.8S ribosomal RNA gene and internal transcribed spacer 2, complete sequence; and 26S ribosomal RNA gene, partial sequence	929	929	98%	0.0	92%	
HM054536.1	Dendrobium amoenum voucher SBB-0138 internal transcribed spacer 1, partial sequence; 5.8S ribosomal RNA gene and internal transcribed spacer 2, complete sequence; and 26S ribosomal RNA gene, partial sequence	929	929	98%	0.0	92%	
HM054539.1	Dendrobium amoenum voucher SBB-0142 internal transcribed spacer 1, partial sequence; 5.8S ribosomal RNA gene and internal transcribed spacer 2, complete sequence; and 26S ribosomal RNA gene, partial sequence	928	928	98%	0.0	92%	
HM054540.1	Dendrobium amoenum voucher SBB-0247 internal transcribed spacer 1, partial sequence; 5.8S ribosomal RNA gene and internal transcribed spacer 2, complete sequence; and 26S ribosomal RNA gene, partial sequence	928	928	98%	0.0	92%	
HM054535.1	Dendrobium amoenum voucher SBB-0137 internal transcribed spacer 1, partial sequence; 5.8S ribosomal RNA gene and internal transcribed spacer 2, complete sequence; and 26S ribosomal RNA gene, partial sequence	928	928	98%	0.0	92%	
HM054543.1	Dendrobium amoenum voucher SBB-0029 internal transcribed spacer 1, partial sequence; 5.8S ribosomal RNA gene and internal transcribed spacer 2, complete sequence; and 26S ribosomal RNA gene, partial sequence	926	926	98%	0.0	92%	
HM054541.1	Dendrobium amoenum voucher SBB-0248 internal transcribed spacer 1, partial sequence; 5.8S ribosomal RNA gene and internal transcribed spacer 2, complete sequence; and 26S ribosomal RNA gene, partial sequence	924	924	98%	0.0	92%	
HM054542.1	Dendrobium amoenum voucher SBB-0249 internal transcribed spacer 1, partial sequence; 5.8S ribosomal RNA gene and internal transcribed spacer 2, complete sequence; and 26S ribosomal RNA gene, partial sequence	922	922	98%	0.0	92%	

NCBI Blast:D bicameratum - Mozilla Firefox

file:///G:/THESIS/NCBI DOWNLOADED MATK SEQUENCES/ITS/BLAST results 93+36 sequences/NCBI BlastD bicameratum_htm.htm

Legend for links to other resources: UniGene GEO Gene Structure Map Viewer PubChem BioAssay

Sequences producing significant alignments:

Accession	Description	Max score	Total score	Query coverage	E value	Max ident	Links
HM054589.1	Dendrobium bicameratum voucher SBB-0228 internal transcribed spacer 1, partial sequence; 5.8S ribosomal RNA gene and internal transcribed spacer 2, complete sequence; and 26S ribosomal RNA gene, partial sequence	1236	1236	100%	0.0	99%	
HM054581.1	Dendrobium bicameratum voucher SBB-0213 internal transcribed spacer 1, partial sequence; 5.8S ribosomal RNA gene and internal transcribed spacer 2, complete sequence; and 26S ribosomal RNA gene, partial sequence	1234	1234	100%	0.0	99%	
HM054591.1	Dendrobium bicameratum voucher SBB-0028 internal transcribed spacer 1, partial sequence; 5.8S ribosomal RNA gene and internal transcribed spacer 2, complete sequence; and 26S ribosomal RNA gene, partial sequence	1227	1227	98%	0.0	100%	
HM054586.1	Dendrobium bicameratum voucher SBB-0222 internal transcribed spacer 1, partial sequence; 5.8S ribosomal RNA gene and internal transcribed spacer 2, complete sequence; and 26S ribosomal RNA gene, partial sequence	1223	1223	98%	0.0	99%	
HM054587.1	Dendrobium bicameratum voucher SBB-0223 internal transcribed spacer 1, partial sequence; 5.8S ribosomal RNA gene and internal transcribed spacer 2, complete sequence; and 26S ribosomal RNA gene, partial sequence	1223	1223	98%	0.0	99%	
HM054585.1	Dendrobium bicameratum voucher SBB-0221 internal transcribed spacer 1, partial sequence; 5.8S ribosomal RNA gene and internal transcribed spacer 2, complete sequence; and 26S ribosomal RNA gene, partial sequence	1221	1221	98%	0.0	100%	
HM054583.1	Dendrobium bicameratum voucher SBB-0216 internal transcribed spacer 1, partial sequence; 5.8S ribosomal RNA gene and internal transcribed spacer 2, complete sequence; and 26S ribosomal RNA gene, partial sequence	1218	1218	98%	0.0	100%	
HM054584.1	Dendrobium bicameratum voucher SBB-0217 internal transcribed spacer 1, partial sequence; 5.8S ribosomal RNA gene and internal transcribed spacer 2, complete sequence; and 26S ribosomal RNA gene, partial sequence	1201	1201	96%	0.0	100%	
HM054590.1	Dendrobium bicameratum voucher SBB-0239 internal transcribed spacer 1, partial sequence; 5.8S ribosomal RNA gene and internal transcribed spacer 2, complete sequence; and 26S ribosomal RNA gene, partial sequence	1182	1182	95%	0.0	99%	
HM054588.1	Dendrobium bicameratum voucher SBB-0226 internal transcribed spacer 1, partial sequence; 5.8S ribosomal RNA gene and internal transcribed spacer 2, complete sequence; and 26S ribosomal RNA gene, partial sequence	1168	1168	94%	0.0	99%	
HM054582.1	Dendrobium bicameratum voucher SBB-0215 internal transcribed spacer 1, partial sequence; 5.8S ribosomal RNA gene, complete sequence; and internal transcribed spacer 2, partial sequence	1149	1149	92%	0.0	99%	
GU339104.1	Dendrobium stuposum internal transcribed spacer 1, partial sequence; 5.8S ribosomal RNA gene, complete sequence; and internal transcribed spacer 2, partial sequence	963	963	95%	0.0	93%	
HM590375.1	Dendrobium aurantiacum voucher NCHU-D89331201-1009 18S ribosomal RNA gene, partial sequence; internal transcribed spacer 1, 5.8S ribosomal RNA gene, and internal transcribed spacer 2, complete sequence; and 26S ribosomal RNA gene, partial sequence	946	946	100%	0.0	92%	
	Dendrobium fimbriatum voucher NCHU-D89331201-1029 18S ribosomal RNA gene, partial sequence; internal transcribed spacer 1, 5.8S [illegible]						

REVIEW | BLAST results 93+36... | Document2 - Micro... | NCBI Blast:D bicam... | BMC | Additional files | 17:36

NCBI Blast:D chrysanthum - Mozilla Firefox

file:///G:/THESIS/NCBI DOWNLOADED MATK SEQUENCES/ITS/BLAST results 93+36 sequences/NCBI BlastD chrysanthum_htm.htm

Legend for links to other resources: UniGene GEO Gene Structure Map Viewer PubChem BioAssay

Sequences producing significant alignments:

Accession	Description	Max score	Total score	Query coverage	E value	Max ident	Links
HM054598.1	Dendrobium chrysanthum voucher SBB-0621 internal transcribed spacer 1, partial sequence; 5.8S ribosomal RNA gene and internal transcribed spacer 2, complete sequence; and 26S ribosomal RNA gene, partial sequence	1218	1218	90%	0.0	100%	
HM054597.1	Dendrobium chrysanthum voucher SBB-0620 internal transcribed spacer 1, partial sequence; 5.8S ribosomal RNA gene and internal transcribed spacer 2, complete sequence; and 26S ribosomal RNA gene, partial sequence	1214	1214	90%	0.0	100%	
HM054599.1	Dendrobium chrysanthum voucher SBB-0622 internal transcribed spacer 1, partial sequence; 5.8S ribosomal RNA gene and internal transcribed spacer 2, complete sequence; and 26S ribosomal RNA gene, partial sequence	1214	1214	90%	0.0	100%	
HM590376.1	Dendrobium chrysanthum voucher NCHU-D89331201-1010 18S ribosomal RNA gene, partial sequence; internal transcribed spacer 1, 5.8S ribosomal RNA gene, and internal transcribed spacer 2, complete sequence; and 26S ribosomal RNA gene, partial sequence	1205	1205	99%	0.0	96%	
HM054596.1	Dendrobium chrysanthum voucher SBB-0619 internal transcribed spacer 1, partial sequence; 5.8S ribosomal RNA gene, complete sequence; and internal transcribed spacer 2, partial sequence	1166	1166	87%	0.0	100%	
HM054593.1	Dendrobium chrysanthum voucher SBB-0503 internal transcribed spacer 1, partial sequence; 5.8S ribosomal RNA gene and internal transcribed spacer 2, complete sequence; and 26S ribosomal RNA gene, partial sequence	1160	1160	86%	0.0	100%	
HM054594.1	Dendrobium chrysanthum voucher SBB-0563 internal transcribed spacer 1, partial sequence; 5.8S ribosomal RNA gene and internal transcribed spacer 2, complete sequence; and 26S ribosomal RNA gene, partial sequence	1157	1157	86%	0.0	100%	
AF355572.1	Dendrobium chrysanthum internal transcribed spacer 1, partial sequence; 5.8S ribosomal RNA gene, complete sequence; and internal transcribed spacer 2, partial sequence	1155	1155	88%	0.0	99%	
HM054595.1	Dendrobium chrysanthum voucher SBB-0618 internal transcribed spacer 1, partial sequence; 5.8S ribosomal RNA gene, complete sequence; and internal transcribed spacer 2, partial sequence	1147	1147	85%	0.0	100%	
HM054592.1	Dendrobium chrysanthum voucher SBB-0033 internal transcribed spacer 1, partial sequence; 5.8S ribosomal RNA gene and internal transcribed spacer 2, complete sequence; and 26S ribosomal RNA gene, partial sequence	1125	1125	84%	0.0	100%	
AF362047.1	Dendrobium chrysanthum internal transcribed spacer 1, partial sequence; 5.8S ribosomal RNA gene, complete sequence; and internal transcribed spacer 2, partial sequence >gb|FJ384738.1| Dendrobium chrysanthum internal transcribed spacer 1, partial sequence; 5.8S ribosomal RNA gene, complete sequence; and internal transcribed spacer 2, partial sequence	1083	1083	88%	0.0	97%	
EU003119.1	Dendrobium chrysanthum voucher CMU DC 0612 internal transcribed spacer 1, partial sequence; 5.8S ribosomal RNA gene, complete sequence; and internal transcribed spacer 2, partial sequence	1062	1062	88%	0.0	96%	
AY485713.1	Dendrobium chrysanthum specimen-voucher ICMdcsa2103-8 internal transcribed spacer 1, partial sequence; 5.8S ribosomal RNA gene, [illegible]	987	987	88%	0.0	94%	

REVIEW | BLAST results 93+36... | Document2 - Micro... | NCBI Blast:D chrysa... | BMC | Additional files | 17:36

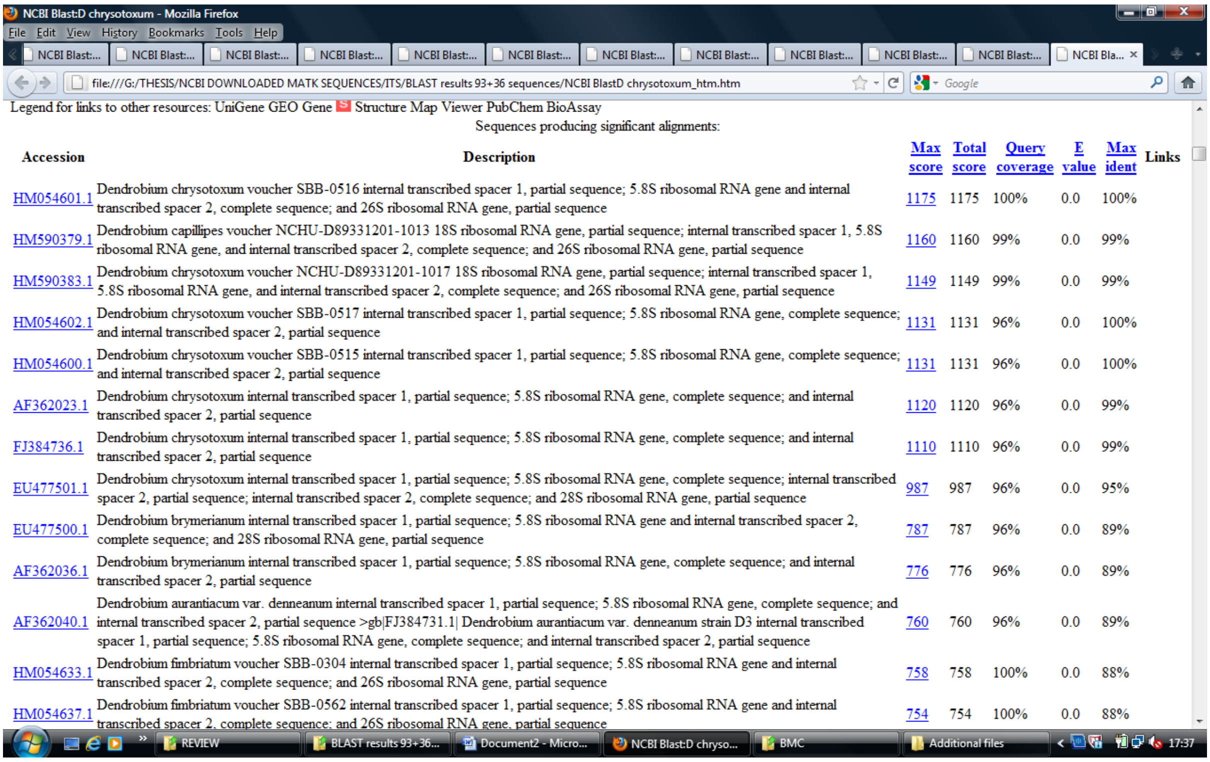

Legend for links to other resources: UniGene GEO Gene Structure Map Viewer PubChem BioAssay

Sequences producing significant alignments:

Accession	Description	Max score	Total score	Query coverage	E value	Max ident	Links
HM054601.1	Dendrobium chrysotoxum voucher SBB-0516 internal transcribed spacer 1, partial sequence; 5.8S ribosomal RNA gene and internal transcribed spacer 2, complete sequence; and 26S ribosomal RNA gene, partial sequence	1175	1175	100%	0.0	100%	
HM590379.1	Dendrobium capillipes voucher NCHU-D89331201-1013 18S ribosomal RNA gene, partial sequence; internal transcribed spacer 1, 5.8S ribosomal RNA gene, and internal transcribed spacer 2, complete sequence; and 26S ribosomal RNA gene, partial sequence	1160	1160	99%	0.0	99%	
HM590383.1	Dendrobium chrysotoxum voucher NCHU-D89331201-1017 18S ribosomal RNA gene, partial sequence; internal transcribed spacer 1, 5.8S ribosomal RNA gene, and internal transcribed spacer 2, complete sequence; and 26S ribosomal RNA gene, partial sequence	1149	1149	99%	0.0	99%	
HM054602.1	Dendrobium chrysotoxum voucher SBB-0517 internal transcribed spacer 1, partial sequence; 5.8S ribosomal RNA gene, complete sequence; and internal transcribed spacer 2, partial sequence	1131	1131	96%	0.0	100%	
HM054600.1	Dendrobium chrysotoxum voucher SBB-0515 internal transcribed spacer 1, partial sequence; 5.8S ribosomal RNA gene, complete sequence; and internal transcribed spacer 2, partial sequence	1131	1131	96%	0.0	100%	
AF362023.1	Dendrobium chrysotoxum internal transcribed spacer 1, partial sequence; 5.8S ribosomal RNA gene, complete sequence; and internal transcribed spacer 2, partial sequence	1120	1120	96%	0.0	99%	
FJ384736.1	Dendrobium chrysotoxum internal transcribed spacer 1, partial sequence; 5.8S ribosomal RNA gene, complete sequence; and internal transcribed spacer 2, partial sequence	1110	1110	96%	0.0	99%	
EU477501.1	Dendrobium chrysotoxum internal transcribed spacer 1, partial sequence; 5.8S ribosomal RNA gene, complete sequence; internal transcribed spacer 2, partial sequence; internal transcribed spacer 2, complete sequence; and 28S ribosomal RNA gene, partial sequence	987	987	96%	0.0	95%	
EU477500.1	Dendrobium brymerianum internal transcribed spacer 1, partial sequence; 5.8S ribosomal RNA gene and internal transcribed spacer 2, complete sequence; and 28S ribosomal RNA gene, partial sequence	787	787	96%	0.0	89%	
AF362036.1	Dendrobium brymerianum internal transcribed spacer 1, partial sequence; 5.8S ribosomal RNA gene, complete sequence; and internal transcribed spacer 2, partial sequence	776	776	96%	0.0	89%	
AF362040.1	Dendrobium aurantiacum var. denneanum internal transcribed spacer 1, partial sequence; 5.8S ribosomal RNA gene, complete sequence; and internal transcribed spacer 2, partial sequence >gb|FJ384731.1| Dendrobium aurantiacum var. denneanum strain D3 internal transcribed spacer 1, partial sequence; 5.8S ribosomal RNA gene, complete sequence; and internal transcribed spacer 2, partial sequence	760	760	96%	0.0	89%	
HM054633.1	Dendrobium fimbriatum voucher SBB-0304 internal transcribed spacer 1, partial sequence; 5.8S ribosomal RNA gene and internal transcribed spacer 2, complete sequence; and 26S ribosomal RNA gene, partial sequence	758	758	100%	0.0	88%	
HM054637.1	Dendrobium fimbriatum voucher SBB-0562 internal transcribed spacer 1, partial sequence; 5.8S ribosomal RNA gene and internal transcribed spacer 2, complete sequence; and 26S ribosomal RNA gene, partial sequence	754	754	100%	0.0	88%	

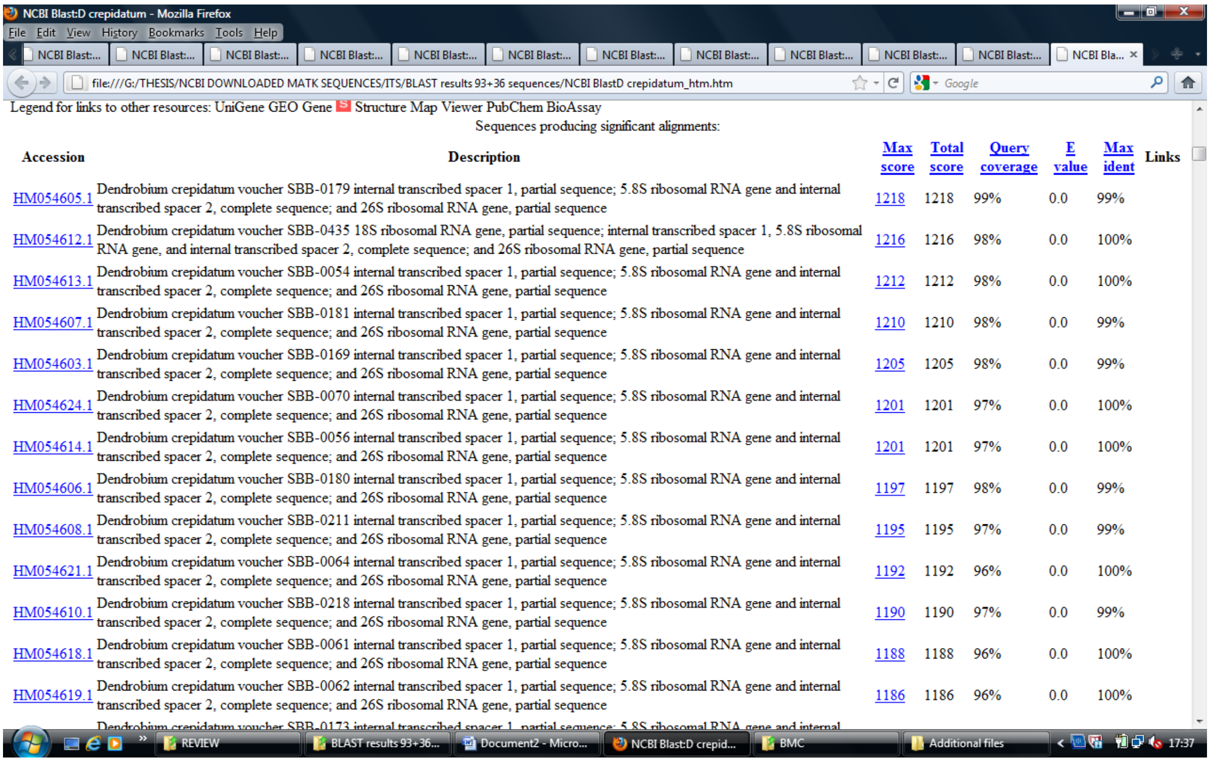

Legend for links to other resources: UniGene GEO Gene Structure Map Viewer PubChem BioAssay

Sequences producing significant alignments:

Accession	Description	Max score	Total score	Query coverage	E value	Max ident	Links
HM054605.1	Dendrobium crepidatum voucher SBB-0179 internal transcribed spacer 1, partial sequence; 5.8S ribosomal RNA gene and internal transcribed spacer 2, complete sequence; and 26S ribosomal RNA gene, partial sequence	1218	1218	99%	0.0	99%	
HM054612.1	Dendrobium crepidatum voucher SBB-0435 18S ribosomal RNA gene, partial sequence; internal transcribed spacer 1, 5.8S ribosomal RNA gene, and internal transcribed spacer 2, complete sequence; and 26S ribosomal RNA gene, partial sequence	1216	1216	98%	0.0	100%	
HM054613.1	Dendrobium crepidatum voucher SBB-0054 internal transcribed spacer 1, partial sequence; 5.8S ribosomal RNA gene and internal transcribed spacer 2, complete sequence; and 26S ribosomal RNA gene, partial sequence	1212	1212	98%	0.0	100%	
HM054607.1	Dendrobium crepidatum voucher SBB-0181 internal transcribed spacer 1, partial sequence; 5.8S ribosomal RNA gene and internal transcribed spacer 2, complete sequence; and 26S ribosomal RNA gene, partial sequence	1210	1210	98%	0.0	99%	
HM054603.1	Dendrobium crepidatum voucher SBB-0169 internal transcribed spacer 1, partial sequence; 5.8S ribosomal RNA gene and internal transcribed spacer 2, complete sequence; and 26S ribosomal RNA gene, partial sequence	1205	1205	98%	0.0	99%	
HM054624.1	Dendrobium crepidatum voucher SBB-0070 internal transcribed spacer 1, partial sequence; 5.8S ribosomal RNA gene and internal transcribed spacer 2, complete sequence; and 26S ribosomal RNA gene, partial sequence	1201	1201	97%	0.0	100%	
HM054614.1	Dendrobium crepidatum voucher SBB-0056 internal transcribed spacer 1, partial sequence; 5.8S ribosomal RNA gene and internal transcribed spacer 2, complete sequence; and 26S ribosomal RNA gene, partial sequence	1201	1201	97%	0.0	100%	
HM054606.1	Dendrobium crepidatum voucher SBB-0180 internal transcribed spacer 1, partial sequence; 5.8S ribosomal RNA gene and internal transcribed spacer 2, complete sequence; and 26S ribosomal RNA gene, partial sequence	1197	1197	98%	0.0	99%	
HM054608.1	Dendrobium crepidatum voucher SBB-0211 internal transcribed spacer 1, partial sequence; 5.8S ribosomal RNA gene and internal transcribed spacer 2, complete sequence; and 26S ribosomal RNA gene, partial sequence	1195	1195	97%	0.0	99%	
HM054621.1	Dendrobium crepidatum voucher SBB-0064 internal transcribed spacer 1, partial sequence; 5.8S ribosomal RNA gene and internal transcribed spacer 2, complete sequence; and 26S ribosomal RNA gene, partial sequence	1192	1192	96%	0.0	100%	
HM054610.1	Dendrobium crepidatum voucher SBB-0218 internal transcribed spacer 1, partial sequence; 5.8S ribosomal RNA gene and internal transcribed spacer 2, complete sequence; and 26S ribosomal RNA gene, partial sequence	1190	1190	97%	0.0	99%	
HM054618.1	Dendrobium crepidatum voucher SBB-0061 internal transcribed spacer 1, partial sequence; 5.8S ribosomal RNA gene and internal transcribed spacer 2, complete sequence; and 26S ribosomal RNA gene, partial sequence	1188	1188	96%	0.0	100%	
HM054619.1	Dendrobium crepidatum voucher SBB-0062 internal transcribed spacer 1, partial sequence; 5.8S ribosomal RNA gene and internal transcribed spacer 2, complete sequence; and 26S ribosomal RNA gene, partial sequence	1186	1186	96%	0.0	100%	
	Dendrobium crepidatum voucher SBB-0173 internal transcribed spacer 1, partial sequence; 5.8S ribosomal RNA gene and internal						

NCBI Blast:D crumenatum - Mozilla Firefox

file:///G:/THESIS/NCBI DOWNLOADED MATK SEQUENCES/ITS/BLAST results 93+36 sequences/NCBI BlastD crumenatum_htm.htm

Legend for links to other resources: UniGene GEO Gene S Structure Map Viewer PubChem BioAssay

Sequences producing significant alignments:

Accession	Description	Max score	Total score	Query coverage	E value	Max ident	Links
HM590370.1	Dendrobium crumenatum voucher NCHU-D89331201-1004 18S ribosomal RNA gene, partial sequence; internal transcribed spacer 1, 5.8S ribosomal RNA gene, and internal transcribed spacer 2, complete sequence; and 26S ribosomal RNA gene, partial sequence	1182	1182	100%	0.0	97%	
HM054625.1	Dendrobium crumenatum voucher SBB-0124 internal transcribed spacer 1, partial sequence; 5.8S ribosomal RNA gene and internal transcribed spacer 2, complete sequence; and 26S ribosomal RNA gene, partial sequence	1166	1166	92%	0.0	100%	
AY273708.1	Dendrobium crumenatum isolate 105 internal transcribed spacer 1, 5.8S ribosomal RNA gene, and internal transcribed spacer 2, complete sequence	1149	1149	100%	0.0	97%	
AY239963.1	Dendrobium crumenatum 18S ribosomal RNA gene, partial sequence; internal transcribed spacer 1, 5.8S ribosomal RNA gene and internal transcribed spacer 2, complete sequence; and 26S ribosomal RNA gene, partial sequence	1133	1133	97%	0.0	97%	
AF521608.1	Dendrobium crumenatum internal transcribed spacer 1, 5.8S ribosomal RNA gene, and internal transcribed spacer 2, complete sequence	1112	1112	93%	0.0	98%	
EU840700.1	Dendrobium crumenatum internal transcribed spacer 1, 5.8S ribosomal RNA gene, and internal transcribed spacer 2, complete sequence	1096	1096	93%	0.0	97%	
AY239961.1	Dendrobium confusum 18S ribosomal RNA gene, partial sequence; internal transcribed spacer 1, 5.8S ribosomal RNA gene and internal transcribed spacer 2, complete sequence; and 26S ribosomal RNA gene, partial sequence	911	911	97%	0.0	91%	
AY239972.1	Dendrobium indivisum 18S ribosomal RNA gene, partial sequence; internal transcribed spacer 1, 5.8S ribosomal RNA gene and internal transcribed spacer 2, complete sequence; and 26S ribosomal RNA gene, partial sequence	905	905	97%	0.0	91%	
AY239951.1	Dendrobium aloifolium 18S ribosomal RNA gene, partial sequence; internal transcribed spacer 1, 5.8S ribosomal RNA gene and internal transcribed spacer 2, complete sequence; and 26S ribosomal RNA gene, partial sequence	905	905	97%	0.0	91%	
AY239988.1	Dendrobium philippinense 18S ribosomal RNA gene, partial sequence; internal transcribed spacer 1, 5.8S ribosomal RNA gene and internal transcribed spacer 2, complete sequence; and 26S ribosomal RNA gene, partial sequence	887	887	97%	0.0	90%	
AY239953.1	Dendrobium anceps 18S ribosomal RNA gene, partial sequence; internal transcribed spacer 1, 5.8S ribosomal RNA gene and internal transcribed spacer 2, complete sequence; and 26S ribosomal RNA gene, partial sequence	883	883	97%	0.0	90%	
AY239969.1	Dendrobium goldfinchii 18S ribosomal RNA gene, partial sequence; internal transcribed spacer 1, 5.8S ribosomal RNA gene and internal transcribed spacer 2, complete sequence; and 26S ribosomal RNA gene, partial sequence	880	880	97%	0.0	90%	
HM054548.1	Dendrobium anceps voucher SBB-0301 18S ribosomal RNA gene, partial sequence; internal transcribed spacer 1, 5.8S ribosomal RNA gene, and internal transcribed spacer 2, complete sequence; and 26S ribosomal RNA gene, partial sequence	874	874	95%	0.0	90%	
AY239975.1	Dendrobium junceum 18S ribosomal RNA gene, partial sequence; internal transcribed spacer 1, 5.8S ribosomal RNA gene and internal transcribed spacer 2, complete sequence; and 26S ribosomal RNA gene, partial sequence	872	872	97%	0.0	90%	

REVIEW | BLAST results 93+36... | Document2 - Micro... | NCBI Blast:D crume... | BMC | Additional files | 17:37

NCBI Blast:D densiflorum - Mozilla Firefox

file:///G:/THESIS/NCBI DOWNLOADED MATK SEQUENCES/ITS/BLAST results 93+36 sequences/NCBI BlastD densiflorum_htm.htm

Legend for links to other resources: UniGene GEO Gene S Structure Map Viewer PubChem BioAssay

Sequences producing significant alignments:

Accession	Description	Max score	Total score	Query coverage	E value	Max ident	Links
HM054626.1	Dendrobium densiflorum voucher SBB-0120 internal transcribed spacer 1, partial sequence; 5.8S ribosomal RNA gene and internal transcribed spacer 2, complete sequence; and 26S ribosomal RNA gene, partial sequence	1234	1234	95%	0.0	100%	
HM054627.1	Dendrobium densiflorum voucher SBB-0547 internal transcribed spacer 1, partial sequence; 5.8S ribosomal RNA gene, complete sequence; and internal transcribed spacer 2, partial sequence	1131	1131	87%	0.0	100%	
HM054758.1	Dendrobium thyrsiflorum voucher SBB-0518 internal transcribed spacer 1, partial sequence; 5.8S ribosomal RNA gene and internal transcribed spacer 2, complete sequence; and 26S ribosomal RNA gene, partial sequence	1035	1035	94%	0.0	94%	
AY240001.1	Dendrobium thyrsiflorum 18S ribosomal RNA gene, partial sequence; internal transcribed spacer 1, 5.8S ribosomal RNA gene and internal transcribed spacer 2, complete sequence; and 26S ribosomal RNA gene, partial sequence	1035	1035	95%	0.0	94%	
FJ384733.1	Dendrobium thyrsiflorum internal transcribed spacer 1, partial sequence; 5.8S ribosomal RNA gene, complete sequence; and internal transcribed spacer 2, partial sequence	992	992	91%	0.0	94%	
EU840699.1	Epigeneium nakaharaei internal transcribed spacer 1, 5.8S ribosomal RNA gene, and internal transcribed spacer 2, complete sequence	992	992	91%	0.0	94%	
AF362029.1	Dendrobium densiflorum internal transcribed spacer 1, partial sequence; 5.8S ribosomal RNA gene, complete sequence; and internal transcribed spacer 2, partial sequence	992	992	91%	0.0	94%	
AF362032.1	Dendrobium thyrsiflorum internal transcribed spacer 1, partial sequence; 5.8S ribosomal RNA gene, complete sequence; and internal transcribed spacer 2, partial sequence	992	992	91%	0.0	94%	
DQ058786.1	Dendrobium densiflorum voucher MH-GZ03-1 internal transcribed spacer 1, 5.8S ribosomal RNA gene, and internal transcribed spacer 2, complete sequence	990	990	91%	0.0	94%	
HM054760.1	Dendrobium thyrsiflorum voucher SBB-0520 internal transcribed spacer 1, partial sequence; 5.8S ribosomal RNA gene and internal transcribed spacer 2, complete sequence; and 26S ribosomal RNA gene, partial sequence	985	985	91%	0.0	94%	
HM054759.1	Dendrobium thyrsiflorum voucher SBB-0519 internal transcribed spacer 1, partial sequence; 5.8S ribosomal RNA gene, complete sequence; and internal transcribed spacer 2, partial sequence	977	977	90%	0.0	94%	
HM054630.1	Dendrobium farmeri voucher SBB-0538 internal transcribed spacer 1, partial sequence; 5.8S ribosomal RNA gene and internal transcribed spacer 2, complete sequence; and 26S ribosomal RNA gene, partial sequence	885	885	92%	0.0	91%	
HM054631.1	Dendrobium farmeri voucher SBB-0549 internal transcribed spacer 1, partial sequence; 5.8S ribosomal RNA gene and internal transcribed spacer 2, complete sequence; and 26S ribosomal RNA gene, partial sequence	885	885	92%	0.0	91%	
HM054629.1	Dendrobium farmeri voucher SBB-0537 internal transcribed spacer 1, partial sequence; 5.8S ribosomal RNA gene, complete sequence; and [illegible]	857	857	90%	0.0	91%	

REVIEW | BLAST results 93+36... | Document2 - Micro... | NCBI Blast:D densifl... | BMC | Additional files | 17:38

NCBI Blast:D devonianum - Mozilla Firefox

file:///G:/THESIS/NCBI DOWNLOADED MATK SEQUENCES/ITS/BLAST results 93+36 sequences/NCBI BlastD devonianum_htm.htm

Legend for links to other resources: UniGene GEO Gene Structure Map Viewer PubChem BioAssay

Sequences producing significant alignments:

Accession	Description	Max score	Total score	Query coverage	E value	Max ident	Links
EU477502.1	Dendrobium devonianum internal transcribed spacer 1, partial sequence; 5.8S ribosomal RNA gene, complete sequence; internal transcribed spacer 2, partial sequence; internal transcribed spacer 2, complete sequence; and 28S ribosomal RNA gene, partial sequence	1118	1118	98%	0.0	98%	
FJ384735.1	Dendrobium devonianum internal transcribed spacer 1, partial sequence; 5.8S ribosomal RNA gene, complete sequence; and internal transcribed spacer 2, partial sequence	1118	1118	98%	0.0	98%	
AF311779.1	Dendrobium devonianum internal transcribed spacer 1, partial sequence; 5.8S ribosomal RNA gene, complete sequence; and internal transcribed spacer 2, partial sequence	1101	1101	98%	0.0	98%	
HM054570.1	Dendrobium aqueum voucher SBB-0367 18S ribosomal RNA gene, partial sequence; internal transcribed spacer 1, 5.8S ribosomal RNA gene, and internal transcribed spacer 2, complete sequence; and 26S ribosomal RNA gene, partial sequence	682	682	99%	0.0	86%	
HM054569.1	Dendrobium aqueum voucher SBB-0364 18S ribosomal RNA gene, partial sequence; internal transcribed spacer 1, 5.8S ribosomal RNA gene, and internal transcribed spacer 2, complete sequence; and 26S ribosomal RNA gene, partial sequence	682	682	99%	0.0	86%	
HM054566.1	Dendrobium aqueum voucher SBB-0361 internal transcribed spacer 1, partial sequence; 5.8S ribosomal RNA gene and internal transcribed spacer 2, complete sequence; and 26S ribosomal RNA gene, partial sequence	682	682	99%	0.0	86%	
HM054564.1	Dendrobium aqueum voucher SBB-0359 18S ribosomal RNA gene, partial sequence; internal transcribed spacer 1, 5.8S ribosomal RNA gene, and internal transcribed spacer 2, complete sequence; and 26S ribosomal RNA gene, partial sequence	682	682	99%	0.0	86%	
HM054562.1	Dendrobium aqueum voucher SBB-0303 18S ribosomal RNA gene, partial sequence; internal transcribed spacer 1, 5.8S ribosomal RNA gene, and internal transcribed spacer 2, complete sequence; and 26S ribosomal RNA gene, partial sequence	682	682	99%	0.0	86%	
AF362040.1	Dendrobium aurantiacum var. denneanum internal transcribed spacer 1, partial sequence; 5.8S ribosomal RNA gene, complete sequence; and internal transcribed spacer 2, partial sequence >gb|FJ384731.1| Dendrobium aurantiacum var. denneanum strain D3 internal transcribed spacer 1, partial sequence; 5.8S ribosomal RNA gene, complete sequence; and internal transcribed spacer 2, partial sequence	676	676	94%	0.0	87%	
HM054571.1	Dendrobium aqueum voucher SBB-0382 internal transcribed spacer 1, partial sequence; 5.8S ribosomal RNA gene and internal transcribed spacer 2, complete sequence; and 26S ribosomal RNA gene, partial sequence	669	669	95%	0.0	86%	
HM054568.1	Dendrobium aqueum voucher SBB-0363 internal transcribed spacer 1, partial sequence; 5.8S ribosomal RNA gene and internal transcribed spacer 2, complete sequence; and 26S ribosomal RNA gene, partial sequence	669	669	95%	0.0	86%	
HM054567.1	Dendrobium aqueum voucher SBB-0362 internal transcribed spacer 1, partial sequence; 5.8S ribosomal RNA gene and internal transcribed spacer 2, complete sequence; and 26S ribosomal RNA gene, partial sequence	669	669	95%	0.0	86%	
HM054565.1	Dendrobium aqueum voucher SBB-0360 internal transcribed spacer 1, partial sequence; 5.8S ribosomal RNA gene and internal transcribed spacer 2, complete sequence; and 26S ribosomal RNA gene, partial sequence	669	669	95%	0.0	86%	

NCBI Blast:D draconis - Mozilla Firefox

file:///G:/THESIS/NCBI DOWNLOADED MATK SEQUENCES/ITS/BLAST results 93+36 sequences/NCBI BlastD draconis_htm.htm

Legend for links to other resources: UniGene GEO Gene Structure Map Viewer PubChem BioAssay

Sequences producing significant alignments:

Accession	Description	Max score	Total score	Query coverage	E value	Max ident	Links
HM054628.1	Dendrobium draconis voucher SBB-0546 18S ribosomal RNA gene, partial sequence; internal transcribed spacer 1, 5.8S ribosomal RNA gene, and internal transcribed spacer 2, complete sequence; and 26S ribosomal RNA gene, partial sequence	1251	1251	100%	0.0	100%	
EU477503.1	Dendrobium draconis internal transcribed spacer 1, partial sequence; 5.8S ribosomal RNA gene and internal transcribed spacer 2, complete sequence; and 28S ribosomal RNA gene, partial sequence	1171	1171	94%	0.0	99%	
HM054669.1	Dendrobium infundibulum voucher SBB-0530 internal transcribed spacer 1, partial sequence; 5.8S ribosomal RNA gene and internal transcribed spacer 2, complete sequence; and 26S ribosomal RNA gene, partial sequence	1020	1020	94%	0.0	95%	
GU339106.1	Dendrobium christyanum internal transcribed spacer 1, partial sequence; 5.8S ribosomal RNA gene, complete sequence; and internal transcribed spacer 2, partial sequence	1016	1016	94%	0.0	95%	
HM054668.1	Dendrobium infundibulum voucher SBB-0529 internal transcribed spacer 1, partial sequence; 5.8S ribosomal RNA gene and internal transcribed spacer 2, complete sequence; and 26S ribosomal RNA gene, partial sequence	1013	1013	93%	0.0	95%	
EF629325.1	Dendrobium christyanum internal transcribed spacer 1, partial sequence; 5.8S ribosomal RNA gene, complete sequence; and internal transcribed spacer 2, partial sequence	1009	1009	94%	0.0	95%	
GU339112.1	Dendrobium longicornu internal transcribed spacer 1, partial sequence; 5.8S ribosomal RNA gene, complete sequence; and internal transcribed spacer 2, partial sequence	1005	1005	94%	0.0	95%	
DQ058796.1	Dendrobium longicornu voucher CJ-SC02-1 internal transcribed spacer 1, 5.8S ribosomal RNA gene, and internal transcribed spacer 2, complete sequence	1000	1000	94%	0.0	94%	
FJ428220.1	Dendrobium williamsonii internal transcribed spacer 1, partial sequence; 5.8S ribosomal RNA gene, complete sequence; and internal transcribed spacer 2, partial sequence	1000	1000	94%	0.0	94%	
AY239967.1	Dendrobium formosum 18S ribosomal RNA gene, partial sequence; internal transcribed spacer 1, 5.8S ribosomal RNA gene and internal transcribed spacer 2, complete sequence; and 26S ribosomal RNA gene, partial sequence	994	994	97%	0.0	93%	
EU592015.1	Dendrobium bellatulum internal transcribed spacer 1, partial sequence; 5.8S ribosomal RNA gene, complete sequence; and internal transcribed spacer 2, partial sequence	989	989	94%	0.0	94%	
AF362027.1	Dendrobium cariniferum internal transcribed spacer 1, partial sequence; 5.8S ribosomal RNA gene, complete sequence; and internal transcribed spacer 2, partial sequence	963	963	94%	0.0	93%	
AF362030.1	Dendrobium williamsonii internal transcribed spacer 1, partial sequence; 5.8S ribosomal RNA gene, complete sequence; and internal transcribed spacer 2, partial sequence	963	963	94%	0.0	93%	
[illegible]	[illegible]	[illegible]	[illegible]	[illegible]	[illegible]	[illegible]	

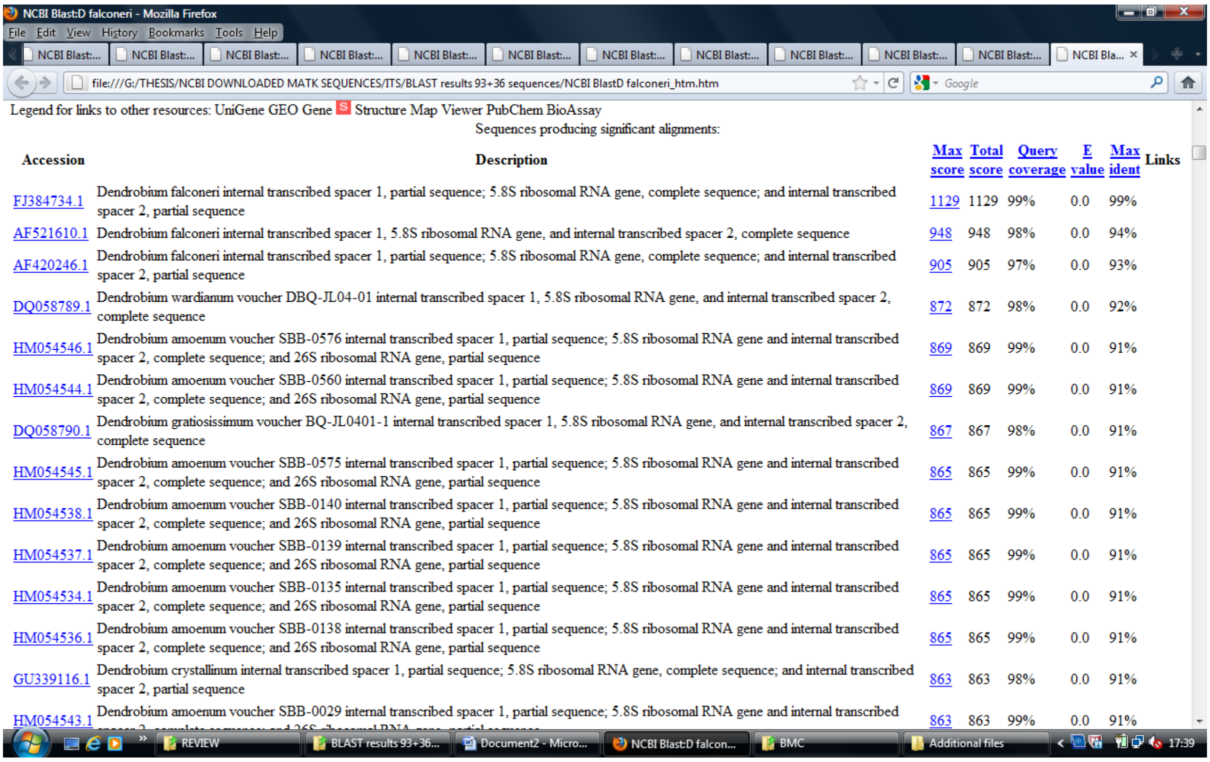

Accession	Description	Max score	Total score	Query coverage	E value	Max ident	Links
FJ384734.1	Dendrobium falconeri internal transcribed spacer 1, partial sequence; 5.8S ribosomal RNA gene, complete sequence; and internal transcribed spacer 2, partial sequence	1129	1129	99%	0.0	99%	
AF521610.1	Dendrobium falconeri internal transcribed spacer 1, 5.8S ribosomal RNA gene, and internal transcribed spacer 2, complete sequence	948	948	98%	0.0	94%	
AF420246.1	Dendrobium falconeri internal transcribed spacer 1, partial sequence; 5.8S ribosomal RNA gene, complete sequence; and internal transcribed spacer 2, partial sequence	905	905	97%	0.0	93%	
DQ058789.1	Dendrobium wardianum voucher DBQ-JL04-01 internal transcribed spacer 1, 5.8S ribosomal RNA gene, and internal transcribed spacer 2, complete sequence	872	872	98%	0.0	92%	
HM054546.1	Dendrobium amoenum voucher SBB-0576 internal transcribed spacer 1, partial sequence; 5.8S ribosomal RNA gene and internal transcribed spacer 2, complete sequence; and 26S ribosomal RNA gene, partial sequence	869	869	99%	0.0	91%	
HM054544.1	Dendrobium amoenum voucher SBB-0560 internal transcribed spacer 1, partial sequence; 5.8S ribosomal RNA gene and internal transcribed spacer 2, complete sequence; and 26S ribosomal RNA gene, partial sequence	869	869	99%	0.0	91%	
DQ058790.1	Dendrobium gratiosissimum voucher BQ-JL0401-1 internal transcribed spacer 1, 5.8S ribosomal RNA gene, and internal transcribed spacer 2, complete sequence	867	867	98%	0.0	91%	
HM054545.1	Dendrobium amoenum voucher SBB-0575 internal transcribed spacer 1, partial sequence; 5.8S ribosomal RNA gene and internal transcribed spacer 2, complete sequence; and 26S ribosomal RNA gene, partial sequence	865	865	99%	0.0	91%	
HM054538.1	Dendrobium amoenum voucher SBB-0140 internal transcribed spacer 1, partial sequence; 5.8S ribosomal RNA gene and internal transcribed spacer 2, complete sequence; and 26S ribosomal RNA gene, partial sequence	865	865	99%	0.0	91%	
HM054537.1	Dendrobium amoenum voucher SBB-0139 internal transcribed spacer 1, partial sequence; 5.8S ribosomal RNA gene and internal transcribed spacer 2, complete sequence; and 26S ribosomal RNA gene, partial sequence	865	865	99%	0.0	91%	
HM054534.1	Dendrobium amoenum voucher SBB-0135 internal transcribed spacer 1, partial sequence; 5.8S ribosomal RNA gene and internal transcribed spacer 2, complete sequence; and 26S ribosomal RNA gene, partial sequence	865	865	99%	0.0	91%	
HM054536.1	Dendrobium amoenum voucher SBB-0138 internal transcribed spacer 1, partial sequence; 5.8S ribosomal RNA gene and internal transcribed spacer 2, complete sequence; and 26S ribosomal RNA gene, partial sequence	865	865	99%	0.0	91%	
GU339116.1	Dendrobium crystallinum internal transcribed spacer 1, partial sequence; 5.8S ribosomal RNA gene, complete sequence; and internal transcribed spacer 2, partial sequence	863	863	98%	0.0	91%	
HM054543.1	Dendrobium amoenum voucher SBB-0029 internal transcribed spacer 1, partial sequence; 5.8S ribosomal RNA gene and internal transcribed [illegible]	863	863	99%	0.0	91%	

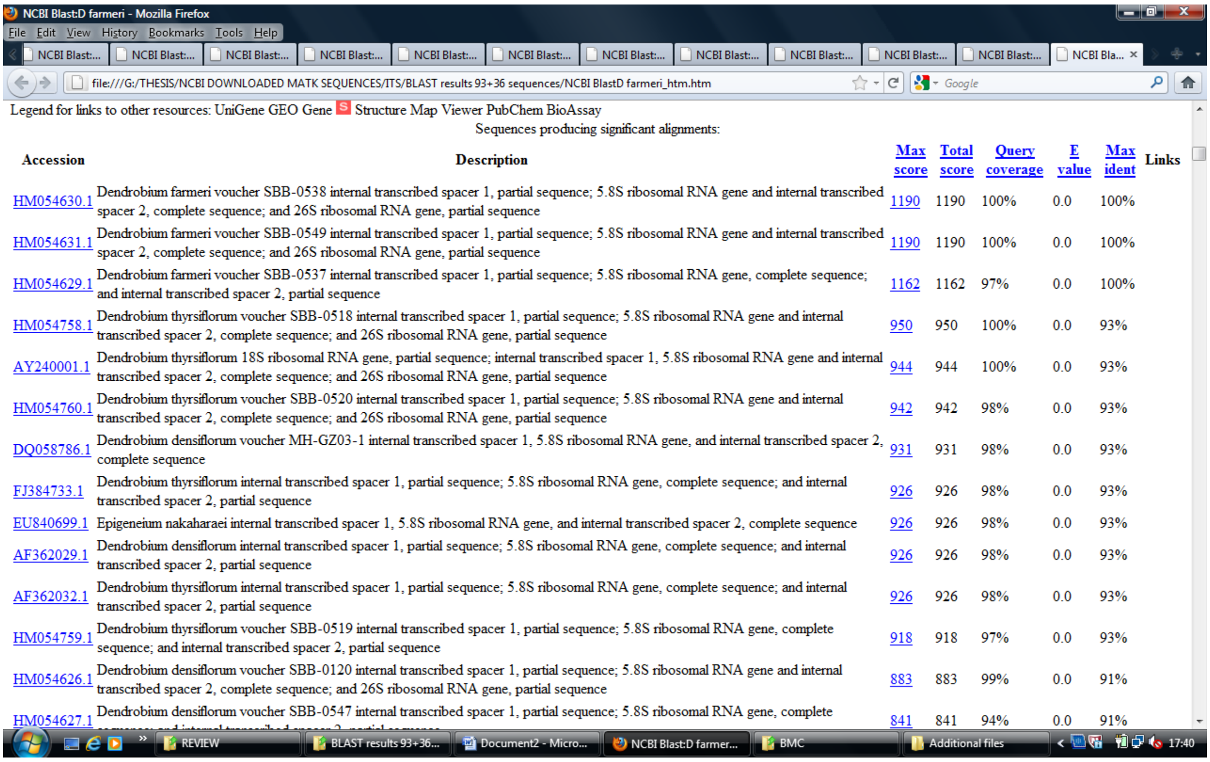

Accession	Description	Max score	Total score	Query coverage	E value	Max ident	Links
HM054630.1	Dendrobium farmeri voucher SBB-0538 internal transcribed spacer 1, partial sequence; 5.8S ribosomal RNA gene and internal transcribed spacer 2, complete sequence; and 26S ribosomal RNA gene, partial sequence	1190	1190	100%	0.0	100%	
HM054631.1	Dendrobium farmeri voucher SBB-0549 internal transcribed spacer 1, partial sequence; 5.8S ribosomal RNA gene and internal transcribed spacer 2, complete sequence; and 26S ribosomal RNA gene, partial sequence	1190	1190	100%	0.0	100%	
HM054629.1	Dendrobium farmeri voucher SBB-0537 internal transcribed spacer 1, partial sequence; 5.8S ribosomal RNA gene, complete sequence; and internal transcribed spacer 2, partial sequence	1162	1162	97%	0.0	100%	
HM054758.1	Dendrobium thyrsiflorum voucher SBB-0518 internal transcribed spacer 1, partial sequence; 5.8S ribosomal RNA gene and internal transcribed spacer 2, complete sequence; and 26S ribosomal RNA gene, partial sequence	950	950	100%	0.0	93%	
AY240001.1	Dendrobium thyrsiflorum 18S ribosomal RNA gene, partial sequence; internal transcribed spacer 1, 5.8S ribosomal RNA gene and internal transcribed spacer 2, complete sequence; and 26S ribosomal RNA gene, partial sequence	944	944	100%	0.0	93%	
HM054760.1	Dendrobium thyrsiflorum voucher SBB-0520 internal transcribed spacer 1, partial sequence; 5.8S ribosomal RNA gene and internal transcribed spacer 2, complete sequence; and 26S ribosomal RNA gene, partial sequence	942	942	98%	0.0	93%	
DQ058786.1	Dendrobium densiflorum voucher MH-GZ03-1 internal transcribed spacer 1, 5.8S ribosomal RNA gene, and internal transcribed spacer 2, complete sequence	931	931	98%	0.0	93%	
FJ384733.1	Dendrobium thyrsiflorum internal transcribed spacer 1, partial sequence; 5.8S ribosomal RNA gene, complete sequence; and internal transcribed spacer 2, partial sequence	926	926	98%	0.0	93%	
EU840699.1	Epigeneium nakaharaei internal transcribed spacer 1, 5.8S ribosomal RNA gene, and internal transcribed spacer 2, complete sequence	926	926	98%	0.0	93%	
AF362029.1	Dendrobium densiflorum internal transcribed spacer 1, partial sequence; 5.8S ribosomal RNA gene, complete sequence; and internal transcribed spacer 2, partial sequence	926	926	98%	0.0	93%	
AF362032.1	Dendrobium thyrsiflorum internal transcribed spacer 1, partial sequence; 5.8S ribosomal RNA gene, complete sequence; and internal transcribed spacer 2, partial sequence	926	926	98%	0.0	93%	
HM054759.1	Dendrobium thyrsiflorum voucher SBB-0519 internal transcribed spacer 1, partial sequence; 5.8S ribosomal RNA gene, complete sequence; and internal transcribed spacer 2, partial sequence	918	918	97%	0.0	93%	
HM054626.1	Dendrobium densiflorum voucher SBB-0120 internal transcribed spacer 1, partial sequence; 5.8S ribosomal RNA gene and internal transcribed spacer 2, complete sequence; and 26S ribosomal RNA gene, partial sequence	883	883	99%	0.0	91%	
HM054627.1	Dendrobium densiflorum voucher SBB-0547 internal transcribed spacer 1, partial sequence; 5.8S ribosomal RNA gene, complete [illegible]	841	841	94%	0.0	91%	

NCBI Blast:D fimbriatum - Mozilla Firefox

file:///G:/THESIS/NCBI DOWNLOADED MATK SEQUENCES/ITS/BLAST results 93+36 sequences/NCBI BlastD fimbriatum_htm.htm

Legend for links to other resources: UniGene GEO Gene S Structure Map Viewer PubChem BioAssay

Sequences producing significant alignments:

Accession	Description	Max score	Total score	Query coverage	E value	Max ident	Links
HM590392.1	Dendrobium fimbriatum voucher NCHU-D89331201-1029 18S ribosomal RNA gene, partial sequence; internal transcribed spacer 1, 5.8S ribosomal RNA gene, and internal transcribed spacer 2, complete sequence; and 26S ribosomal RNA gene, partial sequence	1219	1219	99%	0.0	99%	
HM054636.1	Dendrobium fimbriatum voucher SBB-0510 internal transcribed spacer 1, partial sequence; 5.8S ribosomal RNA gene and internal transcribed spacer 2, complete sequence; and 26S ribosomal RNA gene, partial sequence	1218	1218	98%	0.0	100%	
HM054637.1	Dendrobium fimbriatum voucher SBB-0562 internal transcribed spacer 1, partial sequence; 5.8S ribosomal RNA gene and internal transcribed spacer 2, complete sequence; and 26S ribosomal RNA gene, partial sequence	1205	1205	97%	0.0	100%	
HM590375.1	Dendrobium aurantiacum voucher NCHU-D89331201-1009 18S ribosomal RNA gene, partial sequence; internal transcribed spacer 1, 5.8S ribosomal RNA gene, and internal transcribed spacer 2, complete sequence; and 26S ribosomal RNA gene, partial sequence	1170	1170	99%	0.0	98%	
HM054633.1	Dendrobium fimbriatum voucher SBB-0304 internal transcribed spacer 1, partial sequence; 5.8S ribosomal RNA gene and internal transcribed spacer 2, complete sequence; and 26S ribosomal RNA gene, partial sequence	1168	1168	97%	0.0	98%	
HM054635.1	Dendrobium fimbriatum voucher SBB-0509 internal transcribed spacer 1, partial sequence; 5.8S ribosomal RNA gene and internal transcribed spacer 2, complete sequence; and 26S ribosomal RNA gene, partial sequence	1146	1146	92%	0.0	100%	
HM054632.1	Dendrobium fimbriatum voucher SBB-0115 internal transcribed spacer 1, partial sequence; 5.8S ribosomal RNA gene and internal transcribed spacer 2, complete sequence; and 26S ribosomal RNA gene, partial sequence	1136	1136	95%	0.0	98%	
EU003116.1	Dendrobium fimbriatum voucher CMU DF 0611 internal transcribed spacer 1, partial sequence; 5.8S ribosomal RNA gene, complete sequence; and internal transcribed spacer 2, partial sequence	1129	1129	94%	0.0	98%	
HM054634.1	Dendrobium fimbriatum voucher SBB-0508 internal transcribed spacer 1, partial sequence; 5.8S ribosomal RNA gene, complete sequence; and internal transcribed spacer 2, partial sequence	1116	1116	90%	0.0	100%	
AF362042.1	Dendrobium aurantiacum isolate Guangxi internal transcribed spacer 1, partial sequence; 5.8S ribosomal RNA gene, complete sequence; and internal transcribed spacer 2, partial sequence	1105	1105	94%	0.0	98%	
AF362044.1	Dendrobium aurantiacum isolate Kunming Yunnan internal transcribed spacer 1, partial sequence; 5.8S ribosomal RNA gene, complete sequence; and internal transcribed spacer 2, partial sequence	1099	1099	94%	0.0	97%	
AY842036.1	Dendrobium fimbriatum internal transcribed spacer 1, partial sequence; 5.8S ribosomal RNA gene, complete sequence; and internal transcribed spacer 2, partial sequence	1051	1051	94%	0.0	96%	
HM054581.1	Dendrobium bicameratum voucher SBB-0213 internal transcribed spacer 1, partial sequence; 5.8S ribosomal RNA gene and internal transcribed spacer 2, complete sequence; and 26S ribosomal RNA gene, partial sequence	944	944	99%	0.0	92%	

REVIEW | BLAST results 93+36... | Document2 - Micro... | NCBI Blast:D fimbri... | BMC | Additional files | 17:41

NCBI Blast:D haemoglossum - Mozilla Firefox

file:///G:/THESIS/NCBI DOWNLOADED MATK SEQUENCES/ITS/BLAST results 93+36 sequences/NCBI BlastD haemoglossum_htm.htm

Legend for links to other resources: UniGene GEO Gene S Structure Map Viewer PubChem BioAssay

Sequences producing significant alignments:

Accession	Description	Max score	Total score	Query coverage	E value	Max ident	Links
HM054639.1	Dendrobium haemoglossum voucher SBB-0289 internal transcribed spacer 1, partial sequence; 5.8S ribosomal RNA gene and internal transcribed spacer 2, complete sequence; and 26S ribosomal RNA gene, partial sequence	1253	1253	100%	0.0	100%	
HM054638.1	Dendrobium haemoglossum voucher SBB-0031 internal transcribed spacer 1, partial sequence; 5.8S ribosomal RNA gene and internal transcribed spacer 2, complete sequence; and 26S ribosomal RNA gene, partial sequence	1195	1195	95%	0.0	99%	
EU430384.1	Dendrobium kingianum subsp. carnarvonense 18S ribosomal RNA gene, partial sequence; internal transcribed spacer 1, 5.8S ribosomal RNA gene, and internal transcribed spacer 2, complete sequence; and 26S ribosomal RNA gene, partial sequence	998	998	100%	0.0	93%	
EU430383.1	Dendrobium jonesii var. magnificum 18S ribosomal RNA gene, partial sequence; internal transcribed spacer 1, 5.8S ribosomal RNA gene, and internal transcribed spacer 2, complete sequence; and 26S ribosomal RNA gene, partial sequence	983	983	100%	0.0	92%	
EU430395.1	Dendrobium speciosum var. curvicaule 18S ribosomal RNA gene, partial sequence; internal transcribed spacer 1, 5.8S ribosomal RNA gene, and internal transcribed spacer 2, complete sequence; and 26S ribosomal RNA gene, partial sequence	977	977	100%	0.0	92%	
EU430398.1	Dendrobium speciosum var. pedunculatum 18S ribosomal RNA gene, partial sequence; internal transcribed spacer 1, 5.8S ribosomal RNA gene, and internal transcribed spacer 2, complete sequence; and 26S ribosomal RNA gene, partial sequence	976	976	100%	0.0	92%	
EU430385.1	Dendrobium kingianum var. pulcherrimum 18S ribosomal RNA gene, partial sequence; internal transcribed spacer 1, 5.8S ribosomal RNA gene, and internal transcribed spacer 2, complete sequence; and 26S ribosomal RNA gene, partial sequence	968	968	100%	0.0	92%	
EU430382.1	Dendrobium gracilicaule 18S ribosomal RNA gene, partial sequence; internal transcribed spacer 1, 5.8S ribosomal RNA gene, and internal transcribed spacer 2, complete sequence; and 26S ribosomal RNA gene, partial sequence	966	966	100%	0.0	92%	
EU430380.1	Dendrobium fleckeri 18S ribosomal RNA gene, partial sequence; internal transcribed spacer 1, 5.8S ribosomal RNA gene, and internal transcribed spacer 2, complete sequence; and 26S ribosomal RNA gene, partial sequence	963	963	97%	0.0	92%	
EU430371.1	Dendrobium adae internal transcribed spacer 1, partial sequence; 5.8S ribosomal RNA gene and internal transcribed spacer 2, complete sequence; and 26S ribosomal RNA gene, partial sequence	963	963	97%	0.0	92%	
EU430373.1	Dendrobium bifalce 18S ribosomal RNA gene, partial sequence; and internal transcribed spacer 1, 5.8S ribosomal RNA gene, internal transcribed spacer 2, and 26S ribosomal RNA gene, complete sequence	959	959	99%	0.0	92%	
AY240016.1	Grastidium baileyi 18S ribosomal RNA gene, partial sequence; internal transcribed spacer 1, 5.8S ribosomal RNA gene and internal transcribed spacer 2, complete sequence; and 26S ribosomal RNA gene, partial sequence	955	955	97%	0.0	92%	
HM590380.1	Dendrobium somai voucher NCHU-D89331201-1014 18S ribosomal RNA gene, partial sequence; internal transcribed spacer 1, 5.8S ribosomal RNA gene, and internal transcribed spacer 2, complete sequence; and 26S ribosomal RNA gene, partial sequence	952	952	100%	0.0	91%	

REVIEW | BLAST results 93+36... | Document2 - Micro... | NCBI Blast:D haemo... | BMC | Additional files | 17:41

NCBI Blast:D herbaceum - Mozilla Firefox

file:///G:/THESIS/NCBI DOWNLOADED MATK SEQUENCES/ITS/BLAST results 93+36 sequences/NCBI BlastD herbaceum_htm.htm

Legend for links to other resources: UniGene GEO Gene S Structure Map Viewer PubChem BioAssay

Sequences producing significant alignments:

Accession	Description	Max score	Total score	Query coverage	E value	Max ident	Links
HM054643.1	Dendrobium herbaceum voucher SBB-0110 18S ribosomal RNA gene, partial sequence; internal transcribed spacer 1, 5.8S ribosomal RNA gene, and internal transcribed spacer 2, complete sequence; and 26S ribosomal RNA gene, partial sequence	1190	1190	100%	0.0	100%	
HM054646.1	Dendrobium herbaceum voucher SBB-0036 18S ribosomal RNA gene, partial sequence; internal transcribed spacer 1, 5.8S ribosomal RNA gene, and internal transcribed spacer 2, complete sequence; and 26S ribosomal RNA gene, partial sequence >gb|HM054657.1| Dendrobium herbaceum voucher SBB-0047 18S ribosomal RNA gene, partial sequence; internal transcribed spacer 1, 5.8S ribosomal RNA gene, and internal transcribed spacer 2, complete sequence; and 26S ribosomal RNA gene, partial sequence >gb|HM054662.1| Dendrobium herbaceum voucher SBB-0052 18S ribosomal RNA gene, partial sequence; internal transcribed spacer 1, 5.8S ribosomal RNA gene, and internal transcribed spacer 2, complete sequence; and 26S ribosomal RNA gene, partial sequence >gb|HM054663.1| Dendrobium herbaceum voucher SBB-0053 18S ribosomal RNA gene, partial sequence; internal transcribed spacer 1, 5.8S ribosomal RNA gene, and internal transcribed spacer 2, complete sequence; and 26S ribosomal RNA gene, partial sequence	1190	1190	100%	0.0	100%	
HM054660.1	Dendrobium herbaceum voucher SBB-0050 18S ribosomal RNA gene, partial sequence; internal transcribed spacer 1, 5.8S ribosomal RNA gene, and internal transcribed spacer 2, complete sequence; and 26S ribosomal RNA gene, partial sequence	1186	1186	100%	0.0	99%	
HM054641.1	Dendrobium herbaceum voucher SBB-0108 internal transcribed spacer 1, 5.8S ribosomal RNA gene, and internal transcribed spacer 2, complete sequence; and 26S ribosomal RNA gene, partial sequence	1186	1186	99%	0.0	100%	
HM054658.1	Dendrobium herbaceum voucher SBB-0048 internal transcribed spacer 1, 5.8S ribosomal RNA gene, and internal transcribed spacer 2, complete sequence; and 26S ribosomal RNA gene, partial sequence >gb|HM054661.1| Dendrobium herbaceum voucher SBB-0051 internal transcribed spacer 1, 5.8S ribosomal RNA gene, and internal transcribed spacer 2, complete sequence; and 26S ribosomal RNA gene, partial sequence	1186	1186	99%	0.0	100%	
HM054644.1	Dendrobium herbaceum voucher SBB-0112 internal transcribed spacer 1, partial sequence; 5.8S ribosomal RNA gene and internal transcribed spacer 2, complete sequence; and 26S ribosomal RNA gene, partial sequence	1184	1184	99%	0.0	100%	
HM054653.1	Dendrobium herbaceum voucher SBB-0043 internal transcribed spacer 1, 5.8S ribosomal RNA gene, and internal transcribed spacer 2, complete sequence; and 26S ribosomal RNA gene, partial sequence	1182	1182	99%	0.0	99%	
HM054652.1	Dendrobium herbaceum voucher SBB-0042 internal transcribed spacer 1, partial sequence; 5.8S ribosomal RNA gene and internal transcribed spacer 2, complete sequence; and 26S ribosomal RNA gene, partial sequence	1181	1181	99%	0.0	100%	
HM054650.1	Dendrobium herbaceum voucher SBB-0040 18S ribosomal RNA gene, partial sequence; internal transcribed spacer 1, 5.8S ribosomal RNA gene, and internal transcribed spacer 2, complete sequence; and 26S ribosomal RNA gene, partial sequence	1181	1181	99%	0.0	99%	
HM054648.1	Dendrobium herbaceum voucher SBB-0038 internal transcribed spacer 1, partial sequence; 5.8S ribosomal RNA gene and internal	1177	1177	99%	0.0	99%	

NCBI Blast:D heterocarpum - Mozilla Firefox

file:///G:/THESIS/NCBI DOWNLOADED MATK SEQUENCES/ITS/BLAST results 93+36 sequences/NCBI BlastD heterocarpum_htm.htm

Legend for links to other resources: UniGene GEO Gene S Structure Map Viewer PubChem BioAssay

Sequences producing significant alignments:

Accession	Description	Max score	Total score	Query coverage	E value	Max ident	Links
HM054667.1	Dendrobium heterocarpum voucher SBB-0502 internal transcribed spacer 1, partial sequence; 5.8S ribosomal RNA gene and internal transcribed spacer 2, complete sequence; and 26S ribosomal RNA gene, partial sequence	1192	1192	99%	0.0	100%	
GU339101.1	Dendrobium heterocarpum internal transcribed spacer 1, partial sequence; 5.8S ribosomal RNA gene, complete sequence; and internal transcribed spacer 2, partial sequence	1175	1175	98%	0.0	100%	
HM590382.1	Dendrobium nobile voucher NCHU-D89331201-1016 18S ribosomal RNA gene, partial sequence; internal transcribed spacer 1, 5.8S ribosomal RNA gene, and internal transcribed spacer 2, complete sequence; and 26S ribosomal RNA gene, partial sequence	1014	1014	100%	0.0	95%	
GU339111.1	Dendrobium moniliforme internal transcribed spacer 1, partial sequence; 5.8S ribosomal RNA gene, complete sequence; and internal transcribed spacer 2, partial sequence	1009	1009	98%	0.0	95%	
HM590371.1	Dendrobium linawianum voucher NCHU-D89331201-1005 18S ribosomal RNA gene, partial sequence; internal transcribed spacer 1, 5.8S ribosomal RNA gene, and internal transcribed spacer 2, complete sequence; and 26S ribosomal RNA gene, partial sequence	1003	1003	100%	0.0	94%	
AF362039.1	Dendrobium nobile isolate Guangxi internal transcribed spacer 1, partial sequence; 5.8S ribosomal RNA gene, complete sequence; and internal transcribed spacer 2, partial sequence	1002	1002	98%	0.0	95%	
AF362045.1	Dendrobium nobile isolate Guizhou internal transcribed spacer 1, partial sequence; 5.8S ribosomal RNA gene, complete sequence; and internal transcribed spacer 2, partial sequence >gb|AF362046.1| Dendrobium nobile isolate Sichuan internal transcribed spacer 1, partial sequence; 5.8S ribosomal RNA gene, complete sequence; and internal transcribed spacer 2, partial sequence >gb|FJ384727.1| Dendrobium nobile strain J1 internal transcribed spacer 1, partial sequence; 5.8S ribosomal RNA gene, complete sequence; and internal transcribed spacer 2, partial sequence >gb|FJ378649.1| Dendrobium nobile strain J3 internal transcribed spacer 1, partial sequence; 5.8S ribosomal RNA gene, complete sequence; and internal transcribed spacer 2, partial sequence >gb|FJ530948.1| Dendrobium nobile strain J3 internal transcribed spacer 1, partial sequence; 5.8S ribosomal RNA gene, complete sequence; and internal transcribed spacer 2, partial sequence >gb|FJ804130.1| Dendrobium nobile isolate 5-2 internal transcribed spacer 1, partial sequence; 5.8S ribosomal RNA gene, complete sequence; and internal transcribed spacer 2, partial sequence >gb|FJ804131.1| Dendrobium nobile isolate 7-2 internal transcribed spacer 1, partial sequence; 5.8S ribosomal RNA gene, complete sequence; and internal transcribed spacer 2, partial sequence >gb|FJ804132.1| Dendrobium nobile isolate 10-7 internal transcribed spacer 1, partial sequence; 5.8S ribosomal RNA gene, complete sequence; and internal transcribed spacer 2, partial sequence >gb|FJ804133.1| Dendrobium nobile isolate 11-5 internal transcribed spacer 1, partial sequence; 5.8S ribosomal RNA gene, complete sequence; and internal transcribed spacer 2, partial sequence >gb|FJ804139.1| Dendrobium nobile isolate 24-2 internal transcribed spacer 1, partial sequence; 5.8S ribosomal RNA gene, complete sequence; and internal transcribed spacer 2, partial sequence >gb|FJ804141.1| Dendrobium nobile isolate 26-1 internal transcribed spacer 1, partial sequence; 5.8S ribosomal RNA gene, complete sequence; and internal transcribed spacer 2, partial sequence >gb|FJ804142.1| Dendrobium nobile isolate 27-6 internal transcribed spacer 1, partial sequence; 5.8S ribosomal RNA gene, complete sequence;	1002	1002	98%	0.0	95%	

NCBI Blast:D infundibulum - Mozilla Firefox

Legend for links to other resources: UniGene GEO Gene Structure Map Viewer PubChem BioAssay

Sequences producing significant alignments:

Accession	Description	Max score	Total score	Query coverage	E value	Max ident	Links
HM054669.1	Dendrobium infundibulum voucher SBB-0530 internal transcribed spacer 1, partial sequence; 5.8S ribosomal RNA gene and internal transcribed spacer 2, complete sequence; and 26S ribosomal RNA gene, partial sequence	1182	1182	96%	0.0	100%	
HM054668.1	Dendrobium infundibulum voucher SBB-0529 internal transcribed spacer 1, partial sequence; 5.8S ribosomal RNA gene and internal transcribed spacer 2, complete sequence; and 26S ribosomal RNA gene, partial sequence	1171	1171	95%	0.0	99%	
GU339106.1	Dendrobium christyanum internal transcribed spacer 1, partial sequence; 5.8S ribosomal RNA gene, complete sequence; and internal transcribed spacer 2, partial sequence	1099	1099	94%	0.0	98%	
EF629325.1	Dendrobium christyanum internal transcribed spacer 1, partial sequence; 5.8S ribosomal RNA gene, complete sequence; and internal transcribed spacer 2, partial sequence	1099	1099	94%	0.0	98%	
EU592015.1	Dendrobium bellatulum internal transcribed spacer 1, partial sequence; 5.8S ribosomal RNA gene, complete sequence; and internal transcribed spacer 2, partial sequence	1072	1072	94%	0.0	97%	
HM054628.1	Dendrobium draconis voucher SBB-0546 18S ribosomal RNA gene, partial sequence; internal transcribed spacer 1, 5.8S ribosomal RNA gene, and internal transcribed spacer 2, complete sequence; and 26S ribosomal RNA gene, partial sequence	1059	1059	100%	0.0	95%	
FJ428220.1	Dendrobium williamsonii internal transcribed spacer 1, partial sequence; 5.8S ribosomal RNA gene, complete sequence; and internal transcribed spacer 2, partial sequence	1055	1055	94%	0.0	96%	
GU339112.1	Dendrobium longicornu internal transcribed spacer 1, partial sequence; 5.8S ribosomal RNA gene, complete sequence; and internal transcribed spacer 2, partial sequence	1050	1050	94%	0.0	96%	
AY239967.1	Dendrobium formosum 18S ribosomal RNA gene, partial sequence; internal transcribed spacer 1, 5.8S ribosomal RNA gene and internal transcribed spacer 2, complete sequence; and 26S ribosomal RNA gene, partial sequence	1046	1046	97%	0.0	95%	
DQ058796.1	Dendrobium longicornu voucher CJ-SC02-1 internal transcribed spacer 1, 5.8S ribosomal RNA gene, and internal transcribed spacer 2, complete sequence	1044	1044	94%	0.0	96%	
AF362027.1	Dendrobium cariniferum internal transcribed spacer 1, partial sequence; 5.8S ribosomal RNA gene, complete sequence; and internal transcribed spacer 2, partial sequence	1029	1029	94%	0.0	96%	
AF362030.1	Dendrobium williamsonii internal transcribed spacer 1, partial sequence; 5.8S ribosomal RNA gene, complete sequence; and internal transcribed spacer 2, partial sequence	1029	1029	94%	0.0	96%	
EU477503.1	Dendrobium draconis internal transcribed spacer 1, partial sequence; 5.8S ribosomal RNA gene and internal transcribed spacer 2, complete sequence; and 28S ribosomal RNA gene, partial sequence	1005	1005	94%	0.0	95%	
[illegible]	[illegible]	[illegible]	[illegible]	[illegible]	[illegible]	[illegible]	

NCBI Blast:D jenkinsii - Mozilla Firefox

Legend for links to other resources: UniGene GEO Gene Structure Map Viewer PubChem BioAssay

Sequences producing significant alignments:

Accession	Description	Max score	Total score	Query coverage	E value	Max ident	Links
HM054671.1	Dendrobium jenkinsii voucher SBB-0526 internal transcribed spacer 1, partial sequence; 5.8S ribosomal RNA gene and internal transcribed spacer 2, complete sequence; and 26S ribosomal RNA gene, partial sequence	1236	1236	99%	0.0	100%	
HM054670.1	Dendrobium jenkinsii voucher SBB-0524 18S ribosomal RNA gene, partial sequence; internal transcribed spacer 1, 5.8S ribosomal RNA gene, and internal transcribed spacer 2, complete sequence; and 26S ribosomal RNA gene, partial sequence	1216	1216	97%	0.0	100%	
DQ058785.1	Dendrobium jenkinsii voucher XHH-SC02-1 internal transcribed spacer 1, 5.8S ribosomal RNA gene, and internal transcribed spacer 2, complete sequence	1160	1160	95%	0.0	99%	
DQ058784.1	Dendrobium lindleyi voucher JU-ML03-1 internal transcribed spacer 1, 5.8S ribosomal RNA gene, and internal transcribed spacer 2, complete sequence	944	944	95%	0.0	93%	
HM054673.1	Dendrobium lindleyi voucher SBB-0523 internal transcribed spacer 1, partial sequence; 5.8S ribosomal RNA gene and internal transcribed spacer 2, complete sequence; and 26S ribosomal RNA gene, partial sequence	937	937	98%	0.0	92%	
GU339114.1	Dendrobium lindleyi internal transcribed spacer 1, partial sequence; 5.8S ribosomal RNA gene, complete sequence; and internal transcribed spacer 2, partial sequence	929	929	95%	0.0	92%	
HM054672.1	Dendrobium lindleyi voucher SBB-0521 internal transcribed spacer 1, partial sequence; 5.8S ribosomal RNA gene and internal transcribed spacer 2, complete sequence; and 26S ribosomal RNA gene, partial sequence	896	896	94%	0.0	92%	
GU339106.1	Dendrobium christyanum internal transcribed spacer 1, partial sequence; 5.8S ribosomal RNA gene, complete sequence; and internal transcribed spacer 2, partial sequence	758	758	95%	0.0	88%	
EF629325.1	Dendrobium christyanum internal transcribed spacer 1, partial sequence; 5.8S ribosomal RNA gene, complete sequence; and internal transcribed spacer 2, partial sequence	752	752	95%	0.0	88%	
AY239967.1	Dendrobium formosum 18S ribosomal RNA gene, partial sequence; internal transcribed spacer 1, 5.8S ribosomal RNA gene and internal transcribed spacer 2, complete sequence; and 26S ribosomal RNA gene, partial sequence	745	745	98%	0.0	87%	
HM054669.1	Dendrobium infundibulum voucher SBB-0530 internal transcribed spacer 1, partial sequence; 5.8S ribosomal RNA gene and internal transcribed spacer 2, complete sequence; and 26S ribosomal RNA gene, partial sequence	741	741	93%	0.0	87%	
HM054668.1	Dendrobium infundibulum voucher SBB-0529 internal transcribed spacer 1, partial sequence; 5.8S ribosomal RNA gene and internal transcribed spacer 2, complete sequence; and 26S ribosomal RNA gene, partial sequence	741	741	94%	0.0	87%	
EU592015.1	Dendrobium bellatulum internal transcribed spacer 1, partial sequence; 5.8S ribosomal RNA gene, complete sequence; and internal transcribed spacer 2, partial sequence	741	741	95%	0.0	87%	
[illegible]	[illegible]	[illegible]	[illegible]	[illegible]	[illegible]	[illegible]	

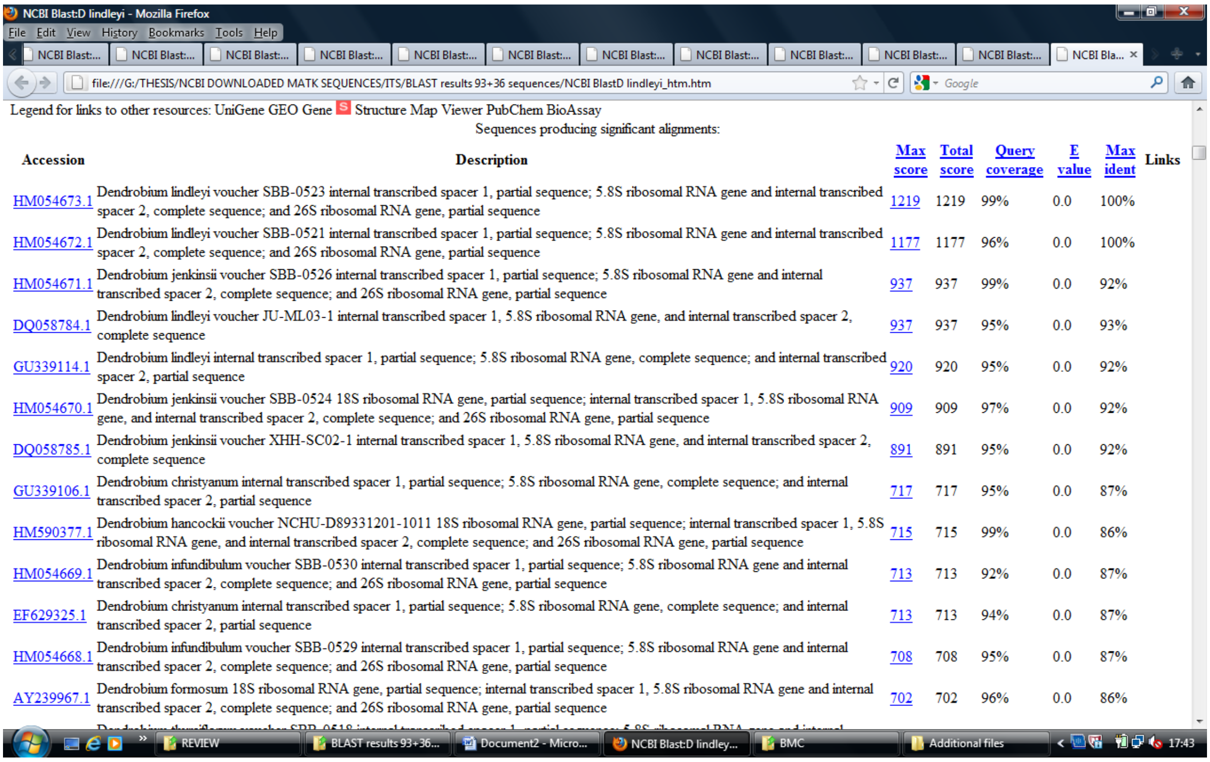

Legend for links to other resources: UniGene GEO Gene Structure Map Viewer PubChem BioAssay

Sequences producing significant alignments:

Accession	Description	Max score	Total score	Query coverage	E value	Max ident	Links
HM054673.1	Dendrobium lindleyi voucher SBB-0523 internal transcribed spacer 1, partial sequence; 5.8S ribosomal RNA gene and internal transcribed spacer 2, complete sequence; and 26S ribosomal RNA gene, partial sequence	1219	1219	99%	0.0	100%	
HM054672.1	Dendrobium lindleyi voucher SBB-0521 internal transcribed spacer 1, partial sequence; 5.8S ribosomal RNA gene and internal transcribed spacer 2, complete sequence; and 26S ribosomal RNA gene, partial sequence	1177	1177	96%	0.0	100%	
HM054671.1	Dendrobium jenkinsii voucher SBB-0526 internal transcribed spacer 1, partial sequence; 5.8S ribosomal RNA gene and internal transcribed spacer 2, complete sequence; and 26S ribosomal RNA gene, partial sequence	937	937	99%	0.0	92%	
DQ058784.1	Dendrobium lindleyi voucher JU-ML03-1 internal transcribed spacer 1, 5.8S ribosomal RNA gene, and internal transcribed spacer 2, complete sequence	937	937	95%	0.0	93%	
GU339114.1	Dendrobium lindleyi internal transcribed spacer 1, partial sequence; 5.8S ribosomal RNA gene, complete sequence; and internal transcribed spacer 2, partial sequence	920	920	95%	0.0	92%	
HM054670.1	Dendrobium jenkinsii voucher SBB-0524 18S ribosomal RNA gene, partial sequence; internal transcribed spacer 1, 5.8S ribosomal RNA gene, and internal transcribed spacer 2, complete sequence; and 26S ribosomal RNA gene, partial sequence	909	909	97%	0.0	92%	
DQ058785.1	Dendrobium jenkinsii voucher XHH-SC02-1 internal transcribed spacer 1, 5.8S ribosomal RNA gene, and internal transcribed spacer 2, complete sequence	891	891	95%	0.0	92%	
GU339106.1	Dendrobium christyanum internal transcribed spacer 1, partial sequence; 5.8S ribosomal RNA gene, complete sequence; and internal transcribed spacer 2, partial sequence	717	717	95%	0.0	87%	
HM590377.1	Dendrobium hancockii voucher NCHU-D89331201-1011 18S ribosomal RNA gene, partial sequence; internal transcribed spacer 1, 5.8S ribosomal RNA gene, and internal transcribed spacer 2, complete sequence; and 26S ribosomal RNA gene, partial sequence	715	715	99%	0.0	86%	
HM054669.1	Dendrobium infundibulum voucher SBB-0530 internal transcribed spacer 1, partial sequence; 5.8S ribosomal RNA gene and internal transcribed spacer 2, complete sequence; and 26S ribosomal RNA gene, partial sequence	713	713	92%	0.0	87%	
EF629325.1	Dendrobium christyanum internal transcribed spacer 1, partial sequence; 5.8S ribosomal RNA gene, complete sequence; and internal transcribed spacer 2, partial sequence	713	713	94%	0.0	87%	
HM054668.1	Dendrobium infundibulum voucher SBB-0529 internal transcribed spacer 1, partial sequence; 5.8S ribosomal RNA gene and internal transcribed spacer 2, complete sequence; and 26S ribosomal RNA gene, partial sequence	708	708	95%	0.0	87%	
AY239967.1	Dendrobium formosum 18S ribosomal RNA gene, partial sequence; internal transcribed spacer 1, 5.8S ribosomal RNA gene and internal transcribed spacer 2, complete sequence; and 26S ribosomal RNA gene, partial sequence	702	702	96%	0.0	86%	

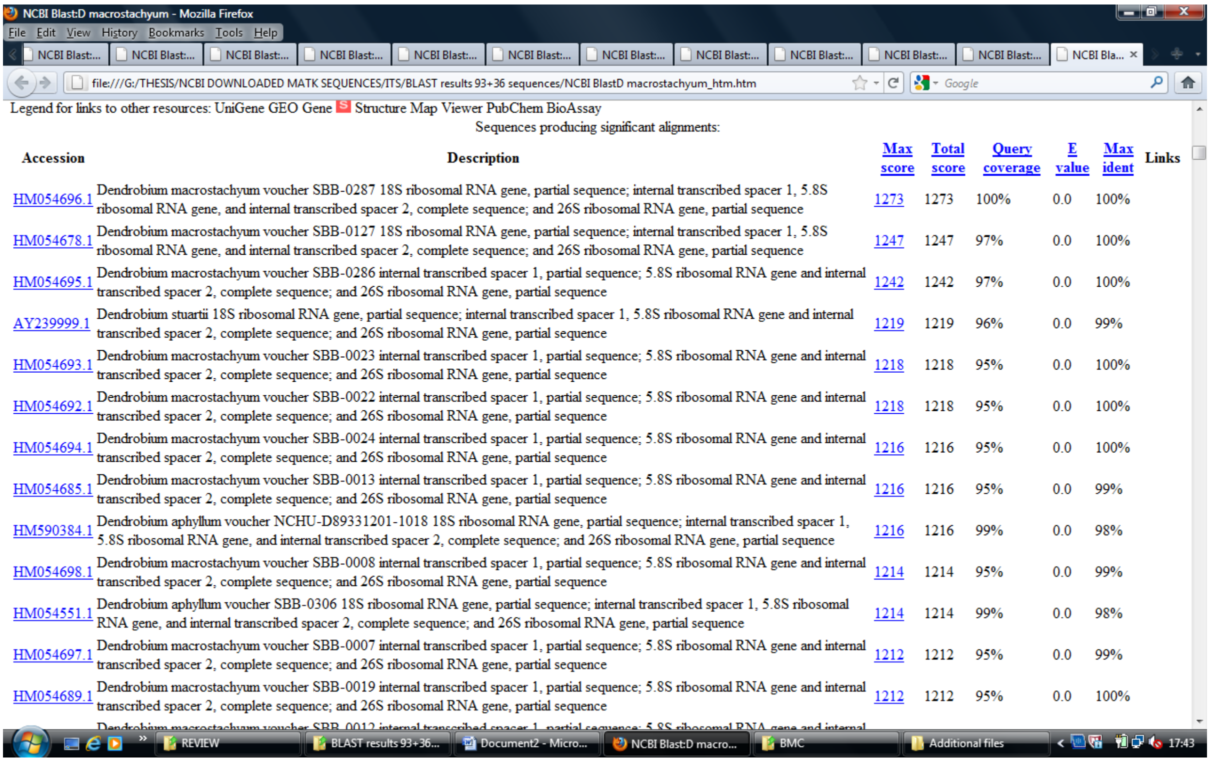

Legend for links to other resources: UniGene GEO Gene Structure Map Viewer PubChem BioAssay

Sequences producing significant alignments:

Accession	Description	Max score	Total score	Query coverage	E value	Max ident	Links
HM054696.1	Dendrobium macrostachyum voucher SBB-0287 18S ribosomal RNA gene, partial sequence; internal transcribed spacer 1, 5.8S ribosomal RNA gene, and internal transcribed spacer 2, complete sequence; and 26S ribosomal RNA gene, partial sequence	1273	1273	100%	0.0	100%	
HM054678.1	Dendrobium macrostachyum voucher SBB-0127 18S ribosomal RNA gene, partial sequence; internal transcribed spacer 1, 5.8S ribosomal RNA gene, and internal transcribed spacer 2, complete sequence; and 26S ribosomal RNA gene, partial sequence	1247	1247	97%	0.0	100%	
HM054695.1	Dendrobium macrostachyum voucher SBB-0286 internal transcribed spacer 1, partial sequence; 5.8S ribosomal RNA gene and internal transcribed spacer 2, complete sequence; and 26S ribosomal RNA gene, partial sequence	1242	1242	97%	0.0	100%	
AY239999.1	Dendrobium stuartii 18S ribosomal RNA gene, partial sequence; internal transcribed spacer 1, 5.8S ribosomal RNA gene and internal transcribed spacer 2, complete sequence; and 26S ribosomal RNA gene, partial sequence	1219	1219	96%	0.0	99%	
HM054693.1	Dendrobium macrostachyum voucher SBB-0023 internal transcribed spacer 1, partial sequence; 5.8S ribosomal RNA gene and internal transcribed spacer 2, complete sequence; and 26S ribosomal RNA gene, partial sequence	1218	1218	95%	0.0	100%	
HM054692.1	Dendrobium macrostachyum voucher SBB-0022 internal transcribed spacer 1, partial sequence; 5.8S ribosomal RNA gene and internal transcribed spacer 2, complete sequence; and 26S ribosomal RNA gene, partial sequence	1218	1218	95%	0.0	100%	
HM054694.1	Dendrobium macrostachyum voucher SBB-0024 internal transcribed spacer 1, partial sequence; 5.8S ribosomal RNA gene and internal transcribed spacer 2, complete sequence; and 26S ribosomal RNA gene, partial sequence	1216	1216	95%	0.0	100%	
HM054685.1	Dendrobium macrostachyum voucher SBB-0013 internal transcribed spacer 1, partial sequence; 5.8S ribosomal RNA gene and internal transcribed spacer 2, complete sequence; and 26S ribosomal RNA gene, partial sequence	1216	1216	95%	0.0	99%	
HM590384.1	Dendrobium aphyllum voucher NCHU-D89331201-1018 18S ribosomal RNA gene, partial sequence; internal transcribed spacer 1, 5.8S ribosomal RNA gene, and internal transcribed spacer 2, complete sequence; and 26S ribosomal RNA gene, partial sequence	1216	1216	99%	0.0	98%	
HM054698.1	Dendrobium macrostachyum voucher SBB-0008 internal transcribed spacer 1, partial sequence; 5.8S ribosomal RNA gene and internal transcribed spacer 2, complete sequence; and 26S ribosomal RNA gene, partial sequence	1214	1214	95%	0.0	99%	
HM054551.1	Dendrobium aphyllum voucher SBB-0306 18S ribosomal RNA gene, partial sequence; internal transcribed spacer 1, 5.8S ribosomal RNA gene, and internal transcribed spacer 2, complete sequence; and 26S ribosomal RNA gene, partial sequence	1214	1214	99%	0.0	98%	
HM054697.1	Dendrobium macrostachyum voucher SBB-0007 internal transcribed spacer 1, partial sequence; 5.8S ribosomal RNA gene and internal transcribed spacer 2, complete sequence; and 26S ribosomal RNA gene, partial sequence	1212	1212	95%	0.0	99%	
HM054689.1	Dendrobium macrostachyum voucher SBB-0019 internal transcribed spacer 1, partial sequence; 5.8S ribosomal RNA gene and internal transcribed spacer 2, complete sequence; and 26S ribosomal RNA gene, partial sequence	1212	1212	95%	0.0	100%	

NCBI Blast:D microbulbon - Mozilla Firefox

file:///G:/THESIS/NCBI DOWNLOADED MATK SEQUENCES/ITS/BLAST results 93+36 sequences/NCBI BlastD microbulbon_htm.htm

Legend for links to other resources: UniGene GEO Gene S Structure Map Viewer PubChem BioAssay

Sequences producing significant alignments:

Accession	Description	Max score	Total score	Query coverage	E value	Max ident	Links
HM054702.1	Dendrobium microbulbon voucher SBB-0335 18S ribosomal RNA gene, partial sequence; internal transcribed spacer 1, 5.8S ribosomal RNA gene, and internal transcribed spacer 2, complete sequence; and 26S ribosomal RNA gene, partial sequence	1254	1254	100%	0.0	100%	
HM054701.1	Dendrobium microbulbon voucher SBB-0334 18S ribosomal RNA gene, partial sequence; internal transcribed spacer 1, 5.8S ribosomal RNA gene, and internal transcribed spacer 2, complete sequence; and 26S ribosomal RNA gene, partial sequence	1253	1253	99%	0.0	100%	
HM054703.1	Dendrobium microbulbon voucher SBB-0337 18S ribosomal RNA gene, partial sequence; internal transcribed spacer 1, 5.8S ribosomal RNA gene, and internal transcribed spacer 2, complete sequence; and 26S ribosomal RNA gene, partial sequence >gb|HM054704.1| Dendrobium microbulbon voucher SBB-0338 18S ribosomal RNA gene, partial sequence; internal transcribed spacer 1, 5.8S ribosomal RNA gene, and internal transcribed spacer 2, complete sequence; and 26S ribosomal RNA gene, partial sequence >gb|HM054707.1| Dendrobium microbulbon voucher SBB-0341 18S ribosomal RNA gene, partial sequence; internal transcribed spacer 1, 5.8S ribosomal RNA gene, and internal transcribed spacer 2, complete sequence; and 26S ribosomal RNA gene, partial sequence	1253	1253	99%	0.0	100%	
HM054700.1	Dendrobium microbulbon voucher SBB-0333 18S ribosomal RNA gene, partial sequence; internal transcribed spacer 1, 5.8S ribosomal RNA gene, and internal transcribed spacer 2, complete sequence; and 26S ribosomal RNA gene, partial sequence	1251	1251	99%	0.0	100%	
HM054706.1	Dendrobium microbulbon voucher SBB-0340 18S ribosomal RNA gene, partial sequence; internal transcribed spacer 1, 5.8S ribosomal RNA gene, and internal transcribed spacer 2, complete sequence; and 26S ribosomal RNA gene, partial sequence	1247	1247	99%	0.0	100%	
HM054708.1	Dendrobium microbulbon voucher SBB-0342 18S ribosomal RNA gene, partial sequence; internal transcribed spacer 1, 5.8S ribosomal RNA gene, and internal transcribed spacer 2, complete sequence; and 26S ribosomal RNA gene, partial sequence	1247	1247	99%	0.0	100%	
HM054705.1	Dendrobium microbulbon voucher SBB-0339 internal transcribed spacer 1, partial sequence; 5.8S ribosomal RNA gene and internal transcribed spacer 2, complete sequence; and 26S ribosomal RNA gene, partial sequence	1229	1229	97%	0.0	100%	
DQ058800.1	Dendrobium minutiflorum voucher MH-SM0311-1 internal transcribed spacer 1, 5.8S ribosomal RNA gene, and internal transcribed spacer 2, complete sequence	473	473	87%	5e-130	81%	
DQ058797.1	Dendrobium strongylanthum voucher SC-SM0311-1 internal transcribed spacer 1, 5.8S ribosomal RNA gene, and internal transcribed spacer 2, complete sequence	455	455	70%	2e-124	84%	
DQ058798.1	Dendrobium monticola voucher ZN-SC02-1 internal transcribed spacer 1, 5.8S ribosomal RNA gene, and internal transcribed spacer 2, complete sequence >gb|DQ058799.1| Dendrobium monticola voucher ZN-SM0311-1 internal transcribed spacer 1, 5.8S ribosomal RNA gene, and internal transcribed spacer 2, complete sequence >gb|FJ384739.1| Dendrobium strongylanthum internal transcribed spacer 1, partial sequence; 5.8S ribosomal RNA gene, complete sequence; and internal transcribed spacer 2, partial sequence >gb|GU339107.1| Dendrobium strongylanthum internal transcribed spacer 1, partial sequence; 5.8S ribosomal RNA gene, complete sequence; and internal	455	455	70%	2e-124	84%	

NCBI Blast:D moschatum - Mozilla Firefox

file:///G:/THESIS/NCBI DOWNLOADED MATK SEQUENCES/ITS/BLAST results 93+36 sequences/NCBI BlastD moschatum_htm.htm

Legend for links to other resources: UniGene GEO Gene S Structure Map Viewer PubChem BioAssay

Sequences producing significant alignments:

Accession	Description	Max score	Total score	Query coverage	E value	Max ident	Links
HM054714.1	Dendrobium moschatum voucher SBB-0568 internal transcribed spacer 1, partial sequence; 5.8S ribosomal RNA gene and internal transcribed spacer 2, complete sequence; and 26S ribosomal RNA gene, partial sequence	1238	1238	99%	0.0	100%	
HM054710.1	Dendrobium moschatum voucher SBB-0322 internal transcribed spacer 1, partial sequence; 5.8S ribosomal RNA gene and internal transcribed spacer 2, complete sequence; and 26S ribosomal RNA gene, partial sequence	1230	1230	98%	0.0	100%	
HM054711.1	Dendrobium moschatum voucher SBB-0511 internal transcribed spacer 1, partial sequence; 5.8S ribosomal RNA gene and internal transcribed spacer 2, complete sequence; and 26S ribosomal RNA gene, partial sequence >gb|HM054712.1| Dendrobium moschatum voucher SBB-0512 internal transcribed spacer 1, partial sequence; 5.8S ribosomal RNA gene and internal transcribed spacer 2, complete sequence; and 26S ribosomal RNA gene, partial sequence >gb|HM054716.1| Dendrobium moschatum voucher SBB-0630 internal transcribed spacer 1, partial sequence; 5.8S ribosomal RNA gene and internal transcribed spacer 2, complete sequence; and 26S ribosomal RNA gene, partial sequence	1230	1230	98%	0.0	100%	
HM054715.1	Dendrobium moschatum voucher SBB-0569 internal transcribed spacer 1, partial sequence; 5.8S ribosomal RNA gene and internal transcribed spacer 2, complete sequence; and 26S ribosomal RNA gene, partial sequence	1229	1229	98%	0.0	100%	
AY239983.1	Dendrobium moschatum 18S ribosomal RNA gene, partial sequence; internal transcribed spacer 1, 5.8S ribosomal RNA gene and internal transcribed spacer 2, complete sequence; and 26S ribosomal RNA gene, partial sequence	1192	1192	97%	0.0	99%	
HM054713.1	Dendrobium moschatum voucher SBB-0567 internal transcribed spacer 1, partial sequence; 5.8S ribosomal RNA gene, complete sequence; and internal transcribed spacer 2, partial sequence	1173	1173	94%	0.0	100%	
HM054709.1	Dendrobium moschatum voucher SBB-0026 internal transcribed spacer 1, partial sequence; 5.8S ribosomal RNA gene and internal transcribed spacer 2, complete sequence; and 26S ribosomal RNA gene, partial sequence	1164	1164	93%	0.0	100%	
EF629326.1	Dendrobium moschatum internal transcribed spacer 1, partial sequence; 5.8S ribosomal RNA gene, complete sequence; and internal transcribed spacer 2, partial sequence	1164	1164	95%	0.0	99%	
HM590387.1	Dendrobium clavatum voucher NCHU-D89331201-1021 18S ribosomal RNA gene, partial sequence; internal transcribed spacer 1, 5.8S ribosomal RNA gene, and internal transcribed spacer 2, complete sequence; and 26S ribosomal RNA gene, partial sequence	898	898	100%	0.0	90%	
AF362043.1	Dendrobium aurantiacum var. denneanum isolate Xishuangbanna Yunnan internal transcribed spacer 1, partial sequence; 5.8S ribosomal RNA gene, complete sequence; and internal transcribed spacer 2, partial sequence >gb|EU840702.1| Dendrobium aurantiacum var. denneanum internal transcribed spacer 1, 5.8S ribosomal RNA gene, and internal transcribed spacer 2, complete sequence >gb|FJ384732.1| Dendrobium aurantiacum var. denneanum strain D4 internal transcribed spacer 1, partial sequence; 5.8S ribosomal RNA gene, complete sequence; and internal transcribed spacer 2, partial sequence	865	865	95%	0.0	91%	

NCBI Blast:D nobile - Mozilla Firefox

file:///G:/THESIS/NCBI DOWNLOADED MATK SEQUENCES/ITS/BLAST results 93+36 sequences/NCBI BlastD nobile_htm.htm

Legend for links to other resources: UniGene GEO Gene Structure Map Viewer PubChem BioAssay

Sequences producing significant alignments:

Accession	Description	Max score	Total score	Query coverage	E value	Max ident	Links
HM054717.1	Dendrobium nobile voucher SBB-0016 internal transcribed spacer 1, partial sequence; 5.8S ribosomal RNA gene and internal transcribed spacer 2, complete sequence; and 26S ribosomal RNA gene, partial sequence	1232	1232	100%	0.0	99%	
HM590382.1	Dendrobium nobile voucher NCHU-D89331201-1016 18S ribosomal RNA gene, partial sequence; internal transcribed spacer 1, 5.8S ribosomal RNA gene, and internal transcribed spacer 2, complete sequence; and 26S ribosomal RNA gene, partial sequence	1182	1182	100%	0.0	98%	
HM590371.1	Dendrobium linawianum voucher NCHU-D89331201-1005 18S ribosomal RNA gene, partial sequence; internal transcribed spacer 1, 5.8S ribosomal RNA gene, and internal transcribed spacer 2, complete sequence; and 26S ribosomal RNA gene, partial sequence	1171	1171	100%	0.0	98%	
HM590381.1	Dendrobium hercoglossum voucher NCHU-D89331201-1015 18S ribosomal RNA gene, partial sequence; internal transcribed spacer 1, 5.8S ribosomal RNA gene, and internal transcribed spacer 2, complete sequence; and 26S ribosomal RNA gene, partial sequence	1160	1160	100%	0.0	97%	
HM590372.1	Dendrobium aduncum voucher NCHU-D89331201-1006 18S ribosomal RNA gene, partial sequence; internal transcribed spacer 1, 5.8S ribosomal RNA gene, and internal transcribed spacer 2, complete sequence; and 26S ribosomal RNA gene, partial sequence	1153	1153	100%	0.0	97%	
AF362028.1	Dendrobium nobile isolate Lijiang Yunnan internal transcribed spacer 1, partial sequence; 5.8S ribosomal RNA gene, complete sequence; and internal transcribed spacer 2, partial sequence	1118	1118	94%	0.0	98%	
AF362037.1	Dendrobium nobile isolate Hainan internal transcribed spacer 1, partial sequence; 5.8S ribosomal RNA gene, complete sequence; and internal transcribed spacer 2, partial sequence >gb|FJ384728.1| Dendrobium nobile strain J2 internal transcribed spacer 1, partial sequence; 5.8S ribosomal RNA gene, complete sequence; and internal transcribed spacer 2, partial sequence >gb|FJ804134.1| Dendrobium nobile isolate 17-7 internal transcribed spacer 1, partial sequence; 5.8S ribosomal RNA gene, complete sequence; and internal transcribed spacer 2, partial sequence >gb|FJ804135.1| Dendrobium nobile isolate 18-3 internal transcribed spacer 1, partial sequence; 5.8S ribosomal RNA gene, complete sequence; and internal transcribed spacer 2, partial sequence >gb|FJ804136.1| Dendrobium nobile isolate 21-4 internal transcribed spacer 1, partial sequence; 5.8S ribosomal RNA gene, complete sequence; and internal transcribed spacer 2, partial sequence	1118	1118	94%	0.0	98%	
EU003117.1	Dendrobium linawianum voucher CMU DL(C) 0615 internal transcribed spacer 1, partial sequence; 5.8S ribosomal RNA gene, complete sequence; and internal transcribed spacer 2, partial sequence	1112	1112	94%	0.0	98%	
EU003115.1	Dendrobium linawianum voucher CMC DL 0301 internal transcribed spacer 1, partial sequence; 5.8S ribosomal RNA gene, complete sequence; and internal transcribed spacer 2, partial sequence	1107	1107	94%	0.0	98%	
AF362039.1	Dendrobium nobile isolate Guangxi internal transcribed spacer 1, partial sequence; 5.8S ribosomal RNA gene, complete sequence; and internal transcribed spacer 2, partial sequence	1107	1107	94%	0.0	98%	
	Dendrobium nobile isolate Guizhou internal transcribed spacer 1, partial sequence; 5.8S ribosomal RNA gene, complete sequence; and internal transcribed spacer 2, partial sequence >gb|AF362046.1| Dendrobium nobile isolate Sichuan internal transcribed spacer 1, partial sequence; 5.8S						

NCBI Blast:D nutantiflorum - Mozilla Firefox

file:///G:/THESIS/NCBI DOWNLOADED MATK SEQUENCES/ITS/BLAST results 93+36 sequences/NCBI BlastD nutantiflorum_htm.htm

Legend for links to other resources: UniGene GEO Gene Structure Map Viewer PubChem BioAssay

Sequences producing significant alignments:

Accession	Description	Max score	Total score	Query coverage	E value	Max ident	Links
HM054718.1	Dendrobium nutantiflorum voucher SBB-0032 internal transcribed spacer 1, partial sequence; 5.8S ribosomal RNA gene and internal transcribed spacer 2, complete sequence; and 26S ribosomal RNA gene, partial sequence	1216	1216	97%	0.0	100%	
AY239967.1	Dendrobium formosum 18S ribosomal RNA gene, partial sequence; internal transcribed spacer 1, 5.8S ribosomal RNA gene and internal transcribed spacer 2, complete sequence; and 26S ribosomal RNA gene, partial sequence	808	808	97%	0.0	89%	
GU339106.1	Dendrobium christyanum internal transcribed spacer 1, partial sequence; 5.8S ribosomal RNA gene, complete sequence; and internal transcribed spacer 2, partial sequence	798	798	94%	0.0	89%	
HM054628.1	Dendrobium draconis voucher SBB-0546 18S ribosomal RNA gene, partial sequence; internal transcribed spacer 1, 5.8S ribosomal RNA gene, and internal transcribed spacer 2, complete sequence; and 26S ribosomal RNA gene, partial sequence	797	797	100%	0.0	88%	
EF629325.1	Dendrobium christyanum internal transcribed spacer 1, partial sequence; 5.8S ribosomal RNA gene, complete sequence; and internal transcribed spacer 2, partial sequence	793	793	94%	0.0	89%	
HM054758.1	Dendrobium thyrsiflorum voucher SBB-0518 internal transcribed spacer 1, partial sequence; 5.8S ribosomal RNA gene and internal transcribed spacer 2, complete sequence; and 26S ribosomal RNA gene, partial sequence	782	782	97%	0.0	88%	
AF362027.1	Dendrobium cariniferum internal transcribed spacer 1, partial sequence; 5.8S ribosomal RNA gene, complete sequence; and internal transcribed spacer 2, partial sequence	778	778	94%	0.0	88%	
AF362030.1	Dendrobium williamsonii internal transcribed spacer 1, partial sequence; 5.8S ribosomal RNA gene, complete sequence; and internal transcribed spacer 2, partial sequence	778	778	94%	0.0	88%	
AY240001.1	Dendrobium thyrsiflorum 18S ribosomal RNA gene, partial sequence; internal transcribed spacer 1, 5.8S ribosomal RNA gene and internal transcribed spacer 2, complete sequence; and 26S ribosomal RNA gene, partial sequence	773	773	97%	0.0	88%	
EU592015.1	Dendrobium bellatulum internal transcribed spacer 1, partial sequence; 5.8S ribosomal RNA gene, complete sequence; and internal transcribed spacer 2, partial sequence	771	771	94%	0.0	88%	
AY239965.1	Dendrobium ellipsophyllum 18S ribosomal RNA gene, partial sequence; internal transcribed spacer 1, 5.8S ribosomal RNA gene and internal transcribed spacer 2, complete sequence; and 26S ribosomal RNA gene, partial sequence	769	769	97%	0.0	87%	
HM054669.1	Dendrobium infundibulum voucher SBB-0530 internal transcribed spacer 1, partial sequence; 5.8S ribosomal RNA gene and internal transcribed spacer 2, complete sequence; and 26S ribosomal RNA gene, partial sequence	767	767	94%	0.0	88%	
HM054668.1	Dendrobium infundibulum voucher SBB-0529 internal transcribed spacer 1, partial sequence; 5.8S ribosomal RNA gene and internal transcribed spacer 2, complete sequence; and 26S ribosomal RNA gene, partial sequence	767	767	93%	0.0	88%	

NCBI Blast:D ochreatum - Mozilla Firefox

file:///G:/THESIS/NCBI DOWNLOADED MATK SEQUENCES/ITS/BLAST results 93+36 sequences/NCBI BlastD ochreatum_htm.htm

Legend for links to other resources: UniGene GEO Gene Structure Map Viewer PubChem BioAssay

Sequences producing significant alignments:

Accession	Description	Max score	Total score	Query coverage	E value	Max ident	Links
HM054720.1	Dendrobium ochreatum voucher SBB-0506 18S ribosomal RNA gene, partial sequence; internal transcribed spacer 1, 5.8S ribosomal RNA gene, and internal transcribed spacer 2, complete sequence; and 26S ribosomal RNA gene, partial sequence	1194	1194	100%	0.0	99%	
HM054719.1	Dendrobium ochreatum voucher SBB-0505 18S ribosomal RNA gene, partial sequence; internal transcribed spacer 1 and 5.8S ribosomal RNA gene, complete sequence; and internal transcribed spacer 2, partial sequence	1131	1131	94%	0.0	99%	
HM590377.1	Dendrobium hancockii voucher NCHU-D89331201-1011 18S ribosomal RNA gene, partial sequence; internal transcribed spacer 1, 5.8S ribosomal RNA gene, and internal transcribed spacer 2, complete sequence; and 26S ribosomal RNA gene, partial sequence	708	708	100%	0.0	86%	
DQ058787.1	Dendrobium hancockii voucher XY-GZ03-1 internal transcribed spacer 1, 5.8S ribosomal RNA gene, and internal transcribed spacer 2, complete sequence	675	675	97%	0.0	85%	
AF362036.1	Dendrobium brymerianum internal transcribed spacer 1, partial sequence; 5.8S ribosomal RNA gene, complete sequence; and internal transcribed spacer 2, partial sequence	675	675	97%	0.0	85%	
AF362025.1	Dendrobium hancockii internal transcribed spacer 1, partial sequence; 5.8S ribosomal RNA gene, complete sequence; and internal transcribed spacer 2, partial sequence	675	675	97%	0.0	85%	
EU477500.1	Dendrobium brymerianum internal transcribed spacer 1, partial sequence; 5.8S ribosomal RNA gene and internal transcribed spacer 2, complete sequence; and 28S ribosomal RNA gene, partial sequence	671	671	97%	0.0	85%	
AF362026.1	Dendrobium salaccense internal transcribed spacer 1, partial sequence; 5.8S ribosomal RNA gene, complete sequence; and internal transcribed spacer 2, partial sequence	669	669	97%	0.0	85%	
FJ428221.1	Dendrobium brymerianum internal transcribed spacer 1, partial sequence; 5.8S ribosomal RNA gene, complete sequence; and internal transcribed spacer 2, partial sequence	664	664	97%	0.0	85%	
AF362040.1	Dendrobium aurantiacum var. denneanum internal transcribed spacer 1, partial sequence; 5.8S ribosomal RNA gene, complete sequence; and internal transcribed spacer 2, partial sequence >gb|FJ384731.1| Dendrobium aurantiacum var. denneanum strain D3 internal transcribed spacer 1, partial sequence; 5.8S ribosomal RNA gene, complete sequence; and internal transcribed spacer 2, partial sequence	656	656	97%	0.0	85%	
EU477506.1	Dendrobium harveyanum internal transcribed spacer 1, partial sequence; 5.8S ribosomal RNA gene and internal transcribed spacer 2, complete sequence; and 28S ribosomal RNA gene, partial sequence	649	649	97%	0.0	85%	
FJ384742.1	Dendrobium densiflorum internal transcribed spacer 1, partial sequence; 5.8S ribosomal RNA gene, complete sequence; and internal transcribed spacer 2, partial sequence	647	647	97%	0.0	85%	
AF362038.1	Dendrobium hancockii isolate Henan internal transcribed spacer 1, partial sequence; 5.8S ribosomal RNA gene, complete sequence; and internal transcribed spacer 2, partial sequence >gb|FJ384726.1| Dendrobium hancockii strain X2 internal transcribed spacer 1, partial sequence; 5.8S [illegible]	645	645	97%	0.0	85%	

NCBI Blast:D ovatum - Mozilla Firefox

file:///G:/THESIS/NCBI DOWNLOADED MATK SEQUENCES/ITS/BLAST results 93+36 sequences/NCBI BlastD ovatum_htm.htm

Legend for links to other resources: UniGene GEO Gene Structure Map Viewer PubChem BioAssay

Sequences producing significant alignments:

Accession	Description	Max score	Total score	Query coverage	E value	Max ident	Links
HM054729.1	Dendrobium ovatum voucher SBB-0423 internal transcribed spacer 1, partial sequence; 5.8S ribosomal RNA gene and internal transcribed spacer 2, complete sequence; and 26S ribosomal RNA gene, partial sequence	1258	1258	98%	0.0	100%	
HM054721.1	Dendrobium ovatum voucher SBB-0001 18S ribosomal RNA gene, partial sequence; internal transcribed spacer 1, 5.8S ribosomal RNA gene, and internal transcribed spacer 2, complete sequence; and 26S ribosomal RNA gene, partial sequence	1254	1254	98%	0.0	99%	
HM054726.1	Dendrobium ovatum voucher SBB-0420 18S ribosomal RNA gene, partial sequence; internal transcribed spacer 1, 5.8S ribosomal RNA gene, and internal transcribed spacer 2, complete sequence; and 26S ribosomal RNA gene, partial sequence	1253	1253	97%	0.0	100%	
HM054734.1	Dendrobium ovatum voucher SBB-0005 18S ribosomal RNA gene, partial sequence; internal transcribed spacer 1, 5.8S ribosomal RNA gene, and internal transcribed spacer 2, complete sequence; and 26S ribosomal RNA gene, partial sequence	1240	1240	97%	0.0	99%	
HM054733.1	Dendrobium ovatum voucher SBB-0004 18S ribosomal RNA gene, partial sequence; internal transcribed spacer 1, 5.8S ribosomal RNA gene, and internal transcribed spacer 2, complete sequence; and 26S ribosomal RNA gene, partial sequence	1236	1236	97%	0.0	99%	
HM054728.1	Dendrobium ovatum voucher SBB-0422 18S ribosomal RNA gene, partial sequence; internal transcribed spacer 1, 5.8S ribosomal RNA gene, and internal transcribed spacer 2, complete sequence; and 26S ribosomal RNA gene, partial sequence	1236	1236	97%	0.0	99%	
HM054730.1	Dendrobium ovatum voucher SBB-0424 internal transcribed spacer 1, partial sequence; 5.8S ribosomal RNA gene and internal transcribed spacer 2, complete sequence; and 26S ribosomal RNA gene, partial sequence	1232	1232	96%	0.0	100%	
HM054731.1	Dendrobium ovatum voucher SBB-0425 internal transcribed spacer 1, partial sequence; 5.8S ribosomal RNA gene and internal transcribed spacer 2, complete sequence; and 26S ribosomal RNA gene, partial sequence	1232	1232	96%	0.0	100%	
HM054732.1	Dendrobium ovatum voucher SBB-0426 internal transcribed spacer 1, partial sequence; 5.8S ribosomal RNA gene and internal transcribed spacer 2, complete sequence; and 26S ribosomal RNA gene, partial sequence	1229	1229	95%	0.0	100%	
HM054724.1	Dendrobium ovatum voucher SBB-0418 internal transcribed spacer 1, partial sequence; 5.8S ribosomal RNA gene and internal transcribed spacer 2, complete sequence; and 26S ribosomal RNA gene, partial sequence	1229	1229	96%	0.0	99%	
HM054725.1	Dendrobium ovatum voucher SBB-0419 internal transcribed spacer 1, partial sequence; 5.8S ribosomal RNA gene and internal transcribed spacer 2, complete sequence; and 26S ribosomal RNA gene, partial sequence	1229	1229	96%	0.0	99%	
HM054727.1	Dendrobium ovatum voucher SBB-0421 internal transcribed spacer 1, partial sequence; 5.8S ribosomal RNA gene and internal transcribed spacer 2, complete sequence; and 26S ribosomal RNA gene, partial sequence	1229	1229	96%	0.0	99%	
HM054722.1	Dendrobium ovatum voucher SBB-0002 internal transcribed spacer 1, partial sequence; 5.8S ribosomal RNA gene and internal transcribed spacer 2, complete sequence; and 26S ribosomal RNA gene, partial sequence	1229	1229	96%	0.0	99%	
[illegible]	[illegible]	[illegible]	[illegible]	[illegible]	[illegible]	[illegible]	

NCBI Blast:D parishii - Mozilla Firefox

file:///G:/THESIS/NCBI DOWNLOADED MATK SEQUENCES/ITS/BLAST results 93+36 sequences/NCBI BlastD parishii_htm.htm

Legend for links to other resources: UniGene GEO Gene S Structure Map Viewer PubChem BioAssay

Sequences producing significant alignments:

Accession	Description	Max score	Total score	Query coverage	E value	Max ident	Links
HM054735.1	Dendrobium parishii voucher SBB-0527 internal transcribed spacer 1, partial sequence; 5.8S ribosomal RNA gene and internal transcribed spacer 2, complete sequence; and 26S ribosomal RNA gene, partial sequence	1229	1229	98%	0.0	100%	
HM054736.1	Dendrobium parishii voucher SBB-0528 18S ribosomal RNA gene, partial sequence; internal transcribed spacer 1, 5.8S ribosomal RNA gene, and internal transcribed spacer 2, complete sequence; and 26S ribosomal RNA gene, partial sequence	1225	1225	98%	0.0	100%	
HM590378.1	Dendrobium parishii voucher NCHU-D89331201-1012 18S ribosomal RNA gene, partial sequence; internal transcribed spacer 1, 5.8S ribosomal RNA gene, and internal transcribed spacer 2, complete sequence; and 26S ribosomal RNA gene, partial sequence	1218	1218	99%	0.0	99%	
HM590369.1	Dendrobium moniliforme voucher NCHU-D89331201-1003 18S ribosomal RNA gene, partial sequence; internal transcribed spacer 1, 5.8S ribosomal RNA gene, and internal transcribed spacer 2, complete sequence; and 26S ribosomal RNA gene, partial sequence	1218	1218	99%	0.0	99%	
EU477499.1	Dendrobium anosmum internal transcribed spacer 1, partial sequence; 5.8S ribosomal RNA gene and internal transcribed spacer 2, complete sequence; and 28S ribosomal RNA gene, partial sequence	1149	1149	94%	0.0	99%	
EU121417.1	Dendrobium parishii internal transcribed spacer 1, 5.8S ribosomal RNA gene, and internal transcribed spacer 2, complete sequence	1138	1138	94%	0.0	98%	
HM054751.1	Dendrobium primulinum voucher SBB-0228 18S ribosomal RNA gene, partial sequence; internal transcribed spacer 1, 5.8S ribosomal RNA gene, and internal transcribed spacer 2, complete sequence; and 26S ribosomal RNA gene, partial sequence	1009	1009	99%	0.0	93%	
HM054750.1	Dendrobium primulinum voucher SBB-0225 18S ribosomal RNA gene, partial sequence; internal transcribed spacer 1, 5.8S ribosomal RNA gene, and internal transcribed spacer 2, complete sequence; and 26S ribosomal RNA gene, partial sequence	1007	1007	99%	0.0	93%	
HM054755.1	Dendrobium primulinum voucher SBB-0277 18S ribosomal RNA gene, partial sequence; internal transcribed spacer 1, 5.8S ribosomal RNA gene, and internal transcribed spacer 2, complete sequence; and 26S ribosomal RNA gene, partial sequence	1007	1007	99%	0.0	93%	
HM054754.1	Dendrobium primulinum voucher SBB-0276 internal transcribed spacer 1, partial sequence; 5.8S ribosomal RNA gene and internal transcribed spacer 2, complete sequence; and 26S ribosomal RNA gene, partial sequence	1002	1002	97%	0.0	94%	
HM054756.1	Dendrobium primulinum voucher SBB-0278 internal transcribed spacer 1, partial sequence; 5.8S ribosomal RNA gene and internal transcribed spacer 2, complete sequence; and 26S ribosomal RNA gene, partial sequence	996	996	97%	0.0	94%	
HM054752.1	Dendrobium primulinum voucher SBB-0273 internal transcribed spacer 1, partial sequence; 5.8S ribosomal RNA gene and internal transcribed spacer 2, complete sequence; and 26S ribosomal RNA gene, partial sequence	996	996	97%	0.0	94%	
HM054748.1	Dendrobium primulinum voucher SBB-0171 internal transcribed spacer 1, partial sequence; 5.8S ribosomal RNA gene and internal transcribed spacer 2, complete sequence; and 26S ribosomal RNA gene, partial sequence	996	996	97%	0.0	94%	
HM054747.1	Dendrobium primulinum voucher SBB-0170 18S ribosomal RNA gene, partial sequence; internal transcribed spacer 1, 5.8S ribosomal [illegible]	996	996	98%	0.0	93%	

NCBI Blast:D peguanum - Mozilla Firefox

file:///G:/THESIS/NCBI DOWNLOADED MATK SEQUENCES/ITS/BLAST results 93+36 sequences/NCBI BlastD peguanum_htm.htm

Legend for links to other resources: UniGene GEO Gene S Structure Map Viewer PubChem BioAssay

Sequences producing significant alignments:

Accession	Description	Max score	Total score	Query coverage	E value	Max ident	Links
HM054739.1	Dendrobium peguanum voucher SBB-0398 18S ribosomal RNA gene, partial sequence; internal transcribed spacer 1, 5.8S ribosomal RNA gene, and internal transcribed spacer 2, complete sequence; and 26S ribosomal RNA gene, partial sequence	1256	1256	99%	0.0	100%	
HM054746.1	Dendrobium peguanum voucher SBB-0406 18S ribosomal RNA gene, partial sequence; internal transcribed spacer 1, 5.8S ribosomal RNA gene, and internal transcribed spacer 2, complete sequence; and 26S ribosomal RNA gene, partial sequence	1249	1249	98%	0.0	99%	
HM054742.1	Dendrobium peguanum voucher SBB-0401 18S ribosomal RNA gene, partial sequence; internal transcribed spacer 1, 5.8S ribosomal RNA gene, and internal transcribed spacer 2, complete sequence; and 26S ribosomal RNA gene, partial sequence	1249	1249	98%	0.0	100%	
HM054745.1	Dendrobium peguanum voucher SBB-0405 18S ribosomal RNA gene, partial sequence; internal transcribed spacer 1, 5.8S ribosomal RNA gene, and internal transcribed spacer 2, complete sequence; and 26S ribosomal RNA gene, partial sequence	1245	1245	98%	0.0	99%	
HM054743.1	Dendrobium peguanum voucher SBB-0402 18S ribosomal RNA gene, partial sequence; internal transcribed spacer 1, 5.8S ribosomal RNA gene, and internal transcribed spacer 2, complete sequence; and 26S ribosomal RNA gene, partial sequence	1245	1245	98%	0.0	99%	
HM054737.1	Dendrobium peguanum voucher SBB-0319 18S ribosomal RNA gene, partial sequence; internal transcribed spacer 1, 5.8S ribosomal RNA gene, and internal transcribed spacer 2, complete sequence; and 26S ribosomal RNA gene, partial sequence	1245	1245	98%	0.0	100%	
HM054740.1	Dendrobium peguanum voucher SBB-0399 18S ribosomal RNA gene, partial sequence; internal transcribed spacer 1, 5.8S ribosomal RNA gene, and internal transcribed spacer 2, complete sequence; and 26S ribosomal RNA gene, partial sequence	1240	1240	98%	0.0	99%	
HM054741.1	Dendrobium peguanum voucher SBB-0400 internal transcribed spacer 1, partial sequence; 5.8S ribosomal RNA gene and internal transcribed spacer 2, complete sequence; and 26S ribosomal RNA gene, partial sequence	1219	1219	96%	0.0	99%	
HM054738.1	Dendrobium peguanum voucher SBB-0397 internal transcribed spacer 1, 5.8S ribosomal RNA gene, and internal transcribed spacer 2, complete sequence; and 26S ribosomal RNA gene, partial sequence	1188	1188	94%	0.0	99%	
HM054744.1	Dendrobium peguanum voucher SBB-0404 18S ribosomal RNA gene, partial sequence; internal transcribed spacer 1, 5.8S ribosomal RNA gene, and internal transcribed spacer 2, complete sequence; and 26S ribosomal RNA gene, partial sequence	1182	1182	93%	0.0	99%	
DQ058800.1	Dendrobium minutiflorum voucher MH-SM0311-1 internal transcribed spacer 1, 5.8S ribosomal RNA gene, and internal transcribed spacer 2, complete sequence	800	800	92%	0.0	89%	
DQ058797.1	Dendrobium strongylanthum voucher SC-SM0311-1 internal transcribed spacer 1, 5.8S ribosomal RNA gene, and internal transcribed spacer 2, complete sequence	797	797	92%	0.0	89%	
[illegible]	Dendrobium monticola voucher ZN-SC02-1 internal transcribed spacer 1, 5.8S ribosomal RNA gene, and internal transcribed spacer 2, complete sequence >gb|DQ058799.1| Dendrobium monticola voucher ZN-SM0311-1 internal transcribed spacer 1, 5.8S ribosomal RNA gene, and internal transcribed spacer 2, complete sequence >gb|FJ384739.1| Dendrobium strongylanthum internal transcribed spacer 1, [illegible]	[illegible]	[illegible]	[illegible]	[illegible]	[illegible]	

NCBI Blast:D primulinum - Mozilla Firefox

file:///G:/THESIS/NCBI DOWNLOADED MATK SEQUENCES/ITS/BLAST results 93+36 sequences/NCBI BlastD primulinum_htm.htm

Legend for links to other resources: UniGene GEO Gene Structure Map Viewer PubChem BioAssay

Sequences producing significant alignments:

Accession	Description	Max score	Total score	Query coverage	E value	Max ident	Links
HM054750.1	Dendrobium primulinum voucher SBB-0225 18S ribosomal RNA gene, partial sequence; internal transcribed spacer 1, 5.8S ribosomal RNA gene, and internal transcribed spacer 2, complete sequence; and 26S ribosomal RNA gene, partial sequence	1242	1242	99%	0.0	100%	
HM054751.1	Dendrobium primulinum voucher SBB-0238 18S ribosomal RNA gene, partial sequence; internal transcribed spacer 1, 5.8S ribosomal RNA gene, and internal transcribed spacer 2, complete sequence; and 26S ribosomal RNA gene, partial sequence	1242	1242	99%	0.0	100%	
HM054755.1	Dendrobium primulinum voucher SBB-0277 18S ribosomal RNA gene, partial sequence; internal transcribed spacer 1, 5.8S ribosomal RNA gene, and internal transcribed spacer 2, complete sequence; and 26S ribosomal RNA gene, partial sequence	1242	1242	99%	0.0	100%	
HM054754.1	Dendrobium primulinum voucher SBB-0276 internal transcribed spacer 1, partial sequence; 5.8S ribosomal RNA gene and internal transcribed spacer 2, complete sequence; and 26S ribosomal RNA gene, partial sequence	1227	1227	98%	0.0	100%	
HM054747.1	Dendrobium primulinum voucher SBB-0170 18S ribosomal RNA gene, partial sequence; internal transcribed spacer 1, 5.8S ribosomal RNA gene, and internal transcribed spacer 2, complete sequence; and 26S ribosomal RNA gene, partial sequence	1227	1227	98%	0.0	100%	
HM054756.1	Dendrobium primulinum voucher SBB-0278 internal transcribed spacer 1, partial sequence; 5.8S ribosomal RNA gene and internal transcribed spacer 2, complete sequence; and 26S ribosomal RNA gene, partial sequence	1225	1225	97%	0.0	100%	
HM054748.1	Dendrobium primulinum voucher SBB-0171 internal transcribed spacer 1, partial sequence; 5.8S ribosomal RNA gene and internal transcribed spacer 2, complete sequence; and 26S ribosomal RNA gene, partial sequence	1223	1223	97%	0.0	100%	
HM054752.1	Dendrobium primulinum voucher SBB-0273 internal transcribed spacer 1, partial sequence; 5.8S ribosomal RNA gene and internal transcribed spacer 2, complete sequence; and 26S ribosomal RNA gene, partial sequence	1218	1218	97%	0.0	100%	
HM054749.1	Dendrobium primulinum voucher SBB-0224 internal transcribed spacer 1, partial sequence; 5.8S ribosomal RNA gene and internal transcribed spacer 2, complete sequence; and 26S ribosomal RNA gene, partial sequence	1216	1216	97%	0.0	100%	
HM054753.1	Dendrobium primulinum voucher SBB-0274 internal transcribed spacer 1, partial sequence; 5.8S ribosomal RNA gene and internal transcribed spacer 2, complete sequence; and 26S ribosomal RNA gene, partial sequence	1210	1210	96%	0.0	100%	
HM054757.1	Dendrobium primulinum voucher SBB-0533 internal transcribed spacer 1, partial sequence; 5.8S ribosomal RNA gene and internal transcribed spacer 2, complete sequence; and 26S ribosomal RNA gene, partial sequence	1203	1203	96%	0.0	100%	
AF362913.1	Dendrobium primulinum internal transcribed spacer 1, partial sequence; 5.8S ribosomal RNA gene, complete sequence; and internal transcribed spacer 2, partial sequence	1153	1153	94%	0.0	99%	
HM590378.1	Dendrobium parishii voucher NCHU-D89331201-1012 18S ribosomal RNA gene, partial sequence; internal transcribed spacer 1, 5.8S ribosomal RNA gene, and internal transcribed spacer 2, complete sequence; and 26S ribosomal RNA gene, partial sequence	1009	1009	99%	0.0	93%	
[illegible]	[illegible]						

NCBI Blast:D thyrsiflorum - Mozilla Firefox

file:///G:/THESIS/NCBI DOWNLOADED MATK SEQUENCES/ITS/BLAST results 93+36 sequences/NCBI BlastD thyrsiflorum_htm.htm

Legend for links to other resources: UniGene GEO Gene Structure Map Viewer PubChem BioAssay

Sequences producing significant alignments:

Accession	Description	Max score	Total score	Query coverage	E value	Max ident	Links
HM054758.1	Dendrobium thyrsiflorum voucher SBB-0518 internal transcribed spacer 1, partial sequence; 5.8S ribosomal RNA gene and internal transcribed spacer 2, complete sequence; and 26S ribosomal RNA gene, partial sequence	1214	1214	100%	0.0	99%	
AY240001.1	Dendrobium thyrsiflorum 18S ribosomal RNA gene, partial sequence; internal transcribed spacer 1, 5.8S ribosomal RNA gene and internal transcribed spacer 2, complete sequence; and 26S ribosomal RNA gene, partial sequence	1194	1194	99%	0.0	99%	
AF362029.1	Dendrobium densiflorum internal transcribed spacer 1, partial sequence; 5.8S ribosomal RNA gene, complete sequence; and internal transcribed spacer 2, partial sequence	1175	1175	96%	0.0	99%	
AF362032.1	Dendrobium thyrsiflorum internal transcribed spacer 1, partial sequence; 5.8S ribosomal RNA gene, complete sequence; and internal transcribed spacer 2, partial sequence	1175	1175	96%	0.0	99%	
FJ384733.1	Dendrobium thyrsiflorum internal transcribed spacer 1, partial sequence; 5.8S ribosomal RNA gene, complete sequence; and internal transcribed spacer 2, partial sequence	1170	1170	96%	0.0	99%	
HM054760.1	Dendrobium thyrsiflorum voucher SBB-0520 internal transcribed spacer 1, partial sequence; 5.8S ribosomal RNA gene and internal transcribed spacer 2, complete sequence; and 26S ribosomal RNA gene, partial sequence	1168	1168	96%	0.0	99%	
DQ058786.1	Dendrobium densiflorum voucher MH-GZ03-1 internal transcribed spacer 1, 5.8S ribosomal RNA gene, and internal transcribed spacer 2, complete sequence	1168	1168	96%	0.0	99%	
EU840699.1	Epigeneium nakaharaei internal transcribed spacer 1, 5.8S ribosomal RNA gene, and internal transcribed spacer 2, complete sequence	1164	1164	96%	0.0	99%	
HM054759.1	Dendrobium thyrsiflorum voucher SBB-0519 internal transcribed spacer 1, partial sequence; 5.8S ribosomal RNA gene, complete sequence; and internal transcribed spacer 2, partial sequence	1160	1160	95%	0.0	99%	
HM054626.1	Dendrobium densiflorum voucher SBB-0120 internal transcribed spacer 1, partial sequence; 5.8S ribosomal RNA gene and internal transcribed spacer 2, complete sequence; and 26S ribosomal RNA gene, partial sequence	1013	1013	98%	0.0	94%	
HM054630.1	Dendrobium farmeri voucher SBB-0538 internal transcribed spacer 1, partial sequence; 5.8S ribosomal RNA gene and internal transcribed spacer 2, complete sequence; and 26S ribosomal RNA gene, partial sequence	948	948	97%	0.0	93%	
HM054631.1	Dendrobium farmeri voucher SBB-0549 internal transcribed spacer 1, partial sequence; 5.8S ribosomal RNA gene and internal transcribed spacer 2, complete sequence; and 26S ribosomal RNA gene, partial sequence	948	948	97%	0.0	93%	
HM054627.1	Dendrobium densiflorum voucher SBB-0547 internal transcribed spacer 1, partial sequence; 5.8S ribosomal RNA gene, complete sequence; and internal transcribed spacer 2, partial sequence	939	939	92%	0.0	94%	
HM054629.1	Dendrobium farmeri voucher SBB-0537 internal transcribed spacer 1, partial sequence; 5.8S ribosomal RNA gene, complete sequence; and internal transcribed spacer 2, partial sequence	920	920	95%	0.0	93%	

NCBI Blast:D wardianum - Mozilla Firefox

Legend for links to other resources: UniGene GEO Gene Structure Map Viewer PubChem BioAssay

Sequences producing significant alignments:

Accession	Description	Max score	Total score	Query coverage	E value	Max ident	Links
DQ058789.1	Dendrobium wardianum voucher DBQ-JL04-01 internal transcribed spacer 1, 5.8S ribosomal RNA gene, and internal transcribed spacer 2, complete sequence	1133	1133	98%	0.0	98%	
HM054544.1	Dendrobium amoenum voucher SBB-0560 internal transcribed spacer 1, partial sequence; 5.8S ribosomal RNA gene and internal transcribed spacer 2, complete sequence; and 26S ribosomal RNA gene, partial sequence	1131	1131	100%	0.0	98%	
HM054538.1	Dendrobium amoenum voucher SBB-0140 internal transcribed spacer 1, partial sequence; 5.8S ribosomal RNA gene and internal transcribed spacer 2, complete sequence; and 26S ribosomal RNA gene, partial sequence	1127	1127	100%	0.0	98%	
HM054539.1	Dendrobium amoenum voucher SBB-0142 internal transcribed spacer 1, partial sequence; 5.8S ribosomal RNA gene and internal transcribed spacer 2, complete sequence; and 26S ribosomal RNA gene, partial sequence	1125	1125	100%	0.0	98%	
HM054540.1	Dendrobium amoenum voucher SBB-0247 internal transcribed spacer 1, partial sequence; 5.8S ribosomal RNA gene and internal transcribed spacer 2, complete sequence; and 26S ribosomal RNA gene, partial sequence	1125	1125	100%	0.0	98%	
HM054534.1	Dendrobium amoenum voucher SBB-0135 internal transcribed spacer 1, partial sequence; 5.8S ribosomal RNA gene and internal transcribed spacer 2, complete sequence; and 26S ribosomal RNA gene, partial sequence	1125	1125	99%	0.0	98%	
HM054536.1	Dendrobium amoenum voucher SBB-0138 internal transcribed spacer 1, partial sequence; 5.8S ribosomal RNA gene and internal transcribed spacer 2, complete sequence; and 26S ribosomal RNA gene, partial sequence	1125	1125	99%	0.0	98%	
HM054537.1	Dendrobium amoenum voucher SBB-0139 internal transcribed spacer 1, partial sequence; 5.8S ribosomal RNA gene and internal transcribed spacer 2, complete sequence; and 26S ribosomal RNA gene, partial sequence	1123	1123	99%	0.0	98%	
HM054535.1	Dendrobium amoenum voucher SBB-0137 internal transcribed spacer 1, partial sequence; 5.8S ribosomal RNA gene and internal transcribed spacer 2, complete sequence; and 26S ribosomal RNA gene, partial sequence	1123	1123	99%	0.0	98%	
HM054546.1	Dendrobium amoenum voucher SBB-0576 internal transcribed spacer 1, partial sequence; 5.8S ribosomal RNA gene and internal transcribed spacer 2, complete sequence; and 26S ribosomal RNA gene, partial sequence	1112	1112	97%	0.0	98%	
HM054545.1	Dendrobium amoenum voucher SBB-0575 internal transcribed spacer 1, partial sequence; 5.8S ribosomal RNA gene and internal transcribed spacer 2, complete sequence; and 26S ribosomal RNA gene, partial sequence	1107	1107	97%	0.0	98%	
HM054543.1	Dendrobium amoenum voucher SBB-0029 internal transcribed spacer 1, partial sequence; 5.8S ribosomal RNA gene and internal transcribed spacer 2, complete sequence; and 26S ribosomal RNA gene, partial sequence	1105	1105	97%	0.0	98%	
HM054541.1	Dendrobium amoenum voucher SBB-0248 internal transcribed spacer 1, partial sequence; 5.8S ribosomal RNA gene and internal transcribed spacer 2, complete sequence; and 26S ribosomal RNA gene, partial sequence	1103	1103	97%	0.0	98%	
[illegible]	Dendrobium gratiosissimum voucher BO-JL0401-1 internal transcribed spacer 1, 5.8S ribosomal RNA gene, and internal transcribed spacer 2 [illegible]	[illegible]	[illegible]	[illegible]	[illegible]	[illegible]	
