# Supplementary figures and images for: The loci recommended as universal barcodes for plants on the basis of floristic studies may not work with congeneric species as exemplified by DNA barcoding of Dendrobium species
Source: BMC Res Notes. 2012 Jan 19;5:42. doi: 10.1186/1756-0500-5-42 (PMC3292824; doi:10.1186/1756-0500-5-42)

## Slide 1
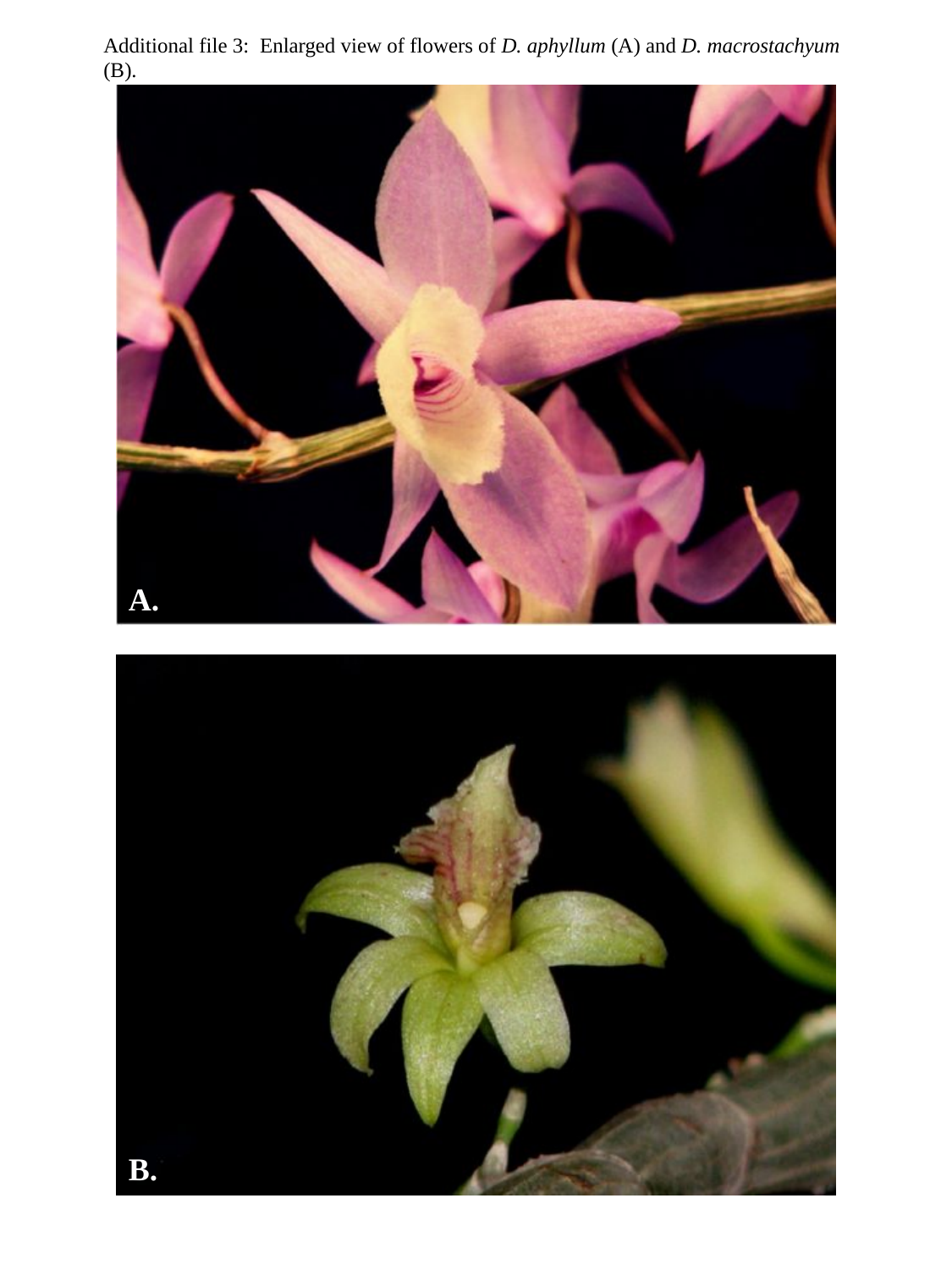

Additional file 3: Enlarged view of flowers of D. aphyllum (A) and D. macrostachyum (B).
A.
B.

Supplement: Additional file 3 — Enlarged view of flowers of D. aphyllum (A) and D. macrostachyum (B). [file 1756-0500-5-42-S3.PPT]
