# Supplementary material for: The loci recommended as universal barcodes for plants on the basis of floristic studies may not work with congeneric species as exemplified by DNA barcoding of Dendrobium species
Source: BMC Res Notes. 2012 Jan 19;5:42. doi: 10.1186/1756-0500-5-42 (PMC3292824; doi:10.1186/1756-0500-5-42)
Supplement: Additional file 4 — Accession numbers of sequences of five loci downloaded from the GenBank. [file 1756-0500-5-42-S4.DOC]

**Additional file 4 – Accession numbers of sequences of five loci downloaded from GenBank.**

ITS sequences of *Dendrobium* species downloaded from GenBank.

| **S. No.** | **Species Name** | **Accession No.** |
| --- | --- | --- |
| 1. | *Dendrobium acinaciforme* | AF362034.1 |
| 2. | *Dendrobium aemulum* | EU430372.1 |
| 3. | *Dendrobium albosanguineum* | EU477498.1 |
| 4. | *Dendrobium amethystoglossum* | AY239952.1 |
| 5. | *Dendrobium aurantiacum* | HM590375.1 |
| 6. | *Dendrobium bifalce* | EU430373.1 |
| 7. | *Dendrobium bracteosum* | AY239954.1 |
| 8. | *Dendrobium brymerianum* | AF362036.1 |
| 9. | *Dendrobium callitrophilum* | EU430374.1 |
| 10. | *Dendrobium camptocentrum* | AY239955.1 |
| 11. | *Dendrobium canaliculatum* | EU430375.1 |
| 12. | *Dendrobium capituliflorum* | AY239956.1 |
| 13. | *Dendrobium cariniferum* | AY485710.1 |
| 14. | *Dendrobium carrii* | EU430376.1 |
| 15. | *Dendrobium chameleon* | HM590385.1 |
| 16. | *Dendrobium clavatum* | HM590387.1 |
| 17. | *Dendrobium confusum* | AY239961.1 |
| 18. | *Dendrobium crystallinum* | GU339116.1 |
| 19. | *Dendrobium cyanocentrum* | AY239964.1 |
| 20. | *Dendrobium dixanthum* | DQ058788.1 |
| 21. | *Dendrobium ellipsophyllum* | AY239965.1 |
| 22. | *Dendrobium epidendropsis* | AY485696.1 |
| 23. | *Dendrobium equitans* | HM590388.1 |
| 24. | *Dendrobium erosum* | AY485706.1 |
| 25. | *Dendrobium exile* | AF362024.1 |
| 26. | *Dendrobium fairchildae* | AY239966.1 |
| 27. | *Dendrobium findleyanum* | EU477504.1 |
| 28. | *Dendrobium finniganense* | EU430378.1 |
| 29. | *Dendrobium formosum* | AY239967.1 |
| 30. | *Dendrobium fulgidum* | AY239968.1 |
| 31. | *Dendrobium funiushanense* | AF479761.1 |
| 32. | *Dendrobium furcatopedicellatum* | AF521611.1 |
| 33. | *Dendrobium gibsonii* | GU339105.1 |
| 34. | *Dendrobium goldfinchii* | AY239969.1 |
| 35. | *Dendrobium goldschmidtianum* | AY239970.1 |
| 36. | *Dendrobium govidjoae* | AY239971.1 |
| 37. | *Dendrobium gracilicaule* | EU430382.1 |
| 38. | *Dendrobium hancockii* | HM590377.1 |
| 39. | *Dendrobium harveyanum* | EU477506.1 |
| 40. | *Dendrobium henryi* | EF629323.1 |
| 41. | *Dendrobium huoshanense* | HM590368.1 |
| 42. | *Dendrobium indivisum* | AY239972.1 |
| 43. | *Dendrobium inflatum* | AY239973.1 |
| 44. | *Dendrobium ionopus* | AY239974.1 |
| 45. | *Dendrobium jonesii* | EU430383.1 |
| 46. | *Dendrobium junceum* | AY239975.1 |
| 47. | *Dendrobium kingianum* | EU430385.1 |
| 48. | *Dendrobium lancifolium* | AY239976.1 |
| 49. | *Dendrobium lawesii* | AY239977.1 |
| 50. | *Dendrobium leonis* | AY239978.1 |
| 51. | *Dendrobium leptocladum* | HM590373.1 |
| 52. | *Dendrobium lituiflorum* | AF355571.1 |
| 53. | *Dendrobium loddigesii* | HM590374.1 |
| 54. | *Dendrobium lohohense* | AF363024.1 |
| 55. | *Dendrobium macrophyllum* | AY239979.1 |
| 56. | *Dendrobium minutiflorum* | DQ058800.1 |
| 57. | *Dendrobium miyakei* | HM590386.1 |
| 58. | *Dendrobium mohlianum* | AY239980.1 |
| 59. | *Dendrobium monophyllum* | EU430387.1 |
| 60. | *Dendrobium moorei* | EU430388.1 |
| 61. | *Dendrobium morrisonii* | AY239982.1 |
| 62. | *Dendrobium nindii* | AY239985.1 |
| 63. | *Dendrobium nothofagicola* | AY239986.1 |
| 64. | *Dendrobium papilio* | AY239987.1 |
| 65. | *Dendrobium parciflorum* | EF629324.1 |
| 66. | *Dendrobium pendulum* | GU339115.1 |
| 67. | *Dendrobium philippinense* | AY239988.1 |
| 68. | *Dendrobium quadrangulare* | AY239989.1 |
| 69. | *Dendrobium racemosum* | EU430389.1 |
| 70. | *Dendrobium rhododioides* | AY239991.1 |
| 71. | *Dendrobium salaccense* | AF362026.1 |
| 72. | *Dendrobium sanguinolentum* | AY239992.1 |
| 73. | *Dendrobium schoeninum* | EU430390.1 |
| 74. | *Dendrobium secundum* | AY239993.1 |
| 75. | *Dendrobium senile* | EU477509.1 |
| 76. | *Dendrobium sinuatum* | AY239995.1 |
| 77. | *Dendrobium smillieae* | AY239996.1 |
| 78. | *Dendrobium somai* | HM590380.1 |
| 79. | *Dendrobium speciosum* | AY239998.1 |
| 80. | *Dendrobium stuartii* | AY239999.1 |
| 81. | *Dendrobium stuposum* | GU339104.1 |
| 82. | *Dendrobium subuliferum* | AY240000.1 |
| 83. | *Dendrobium sulcatum* | EU477510.1 |
| 84. | *Dendrobium terminale* | DQ058801.1 |
| 85. | *Dendrobium tetragonum* | EU430401.1 |
| 86. | *Dendrobium tortile* | EU477511.1 |
| 87. | *Dendrobium tosaense* | HM590367.1 |
| 88. | *Dendrobium trigonopus* | FJ384741.1 |
| 89. | *Dendrobium truncatum* | AY240002.1 |
| 90. | *Dendrobium usterioides* | AY240003.1 |
| 91. | *Dendrobium victoriaereginae* | EU840694.1 |
| 92. | *Dendrobium violaceum* | AY240005.1 |
| 93. | *Dendrobium yeageri* | AY240006.1 |

*matK* sequences of *Dendrobium* species downloaded from GenBank.

| **S. No.** | **Species Name** | **Accession No.** |
| --- | --- | --- |
| 1. | *Dendrobium acinaciforme* | AF447067.1 |
| 2. | *Dendrobium aurantiacum* | FJ794057.1 |
| 3. | *Dendrobium bellatulum* | GU569964.1 |
| 4. | *Dendrobium brymerianum* | GU991357.1 |
| 5. | *Dendrobium capillipes* | AF447069.1 |
| 6. | *Dendrobium chryseum* | AF448861.1 |
| 7. | *Dendrobium crystallinum* | AF445447.1 |
| 8. | *Dendrobium dixanthum* | GU569960.1 |
| 9. | *Dendrobium findleyanum* | EF079348.1 |
| 10. | *Dendrobium flexicaule* | FJ794061.1 |
| 11. | *Dendrobium gibsonii* | FJ216637.1 |
| 12. | *Dendrobium gratiosissimum* | GU906224.1 |
| 13. | *Dendrobium hancockii* | FJ794051.1 |
| 14. | *Dendrobium harveyanum* | FJ216633.1 |
| 15. | *Dendrobium hercoglossum* | GU569963.1 |
| 16. | *Dendrobium hookerianum* | GU569961.1 |
| 17. | *Dendrobium kingianum* | AF263651.1 |
| 18. | *Dendrobium lituiflorum* | FJ216651.1 |
| 19. | *Dendrobium loddigesii* | AF448864.2 |
| 20. | *Dendrobium minutiflorum* | GU569962.1 |
| 21. | *Dendrobium miyakei* | GU569965.1 |
| 22. | *Dendrobium moniliforme* | AB519774.1 |
| 23. | *Dendrobium officinale* | FJ794044.1 |
| 24. | *Dendrobium parciflorum* | FJ216667.1 |
| 25. | *Dendrobium pendulum* | GU991358.1 |
| 26. | *Dendrobium prasinum* | AJ310020.1 |
| 27. | *Dendrobium pulchellum* | AB519778.1 |
| 28. | *Dendrobium salaccense* | AF445451.1 |
| 29. | *Dendrobium scoriarum* | GU569959.1 |
| 30. | *Dendrobium stuposum* | FJ216645.1 |
| 31. | *Dendrobium tosaense* | AB519771.1 |
| 32. | *Dendrobium trigonopus* | FJ216634.1 |
| 33. | *Dendrobium williamsonii* | FJ216632.1 |

*rbcL* sequences of *Dendrobium* species downloaded from GenBank.

| **S. No.** | **Species Name** | **Accession No.** |
| --- | --- | --- |
| 1. | *Dendrobium acinaciforme* | FJ216578.1 |
| 2. | *Dendrobium aggregatum* | AF074145.1 |
| 3. | *Dendrobium brymerianum* | FJ216554.1 |
| 4. | *Dendrobium capillipes* | FJ216545.1 |
| 5. | *Dendrobium findlayanum* | FJ216558.1 |
| 6. | *Dendrobium gibsonii* | FJ216551.1 |
| 7. | *Dendrobium gratiosissimum* | FJ216557.1 |
| 8. | *Dendrobium hancockii* | FJ216569.1 |
| 9. | *Dendrobium harveyanum* | FJ216548.1 |
| 10. | *Dendrobium lituiflorum* | FJ216565.1 |
| 11. | *Dendrobium loddigesii* | FJ216573.1 |
| 12. | *Dendrobium moniliforme* | AB519787.1 |
| 13. | *Dendrobium officinale* | FJ216567.1 |
| 14. | *Dendrobium parciflorum* | FJ216579.1 |
| 15. | *Dendrobium pendulum* | FJ216555.1 |
| 16. | *Dendrobium pulchellum* | AB519789.1 |
| 17. | *Dendrobium stuposum* | FJ216559.1 |
| 18. | *Dendrobium tosaense* | AB519782.1 |
| 19. | *Dendrobium trigonopus* | FJ216549.1 |
| 20. | *Dendrobium williamsonii* | FJ216547.1 |

*rpoB* sequences of *Dendrobium* species downloaded from GenBank.

| **S. No.** | **Species Name** | **Accession No.** |
| --- | --- | --- |
| 1. | *Dendrobium acinaciforme* | FJ216536.1 |
| 2. | *Dendrobium brymerianum* | FJ216524.1 |
| 3. | *Dendrobium capillipes* | EU553926.1 |
| 4. | *Dendrobium crystallinum* | EU553936.1 |
| 5. | *Dendrobium findlayanum* | FJ216526.1 |
| 6. | *Dendrobium gibsonii* | FJ216522.1 |
| 7. | *Dendrobium gratiosissimum* | FJ216525.1 |
| 8. | *Dendrobium hancockii* | FJ216531.1 |
| 9. | *Dendrobium harveyanum* | EU553928.1 |
| 10. | *Dendrobium lituiflorum* | FJ216529.1 |
| 11. | *Dendrobium loddigesii* | EU553942.1 |
| 12. | *Dendrobium moniliforme* | EU553939.1 |
| 13. | *Dendrobium officinale* | EU553938.1 |
| 14. | *Dendrobium parciflorum* | FJ216537.1 |
| 15. | *Dendrobium pendulum* | EU553931.1 |
| 16. | *Dendrobium stuposum* | EU553933.1 |
| 17. | *Dendrobium trigonopus* | EU553929.1 |
| 18. | *Dendrobium williamsonii* | EU553927.1 |

*rpoC1* sequences of *Dendrobium* species downloaded from GenBank.

| **S. No.** | **Species Name** | **Accession No.** |
| --- | --- | --- |
| 1. | *Dendrobium brymerianum* | FJ216496.1 |
| 2. | *Dendrobium capillipes* | EU553943.1 |
| 3. | *Dendrobium crystallinum* | EU553953.1 |
| 4. | *Dendrobium findlayanum* | FJ216498.1 |
| 5. | *Dendrobium gibsonii* | FJ216494.1 |
| 6. | *Dendrobium gratiosissimum* | FJ216497.1 |
| 7. | *Dendrobium hancockii* | FJ216503.1 |
| 8. | *Dendrobium harveyanum* | EU553945.1 |
| 9. | *Dendrobium lituiflorum* | FJ216501.1 |
| 10. | *Dendrobium loddigesii* | EU553959.1 |
| 11. | *Dendrobium moniliforme* | EU553956.1 |
| 12. | *Dendrobium officinale* | EU553955.1 |
| 13. | *Dendrobium parciflorum* | FJ216510.1 |
| 14. | *Dendrobium pendulum* | EU553948.1 |
| 15. | *Dendrobium stuposum* | EU553950.1 |
| 16. | *Dendrobium trigonopus* | EU553946.1 |
| 17. | *Dendrobium williamsonii* | EU553944.1 |
